# Ecological partitioning and diversity in tropical planktonic foraminifera

**DOI:** 10.1186/1471-2148-12-54

**Published:** 2012-04-16

**Authors:** Heidi A Seears, Kate F Darling, Christopher M Wade

**Affiliations:** 1 School of Biology, University of Nottingham, Nottingham, UK; 2 School of GeoSciences and Institute of Evolutionary Biology, University of Edinburgh, Edinburgh, UK

## Abstract

**Background:**

Ecological processes are increasingly being viewed as an important mode of diversification in the marine environment, where the high dispersal potential of pelagic organisms, and a lack of absolute barriers to gene flow may limit the occurrence of allopatric speciation through vicariance. Here we focus on the potential role of ecological partitioning in the diversification of a widely distributed group of marine protists, the planktonic foraminifera. Sampling was conducted in the tropical Arabian Sea, during the southwest (summer) monsoon, when pronounced environmental conditions result in a strong disparity in temperature, salinity and productivity between distinct northern and southern water masses.

**Results:**

We uncovered extensive genetic diversity within the Arabian Sea planktonic foraminifera, identifying 13 morphospecies, represented by 20 distinct SSU rRNA genetic types. Several morphospecies/genetic types displayed non-random biogeographical distributions, partitioning between the northern and southern water masses, giving a strong indication of independent ecological adaptations.

**Conclusions:**

We propose sea-surface primary productivity as the main factor driving the geographical segregation of Arabian Sea planktonic foraminifera, during the SW monsoon, with variations in symbiotic associations possibly playing a role in the specific ecological adaptations observed. Our findings suggest that ecological partitioning could be contributing to the high levels of 'cryptic' genetic diversity observed within the planktonic foraminifera, and support the view that ecological processes may play a key role in the diversification of marine pelagic organisms.

## Background

The vast environment of the global ocean presents a challenge to the study of speciation. Marine planktonic microorganisms exist in huge populations, and carry a high passive dispersal potential [1]. With the presence of few physical barriers to gene flow in the open ocean, the occurrence of speciation through vicariant processes should be severely reduced, leading to large cosmopolitan, and genetically uniform populations [2,3]. Yet genetic data is increasingly highlighting the presence of "cryptic" diversity within many marine organisms [4-18], indicating that species diversity within the pelagic realm is significantly higher than suggested from many morphological taxonomies (reviewed in [2]). While vicariance clearly does play a role in the diversification of pelagic organisms [9,19], ecological speciation is increasingly being viewed as an important mode of diversification in the marine environment [2,20-22]. Here reproductive isolation can be achieved in the absence of intrinsic barriers to gene flow, by means of divergent selection for alternative environmental conditions or food resources [22-26]. Ecological partitioning has now been demonstrated to play a role in the speciation of a number of marine organisms [4,7-9,20,27-30], however, a great deal of further study will be necessary before the process can be fully understood.

Here we focus on the potential role of ecological partitioning in the diversification of the Planktonic Foraminifera, a highly diverse and widespread group of marine pelagic protists. The foraminifera are an important group, used frequently for paleoceanographic studies, and as a proxy for past climate change. Their utility is owed to an exceptional fossil record, spanning over 180 million years (Ma), and to the fact that individual "morphospecies" (identified by shell morphology) display characteristic environmental preferences, which are reflected in their spatial and temporal distribution in the oceans, and in the chemistry of their calcite shells. High levels of sequence variation have been found in the small subunit (SSU) ribosomal (r) RNA gene of the planktonic foraminiferal morphospecies, indicating the presence of numerous 'cryptic' genetic types [4-13,15], with mounting evidence indicating that these individual genetic types may display non-random geographical distributions, indicative of distinct ecological adaptations (ecotypes) [4-13,15].

Genetic surveys of the planktonic foraminifera have been undertaken over a wide range of oceanic water masses [4-6,8,10,11,13,22,31-36], though these ranged largely towards the mid to higher latitudes, with the species-rich tropics [37] remaining relatively under-sampled by comparison. Studies of high latitude planktonic foraminifera indicate that both vicariant [6-9,38,39] and ecological [4,7-9] processes may play a role in their diversification. Vicariance is implied by the presence of isolated or endemic genetic types within some morphospecies, likely resulting from the presence of physical barriers, such as the shallow Bering and Chukchi seas [8], or from oceanographic barriers such as the tropics and subtropics [4,8]. However, Darling and Wade [9] concluded that ecological constraints appeared to be major drivers of divergence in planktonic foraminifers in the high latitudes and anticipated that ecological factors would prove to be of prime importance in diversification in the mid to lower latitudes, where vertical niche partitioning is thought to be the principle factor controlling the distribution of foraminiferal morphospecies diversity [37].

For this study, the Arabian Sea was chosen as a tropical region of high priority. This unique marine environment is one of the richest marine biological areas in the world, and harbours a broad range of planktonic foraminiferal morphospecies [40]. It is subject to greater seasonal variability than any other ocean basin on the globe [41,42], with seasonally reversing monsoon winds inverting its circulation completely on a biannual basis [43,44]. In the winter months (November - February) prevailing winds progress in a northeasterly direction (the northeast monsoon), while in the summer months (June - September) they progress in a southwesterly direction (the southwest monsoon). During the summer monsoon, the formation of a major low-level air current, the Findlater jet [45], promotes upwelling in the coastal regions of Somalia, Yemen, and Oman [46], bringing nutrients into the euphotic zone. An enormous increase in primary productivity in the region results [47,48], transforming the normally nutrient poor (oligotrophic) waters of the northern Arabian Sea into one of the most productive (eutrophic) marine environments on Earth. At the same time, current circulations prevent the effect of this influx extending to the southern reaches of the Arabian Sea, which remain low in nutrients.

The Arabian Sea has been the focus of a number of studies linking physical oceanographic conditions to the distribution of planktonic foraminiferal morphospecies [49-52], however, this is the first time that the genetic diversity of the foraminifera within this region has been examined. This study investigates the biogeographical distributions of planktonic foraminiferal SSU rRNA genetic types in the Arabian Sea mixed layer during the SW (summer) monsoon, when pronounced environmental conditions lead to a distinct disparity in temperature, salinity and productivity between adjacent northern and southern water masses. Our results reveal non-random biogeographical distributions in several planktonic foraminiferal morphospecies/genetic types within the Arabian Sea during the SW monsoon, providing clear evidence of ecological partitioning.

## Methods

### Cruise track and oceanographic setting

**Figure 1:**
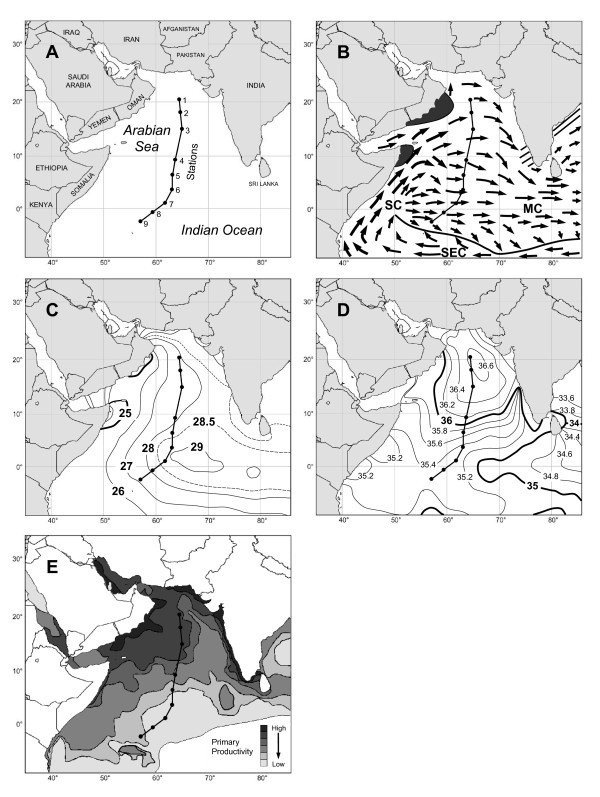
**Maps of the Arabian Sea showing the cruise transect and environmental conditions during the SW monsoon**. (A) CD148 cruise transect and stations, (B) Surface currents during August at the SW monsoon peak. Regions of intense seasonal upwelling (dark grey), weak sporadic upwelling (hatched) SC = Somali Current, MC = Monsoon Current, SEC = Southern Equatorial Current (modified from [50]), (C) Average sea-surface temperature for the SW Monsoon in July 2005 (adapted from [54]), (D) Average sea-surface salinity for the SW Monsoon in July 2005 (adapted from [55]), (E) Average primary productivity during the SW monsoon in July - September 1979 (adapted from Coastal Zone Colour Scanner composite images of the region, NASA Earth-Sun System Division, Earth Sciences (GES) Data and Information Services Center (DISC) Distributed Active Archive Center (DAAC)).

Specimens of planktonic foraminifera were collected at nine stations along a north/south cruise transect in the central Arabian Sea (20°22.81 N/64°29.36E-02°36.03 S/56°54.75E) during the summer monsoon of late June/July 2003 (Figure 1A; cruise Charles Darwin CD148, NERC). The oceanography of the Arabian Sea during the SW monsoon is shown in Figure 1B-E. Cyclonic surface circulation during the SW monsoon drives an eastward flowing monsoon current (MC) north of 10°S across the equatorial region (Figure 1B). A temperature gradient forms from west to east (Figure 1C) and there is a clear north/south differentiation in salinity (Figure 1D). Levels of primary productivity are elevated in the north of the Arabian Sea, but remain low in the oligotrophic south, with a water mass interface around stations 3-4 of the cruise transect (Figure 1E). Conductivity, temperature, depth (CTD) profiles from station 3 (15°01.11 N/65°00.02E) indicate that the mixed layer was 75 m deep at this position with a temperature of 28.5°C and a salinity of 36.7 psu, consistent with the maps in Figure 1C and 1D. The thermocline dipped steeply between 75 and 150 m (19°C) and then reduced its steepness coincident with a salinity minimum of 35.7 psu. Projections of mixed layer depth in July from Prasanna Kumar & Narvekar [53] indicate a mixed layer depth of ~50 m north of station 3, shoaling to a 40 m mixed layer depth south of station 5.

### Planktonic foraminiferal sampling

Samples were collected by pumping (5 m depth) from the ships' non-toxic water supply through a plankton screen (83 μm mesh) or by vertical net tow (0-100 and 0-200 m depth, 83 μm mesh) in waters with an average depth of 3,500 m. For genetic analysis, a representative sample of specimens was collected at each station. Individual specimens were identified using a stereomicroscope, and morphotype and cytoplasmic colouration were recorded by digital video imaging. Only adult specimens containing cytoplasm were selected for genetic analysis. These were crushed in a lysis buffer [56] and incubated for 1 hour at 60°C, before being transported to the lab where they were stored at -80°C. For assemblage assessment, bulk samples were taken at each station with the specimens either dried on slides directly or collected as bulk samples in ethanol. The preserved assemblages were then individually picked and placed onto micropalaeontological slides. The high incidence of small juveniles compared to the low incidence of mature specimens made identification too uncertain to carry out relative abundance counts along the transect, however, visual assessment of the bulk assemblages was undertaken.

### PCR amplification and sequencing

The PCR amplification of an approximately 1,000 bp region of the terminal 3' end of the foraminiferal SSU rRNA gene was carried out using a nested PCR approach. 3 μl of template were used in the first round of PCR, using primer C5 coupled with either primer 138 or NS8 (Table 1). 1 μl of product from the first round was used as the template in the second round, initially using primers 2082F and 2514R (Table 1) for the identification of genetic types. For sequences found to be novel to the Arabian Sea, an ~1,000 bp fragment was amplified using primers 2082F and 3014R (Table 1) for use in phylogenetic tree reconstruction. PCR amplification was performed using 1 unit of Taq polymerase (Qiagen) or Vent_R _polymerase (New England BioLabs) dependent upon success, with 200 μM each primer, 0.2 μM dNTPs, and 1.5 mM magnesium chloride in a 50 μl final volume. Thermal cycling (with a Perkin Elmer cycler) was performed with cycling parameters of 96°C for 2 mins, followed by 35 cycles of 96°C for 30 sec, 55°C for 30 sec and 72°C for 2 mins. Amplification products were purified from an agarose gel using a QIAprep spin miniprep (Qiagen). For taxa where direct sequencing was impossible due to the presence of multiple templates, cloning of the 1,000 bp fragment was carried out prior to sequencing using the TOPO TA^® ^method (Invitrogen). Both sense and antisense strands were sequenced directly on an Applied Biosystems 377 DNA sequencer using BigDye terminator cycle sequencing.

**Table 1:** SSU rRNA primer sequences

SSU primer	Sequence	Reference
**C5**	5'-GTAGTATGCACGCAAGTGTGA-3'	

**138**	5'-TGATCCTGCAGGTTCACCTAC-3'	[57]

**N8**	5'-TCCGCAGGTTCACCTACGGA-3'	[58]

**2082 F**	5'-TGAAACTTGAAGGAATTGACGGAAG-3'	Modified from NS5, [58]

**2514R**	5'-GGCATCACAGACCTGTTATTGCC-3'	Modified from NS6, [58]

**3014R**	5'-GTCGTAACAAGGCATCGGTAG-3'	

### Sequence analysis

Sequences were assembled using Gap4 in the Staden package [59] and then aligned manually within version 2.2 of the Genetic Data Environment (GDE) package [60]. 90 foraminiferal taxa were selected for use in the main phylogenetic analysis, including all species/genetic types obtained from the Arabian Sea, together with examples of every species and genetic type of planktonic foraminifera currently available in GenBank, plus a representative group of benthic foraminifera (1 per family in GenBank, see Additional file 1). Great care was taken during the process of sequence alignment and in the selection of sites for use in subsequent analyses. The unusually high levels of sequence divergence observed among foraminiferal taxa in their rRNA genes makes the selection of unambiguously aligned sites for use in phylogenetic analysis particularly challenging. To ensure the accuracy of our phylogenetic analyses we adopted a conservative approach, utilising only those sites for which positional homology across all taxa was certain. In total, 407 base pairs (bp) could be unambiguously aligned across all foraminiferal taxa. To improve resolution, additional phylogenies were constructed for four of the most common Arabian Sea morphospecies, thus allowing a greater number of unambiguously aligned sites to be recruited into the analyses (*Globigerinella siphonifera/Globigerinella calida *(668 bp), *Globigerinoides ruber/Globigerinoides conglobatus *(589 bp), *Globigerina bulloides *(669 bp)*, Turborotalita quinqueloba *(748 bp)). DNA sequence alignments are shown in Additional file 2 and Additional file 3.

Phylogenetic trees were constructed using Bayesian inference (BI) [61,62], maximum likelihood (ML) [63]), neighbour-joining (NJ) [64] and maximum parsimony (MP) [65]. BI was performed using the MrBayes (version 3.1.2) package [61] with multiple hits accounted for using a GTR + Γ model [66,67] and with the tree space explored using four chains of a Markov Chain Monte Carlo algorithm for 5 million generations (1 million for subset analyses), sampling every 100 generations. The run was terminated only after the Bayesian MCMC searches had reached a stationary phase (plateau), indicating convergence of the chain onto the target distribution, and a consensus tree built using the last 1000 trees (burn-in = 49001 samples for main tree, 9001 samples for subset analyses). ML analysis was undertaken within the Phyml package [68] using a GTR + Γ model [66,67], with parameters estimated within Phyml. NJ and MP analyses were performed using PAUP* (version 4.0d65; [69]). For NJ, distances were corrected using a GTR + Γ model [66,67] with the rate matrix, base frequencies, and shape parameter (α) of the gamma distribution (based on 16 rate categories) estimated using likelihood by iteration from an initial NJ tree. Bootstrap resampling [70] was undertaken using ML, NJ and MP with 1000 bootstrap replicates in order to assign support to particular branches within the tree. Bayesian posterior probabilities were obtained within MrBayes from the last 1000 trees generated.

The planktonic foraminiferal SSU rDNA sequences presented in this study are deposited in GenBank, accession numbers JQ799892 to JQ799900.

## Results

363 specimens of planktonic foraminifera were collected from 8 stations along a cruise transect in the Arabian Sea during the summer monsoon of 2003 (Figure 1A). Small subunit rRNA gene sequences were successfully amplified for 213 individual specimens. Examination of the SSU rDNA sequences revealed high levels of genetic diversity within the Arabian Sea mixed layer planktonic foraminiferal population, with 20 different genetic types being recognised from 13 different morphospecies

### Phylogenetic placement of the Arabian Sea foraminiferal genetic types

**Figure 2:**
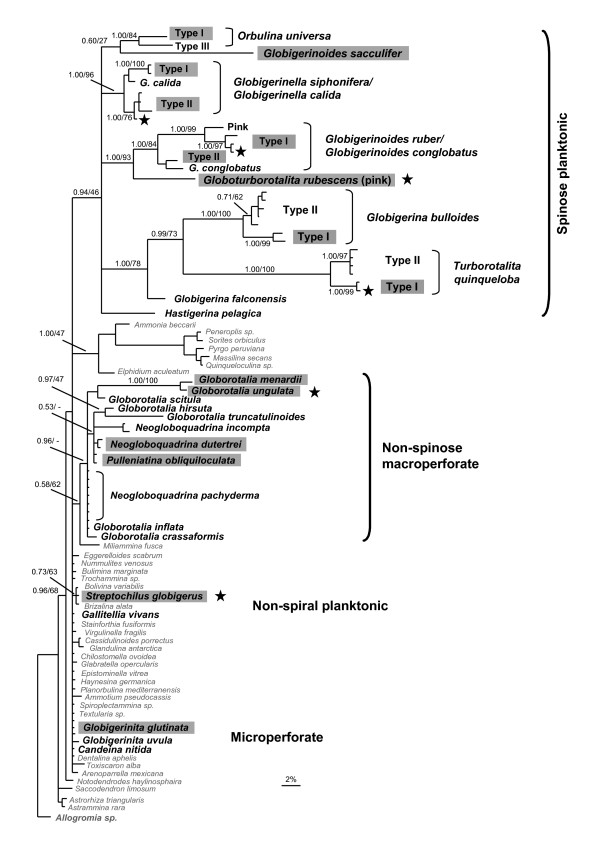
**Bayesian inference SSU rDNA phylogenetic tree showing the position of the Arabian Sea morphospecies and genotypes within the foraminifera**. The phylogeny is based on 407 unambiguously aligned nucleotide sites and is rooted on the benthic foraminifer *Allogromia sp*. Bayesian posterior probabilities (from the last 1000 trees, obtained within MrBayes) and ML bootstraps (expressed as a percentage, 1000 replicates) are shown on the tree (BI posterior probabilities/ML bootstraps). The scale bar corresponds to a genetic distance of 2%. Benthic foraminiferal taxa are shown in grey text, and planktonic foraminifera are shown in black. Morphospecies and genotypes found in the Arabian Sea are shown on a grey background. A star indicates a novel sequence obtained from the Arabian Sea cruise (CD148). The sequence for *S. globigerus *is also presented in [72].

A comprehensive foraminiferal phylogeny, based on 407 bp of the SSU rRNA gene (Figure 2) highlights the placement of the Arabian Sea taxa. All methods of phylogeny reconstruction utilised were largely consistent in their inferred trees, and the phylogeny is in general agreement with previous studies [4,5,7,10,22,35]. The planktonic foraminifera appear polyphyletic, falling in at least 4 separate areas of the tree (Figure 2), consistent with the morphological groupings of the spinose (Globigerinidae and Hastigerinidae), non-spinose macroperforate (Globorotaliidae & Pulleniatinidae), non-spinose microperforate (Candeinidae), and the non-spiral planktonic foraminifera (see [71]).

**Figure 3:**
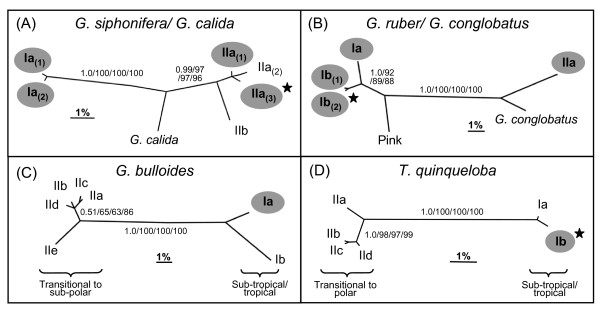
**SSU rDNA phylogenetic trees of (A) *Globigerinella siphonifera/Globigerinella calida *(668 unambiguously aligned nucleotide sites), (B) *Globigerinoides ruber/Globigerinoides conglobatus *(589 bp), (C) *Globigerina bulloides *(669 bp), (D) *Turborotalita quinqueloba *(748 bp)**. The phylogenies were constructed using Bayesian Inference and are unrooted. Bayesian posterior probabilities and ML, NJ, and MP bootstraps (expressed as a percentage) are shown on the trees (BI/ML/NJ/MP). The scale bar corresponds to a genetic distance of 1%. Morphospecies and genotypes found in the Arabian Sea are shown on a grey background. A star indicates a novel sequence obtained from the Arabian Sea cruise (CD148).

The spinose planktonic foraminifera were represented by seven morphospecies within the Arabian Sea mixed layer (*Orbulina universa*, *Globigerinoides sacculifer*, *Globigerinella siphonifera*, *Globigerinoides ruber *(white), *Globoturborotalita rubescens *(pink), *Globigerina bulloides*, and *Turborotalita quinqueloba)*. Only a single *O. universa *Type I individual was genotyped, falling together with *G. sacculifer *(Figure 2). Four genetic types of *G. siphonifera *were identified (Types Ia_(1)_, Ia_(2)_, IIa_(1)_, and the novel IIa_(3)_) (668 bp SSU rDNA phylogeny; Figure 3A). The subtle *G. siphonifera *Type IIa sub-types, shown previously as the IIa complex by Darling and Wade [9], are named here as subtypes IIa_(1) _(Genbank:U80788), IIa_(2) _(Genbank:AF102227, Genbank:AJ3905674, Genbank:Z83960), and IIa_(3) _(this study). *Globoturborotalita rubescens *(pink) is included in a foraminiferal phylogeny for the first time and falls in a well-supported clade as the sister taxon to *G. ruber/G. conglobatus *(Bayesian posterior probability (pp) = 1.00, 93% ML bootstrap support) (Figure 2). *Globigerinoides ruber *(white) was represented by four genetic types (Types Ia, Ib_(1)_, the novel Ib_(2)_, and IIa) (589 bp SSU rDNA phylogeny; Figure 3B). A subtly different variant of *G. ruber *Type Ib was discovered in the Arabian Sea, splitting Ib into subtypes Ib_(1) _and the new Ib_(2)_. *Globigerina bulloides *was represented by Type Ia, which falls as a sister to Type Ib (669 bp SSU rDNA phylogeny; Figure 3C). A new variant of *T. quinqueloba *Type I was discovered, though only a single individual was successfully sequenced. It is named here as Type Ib and falls together with Type Ia (748 bp SSU rDNA phylogeny; Figure 3D).

Four non-spinose macroperforate morphospecies were present in the Arabian Sea (*Globorotalia menardii*, *Globorotalia ungulata, Neogloboquadrina dutertrei*, and *Pulleniatina obliquiloculata*) (Figure 2). *Globorotalia menardii *and the newly sequenced *G. ungulata *fell together (pp = 1.00 BI, 100% ML, Figure 2) with the other macroperforates, though the placement of *G. menardii *was inconsistent across tree reconstruction methods, possibly a result of its unusually high rate of evolution. Very minor sequence variation was detected in *G. menardii*, though insufficient to warrant sub-type status. The three specimens of *G. ungulata *exhibited the discriminating morphological features of this morphospecies (e.g. a keel structure on the umbilical shoulder of the test [71]), though some workers believe *G. ungulata *to be an immature form of *G. tumida*. For *N. dutertrei*, minor sequence variation was detected in the most variable regions of the SSU gene, as noted in other neogloboquadrinid morphospecies [9], however, extensive cloning would be required to determine whether individual genetic types are present. All *Pulleniatina obliquiloculata *sequences obtained were identical to each other, however differed subtly from those currently in GenBank. Further investigation will be necessary to determine if they represent a genetic sub-type of the species.

Of the three microperforate planktonic morphospecies sequenced to date; *Globigerinita uvula *[35], *Globigerinita glutinata *[22] and *Candeina nitida *[36], only *G. glutinata *was found in the Arabian Sea mixed layer. Examining all *G. glutinata *sequences available to date, three subtly different genetic types can be identified, named here as Type 1a_(1) _(Genbank:AF250105; and Arabian Sea) 1a_(2) _(Genbank:Z83974), and 1a_(3) _(Genbank:AY453136). Recent cloning of *G. glutinata *from North-West Pacific assemblages [36] indicates that these are most likely to be genuine subtype differences.

The non-spiral morphospecies, *Streptochilus globigerus*, one of two extant biserial planktonic foraminifera, was abundant in the Arabian Sea mixed layer and falls together with infaunal benthic biserial species *Bolivina variabilis *in the phylogeny (Figure 2; [72]). The morphospecies exhibits minor intra-specific variation in the SSU sequences, as in other benthic foraminiferal species [9].

### Biogeographical distributions of the Arabian Sea foraminiferal genetic types

**Figure 4:**
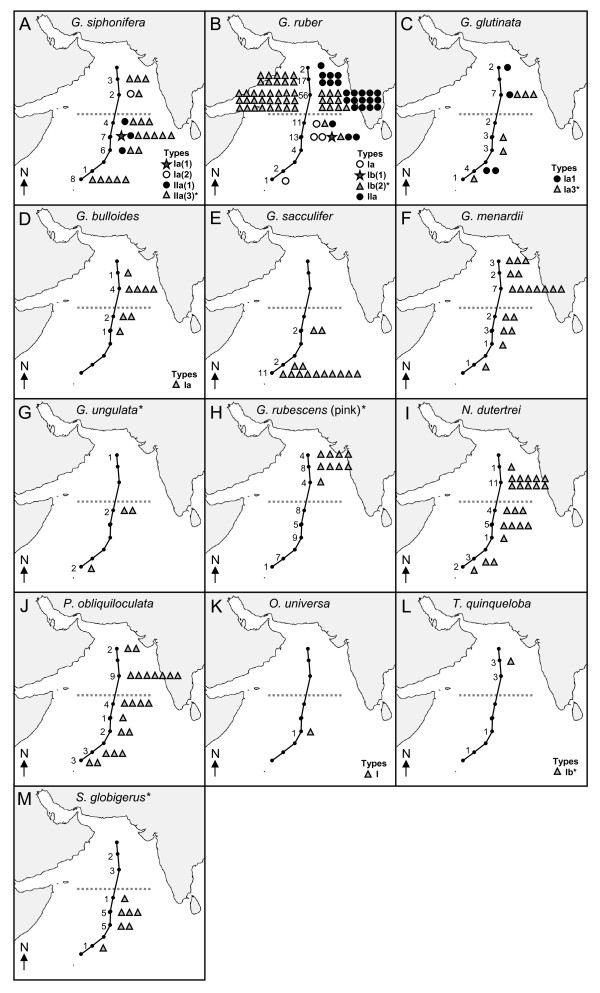
**Arabian Sea maps showing the spatial distribution of genetic types of the morphospecies identified along the cruise transect**. Their numbers do not necessarily reflect the absolute frequency of morphospecies or genotypes in the water column. The dotted line denotes approx. water mass boundary (higher productivity, high salinity in the north/oligotrophic, low salinity in the south) as determined from Figure 1D & 1E. The number of specimens collected at each station for genetic analysis is indicated by a number. A key to genotypes is provided on each map. * indicates novel sequences obtained from the Arabian Sea cruise (CD148).

Analysis of the morphospecies genetic type distribution data (Figure 4) combined with a visual assessment of the bulk assemblage data showed some distinct ecological segregation related to the physical oceanography of the Arabian Sea.

#### Globigerinella siphonifera

*Globigerinella siphonifera *was distributed throughout the transect and was represented by four genetic types (Figure 4A). The newly recognised Type IIa_(3) _(n = 19) was distributed throughout the cruise transect, thriving equally in both the northern and southern water masses. The other genetic types appeared more rare. Type Ia_(2) _(n = 1) was found only in the northern water mass and Types Ia_(1) _(n = 1) and IIa_(1) _(n = 3) were found in low numbers in the southern water mass.

#### Globigerinoides ruber

Assessment of the bulk assemblage samples revealed that *G. ruber *was the dominant morphospecies in the Arabian Sea during the SW monsoon. It was found in high numbers in the more eutrophic, high salinity water of the north and occurred in significantly lower numbers in the more oligotrophic lower salinity water mass to the south. There are four genetic types of *G. ruber *in the Arabian Sea assemblage (Figure 4B), which have distinctive biogeographies. Only Type IIa (n = 24) and Type Ib_(2) _(n = 46) were found in the more eutrophic, higher salinity water mass of the northern Arabian Sea. The other *G. ruber *genetic types, Ia (n = 4) and Ib_(1) _(n = 1), were not found in the northern water mass following extensive genotyping of the water column. These genetic types were found in low numbers within the southern water mass, with only a single specimen of *G. ruber *Type Ia identified at station 9.

#### Globigerinita glutinata

*Globigerinita glutinata *was found throughout the transect. Two potentially distinct subtypes of Type Ia were identified in the central Arabian Sea mixed layer; Types 1a_(1) _(n = 4) and 1a_(3) _(n = 6), each distributed along the length of the cruise transect (Figure 4C).

#### Globigerina bulloides

*Globigerina bulloides *was present in very low numbers in the bulk samples and was distributed mainly in the more eutrophic, high salinity water mass of the northern region. Only genetic type Ia (n = 8) was found, confined to the northern water mass (Figure 4D).

#### Globigerinoides sacculifer

*Globigerinoides sacculifer *was found only in the southern waters, south of station 4. Only a single genetic type was found (n = 14), which was identical to all other *G. sacculifer *sequenced to date (Figure 4E).

#### Globorotalia menardii

The bulk assemblage data clearly showed that *G. menardii *was present across the whole transect with numbers increasing significantly towards the South, though this pattern was not reflected in the number of specimens collected for genotyping (Figure 4F). Despite the distribution difference between the water masses, only a single genetic type (n = 18) was found in the mixed layer along the cruise transect.

#### Globorotalia ungulata

Assessment of the bulk assemblage showed that *Globorotalia ungulata *was more common in the southern part of the cruise transect. Only three specimens were sequenced and a single genetic type found (n = 3; Figure 4G).

#### Globoturborotalita rubescens (pink)

*Globoturborotalita rubescens *(pink) was present throughout the transect, though only nine specimens were successfully amplified (Figure 4H). This newly sequenced morphospecies showed no sequence variation in the specimens collected between stations 1-3.

#### Neogloboquadrina dutertrei

*Neogloboquadrina dutertrei *was distributed along the length of the cruise transect, and is most likely represented by a single genetic type in the Arabian Sea (n = 22; Figure 4I). However, as in most *Neogloboquadrina*, *N. dutertrei *specimens exhibit intra-individual variation in their SSU gene repeats and the presence of more than one genetic type cannot be ruled out without extensive cloning.

#### Pulleniatina obliquiloculata

*Pulleniatina obliquiloculata *was distributed along the length of the cruise transect. Only a single genetic type was found (n = 21; Figure 4J).

#### Orbulina universa

*Orbulina universa *was very rare in the water column. Only a single specimen of Type I was identified in the southern water mass at station 6 (Figure 4K).

#### Turborotalita quinqueloba

It is difficult to differentiate *T. quinqueloba *from tiny juveniles of other morphospecies, but mature specimens were rare. Only a single specimen of Type Ia was amplified at station 2 (Figure 4L).

#### Streptochilus globigerus

Assessment of the bulk assemblage showed that the biserial morphospecies, *S. globigerus*, occurred in substantial numbers along the length of the cruise transect. Only a single genetic type was identified (n = 7; Figure 4M).

## Discussion

Sampling of the tropical Arabian Sea during the SW monsoon uncovered a wealth of planktonic foraminiferal diversity. The 13 morphospecies found displayed high levels of SSU rRNA genetic diversity, with a total of 20 independent genetic types being recorded between them.

Three morphospecies: *Globoturborotalita rubescens *(pink), *Globorotalia ungulata *and *Streptochilus globigerus *were sequenced for the first time from Arabian Sea cruise CD148. *Globoturborotalita rubescens *(pink) falls at the base of a well-supported cluster with *G. ruber *and *G. conglobatus *(Figure 2). Fossil record studies show that it first appeared in the Middle Pliocene, around 3.6 million years ago [73] and may have evolved from *Globigerina woodi *[74] via the morphospecies *Globigerina decoraperta *[73]. *Globorotalia ungulata *falls together with the morphologically similar species, *G. menardii*, at the end of a relatively long branch in the main phylogeny (Figure 2). It appeared in the Late Pliocene around 2.5 million years ago and is thought to have evolved from *Globorotalia tumida *[73], however other extant globorotaliid morphospecies will need to be sequenced before their exact ancestry can be determined. *Streptochilus globigerus *fell among the benthic foraminifera in the main phylogeny (Figure 2), exhibiting extremely high sequence identity to the benthic species *Bolivina variabilis*, sufficient to suggest that *S. globigerus *and *B. variabilis *are one and the same morphospecies (discussed in more detail in [72]). In addition, four new foraminiferal genetic types (*G. ruber *Type Ib_(2)_, *G. siphonifera *Type IIa_(3)_, *T. quinqueloba *Type Ib and *G. glutinata *Type 1a_(1)_) were identified from this Arabian Sea cruise.

### Evidence for ecological partitioning among the Arabian Sea morphospecies/genetic types

The varied hydrographic conditions and extreme seasonal variation of the Arabian Sea provide a unique environment within which to study the ecological adaptations of planktonic foraminiferal morphospecies and their individual genetic types. The cruise transect was conducted during the SW monsoon, when environmental conditions were most pronounced and a strong disparity existed between a high salinity, more eutrophic water mass in the north (Figure 1A-E; stations 1-3), and a low salinity, oligotrophic water mass in the south (Figure 1A-E; stations 4-9). Analysis of the spatial distributions of planktonic foraminiferal morphospecies and genetic types within the Arabian Sea mixed layer at this time revealed a number of non-random patterns of geographical distribution, suggestive of distinct ecological adaptations.

The spinose morphospecies, *Globigerinella siphonifera *(Figure 4A) and *Globigerinoides ruber *(Figure 4B), appear to offer particularly excellent examples of divergent biogeographies in their genetic types, each being represented by four individual genetic types, exhibiting apparently ecologically distinct distribution patterns.

*Globigerinella siphonifera *is represented by two highly divergent SSU rRNA genetic lineages, Type I and Type II, which from a wealth of biological evidence may be considered as two distinct species [4,15,75]. The newly recognised Type IIa_(3) _was the dominant genetic type of *G. siphonifera *in the Arabian Sea, and was distributed throughout both water masses in large numbers (n = 19) (Figure 4A), suggesting a broad tolerance for the varying hydrographic conditions. This new genetic type has yet to be found elsewhere, but may eventually be discovered in other parts of the Indo-Pacific, a region that has not been sampled extensively. Type IIa_(1)_, conversely, was found only in small numbers in the southern water mass (n = 3), suggesting that despite the low level of genetic distinction (Figure 3A), Type IIa_(1) _may have more specialised ecological requirements than Type IIa_(3)_. The closely related genetic types Ia_(1) _and Ia_(2), _represented by only single individuals, also displayed divergent ecologies, the former appearing in the oligotrophic southern water mass and the latter in the more eutrophic north. It is interesting to note that the main ecological divide between genetic types does not reflect their phylogenetic separation into the Type I and Type II lineages. Ecological partitioning instead appears to play a greater role in the divergence of closely related genetic types.

The SSU rRNA phylogeny of *G. ruber *is again characterised by a deep divergence between two extant lineages (lineage 1: *G. ruber *(white) types Ia, Ib_(1)_, Ib_(2)_, and *G. ruber *(pink), lineage 2: *G. ruber *(white) Type IIa and *G. conglobatus*) (Figure 3B), indicative of a species level distinction [5,9,31]. The biogeographical distribution of *G. ruber *genetic types in the Arabian Sea was unmistakably correlated to the hydrographic provinces during the SW monsoon (Figure 4B). *Globigerinoides ruber *dominated the more eutrophic/higher salinity water mass of the northern Arabian Sea, though genotyping revealed the presence of only two of the *G. ruber *genetic types here (Ib_(2) _and IIa), both occurring in equally high numbers. The other two genetic types (Ia and Ib_(1)_) were absent from the more eutrophic/higher salinity waters of the northern Arabian Sea, being found exclusively in the oligotrophic/low salinity southern water mass. It can reasonably be deduced that primary productivity is the main factor determining the distribution of the genetic types across the region, giving *G. ruber *great potential as a paleoproxy for ocean productivity.

To fulfil this role, a link must be demonstrated between genetic type and subtle variations in shell morphology, as has already been achieved for fellow spinose morphospecies, *O. universa *[11]. Several morphological variants or 'morphotypes' have already been recorded within *G. ruber *(white) [76-79] and crucially, *G. ruber *(white) genetic types I and II can be distinguished morphologically [80], and are consistent with the *G. ruber *senso stricto (s.s.) and *G. ruber *senso lato (s.l) morphotypes of Wang [81]. Differences in ecological behaviour have been noted between these two genetic types/morphotypes, with stable isotope and Mg/Ca data together with field observations revealing differing depth habitats and nutrient requirements between the two [78,81-83]. The combined findings suggest an adaptation of *G. ruber *Type I to oligotrophic, shallow conditions, and an adaptation of Type II to eutrophic, deeper conditions.

In the Arabian Sea we indeed see a clear ecological distinction between the *G. ruber *Type Ia and Type II lineages; Type 1a occupying only the oligotrophic southern water mass, and Type IIa almost exclusively occupying the more eutrophic north. Type 1a has also been found to be restricted to oligotrophic waters in both the North Atlantic subtropical gyre and the eastern Mediterranean Sea [31]. It likely dominates during the more oligotrophic periods of the seasonal cycle in the Arabian Sea. Of the other Type I genetic types, Ib_(1) _also fits the 'oligotrophic Type I profile', being present in the low-nutrient south of the Arabian Sea, though it was represented by only a single specimen. Type Ib_(2)_, however, is far from being adapted to oligotrophic conditions, occupying the eutrophic northern water mass of the Arabian Sea together with Type IIa. It seems then that as in *G. siphonifera*, ecological partitioning of the *G. ruber *genetic types may not always reflect the Type I and Type II lineage differentiation. *Globigerinoides ruber *Types Ib_(1) _and Ib_(2) _appear ecologically distinct in their distribution patterns, despite being only subtly different at the genetic level.

The three planktonic foraminiferal morphospecies, *Globigerina bulloides, Globigerinoides sacculifer*, and *Orbulina universa*, were each represented by only single genetic types in the Arabian Sea, which again displayed non-random biogeographical distributions between the northern and southern water masses. The disparate distributions of *Globigerina bulloides *and *G. sacculifer *(Figures 4D, 3 and 4E) give strong indications of specific ecological requirements, which are most likely related to nutrient availability.

*Globigerina bulloides *is more typical of sub-polar regions [84], but also characterises upwelling zones in lower latitudes [85]. It is comprised of two major lineages (Figure 3C), Type I occurring in warm waters, and Type II occurring in cold waters [9]. In the Arabian Sea only Type Ia was present, occurring predominately towards the north of the region (Figure 3, 4D). It's absence from the most oligotrophic, lower salinity waters (stations 6 - 9) (bulk sample assessment and Figure 3, 4D) perhaps indicates an adaptation to slightly more eutrophic, higher salinity conditions. Interestingly, *Globigerina bulloides *dominates the planktonic foraminiferal assemblages in the cooler upwelling coastal waters of the Arabian Sea [52]. It remains to be seen whether the warm water genetic type of the central Arabian Sea mixed layer (Type 1a) is ecologically distinct from those found in high numbers in the upwelling coastal regions of the Arabian Sea.

*Globigerinoides sacculifer*, by contrast, was the dominant morphospecies in the southern Arabian Sea during the SW monsoon. Only a single genetic type of *G. sacculifer *has been recorded globally. In this study it was confined to the southern oligotrophic water mass (Figure 4E), reflecting a possible adaptation to more oligotrophic waters [86,87]. It has been postulated that other factors such as the chlorophyll maximum or thermocline development may affect its distribution [88], and its status in the Arabian Sea water column has been shown to vary with temperature, salinity, nutrients and thermocline depth [52]. Salinity is an unlikely limiting factor as *G. sacculifer *is a euryhaline species, capable of tolerating salinities in a range of 24‰ - 47‰ [71].

One further morphospecies, *O. universa*, was restricted in its distribution. It was represented by only a single Type 1a specimen, found in the oligotrophic southern water mass. Though insufficient data prevents us from drawing conclusions regarding its ecological adaptations, this is consistent with the previous classification of *O. universa *Type Ia as an oligotrophic-adapted type [11].

Some morphospecies from the Arabian Sea displayed broad distributions during the SW monsoon, indicating that they are not restricted by adaptations to sea surface productivity, the main discriminating ecological factor between the northern & southern water masses. The prominent morphospecies, *G. menardii*, *G. rubescens *(pink), *N. dutertrei *and *P. obliquiloculata *(Figure 4F, H, I and 4J) were each represented by only single genetic types, exhibiting wide distributions along the whole transect. Bulk samples did indicate that *G. menardii *numbers tended to increase in the assemblage towards the most southern part of the cruise transect. It should also be noted that different genetic types have potentially been recognised within *N. dutertrei *[39] and *P. obliquiloculata *(unpublished observation), though extensive sampling and cloning will be required before their individual biogeographical distributions can be determined.

Other broadly distributed morphospecies included *G. glutinata *(represented by 2 genetic types), *G. ungulata, T. quinqueloba *(only one Type 1b specimen genotyped), and *S. globigerus *(Figures 4C, G, L, and 4M). The tiny spinose morphospecies *T. quinqueloba *is likely to be underrepresented in the data set; it's small size leading to difficulties in collection. *Streptochilus globigerus *was found throughout the cruise transect and may be of particular interest. This sporadically occurring, biserial planktonic foraminifer [89,90] displayed such high levels of SSU rDNA sequence identity to the benthic species *Bolivina variabilis *from the Kenyan coastal region [91] (located south west of our central Arabian Sea sampling stations) that they must represent the same species [72]. In the benthos, *B. variabilis/S. globigerus *lives as a shallow to intermediate infaunal dweller in the continental shelf sediments. During the SW monsoon (Figure 1B), its populations become expatriated by the winds and currents far offshore, where they continue to live and grow as plankton in the open ocean [72]. *Streptochilus globigerus *is therefore tychopelagic [92] in nature, exploiting both benthic and planktonic habitats [72].

Ecological processes are increasingly being viewed as a vital mode of diversification in the marine environment, with evidence of ecological partitioning being reported for many marine taxa [4,7-9,20,27-30]. From our study of the tropical Arabian Sea, we have demonstrated that biogeographical distributions of the planktonic foraminiferal morphospecies/genetic types can be influenced by adaptations to differing hydrographic conditions. Salinity is unlikely to be a limiting factor in the biogeographic distributions of planktonic foraminifera, as it has previously been shown that planktonic foraminifera are tolerant of extremes of salinity [93]. We therefore propose that primary productivity is the principal variable determining the disparate distributions observed. Evidence of distinct ecological requirements between closely related genetic types implies that ecological partitioning may indeed play a role in the diversification of some planktonic foraminifera. Vicariant processes, however, have also been shown to play an important role in the diversification of several planktonic foraminiferal morphospecies, particularly in the higher latitudes [6-9,38,39]. In reality, the mechanisms of diversification and speciation within the marine environment are undoubtedly quite complex. It will only be with further research that the relative roles of ecological and vicariant processes can be fully elucidated.

### Do algal symbionts play a role in divergent adaptations to sea-surface nutrients?

In the Arabian Sea, primary productivity appeared to represent the primary cause of divergent ecological adaptation amongst foraminiferal genetic types, however, we have yet to explore the biological mechanisms involved. Storz *et al. *[94] proposed that planktonic foraminiferal species respond primarily to productivity, triggered by the seasonal dynamics of vertical stratification of the upper water column and speculated that the distinct nutrition strategies of strictly asymbiontic, facultatively symbiontic, and symbiontic species may play a key role in explaining their abundances and temporal succession.

In fact differences in symbiont affiliations may indeed help to explain the divergent adaptations to sea-surface nutrients observed in the Arabian Sea morphospecies. *Globigerina bulloides*, for example, was distributed mainly towards the more eutrophic north of the Arabian Sea and is known to be symbiont barren [71], reliant on high levels of primary productivity and food availability in the water column. *Globigerinoides sacculifer *conversely was restricted to the oligotrophic waters of the southern water mass, and is known to be obligatory symbiont bearing [71], benefiting from photosynthetic energy contributions. Finally, of the broadly distributed morphospecies within the Arabian Sea, *G. menardii*, *N. dutertrei*, *P. obliquiloculata*, and *G. glutinata *are all known to harbour facultative symbionts [71], meaning that they can either lack or possess symbionts. This may result in their observed versatility, allowing them to either exploit high nutrient conditions (e.g. those in the northern Arabian Sea), or to survive under highly oligotrophic conditions (e.g. those in the southern Arabian Sea), by means of photosynthesis.

It may be that variations in symbiont association could also be involved in the ecological partitioning of individual genetic types within morphospecies, driving their diversification, though little data is available at present. Differences in symbiotic associations have certainly been cited as a possible explanation for the different depth habits and nutrient requirements of the Type I and Type II lineages in both *G. siphonifera *[15,75] and *G. ruber *(corresponding to the *G. ruber *s.s. or type b "platys" morphotype, and the *G. ruber *s.l. or type a "normal" morphotype respectively) [79,82]. However, such studies did not account for possible ecological partitioning between genetic sub-types within the major Type I and Type II lineages in either *G. siphonifera *or *G. ruber*. The results gained here certainly suggest that not all genetic types fall within the 'Type I' and 'Type II' profiles as far as adaptation to nutrients is concerned. The relationship between ecological partitioning in the planktonic foraminifera genetic types and variations in their symbiotic associations certainly warrants further investigation.

## Conclusions

During the SW monsoon, pronounced environmental conditions lead to a strong disparity between the northern and southern water masses of the Arabian Sea. We find a distinct difference in the distribution and ecology of the planktonic foraminifera of the Arabian Sea mixed layer at this time, segregating morphospecies and genetic types between the high salinity, more eutrophic north and the lower salinity, oligotrophic south. In the north, *Globigerinoides ruber *dominated, followed by *Neogloboquadrina dutertrei, Pulleniatina obliquiloculata, Globorotalia menardii*, and *Globigerinita glutinata*. In the south *Globigerinoides sacculifer *dominated, followed by *Globigerinoides ruber *and *Globorotalia menardii*. For those morphospecies represented by complexes of several discrete genetic types within the Arabian Sea mixed layer, individual genetic types were found to have distinct ecologies and novel adaptations to differing physical oceanographic conditions. *Globigerinoides ruber *showed a clear ecological distinction between its Type Ia/Ib_(1) _and Type II lineages, supporting past opinions that Types I and II represent independent species [5,9,31]. However, Type Ib_(2)_, did not fit the typical *G. ruber *Type I 'oligotrophic' profile [79,80,82], indicating a divergent ecological adaptation from close relative, Type Ib_(1)_. Within both *Globigerinoides ruber *and *Globigerinella siphonifera*, subtle sub-types were found to display differing geographical distributions, implicating sea-surface productivity as a significant ecological source of divergent selection in closely related planktonic foraminiferal genetic types. Differing symbiotic associations are a possible mechanism by which divergent nutrient-related adaptations may have arisen in the planktonic foraminiferal morphospecies and possibly even their genetic types.

We have found compelling evidence for ecological partitioning within the planktonic foraminifera of the Arabian Sea. Future efforts should now concentrate on gathering similar data from other global locations, to build a more extensive picture of the ecological requirements of the different foraminiferal genetic types. The ability of foraminiferal genetic types to become specialised and adapted to life in regionally distinct ecosystems is a likely driver of their divergence and speciation in the open ocean, running counter to the apparent lack of barriers to gene flow. If the ecologically divergent foraminiferal genetic types could also be identified from the morphology of their shells, it could represent a considerable improvement to quantitative faunal and geochemical palaeoenvironmental reconstructions.

## Supplementary Material

Additional file 1 **Table S1**. A list of all planktonic and benthic foraminiferal morphospecies/genetic types included in the phylogenetic analyses, with their GenBank accession numbers. Click here for file

Additional file 2 **Figure S1**. SSU rDNA sequence alignment for the foraminifera showing the 407 unambiguously aligned nucleotide sites used to reconstruct the main phylogeny in Figure 2. Click here for file

Additional file 3 **Figure S2**. SSU rDNA sequence alignments for four of the most common Arabian Sea planktonic foraminiferal morphospecies, showing the unambiguously aligned nucleotide sites used to reconstruct the phylogenies in Figure 3: *Globigerinella siphonifera *and *Globigerinella calida *(668 bp), *Globigerinoides ruber *and *Globigerinoides conglobatus *(589 bp), *Globigerina bulloides *(669 bp), and *Turborotalita quinqueloba *(748 bp). Click here for file
