# Supplementary material for: Ecological partitioning and diversity in tropical planktonic foraminifera
Source: BMC Evol Biol. 2012 Apr 16;12:54. doi: 10.1186/1471-2148-12-54 (PMC3361484; doi:10.1186/1471-2148-12-54)
Supplement: Additional file 1 — Table S1. A list of all planktonic and benthic foraminiferal morphospecies/genetic types included in the phylogenetic analyses, with their GenBank accession numbers. [file 1471-2148-12-54-S1.PDF]

Table S1 – Foraminifera taxa list

Abbreviated name	Full name	Genetic Type	GenBank Accession
G. siphonifera	*Globigerinella siphonifera*	Ia(1)	U65631
		Ia(2)	JQ743484
		IIa(1)	U80788
		IIa(2)	AF102227
		IIa(3)	**JQ799892**
		IIb	AF102228
G. calida	*Globigerinella calida*		JQ743485
O. universa	*Orbulina universa*	I	U80791
		III	AF102229
G. sacculifer	*Globigerinoides sacculifer*		U65633
G. ruber	*Globigerinoides ruber*	Pink	U65634
		Ia	U80789
		Ib(1)	Z69599
		Ib(2)	**JQ799893**
		IIa	AF102230
G. conglobatus	*Globigerinoides conglobatus*		U80790
G. rubescens (pink)	*Globoturborotalita rubescens*	Pink	**JQ799894**
G. bulloides	*Globigerina bulloides*	Ia	U80793
		Ib	Z83957
		IIa	AF250107
		IIb	AF250109
		IIc	AF250111
		IId	AF250106
		IIe	HM641800
T. quinqueloba	*Turborotalita quinqueloba*	Ia	AF25250116
		Ib	**JQ799895**
		IIa	AF250112
		IIb	AF250114
		IIc	AF250115
		IId	AY241710
G. falconensis	*Globigerina falconensis*		AF387172
H. pelagica	*Hastigerina pelagica*		Z83958
G. menardii	*Globorotalia menardii*		**JQ799896**
G. ungulata	*Globorotalia ungulata*		**JQ799897**
G. hirsuta	*Globorotalia hirsuta*		Z83973
G. scitula	*Globorotalia scitula*		**JQ799898**
G. truncatulinoides	*Globorotalia truncatulinoides*		Z83968
N. pachyderma	*Neogloboquadrina pachyderma*	I	AY3053
		II	AY305330
		III	AF250119
		IV	AF250120
		V	AY305332
		VI	EF447102
		VII	EF447103
N. dutertrei	*Neogloboquadrina dutertrei*	C	U65635
		1b	AY241707
P. obliquiloculata	*Pulleniatina obliquiloculata*	BR	AY241709
		AS	**JQ799899**
G. inflata	*Globorotalia inflata*		Z83971
G. crassaformis	*Globorotalia crassaformis*		AY453134
N. incompta	*Neogloboquadrina incompta*	I	AF250117
		II	AY241711
G. glutinata	*Globigerinita glutinata*	Ia(1)	AF250105
		Ia(2)	Z83974
		Ia(3)	**JQ799900**
C. nitida	*Candeina nitida*		AB468837
G. uvula	*Globigerinita uvula*		AF387173
B. variabilis	*Bolivina variabilis*		AY359140
S. globigerus	*Streptochilus globigerus*		GQ265800
B. alata	*Brizalina alata*		AF533837
G. vivans	*Gallitellia vivans*		AB364520
C. porrectus	*Cassidulinoides porrectus*		AY934737

C. ovoidea	*Chilostomella ovoidea*	AY465842
G. opercularis	*Glabratella opercularis*	Z69614
E. aculeatum	*Elphidium aculeatum*	Z69618
E. vitrea	*Epistominella vitrea*	AM491316
H. germanica	*Haynesina germanica*	AF190721
P. mediterranensis	*Planorbulina mediterranensis*	DQ452709
S. fusiformis	*Stainforthia fusiformis*	AY934745
V. fragilis	*Virgulinella fragilis*	AY359192
A. pseudocassis	*Ammotium pseudocassis*	AJ312434
Spiroplectammina sp.	*Spiroplectammina sp.*	AJ504689
Textularia sp.	*Textularia sp.*	Z69612
S. limosum	*Saccodendron limosum*	AJ319988
G. antarctica	*Glandulina antarctica*	AY179177
D. aphelis	*Dentalina aphelis*	AJ972511
P. peruviana	*Pyrgo peruviana*	AY179176
M. secans	*Massilina secans*	Z69606
Quinqueloculina sp.	*Quinqueloculina sp.*	Z69605
N. haylinosphaira	*Notodendrodes haylinosphaira*	AJ311214
M. fusca	*Miliammina fusca*	AY822040
T. alba	*Toxiscaron alba*	AJ307749
A. mexicana	*Arenoparrella mexicana*	AJ307741
A. triangularis	*Astrorhiza triangularis*	AJ318224
A. rara	*Astrammina rara*	AJ318223
E. scabrum	*Eggerelloides scabrum*	AJ318228
N. venosus	*Nummulites venosus*	AJ318226
B. marginata	*Bulimina marginata*	DQ408646
Trochammina sp.	*Trochammina sp.*	X86095
Peneroplis sp.	*Peneroplis sp.*	AJ132368
S. orbiculus	*Sorites orbiculus*	AJ404310
Allogromia sp.	*Allogromia sp.*	X86093

Foraminiferal taxa included in the phylogenetic analyses of this study, including their abbreviated and full names, genetic types, and GenBank accession numbers (those highlighted in grey were submitted for this study). *Sample collected from the North Atlantic Ocean, April 2002, RRV Discovery Cruise D262, sequenced for this study.
