# Supplementary material for: Ecological partitioning and diversity in tropical planktonic foraminifera
Source: BMC Evol Biol. 2012 Apr 16;12:54. doi: 10.1186/1471-2148-12-54 (PMC3361484; doi:10.1186/1471-2148-12-54)
Supplement: Additional file 2 — Figure S1. SSU rDNA sequence alignment for the foraminifera showing the 407 unambiguously aligned nucleotide sites used to reconstruct the main phylogeny in Figure 2. [file 1471-2148-12-54-S2.PDF]

Figure S1 - SSU rDNA sequence alignment for the foraminifera

The 407 unambiguously aligned nucleotide sites used to reconstruct the phylogeny in Figure 2 are highlighted in grey. For full species names and GenBank accession numbers please see Table S1 (Additional file 1).

```
                         1          11         21         31         41         51         61         71         81         91        100
                         |          |          |          |          |          |          |          |          |          |         |
G. siphonifera Ia1       GCACCACAAG --A----GCG TGGAGCATGT GGCTTAATTT GACTCAACGC GGGGAATCTT ACCGGGTCCG GACACACTGA GGATTGACAG -----ACAGT
G. siphonifera Ia2       GCACCACAAG --A----GCG TGGAGCATGT GGCTTAATTT GACTCAACGC GGGGAATCTT ACCGGGTCCG GACACACTGA GGATTGACAG -----ACAGT
G. siphonifera IIa1      GCACTACAAG --A----GCG TGGAGCATGT GGCTTAATTT GACTCAACGC GGGGAATCTT ACCGGGTCCG GACACACTGA GGATTGACAG -----ACAAT
G. siphonifera IIa2      GCACTACAAG --A----GCG TGGAGCATGT GGCTTAATTT GACTCAACGC GGGGAATCTT ACCGGGTCCG GACACACTGA GGATTGACAG -----ACAAT
G. siphonifera IIa3      GCACTACAAG --A----GCG TGGAGCATGT GGCTTAATTT GACTCAACGC GGGGAATCTT ACCGGGTCCG GACACACTGA GGATTGACAG -----ACAAT
G. siphonifera IIa       GCACCACAAG --A----GCG TGGAGCATGT GGCTTAATTT GACTCAACGC GGGGAATCTT ACCGGGTCCG GACACACTGA GGATTGACAG -----ACAAT
G. siphonifera IIb       GCACTACAAG --A----GCG TGGAGCATGT GGCTTAATTT GACTCAACGC GGGGAATCTT ACCGGGTCCG GACACACTGA GGATTGACAG -----ACAAT
G. calida                GCACCACAAG --A----GCG TGGAGCATGT GGCTTAATTT GACTCAACGC GGGGAATCTT ACCGGGTCCG GACATACTGA GGATTGACAG -----ACATC
O. universa I            GCACCACAAG ----C--GCG TGGAGCATGT GGCTTAATTT GACTCAACGC GGGAAATCTT ACCAGGTCAG GACACATTGA GGATTGACAG -----ATAGT
O. universa III          GCACCACAAG ----C--GCG TGGAGCATGT GGCTTAATTT GACTCAACGC GGGAAATCTT ACTAGATCAG GACACTTTGA GGATTGACAG -----ACAGT
G. sacculifer            GCACCACAAG ----C--GCG TGGAGCATGT GGCTTAATTT GACTCAACGC GGGAAATCTT ACCAGGTCCA GACATATGGA GGATTGACAG -----ACAGT
G. ruber pink            GCACCACAAG ----C--GCG TGGAGCATGT GGCTTAATTT GACTCAACGC GGGAAATCTT ACCGGGTCCG GACATATCGA GGATTGACAG -----ACAGT
G. ruber Ia              GCACCACAAG ----C--GCG TGGAGCATGT GGCTTAATTT GACTCAACNC GGGAAATCTT ACCGGGTCCG GACATATCGA GGATTGACAG -----ACAGT
G. ruber Ib1             GCACCACAAG ----C--GCG TGGAGCATGT GGCTTAATTT GACTCAACAC GGGAAATCTT ACCGGGTCCG GACGTATCGA GGATTGACAG -----ATAGT
G. ruber Ib2             GCACCACAAG ----C--GCG TGGAGCATGT GGCTTAATTT GACTCAACAC GGGAAATCTT ACCGGGTCCG GACGTATCGA GGATTGACAG -----ATAGT
G. ruber IIa             GCACCACAAG ----C--GCG TGGAGCATGT GGCTTAATTT GACTCAACAC GGGAAATCTT ACCGGGTCCG GACATACCGA GGATTGACAG -----ACTGT
G. conglobatus           GCACCACAAG ----C--GCG TGGAGCATGT GGCTTAATTT GACTCAACCG GGGAAATCTT ACCGGGTCCG GACATACCGA GGATTGACAG -----ACAGT
G. rubescens (pink)      GCACCACAAG ----T--GCG TGGAGCGTGT GGCTTAATTT GACTCAACAC GGGAAATCTT ACCGGGTCCG GACATATCGA GGATTGACAG -----ATTGT
G. bulloides Ia          GCACCACAAG --A----GCG TGGAGTATGT GGCTTAATTT GACTCAACGC GGAAAAGCTT ATCTGGTCCG GACACAGTGA GGATTGACAG -----ACGGT
G. bulloides Ib          GCACCACAAG --A----GCG TGGAGTATGT GGCTTAATTT GACTCAACGC GGAAAAGCCT ATTTGGTCCG GACACAGTGA GGATTGACAG -----ACATG
G. bulloides IIa         GCACCACAAG --A----GCG TGGAGTATGT GGCTTAATTT GACTCAACGC GGAAAAGCTT ATCTGGTCCG GACGCAGTGA GGATTGACAG -----ACAGT
G. bulloides IIb         GCACCACAAG --A----GCG TGGAGTATGT GGCTTAATTT GACTCAACGC GGAAAAGCTT ATCTGGTCCG GACGCAGTGA GGATTGACAG -----ACAGT
G. bulloides IIc         GCACCACAAG --A----GCG TGGAGTATGT GGCTTAATTT GACTCAACGC GGAAAAGCTT ATCTGGTCCG GACACAGTGA GGATTGACAG -----ACAGT
G. bulloides IId         GCACCACAAG --A----GCG TGGAGTATGT GGCTTAATTT GACTCAACGC GGAAAAGCTT ATCTGGTCCG GACACAGTGA GGATTGACAG -----ACAGT
G. bulloides IIe         GCACCACAAG --A----GCG TGGAGTATGT GGCTTAATTT GACTCAACGC GGAAAAGCTT ATCTGGTCCG GACACAGTGA GGATTGACAG -----ACGGT
T. quinqueloba Ia        GCACCACAAG --A----GCG TGGAGTATGT GGCTTAATTC GACTCAACGC GCAATAACTT ACTTGGTCCG AACGCTTTGA GGATTGACAG -----TTTCT
T. quinqueloba Ib        GCACCACAAG --A----GCG TGGAGTATGT GGCTTAATTC GACTCAACGC GCAATAACTT ATTTGGTCCG AACGCTTTGA GGATTGACAG -----TTTCT
T. quinqueloba IIa       GCACCACAAG --A----GCG TGGAGTATGT GGCTTAATTC GACTCAACGC GCAACAATTT ACTTGGTCCG AACGCTTTGG GGATTGACAG -----TTATT
T. quinqueloba IIb       GCACCACAAG --A----GCG TGGAGTATGT GGCTTAATTC GACTCAACGC GCAACAATTT ACTTGGTCCG AACGCTTTGA GGATTGACAG -----TTATT
T. quinqueloba IIc       GCACCACAAG --A----GCG TGGAGTATGT GGCTTAATTC GACTCAACGC GCAACAATTT ACTTGGTCCG AACGCTTTGA GGATTGACAG -----TTATT
T. quinqueloba IId       GCACCACAAG --A----GCG TGGAGTATGT GGCTTAATTC GACTCAACGC GCAACAATTT ACTTGGTCCG AACGCTTTGA GGATTGACAG -----TTATT
G. falconensis           GCACCACAAG --A----GCG TGGAGCATGT GGCTTAATTT GACTCAACGC GGAAAAACTT ACCAGGTCCG GACACACTGA GGATTGACAG -----ACGGT
H. pelagica              GCACCACAAG --A----GCG TGGAGCATGT GGCTTAATTT GACTCAACGC GGGAAATCTT ACCGGGTCCG GACACACTGA GGATTGACAG -----AGTGG
G. menardii              GCACCACAAG --AAC--GCG TGGAGCATGT GGCTTAATTT GACTCAACGC GGGAAATCTT ACCGGGTCCG GATGCACTGA GGATTGACAG -----ACAGG
G. ungulata              GCACCACAAG --AAT--GCG TGGAGCATGT GGCTTAATTT GACTCAACGC GGGAAATCTT ACCGGGTCCG GATACACTGA GGATTGACAG -----ACAAC
G. hirsuta               GCACCACAAG --AAC--GCG TGGAGCATGT GGCTTAATTT GACTCAACGC GGGGAATCTT ACCAGGTCCG GACACACTGA GGATTGACAG -----GCAAA
G. scitula               GCACCACAAG --AAC--GCG TGGAGCATGT GGCTTAATTT GACTCAACGC GGGAAATCTT ACCGGGTCCG GACACACTGA GGATTGACAG -----GCGAT
G. truncatulinoides      GCACCACAAG --AAC--GCG TGGAGCATGT GGCTTAATTT GACTCAACGC GGGGAATCTT ACCGGGTCCG GACACACTGA GGATTGACAG -----ACGGT
N. pachyderma I          GCACCACAAG --AAC--GCG TGGAGCATGT GGCTTAATTT GACTCAACGC GGGAAATCTT ACCGGGTCCG GACACACTGA GGATTGACAG -----GCAAT
N. pachyderma II         GCACCACAAG --AAC--GCG TGGAGCATGT GGCTTAATTT GACTCAACGC GGGAAATCTT ACCGGGTCCG GACACACTGA GGATTGACAG -----GCAAT
N. pachyderma III        GCACCACAAG --AAC--GCG TGGAGCATGT GGCTTAATTT GACTCAACGC GGGAAATCTT ACCGGGTCCG GACACACTGA GGATTGACAG -----GCAAT
N. pachyderma IV         GCACCACAAG --AAC--GCG TGGAGCATGT GGCTTAATTT GACTCAACGC GGGAAATCTT ACCGGGTCCG GACACACTGA GGATTGACAG -----GCAAT
N. pachyderma V          GCACCACAAG --AAC--GCG TGGAGCATGT GGCTTAATTT GACTCAACGC GGGAAATCTT ACCGGGTCCG GACACACTGA GGATTGACAG -----GCAAT
N. pachyderma VI         GCACCACAAG --AAC--GCG TGGAGCATGT GGCTTAATTT GACTCAACGC GGGAAATCTT ACCGGGTCCG GACACACTGA GGATTGACAG -----GTAAT
N. pachyderma VII        GCACCACAAG --AAC--GCG TGGAGCATGT GGCTTAATTT GACTCAACGC GGGAAATCTT ACCGGGTCCG GACACACTGA GGATTGACAG -----GCAAT
N. dutertrei_C           GCACCACAAG --AAC--GCG TGGAGCATGT GGCTTAATTT GACTCAACGC GGGAAATCTT ACCGGGTCCG GACACACTGA GGATTGACAG -----GCAAT
N. dutertrei_Ib          GCACCACAAG --AAC--GCG TGGAGCATGT GGCTTAATTT GACTCAACGC GGGAAATCTT ACCGGGTCCG GACACACTGA GGATTGACAG -----GCAAT
P. obliquiloculata_BR    GCACCACAAG --AAC--GCG TGGAGCATGT GGCTTAATTT GACTCAACGC GGGAAATCTT ACCGGGTCCG GACACACTGA GGATTGACAG -----GCAAT
P. obliquiloculata_AS    GCACCACAAG --AAC--GCG TGGAGCATGT GGCTTAATTT GACTCAACGC GGGAAATCTT ACCGGGTCCG GACACACTGA GGATTGACAG -----GCAGT
G. inflata               GCACCACAAG --AAC--GCG TGGAGCATGT GGCTTAATTT GACTCAACGC GGGAAATCTT ACCGGGTCCG GACACACTGA GGATTGACAG -----GCAAT
G. crassaformis          GCACCACAAG --AAC--GCG TGGAGCATGT GGCTTAATTT GACTCAACGC GGGAAATCTT ACCGGGTCCG GACACACTGA GGATTGACAG -----GCAAT
N. incompta I            GCACCACAAG --AAC--GCG TGGAGCATGT GGCTTAATTT GACTCAACGC GGGAAATCTT ACCAGGTCCG GACACACTGA GGATTGACAG -----GAAGT
N. incompta II           GCACCACAAG --AAC--GCG TGGAGCATGT GGCTTAATTT GACTCAACGC GGGAAATCTT ACCAGGTCCG GACACACTGA GGATTGACAG -----GAAGT
G. glutinata Ia1         GCACCACAAG --AAC--GCG TGGAGCATGT GGCTTAATTT GACTCAACGC GGGAAATCTT ACCGGGTCCG GACACACTGA GGATTGACAG -----GTATT
G. glutinata Ia2         GCACCACAAG --AAC--GCG TGGAGCATGT GGCTTAATTT GACTCAACGC GGGAAATCTT ACCGGGTCCG GACACACTGA GGATTGACAG -----GTATT
G. glutinata Ia3         GCACCACAAG --AAC--GCG TGGAGCATGT GGCTTAATTT GACTCAACGC GGGAAATCTT ACCGGGTCCG GACACACTGA GGATTGACAG -----GTATT
C. nitida                GCACCACAAG --AAC--GCG TGGAGCATGT GGCTTAATTT GACTCAACGC GGGAAATCTT ACCGGGTCCG GACACACTGA GGATTGACAG -----GTATT
G. uvula                 GCACCACAAG --AAC--GCG TGGAGCATGT GGCTTAATTT GACTCAACGC GGGAAATCTT ACCGGGTCCG GACACACTGA GGATTGACAG -----GCAAT
B. variabilis            GCACCACAAG --AAC--GCG TGGAGCATGT GGCTTAATTT GACTCAACGC GGGAAATCTT ACCGGGTCCG GACACACTGA GGATTGACAG -----GTAAC
S. globigerus            GCACCACAAG --AAC--GCG TGGAGCATGT GGCTTAATTT GACTCAACGC GGGAAATCTT ACCGGGTCCG GACACACTGA GGATTGACAG -----GTAAC
B. alata                 ---------- ---------G TGGAGCATGT GGCTTAATTT GACTCAACGC GGGAAATCTT ACCGGGTCCG GACACACTGA GGATTGACAG -----GTAAC
G. vivans                ---------- ---------- ---------- -----AATTT GACTCAACGC GGGAAATCTT ACCGGGTCCG GACACACTGA GGATTGACAG -----GCAAT
C. porrectus             GCACCACAAG --AAC--GCG TGGAGCATGT GGCTTAATTT GACTCAACGC GGGAAATCTT ACCGGGTCCG GACACACTGA GGATTGACAG -----GCAAT
C. ovoidea               GCACCACAAG --AAC--GCG TGGAGCATGT GGCTTAATTT GACTCAACGC GGGAAATCTT ACCGGGTCCG GACACACTGA GGATTGACAG -----GCGAT
G. opercularis           GCACCACAAG --AAC--GCG TGGAGCATGT GGCTTAATTT GACTCAACGC GGGAAATCTT ACCGGGTCCG GACACACTGA GGATTGACAG -----GTTTT
E. aculeatum             GCACCACAAG --AAC--GCG TGGAGCATGT GGCTTAATTT GACTCAACGC GGGAAATCTT ACCGGGTCCG GACACACTGA GGATTGACAG -----ATATA
E. vitrea                GCACCACAAG --AAC--GCG TGGAGCATGT GGCTTAATTT GACTCAACGC GGGAAATCTT ACCGGGTCCG GACACACTGA GGATTGACAG -----GCAAT
H. germanica             GCACCACAAG --AAC--GCG TGGAGCATGT GGCTTAATTT GACTCAACGC GGGAAATCTT ACCGGGTCCG GACACACTGA GGATTGACAG -----ACAAA
P. mediterranensis       GCACCACAAG --AAC--GCG TGGAGCATGT GGCTTAATTT GACTCAACGC GGGAAATCTT ACCGGGTCCG GACACACTGA GGATTGACAG -----GCAAT
S. fusiformis            GCACCACAAG --AAC--GCG TGGAGCATGT GGCTTAATTT GACTCAACGC GGGAAATCTT ACCGGGTCCG GACACACTGA GGATTGACAG -----GCAA-
V. fragilis              GCACCACAAG --AAC--GCG TGGAGCATGT GGCTTAATTT GACTCAACGC GGGAAATCTT ACCGGGTCCG GACACACTGA GGATTGACAG -----GCAAT
A. pseudocassis          GCACCACAAG --AAC--CGG TGGAGCATGT GGCTTAATTT GACTCAACGC GGGAAATCTT ACCGGGTCCG GACACACTGA GGATTGACAG -----GCAAT
Spiroplectammina sp.     GCACCACAAG --AAC--GCG TGGAGCATGT GGCTTAATTT GACTCAACGC GGGAAATCTT ACCGGGTCCG GACACACTGA GGATTGACAG -----G-CAA
Textularia sp.           GCACCACAAG --AAC--GCG TGGAGCATGT GGCTTAATTT GACTCAACGC GGGAAATCTT ACCGGGTCCG GACACACTGA GGATTGACAG -----GTATT
S. limosum               GCACCACAAG --AAC--GCG TGGAGCATGT GGCTTAATTT GACTCAACGC GGGAAATCTT ACCGGGTCCG GACATACTGA GGATTGACAG -----GCTTT
G. antarctica            GCACCACAAG --AAC--GCG TGGAGCATGT GGCTTAATTT GACTCAACGC GGGAAATCTT ACCGGGTCCG GACACACTGA GGATTGACAG -----GCAAT
D. aphelis               GCACCACAAG --AAC--GCG TGGAGCATGT GGCTTAATTT GACTCAACGC GGGAAATCTT ACCGGGTCCG GACACACTGA GGATTGACAG -----GCAAT
P. peruviana             GCACCACAAG --AAC--GCG TGGAGCATGT GGCTTAATTT GACTCAACGC GGGAAATCTT ACCAGGTCCA GACATATTGA GGATTGACAG -----GTGAT
M. secans                GCACCACAAG --AAC--GCG TGGAGCATGT GGCTTAATTT GACTCAACGC GGGAAATCTT ACCAGGTCCG GACATATTGA GGATTGACAG -----GTGAT
Quinqueloculina sp.      GCACCACAAG --AAC--GCG TGGAGCATGT GGCTTAATTT GACTCAACGC GGGAAATCTT ACCAGGTCCA GACATATTGA GGATTGACAG -----GTGAT
N. haylinosphaira        GCACCACAAG --AAC--GCG TGGAGCATGT GGCTTAATTT GACTCAACGC GGGAAATCTT ACCGGGTCCG GACACACTGA GGATTGACAG -----GTGTT
M. fusca                 GCACCACAAG --AAC--GCG TGGAGCATGT GGCTTAATTT GACTCAACGC GGGAAATCTT ACCGGGTCCG GACACACTGA GGATTGACAG -----GCGAT
T. alba                  GCACCACAAG --AAC--GCG TGGAGCATGT GGCTTAATTT GACCCAACGC GGGAAATGTT ACCGGGTCCG GACACACTGA GGATTGACAG -----GCAAT
A. mexicana              GCACCACAAG --AAC--GCG TGGAGCATGT GGCTTAATTT GACTCAACGC GGGAAATCTT ACCGGGACCG GACACACTGA GGATTGACAG -----GCAAT
A. triangularis          GCACCACAAG --AAC--GCG TGGAGCATGT GGCTTAATTT GACTCAACGC GGGAAATCTT ACCGGGTCCG GACATACTGA GGATTGACAG -----GTGCA
```

```
A. rara                  GCACCACAAG --AAC--GCG TGGAGCATGT GGCTTAATTT GACTCAACGC GGGAAATCTT ACCGGGTCCG GACATACTGA GGATTGACAG -----GTGCA
E. scabrum               GCACCACAAG --AAC--GCG TGGAGCATGT GGCTTAATTT GACTCAACGC GGGAAATCTT ACCGGGTCCG GACACACTGA GGATTGACAG -----GCAAA
N. venosus               GCACCACAAG --AAC--GCG TGGAGCATGT GGCTTAATTT GACTCAACGC GGGAAATCTT ACCGGGTCCG GACACACTGA GGATTGACAG -----GTATT
B. marginata             GCACCACAAG --AAC--GCG TGGAGCATGT GGCTTAATTT GACTCAACGC GGGAAATCTT ACCGGGTCCG GACACACTGA GGATTGACAG -----GCAAT
Trochammina sp.          GCACCACAAG --AAC--GCG TGGAGCATGT GGCTTAATTT GACTCAACGC GGGAAATCTT ACCGGGTCCG GACACACTGA GGATTGACAG -----GCAAT
Peneroplis sp.           GCACCACAAG --AAC--GCG TGGAGCATGT GGCTTAATTT GACTCAACGC GGGAAATCTT ACCAGGTCCG GACATATTGA GGATTGACAG -----GCGAT
S. orbiculus             GCACCACAAG --AAC--GCG TGGAGCATGT GGCTTAATTT GACTCAACCC GGGAAATCTT ACCAGGTCCA GACATATTGA GGATTGACAG -----GCGAT
Allogromia sp.           GCACCACAAG --AAC--GCG TGGAGCATGT GGCTTAATTT GACTCAACGC GGGAAATCTT ACCAGGTCCG AACACGCTGA GGATTGACAG -----GTTTT


                        101        111        121        131        141        151        161        171        181        191       200
                         |          |          |          |          |          |          |          |          |          |         |
G. siphonifera Ia1       T--GTCTTT- ----CCCTCC C--------- ---------- ---------- -TTC---GGG GTGGGA---- ---------- ---------- ----------
G. siphonifera Ia2       T--GTCTTT- ----CCCTCC C--------- ---------- ---------- -TTC---GGG GTGGGA---- ---------- ---------- ----------
G. siphonifera IIa1      T--GTCTTT- -TTGTCAAAT TA-------- ---------- ---------- ---------- ---------- ---------- ---------- ----------
G. siphonifera IIa2      T--GTCTTT- -TTGTCAAAT TT-------- ---------- ---------- ---------- ---------- ---------- ---------- ----------
G. siphonifera IIa3      T--GTCTTT- -TGGTCAAAT TT-------- ---------- ---------- ---------- ---------- ---------- ---------- ----------
G. siphonifera IIa       T--GTCTTT- -TTGTCAAAT TT-------- ---------- ---------- ---------- ---------- ---------- ---------- ----------
G. siphonifera IIb       T--GTCCTT- -TTGGC-TTA ---------- ---------- ---------- ---------- ---------- ---------- ---------- ----------
G. calida                ACCACGTCTT TTTGTCCTTA AAA------- ---------- ---------- ---------- ---------- ---------- ---------- ----------
O. universa I            T--------- ---------- ---------- ATACCAAAC- ACGAGCTCGA ACCAGAGTTC ---GTATATG AT-------- ---------- ----------
O. universa III          T--------- ---------- ---------- ATACCGAACT ACGAATAATG AATGGTCCAG GGT------- ---------- ---------- ----------
G. sacculifer            T--------- ---------- ---------- AACCAAAACG CAGC------ ---------- --AGCTAG-T T--------- ---------- ----------
G. ruber pink            T--------- ACCATG---- -CCCTTTCGA A--------- ---------- ---------- ---AGGAGTC AG-------- ---------- ----------
G. ruber Ia              T--------A TACATG---- -CCCT--ACA AGT------- ---------- ---------- ---AGGAGTC AG-------- ---------- ----------
G. ruber Ib1             T--------A TACATG---- -CCCT--ACA AGCAAGT--- ---------- ---------- ---AGGAGTC AG-------- ---------- ----------
G. ruber Ib2             T--------A TACATG---- -CCCT--ACA AGCAAGT--- ---------- ---------- ---AGGAGTC AG-------- ---------- ----------
G. ruber IIa             T--------- -AACTGCAAC ACATGCTGCT AA-------- ---------- ---------- ---------- ---------- ---------- ----------
G. conglobatus           T--------- ---------- ---------- AACTTGCCCT ACCTATAGGA GCTAG----- ---------- ---------- ---------- ----------
G. rubescens (pink)      T--------- -AACGTGCAC CAAAGGTGTT GA-------- ---------- ---------- ---------- ---------- ---------- ----------
G. bulloides Ia          C--------- ---TTATTGG TGGACTCT-A AGACGTC--- ---------- ---------- ---------- ---------- ---------- ----------
G. bulloides Ib          T--------- --CGTATTGG TGGACTTT-A TGA----CAA A--------- ---------- ---------- ---------- ---------- ----------
G. bulloides IIa         T--------- ---TCAGGAG TGGTTCTT-G GTAAA--CAA ---------- ---------- ---------- ---------- ---------- ----------
G. bulloides IIb         T-------AG A---CAGAAG TGGTTTA--G GTAA---CAA ---------- ---------- ---------- ---------- ---------- ----------
G. bulloides IIc         T--------- --GGCAGGAG TGGTTCTT-G GTAAAAACAA ---------- ---------- ---------- ---------- ---------- ----------
G. bulloides IId         T-------AG A---CAGAAG TGAGGTTTTG GTAA---CAA ---------- ---------- ---------- ---------- ---------- ----------
G. bulloides IIe         T--------- ---TTATTAG TGGTTTCT-A AAACACA--- ---------- ---------- ---------- ---------- ---------- ----------
T. quinqueloba Ia        G-------GT ACAATGTGCG GCTTGTTTGT TACAACTACG AATGATTCGA ATTGTTGTAA ATATGACTTG GCCGGCCTTC GGGTGTCTTG GATCGG-CGT
T. quinqueloba Ib        G-------GT ACAATGTGCG GCTTGTTTGT TACAACTGCG AATGACTCGA TTTGTTGTAA ATATGACTTG ACCGGCCTTC GGGTGTCTTG GATGGGTCGT
T. quinqueloba IIa       G-------TA TAGTTCTGAT ATGAGAGGTC TTTGTAGTCA ACGTGTAGGT AGTTGTA--- ---------- ---------- ---------- ----------
T. quinqueloba IIb       G-------TA TAGTTCTGAT ATGGGTGGTA TTTGTAGTCA ACGTGTAGGT AGTTGTA--- ---------- ---------- ---------- ----------
T. quinqueloba IIc       G-------TA TAGTTCTGAT ATGGGTGGTA TTTGTAGTCA ACGTGTAGGT AGTTGTA--- ---------- ---------- ---------- ----------
T. quinqueloba IId       G-------TA TAGTTCTGAT ATGGGTGGTA TTTGTAGTCA ACGTGTAGGT AGTTGTA--- ---------- ---------- ---------- ----------
G. falconensis           T--------- ------GACG CCCTTCGGGG CAAA------ ---------- ---------- ---------- ---------- ---------- ----------
H. pelagica              C-------TT AGGCCACAGA CACAGAATGG GTTTCTTTTA ATACACCCCA TTTGC----- ---------- ---------- ---------- ----------
G. menardii              C-------CA TAGAACAGCT GTATGTATAC AATTGTTATT ATTTAAAT-A ATACAATTAG GA-GCGCCGC TTCTGCGTC- GT-------- ----------
G. ungulata              C-------AT AGACAGAATA CACATACGAT TGTATTCCTA ACTGAATACG ATTAGTGTTG GTCTAAGGAT ---------- ---------- ----------
G. hirsuta               T-------CT AATTAGAATC TTTCTCATTT TTAATGAGTA TAGATTTCTA TTT------- ---------- ---------- ---------- ----------
G. scitula               ATAACGAATC --TTATTT-C T-TAATAAGA TATTCGT--- ---------- ---------- ---------- ---------- ---------- ----------
G. truncatulinoides      T-------AT TGACTTATAC TCGCTTAATG GCTTTAAGCT GTTAAACGTT TAACACCTT- ---------- ---------- ---------- ----------
N. pachyderma I          A-------TC TCA--TGTTT CATTAACC-- ------GTT- ATTAACGTAT CGGTTATTTC C--------- ---------T TAA---CATC GA--------
N. pachyderma II         A-------TC TCA--TGGTT -ATTAACC-G ------GTT- AAT------- -GGTTATTTA TTTAATAGCC ATCGGTTA-T TAAG--CATC GA--------
N. pachyderma III        A-------TC TCA--TGGTT -ATTAACCCG ------GTT- ATTACATTTT GTTTAA---- ---------- -TCGGTTA-T TAAG--CATC GA--------
N. pachyderma IV         A-------TC TCA--TG-TT ----AAC--G ------GTAA GTTTACTTAC CGC------- ---------- ---------T TAA---CATC GA--------
N. pachyderma V          A-------TC TCA--TGGTT -ATTAACC-G ------GTTA AATAGATTTA TTTATTTAG- ---------- -GCGGTTAAT TAAG--CATC GA--------
N. pachyderma VI         A-------TC TCA--TGGTT -ATTAACCCG ------GTT- ATTTGTTTAA ---------- ---------- -TCAGTTA-T TAAG--CATC GA--------
N. pachyderma VII        A-------TC TCA--TGTTT -AATAACC-- ------GTT- ATTAACGTAT CGTGTTATTT AATA------ ---------- ------CATC GA--------
N. dutertrei_C           A-------TC TAAA------ ---------- -----TCGTT TATAATACTT CCTATTATAA TACGC-ATTT A--------- ---------- ----------
N. dutertrei_Ib          A-------TC TAAA------ ---------- -----TCGTT TATAA----T TCTATTATAA TACG-TATTT A--------- ---------- ----------
P. obliquiloculata_BR    A-------TC TATTAAAAGA A--------- -----TTGTT -ATTATAACA TTCTCTTTTT --AATA---- ---------- ---------- ----------
P. obliquiloculata_AS    A-------TC TATTAAAAAG A--------- -----TTATA TTTTTAATAT AATTCTTTTT TTAATA---- ---------- ---------- ----------
G. inflata               A-------TA TTAGCATAAA GATTCGTCTT TAGCGCTAA- ---------- ---------- ---------- ---------- ---------- ----------
G. crassaformis          A-------TA TTAGCATAAA GACTCGTCTT TAGCGCTAA- ---------- ---------- ---------- ---------- ---------- ----------
N. incompta I            A-------TC GTC--TTTTG AATTCTTTAA GGACATGTCG TTTTTAATGA CA----TCTT TTAGATGGAT GATTC----- ---------- ----------
N. incompta II           A-------TC GTC--TTTTG AAATCTTTAA GCACATGTCG TTTCTAATGA CA----TCTT TTAGATGAAT GATTC----- ---------- ----------
G. glutinata Ia1         A-------TA TAGCGCGCTT GCGCGTCTA- ---------- ---------- ---------- ---------- ---------- ---------- ----------
G. glutinata Ia2         A-------TA TAGCATGTAC TTCGGTGCGT GTCTA----- ---------- ---------- ---------- ---------- ---------- ----------
G. glutinata Ia3         A-------TA TAGCAACTTT CGGGTTGTCT A--------- ---------- ---------- ---------- ---------- ---------- ----------
C. nitida                A-------TA TGATTGCTCT TCGGAGCTTT TA-------- ---------- ---------- ---------- ---------- ---------- ----------
G. uvula                 A-------TA TGTGAC-TCT TC-GGA-GTT TG-CA----- ---------- ---------- ---------- ---------- ---------- ----------
B. variabilis            A-------TC --TCATACAT --CGCTTCGG CATG-T---G TG-------- ---------- ---------- ---------- ---------- ----------
S. globigerus            A-------TC --TCATACAT --CGCTTCGG CATG-T---G TG-------- ---------- ---------- ---------- ---------- ----------
B. alata                 A-------TC TCTTATGGCA TTTCGGTGCT GTACAG---- ---------- ---------- ---------- ---------- ---------- ----------
G. vivans                A-------TT AGCTACACTC TCGAGTGTCA GCT------- ---------- ---------- ---------- ---------- ---------- ----------
C. porrectus             A-------TT ATAATTTTTT AATTGGAATA AATTTTTATT AATTTATTTT TTTTATTTTT TAT------- ---------- ---------- ----------
C. ovoidea               ATCAACTTTT TACTTTTTTT ATCATCTCTC ATTGATGATT AAGAGAATGT ATGAGAGTT- ---------- ---------- ---------- ----------
G. opercularis           A-------TC CATATATTTT ATATATG--- ---------- ---------- ---------- ---------- ---------- ---------- ----------
E. aculeatum             C-------GT ATACTATATG TATA------ ---------- ---------- ---------- ---------- ---------- ---------- ----------
E. vitrea                A-------TT AATTTCTGCA GCTTCGGCTC AGTTATT--- ---------- ---------- ---------- ---------- ---------- ----------
H. germanica             T-------AC ACATACT--- ACGGTGTGT- ---------- ---------- ---------- ---------- ---------- ---------- ----------
P. mediterranensis       A-------TC TGAGTTGACA TCTCTGTATG TTTCTCA--- ---------- ---------- ---------- ---------- ---------- ----------
S. fusiformis            T-------AT TAATTTCTGG CCTCCGGGTC GATTTT---- ---------- ---------- ---------- ---------- ---------- ----------
V. fragilis              ATTAATATTT CACAAGTACT TCGGTATCTT TGTCGATTTA TT-------- ---------- ---------- ---------- ---------- ----------
A. pseudocassis          A-------TT AAAATGCTTT TCTTGCGTGC AAGAGAACAT TTT------- ---------- ---------- ---------- ---------- ----------
Spiroplectammina sp.     T-------AT TAAAAGAAAA ATTTTAATTT TC-------- ---------- ---------- ---------- ---------- ---GGATTAA ATATTTTTTT
Textularia sp.           A-------TT ATATTGCATT CGATTTAATC GTATTGTCAA TAT------- ---------- ---------- ---------- ---------- ----------
S. limosum               T-------TA ACACCTTTTT TATTTTTTTA ATCTTATGTG T--------- ---------- ---------- ---------- ---------- ----------
G. antarctica            ATTATAAAGT AAATCTTTGT GTCATTCGTG GCTCAAGCGA TTTTTTTAT- ---------- ---------- ---------- ---------- ----------
D. aphelis               A-------TT AATACGTTTT GCTTCGGTAA CATCGTATT- ---------- ---------- ---------- ---------- ---------- ----------
P. peruviana             CGCATAATAG AATTTATTTT ATTA------ ---------- ---------- ---------- ---------- ---------- ---------- ----------
M. secans                C-------AC TAATATAATT TATTATATT- ---------- ---------- ---------- ---------- ---------- ---------- ----------
Quinqueloculina sp.      A-------AC TAATATATTT TTAATAT--- ---------- ---------- ---------- ---------- ---------- ---------- ----------
N. haylinosphaira        T-------CG TTTTCATATG GTATTGTATC AATGCGTTTT TCCTTTTTGG AGAAATGTAC ATATGTACTA TTATGCAAAA C--------- ----------
M. fusca                 A-------GT ACAATAAATT TATTTGTTTG ---------- ---------- ---------- ---------- ---------- ---------- ----------
T. alba                  C-------AT GTGAACAGCT TTTTGACTAG AATTTTTTGA TTTTAGTTTT AAGAGGCGTT CT-------- ---------- ---------- ----------
A. mexicana              A-------TT AAAAATGTTT GTAATTTATT TTATAAACTT ATTTTT---- ---------- ---------- ---------- ---------- ----------
A. triangularis          A-------AA ATGTAATTTA TTATATTCAT TTATAACATA TT-------- ---------- ---------- ---------- ---------- ----------
A. rara                  A-------AA ATGTATTATW ATTTATAATT TTATTATTAT ATTTTATTAA T--------- ---------- ---------- ---------- ----------
```

```
E. scabrum            T-------AT ATAT------ ---------- ---------- ---------- ---------- ---------- ---------- ---------- ----------
N. venosus            A-------TT GTATAACACT WTTTAYGTGT TATAC----- ---------- ---------- ---------- ---------- ---------- ----------
B. marginata          A-------TT AGCACTTAGC TTCGGCGACG TGTT------ ---------- ---------- ---------- ---------- ---------- ----------
Trochammina sp.       A-------TT AATGCTAATC ATAATGCACA TTTGTGTATT ATTGATTTAG TATT------ ---------- ---------- ---------- ----------
Peneroplis sp.        A-------GT AATATTATTA TATAAT---- ---------- ---------- ---------- ---------- ---------- ---------- ----------
S. orbiculus          A-------GT ATATAAAAAT ATATATTTTT ATA------- ---------- ---------- ---------- ---------- ---------- ----------
Allogromia sp.        T-------AT AAGACTATAT ATAATTTTTT TTAAAATTAT ATATAGCAAT TCTT------ ---------- ---------- ---------- ----------

                      201        211        221        231        241        251        261        271        281        291      300
                      |          |          |          |          |          |          |          |          |          |        |
G. siphonifera Ia1    ---------- ---------T TACAAAAGAT AACA-GA--- ---------- ---------- -TCTTTCATG ATCATGTGAT GGGTGGTGCA TGGCCGTTCT
G. siphonifera Ia2    ---------- ---------T TACAAAAGAT AACA-GA--- ---------- ---------- -TCTTTCATG ATCATGTGAT GGGTGGTGCA TGGCCGTTCT
G. siphonifera IIa1   ---------- -------AAA GACAAAAGAT AACA-GA--- ---------- ---------- -TCTTTCATG ATTATGTGAT GGGTGGTGCA TGGCCGTTCT
G. siphonifera IIa2   ---------- -------AAA GACAAAAGAT AACA-GA--- ---------- ---------- -TCTTTCATG ATTATGTGAT GGGTGGTGCA TGGCCGTTCT
G. siphonifera IIa3   ---------- -------AAA GACAAAAGAT AATA-GA--- ---------- ---------- -TCTTTCATG ATTATGTGAT GGGTGGTGCA TGGCCGTTCT
G. siphonifera IIa    ---------- -------AAA GACAAAAGAT AACA-GA--- ---------- ---------- -TCTTTCATG ATTATGTGAT GGGTGGTGCA TGGCCGTTCT
G. siphonifera IIb    ---------- -------AAA GACAAAAGAT AACA-GA--- ---------- ---------- -TCTTTCATG ATTATGTGAT GGGTGGTGCA TGGCCGTTCT
G. calida             ---------- ---------A GACAAAAGAT AACA-GA--- ---------- ---------- -TCTTTCATG ATTATGTGGT GGGTGGTGCA TGGCCGTTCT
O. universa I         ---------- ---------T TACAAAATCT AACG-GC--- ---------- ---------- -TCTTACATG ATTATGTGAT AGGTGGTGCA TGGCCGTTCT
O. universa III       ---------- ---------A GGCAAAAACT AATA-GC--- ---------- ---------- -TCTTTTATG ATTATGTGAT AGGTGGTGCA TGGCCGTTCT
G. sacculifer         ---------- ---------- TAACAAAACT AACA-GC--- ---------- ---------- -TCTTTCAAG ATTATGTGGT AGGTGGTGCA TGGCCGTTCT
G. ruber pink         ---------- --------G- TTTTAAACCT AATG-GC--- ---------- ---------- -TCTTTCATG ATTATATGAT AGGTGGTGCA TGGCCGTTCT
G. ruber Ia           ---------- --------G- TTTTAAACCT AATG-GC--- ---------- ---------- -TCTTTCATG ATTATATGAT AGGTGGTGCA TGGCCGTTCT
G. ruber Ib1          ---------- --------G- TTTTAAACCT AATG-GC--- ---------- ---------- -TCTTTCATG ATTATATGAT AGGTGGTGCA TGGCCGTTCT
G. ruber Ib2          ---------- --------G- TTTTAAACCT AATG-GC--- ---------- ---------- -TCTTTCATG ATTATATGAT AGGTGGTGCA TGGCCGTTCT
G. ruber IIa          ---------- --------G- TTTTAAACTT AATA-GC--- ---------- ---------- -TCTTTCATG ATTATATGAT AGGTGGTGCA TGGCCGTTCT
G. conglobatus        ---------- --------G- TTCTAAACCT AATA-GC--- ---------- ---------- -TCTTTCATG ATTATATGAT AGGTGGTGCA TGGCCGTTCT
G. rubescens (pink)   ---------- --------GT TTTTAAACTT AACA-GC--- ---------- ---------- -TCTTTCATG ATTATATGAT AGGTGGTGCA TGGCCGTTCT
G. bulloides Ia       ---------- ---------A TG-GTAAAGT TGAA-GT--- ---------- ---------- -TCTTTCATG ATCTTGTGGC AGGTGGTGCA TGGCCGTTCT
G. bulloides Ib       ---------- ---------A TG-GTAAAGT TGAA-GT--- ---------- ---------- -TCTTTCATG ATCTTGTGGT AGGTGGTGCA TGGCCGTTCT
G. bulloides IIa      ---------- ---------T TG-AGAGAGT TGAA-GT--- ---------- ---------- -TCTTTCATG ATCTTGTGAG AGGTGGTGCA TGGCCGTTCT
G. bulloides IIb      ---------- ---------T TG-AGAGAGT TGAA-GT--- ---------- ---------- -TCTTTCATG ATCTTGTGAG AGGTGGTGCA TGGCCGTTCT
G. bulloides IIc      ---------- ---------A TG-AGAGAGT TGAA-GT--- ---------- ---------- -TCTTTCATG ATCTTGTGAG AGGTGGTGCA TGGCCGTTCT
G. bulloides IId      ---------- ---------T TG-AGAGAGT TGAA-GT--- ---------- ---------- -TCTTTCATG ATCTTGTGAG AGGTGGTGCA TGGCCGTTCT
G. bulloides IIe      ---------- ---------T TG-AGAAAGT TGAA-GT--- ---------- ---------- -TCTTTCATG ATCTTGTGAG AGGTGGTGCA TGGCCGTTCT
T. quinqueloba Ia     ATCAGGTCGT ACATTG---T AT-TCAATGA GAAA-GT--- ---------- ---------- -TCTTTTATG ATTATGTGGT AGGTGGTGCA TGGCCGTCTT
T. quinqueloba Ib     ATCAGGTCGT ACATTG---T AT-TCAATGA GAAA-GT--- ---------- ---------- -TCTTTTATG ATTATGTGGT AGGTGGTGCA TGGCCGTCTT
T. quinqueloba IIa    ---------- ---------T AT-TAAATAT GAAA-GT--- ---------- ---------- -TCTTTTATG ATTATGTGAT AGGTGGTGCA TGGCCGTCCT
T. quinqueloba IIb    ---------- ---------T AT-TAAATAT GAAA-GT--- ---------- ---------- -TCTTTTATG ATTATGTGAT AGGTGGTGCA TGGCCGTCCT
T. quinqueloba IIc    ---------- ---------T AT-TAAATAT GAAA-GT--- ---------- ---------- -TCTTTTATG ATTATGTGAT AGGTGGTGCA TGGCCGTCCT
T. quinqueloba IId    ---------- ---------T AT-TAAATAT GAAA-GT--- ---------- ---------- -TCTTTTATG ATTATGTGAT AGGTGGTGCA TGGCCGTCCT
G. falconensis        ---------- ---------G AG-ACAAAAT TTTTGGC--- ---------- ---------- -TCTTTCATG ATTATGTGAT AGGTGGTGCA TGGCCGTTCT
H. pelagica           ---------- ---------T GTGTCAACGT GCCG-GT--- ---------- ---------- -TCTTTCATG ATTGTGTGAT AGGTGGTGCA TGGCCGTTCT
G. menardii           ---------- ---------G TGTAAACTGT TCTA-AGGCT TCATACACAG GCACTA---- -TCTCTCATG ATTATGTTGC AGGTGGTGCA TGGCCGTTGT
G. ungulata           ---------- -------TT- ACACACAGGC ACTA------ ---------- ---------- -TCTCTCATG ATTATGTTGT AGGTGGTGCA TGGCCGTTGT
G. hirsuta            ---------- ---------G TTATAAAGAT GCTA-GT--- ---------- ---------- -CCTTTCATG ATTATGTGAT AGGTGGTGCA TGGCCGTTCT
G. scitula            ---------- ---------T ATTCAAATAT GCTA-GT--- ---------- ---------- -CCTTTCATG ATTATGTGGT AGGTGGTGCA TGGCCGTTCT
G. truncatulinoides   ---------- ---------T ACACAAAGAT GCTA-GT--- ---------- ---------- -CCTTTCATG ATCGTTTGAT AGGTGGTGCA TGGCCGTTCT
N. pachyderma I       ---------- ---------G TGTTAAATAT GCTA-GT--- ---------- ---------- -CCTTTCATG ATTATGTGAT AGGTGGTGCA TGGCCGTTCT
N. pachyderma II      ---------- ---------G TGTTAAATAT GCTA-GT--- ---------- ---------- -CCTTTCATG ATTATGTGAT AGGTGGTGCA TGGCCGTTCT
N. pachyderma III     ---------- ---------G TGTTAAATAT GCTA-GT--- ---------- ---------- -CCTTTCATG ATTATGTGAT AGGTGGTGCA TGGCCGTTCT
N. pachyderma IV      ---------- ---------G TGTTAAATAT GCTA-GT--- ---------- ---------- -CCTTTCATG ATTATGTGAT AGGTGGTGCA TGGCCGTTCT
N. pachyderma V       ---------- ---------G TGTTAAATAT GCTA-GT--- ---------- ---------- -CCTTTCATG ATTATGTGAT AGGTGGTGCA TGGCCGTTCT
N. pachyderma VI      ---------- ---------G TGTTAAATAT GCTA-GT--- ---------- ---------- -CCTTTCATG ATTATGTGAT AGGTGGTGCA TGGCCGTTCT
N. pachyderma VII     ---------- ---------G TGTTAAATAT GCTA-GT--- ---------- ---------- -CCTTTCATG ATTATGTGAT AGGTGGTGCA TGGCCGTTCT
N. dutertrei_C        ---------- ---------G TGTTAAATAT GCTA-GT--- ---------- ---------- -CCTTTCATG ATTATGTGAT AGGTGGTGCA TGGCCGTTCT
N. dutertrei_Ib       ---------- ---------G TGTTAAATAT GCTA-GT--- ---------- ---------- -CCTTTCATG ATTATGTGAT AGGTGGTGCA TGGCCGTTCT
P. obliquiloculata_BR ---------- ---------G TGTTAAATAT GCTA-GT--- ---------- ---------- -CCTTTCATG ATTATGTGAT AGGTGGTGCA TGGCCGTTCT
P. obliquiloculata_AS ---------- ---------G TGTTAAATAT GCTA-GT--- ---------- ---------- -CCTTTCATG ATTATGTGAT AGGTGGTGCA TGGCCGTTCT
G. inflata            ---------- ---------T TGTTAAATAT GCTA-GT--- ---------- ---------- -CCTTTCATG ATTATGTGAT AGGTGGTGCA TGGCCGTTCT
G. crassaformis       ---------- ---------T TGTTAAATAT GCTA-GT--- ---------- ---------- -CCTTTCATG ATTATGTGAT AGGTGGTGCA TGGCCGTTCT
N. incompta I         ---------- ---------G TG-TAAATAT GCTA-GT--- ---------- ---------- -TCTTTCATG ATTATGTGAT AGGTGGTGCA TGGCCGTTCT
N. incompta II        ---------- ---------G TG-TAAATAT GCTA-GT--- ---------- ---------- -TCTTTCATG ATTATGTGAT AGGTGGTGCA TGGCCGTTCT
G. glutinata Ia1      ---------- ---------T TGTTAAATAT GCTA-GT--- ---------- ---------- -CCTTTCATG ATTATGTGAT AGGTGGTGCA TGGCCGTTCT
G. glutinata Ia2      ---------- ---------T TGTTAAATAT GCTA-GT--- ---------- ---------- -CCTTTCATG ATTATGTGAT AGGTGGTGCA TGGCCGTTCT
G. glutinata Ia3      ---------- ---------T TGTTAAATAT GCTA-GT--- ---------- ---------- -CCTTTCATG ATTATGTGAT AGGTGGTGCA TGGCCGTTCT
C. nitida             ---------- ---------T TGTTAAATAT GCTA-GT--- ---------- ---------- -CCTTTCATG ATTATGTGAT AGGTGGTGCA TGGCCGTTCT
G. uvula              ---------- ---------T TGTTAAATAT GCTA-GT--- ---------- ---------- -CCTTTCATG ATTATGTGAT AGGTGGTGCA TGGCCGTTCT
B. variabilis         ---------- ---------G GTATAAATAT GCTA-GT--- ---------- ---------- -CCTTTCATG ATTATGTGAT AGGTGGTGCA TGGCCGTTCT
S. globigerus         ---------- ---------G GTATAAATAT GCTA-GT--- ---------- ---------- -CCTTTCATG ATTATGTGAT AGGTGGTGCA TGGCCGTTCT
B. alata              ---------- ---------A GTATAAATAT GCTA-GT--- ---------- ---------- -CCTTTCATG ATTATGTGAT AGGTGGTGCA TGGCCGTTCT
G. vivans             ---------- ---------A TGTTAAATAT GCTA-GT--- ---------- ---------- -CCTTTCATG ATTATGTGAT AGGTGGTGCA TGGCCGTTCT
C. porrectus          ---------- ---------A TGTTAAATAT GCTA-GT--- ---------- ---------- -CCTTTCATG ATTATGTGAT AGGTGGTGCA TGGCCGTTCT
C. ovoidea            ---------- ---------G TGTTAAATAT GCTA-GT--- ---------- ---------- -CCTTTCATG ATTATGTGAT AGGTGGTGCA TGGCCGTTCT
G. opercularis        ---------- ---------G TGTCAAAAAT GCTA-GT--- ---------- ---------- -CCTTTCATG ATTATGTGAT AGGTGGTGCA TGGCCGTTCT
E. aculeatum          ---------- ---------C GTTCAAAGAT GCTA-GT--- ---------- ---------- -TCTTTCATG ATTATGTGAT AGGTGGTGCA TGGCCGTTCT
E. vitrea             ---------- ---------A TGTTAAATAT GCTA-GT--- ---------- ---------- -CCTTTCATG ATTATGTGAT AGGTGGTGCA TGGCCGTTCT
H. germanica          ---------- ---------G CATGAAATAT GCTA-GT--- ---------- ---------- -TCTTTCATG ATTATGTGAT AGGTGGTGCA TGGCCGTTCT
P. mediterranensis    ---------- ---------G TGTTAAATAT GCTA-GT--- ---------- ---------- -CCTTTCATG ATTATGTGAT AGGTGGTGCA TGGCCGTTCT
S. fusiformis         ---------- ---------A TGTTAAATAT GCTA-GT--- ---------- ---------- -CCTTTCATG ATTATGTGAT AGGTGGTGCA TGGCCGTTCT
V. fragilis           ---------- ---------A TGTTAAATAT GCTA-GT--- ---------- ---------- -CCTTTCATG ATTATGTGAT AGGTGGTGCA TGGCCGTTCT
A. pseudocassis       ---------- ---------A TGTCAAATAT GCTA-GT--- ---------- ---------- -CCTTTCATG ATTATGTGAT AGGTGGTGCA TGGCCGTTCT
Spiroplectammina sp.  TTT------- ---------A TGTTAAATAT GCTA-GT--- ---------- ---------- -CCTTTCATG ATTATGTGAT AGGTGGTGCA TGGCCGTTCT
Textularia sp.        ---------- ---------A TGTTAAATAT GCTA-GT--- ---------- ---------- -CCTTTCATG ATTATGTGAT AGGTGGTGCA TGGCCGTTCT
S. limosum            ---------- ---------T AAGCACATAT GCTA-GT--- ---------- ---------- -CCTTTCATG ATTGTATGAT AGGTGGTGCA TGGCCGTTCT
G. antarctica         ---------- ---------A TGTTAAATAT GCTA-GT--- ---------- ---------- -CCTTTCATG ATTATGTGAT AGGTGGTGCA TGGCCGTTCT
D. aphelis            ---------- ---------A TGTTAAATAT GCTA-GT--- ---------- ---------- -CCTTTCATG ATTATGTGAT AGGTGGTGCA TGGCCGTTCT
P. peruviana          ---------- ---------T GTACAAATAT GATA-GT--- ---------- ---------- -CCTTTCATG ATTATATGAT AGGTGGTGCA TGGCCGTTCT
M. secans             ---------- ---------A GTACAAATAT GATA-GT--- ---------- ---------- -CCTTTCATG ATTATATGAT AGGTGGTGCG TGGCCGTTCT
Quinqueloculina sp.   ---------- ------ATTA GTACAAATAT GATA-GT--- ---------- ---------- -CCTTTCATG ATTATATGAT AGGTGGTGCA TGGCCGTTCT
N. haylinosphaira     ---------- ---------A TTTCAAATAT GCTA-GT--- ---------- ---------- -CCTTTCATG ATTGTGTGAT AGGTGGTGCA TGGCCGTTCT
M. fusca              ---------- ---------T ATATAAATAT GATA-GT--- ---------- ---------- -CCTTTCATG ATTATGTGAT AGGTGGTGTA TGGCCGTTCT
T. alba               ---------- ---------C ATATAAATAT GCTA-GT--- ---------- ---------- -CCTTTCATG ATTATGTGAT AGGTGGTGCA TGGCCGTTCT
A. mexicana           ---------- ---------A TGTTAAATAT GCTA-GT--- ---------- ---------- -CCTTTCATG ATTATGTGAT AGGTGGTGCA TGGCCGTTCT
A. triangularis       ---------- ------ACAT TATCAAATAT GCTA-GT--- ---------- ---------- -CCTTTCATG ATTGTGTGAT AGGTGGTGCA TGGCCGTTCT
A. rara               ---------- ------ACGT TATCAAATAT GCTA-GT--- ---------- ---------- -CCTTTCATG ATTGTGTGAT AGGTGGTGCA TGGCCGTTCT
E. scabrum            ---------- ---------A TATTAAATAT GCTA-GT--- ---------- ---------- -CCTTTCATG ATTATGTGAT AGGTGGTGCA TGGCCGTTCT
```

```
N. venosus                ---------- ---------A TGTTAAATAT GCTA-GT--- ---------- ---------- -CCTTTCATG ATTATGTGAT AGGTGGTGCA TGGCCGTTCT
B. marginata              ---------- ---------A TGTTAAATAT GCTA-GT--- ---------- ---------- -CCTTTCATG ATTATGTGAT AGGTGGTGCA TGGCCGTTCT
Trochammina sp.           ---------- ---------A TGTTGAATAT GCTA-GT--- ---------- ---------- -CCTTTCATG ATTATGTGAT AGGTGGTGCA TGGCCGTTCT
Peneroplis sp.            ---------- -------ATT GCATAAAAAT GATA-GT--- ---------- ---------- -CCTTTCATG ATTATATGAT AGGTGGTGCA TGGCCGTTCT
S. orbiculus              ---------- ---------T GCATAAAAAT GATA-GT--- ---------- ---------- -CCTTTCATG ATTATATGAT AGGTGGTGCA TGGCCGTTCT
Allogromia sp.            ---------- ---------A TATCAAATAT GCTA-GT--- ---------- ---------- -CCTTTCATG ATTGCGTGAT AGGTGGTGCA TGGCCGTTCT

                          301        311        321        331        341        351        361        371        381        391      400
                          |          |          |          |          |          |          |          |          |          |        |
G. siphonifera Ia1        TAGTTCGTGG AGTGATCTGT CTGCTTAATT GCGT-----T TC-AAATTTT ATATTTGTTA ACAT-GCGAG TCTTCAATGA CGGCCGCTCC ACCTTATAGA
G. siphonifera Ia2        TAGTTCGTGG AGTGATCTGT CTGCTTAATT GCGT-----T TC-AAATTTT ATATTTGTTA ACAT-GCGAG TCTTCAATGA CGGCCACTCC ACCTTATAGA
G. siphonifera IIa1       TAGTTCGTGG AGTGATCTGT CTGCTTAATT GCGT-----T TC-AAATATA TTTTATTATA TA-C-GAGTC TACAAA---- -GGAGTGGTT CA-TT--AAC
G. siphonifera IIa2       TAGTTCGTGG AGTGATCTGT CTGCTTAATT GCGT-----T TC-AAATATA TTTTATTATA TAAC-GAGTC TACAAA---- -GGAGTGGTT CA-TT--AAC
G. siphonifera IIa3       TAGTTCGTGG AGTGATCTGT CTGCTTAATT GCGT-----T TC-AAATATA TTTTATTATA TAAC-GAGTC TACAAA---- -GGAGTGGTT CA-TT--AAC
G. siphonifera IIa        TAGTTCGTGG AGTGATCTGT CTGCTTAATT GCGT-----T TC-AAATATA TTTTATTATA TAAC-GAGTC TACAAA---- -GGAGTGGTT CA-TT--AAC
G. siphonifera IIb        TAGTTCGTGG AGTGATCTGT CTGCTTAATT GCGT-----T TC-AAATATA CTTTTTTT-A CAAC-GAGTC TACAAA---- -GGAGTGGTT CAGTTACAAC
G. calida                 TAGTTCGTGG AGTGATCTGT CTGCTTAATT GCGT-----T TC-AAATGTA TTTTTGATTT CT-C-AAACG AGTCTA---- -CGAAGATGT CTATTCATAA
O. universa I             TAGTTCGTGG AGTGATCTGT CTGCTTAATT GCGT-----T TG-GATACAA CAATTTT--- -----GGAAT CGCGGCAATG TTCAGCTTAA TGGCGTCGAT
O. universa III           TAGTTCGTGG AGTGATCTGT CTGCTTAATT GCGT-----T TC-AAAACAA CTGATACACA GAGTCTTGGC ACAGTTCAGT TTAATGGCGT CGAGTATCTT
G. sacculifer             TAGTTCGTGA AGTGATTCGT TCGCCCAATC GCAT-----T TCCAAA-CAA CTAACAACTC A----GAATG CTGGGCAATA GGACGTTTAA AGTGGCTCCT
G. ruber pink             TAGTTCGTGG AGTGATCTGT CTGCTTAATT GCGT-----T TC-AAATTTT -TATTT---- -----GAGGA CCAGGTTTTG GTTTGGGTAG TGGTCCTACG
G. ruber Ia               TAGTTCGTGG AGTGATCTGT CTGCTTAATT GCGT-----T TC-AAAATTT -AACTT---- -----GAGGA CCGGGTTTTG GTTTGGCTAG TGGTTCTACG
G. ruber Ib1              TAGTTCGTGG AGTGATCTGT CTGCTTAATT GCGT-----T TC-AAAATTT -AACTT---- -----GAGGA CCGGGTTTTG GTTTGGCTAG TGGTCCTACG
G. ruber Ib2              TAGTTCGTGG AGTGATCTGT CTGCTTAATT GCGT-----T TC-AAATTTT TAACC----- -----GAGGA CCGGGTTTTG GTTTGGCTAG TGGTCCTACG
G. ruber IIa              TAGTTCGTGG AGTGATCTGT CTGCTTAATT GCGT-----T TC-AAAATCT AAACCT---- -----GAGCG ACAAGGTGCT GCTATCGAAA CTGTCTTTGA
G. conglobatus            TAGTTCGTGG AGTGATCTGT CTGCTTAATT GCGT-----T TC-AAA-TAT GAATTTGAGT ACCAGGTCCC AAGTGTTTCA AGTAAATCTG TCTCTGAGTG
G. rubescens (pink)       TAGTTCGTGG AGTGATCTGT CTGCTTAATT GCGT-----T TC-AAATTTT TTTTCATTTC TC---AGGAG TCGGGTTTAC GTGTCATATT TACAATTGAG
G. bulloides Ia           TAGTTCGTGT AGTGATACGT CTGCCTAATC GCGT-----C AC-GATAACC TATTTCTCGA CAATCCAAAG ATC-TTCG-T GCAGTATCTA TTGGTACGGT
G. bulloides Ib           TAGTTCGTGT AGTGATACGT CTGCCTAATC GCGT-----C AC-GATAAGC TATTTCTCGA CAATCCAAAA ATC-ACGGCG GGAGGTTCTA CTGGTACGGG
G. bulloides IIa          TAGTTCGTGT AGTGATATGT CTGCCTAATC GCGT-----C AC-GATAACC TATTGGTCGA CAACCCAATT ATC-ACAACT GCAGCATAAC TCCCCTTGGG
G. bulloides IIb          TAGTTCGTGT AGTGATATGT CTGCCTAATC GCGT-----C AC-GATAACC TATTGGTCGA CAACCCAATT ATC-ACAACT GCAGCATAAC TCCCCTTGGG
G. bulloides IIc          TAGTTCGTGT AGTGATATGT CTGCCTAATC GCGT-----C AC-GATAACC TATTGGTCGA CAACCCAATT ATC-ACAACT GCAGCATAAC TCCCCTTGGG
G. bulloides IId          TAGTTCGTGT AGTGATATGT CTGCCTAATC GCGT-----C AC-GATAACC TATTGGTCGA CAACCCAATT ATC-ACAACT GCAGCATAAC TCCCCTTGGG
G. bulloides IIe          TAGTTCGTGT AGTGATATGT CTGCCTAATC GCGT-----C AC-GATAACC TATTGGTCGA CAACCCAATT ATC-ACAACT GCAGCATAAC TCCCCTTGGA
T. quinqueloba Ia         TAATTCGTGG AGTGATCTGT CTGCTTAATT GCGC-----A TT-GCAAATT GTAATTGATC TTTGAACAGA TCCGTCTT-- TTTATGTTG- -AATAGTACG
T. quinqueloba Ib         TAATTCGTGG AGTGATCTGT CTGCTTAATT GCGC-----A TT-GCAAATT GTAATTAATC TGGCTTGGAT GATCGTTTGA TTTCGATTTG AATGCCATTT
T. quinqueloba IIa        TAATTCGTGG AGTGATTTGT CTGCTTAATT GCGC-----A TT-GCAAATT GTAATTGATC TTTTACCAGT CTCGTTCCGA TTGATGTTGT GAATATTGTA
T. quinqueloba IIb        TAATTCGTGG AGTGATTTGT CTGCTTAATT GCGC-----A TT-GCAAATT GTAATTGATC TTTTACCAGT CTCGTTCTGA TTTATGTTGT -AATATTGTA
T. quinqueloba IIc        TAATTCGTGG AGTGATTTGT CTGCTTAATT GCGC-----A TT-GCAAATT GTAATTGATC TTTTACCAGT CTCGTTCTGA TTTATGTTGT -AATATTGTA
T. quinqueloba IId        TAATTCGTGG AGTGATTTGT CTGCTTAATT GCGC-----A TT-GCAAATT GTAATTGATC TTTTACCAGT CTCGTTCTGA TTTTTGTTGT GAATATTGTA
G. falconensis            TAGTTCGTGG AGTGATCTGT CTGCCCAATC GCGT-----T TC-ATAAATC TTAAGCTCAA AAGGCAGTCT TACCTTCGGG TTGAGTTTTG CTGGGAGA--
H. pelagica               TAGTTCGTGG AGTGATCTGT CTGCTTAATT GCGT-----T TC-AAATACT TCGAGGATAT AAACGCATTC ATTGTGAGTT GCTATTTAGC TGGGAATAGC
G. menardii               TAGTTCGTGG AGTAATCTGT CTGCTTAATT GCGT-----T TC-GTACAA- GTACTCACTC AATCCTGGAC GTATCTCAAT TATATGTGGC GGTG--TCTG
G. ungulata               TAGTTCGTGG AGTAATCTGT CTGCTTAATT GCGT-----T TC-GTACAAA GTACTCACTC AATCCTAGGC ATAGTTCAAT TATATGTGGC GGCATCTAAA
G. hirsuta                TAGTTCGTGG AGTGATCTGT CTGCTTAATT GCGT-----T TC-ACTAAGG --TCCAGTAA TC--TAAGGT ATGCTCGTCT ACTTATCTCA TAT---GACG
G. scitula                TAGTTCGTGG AGTGATCTGT CTGCTTAATT GCGT-----T TC-ACTAAGG --TCCCATAT AC--TAAAGG CATGTCGACC TCTCTTTAGG CTTAATTAAG
G. truncatulinoides       TAGTTCGTGG AGTGATCTGT CTGCCTAATT GCGT-----T TC-ACTAAGG TCCCATTCTT CATACGTATG AATAGCGTCT TAACTCACTT TTACCGCTAC
N. pachyderma I           TAGTTCGTGG AGTGATCTGT CTGCTTAATT GCGT-----T TC-ACTAAGG G-CCCATAAA TT--CAAGGT ATGTTAGCTA T-C-GCCGCT CTATG--GAC
N. pachyderma II          TAGTTCGTGG AGTGATCTGT CTGCTTAATT GCGT-----T TC-ACTAAGG G-CCCATAAA TT--CAAGGT ATGTTAGCTA T-C-GTCGCT CTATG--GAC
N. pachyderma III         TAGTTCGTGG AGTGATCTGT CTGCTTAATT GCGT-----T TC-ACTAAGG G-CCCATAAA TT--CAAGGT ATGTTAGCTA T-C-GTCGCT CTATG--GAC
N. pachyderma IV          TAGTTCGTGG AGTGATCTGT CTGCTTAATT GCGT-----T TC-ACTAAGG G-CCCATAAA TT--CAAGGT ATGTTAGCTC T-C-GTCGCT CTATG--GAC
N. pachyderma V           TAGTTCGTGG AGTGATCTGT CTGCTTAATT GCGT-----T TC-ACTAAGG G-CCCATAAA TT--CAAGGT ATGTTAGCTA T-C-GTCGCT CTATG--GAC
N. pachyderma VI          TAGTTCGTGG AGTGATCTGT CTGCTTAATT GCGT-----T TC-ACTAAGG G-CCCATAAA TT--CAAGGT ATGTTAGCTA T-C-GTCGCT CTATG--GAC
N. pachyderma VII         TAGTTCGTGG AGTGATCTGT CTGCTTAATT GCGT-----T TC-ACTAAGG G-CCCATAAA TT--CAAGGT ATGTTAGCTA T-CTGTCGCT CTATG--GAC
N. dutertrei_C            TAGTTCGTGG AGTGATCTGT CTGCTTAATT GCGT-----T TC-ACTAAGG G-CCCATAAA TT--CAAGGT ATGTTAGCTA T-C-GTTTCT CAATT--GAC
N. dutertrei_Ib           TAGTTCGTGG AGTGATCTGT CTGCTTAATT GCGT-----T TC-ACTAAGG G-CCCATAAA TT--CAAGGT ATGTTAGCTA T-C-GTTTCT CAATT--GAC
P. obliquiloculata_BR     TAGTTCGTGG AGTGATCTGT CTGCTTAATT GCGT-----T TC-ACTAAGG G-CCCATAAA TT--CAAGGT ATGTTAGCTA T-T-GTTTCC CTATT--GAC
P. obliquiloculata_AS     TAGTTCGTGG AGTGATCTGT CTGCTTAATT GCGT-----T TC-ACTAAGG G-CCCATAAA TT--CAAGGT ATGTTAGCTA T-T-GTTTCC CTATT--GAC
G. inflata                TAGTTCGTGG AGTGATCTGT CTGCTTAATT GCGT-----T TC-ACTAAGG G--CCATAAA TT--CAAGGT ATGTTAGCAA A-T-GCTGCT CTATT--GAC
G. crassaformis           TAGTTCGTGG AGTGATCTGT CTGCTTAATT GCGT-----T TC-ACTAAGG G-CCCATAAA TT--CAAGGT ATGTTAGCAA A-T-GCTGCT CTATT--GAC
N. incompta I             TAGTTCGTGG AGTGATCTGT CTGCTTAATT GCGT-----T TC-ACTAAGG -CCCCAAAGT TAG-CAAATT ATCAATCGTT ACAGAGTC-- -------GAC
N. incompta II            TAGTTCGTGG AGTGATCTGT CTGCTTAATT GCGT-----T TC-ACTATGG -CCCCAAAGT TAG-CAAGTT AT-CCAATCA TGACCTTCGT C------GAC
G. glutinata Ia1          TAGTTCGTGG AGTGATCTGT CTGCTTAATT GCGT-----T TC-AGATAAG GATCTATATT CTTTACAGAA --GTGTTGCG TAGTGC-ATA TGACCCCTCG
G. glutinata Ia2          TAGCTCGTGG AGTGATCTGT CTGCTTAATT GCGT-----T TC-AGATAAG GATCTATATT CTTTACA--- --GTGTCGTA GTGTTTTAAT TGACCCCTCT
G. glutinata Ia3          TAGTTCGTGG AGTGATCTGT CTGCTTAATT GCGT-----T TC-AGATAAG GATCTATATT CTTTACA--- --GTGTCGTA GTGTTTTAAT TGACCCCTCT
C. nitida                 TAGTTCGTGG AGTGATCTGT CTGCTTAATT GCGT-----T TCACTATAAG GATCTATATT CTTTACA--- --GTGTGCAG TGC-----AT TGACCCCTTC
G. uvula                  TAGTTCGTGG AGTGATCTGT CTGCTTAATT GCGT-----T TC-AAATTAC TTATAAGGTA GCAATACGCG ATGAAGCTTT CAATAGTCCT CACGGATTCA
B. variabilis             TAGTTCGTGG AGTGATCTGT CTGCTTAATT GCGT-----T TC-ACTATTG G-ATCTATAT ACCGTGCATG TGTTGCGGCA TT------GA CCCC--AT-T
S. globigerus             TAGTTCGTGG AGTGATCTGT CTGCTTAATT GCGT-----T TC-ACTATTG G-ATCTATAT ACCGTGCATG TGTTGCGGCA TT------GA CCCC--AT-T
B. alata                  TAGTTCGTGG AGTGATCTGT CTGCTTAATT GCGT-----T TC-ACTATTG G-ATCTATAT AC-CGTATAT GTGTTGCGGC TTT-----GA CCCCTTTATA
G. vivans                 TAGTTCGTGG AGTGATCTGT CTGCTTAATT GCGT-----T TC-ACTAAGG GCCTATAATT TAACGTGTGT TGCGGCACTT TGACCCCTCT TCACTGAGCG
C. porrectus              TAGTTCGTGG AGTGATCTGT CTGCTTAATT GCGT-----T TC-ACTATTG GGCTTATATA ATTACGTATG TTGTTAACGC TTTGACCCCT TTCTACGGAT
C. ovoidea                TAGTTCGTGG AGTGATCTGT CTGCTTAATT GCGT-----T TC-ACTAAGG GCCTATATCA ATATAGCATG TCTATGCAAT TCTAGCGCAT TGACCCCTCA
G. opercularis            TAGTTCGTGG AGTGATCTGT CTGCTTAATT GCGT-----T TC-ACTACGA ATCTACTTTA AACGTGTGTT TGTGAGATGG ACTGATCCCT CCCACCTCTC
E. aculeatum              TAGTTCGTGG AGTGATCTGT CTGCTTAATT GCGT-----T TC-ATATATA CGTACATATT TTCATATGT- ---------- ---------- ----------
E. vitrea                 TAGTTCGTGG AGTGATCTGT CTGCTTAATT GCGT-----T TC-ACTAAGG GCCTATAAAT TACGTGTGTT GCGGTACTTT GACCCCTCTT TTTTCGAGAA
H. germanica              TAGTTCGTGG AGTGATCTGT CTGCTTAATT GCGT-----T TC-ACTAAAG GCACATACAC ATAATATTGA TGCGTTGTGT GTATAATTTA TAATTATACG
P. mediterranensis        TAGTTCGTGG AGTGATCTGT CTGCTTAATT GCGT-----T TC-ACTAAGG TTCTATATTT AATGCATGTT GTCAGCGCAT TGACCCCTCG TTTTCTTCAT
S. fusiformis             TAGTTCGTGG AGTGATCTGT CTGCTTAATT GCGT-----T TC-ACTAAGG GCCTATAATT TAACGTGTGT TGCGGCACTT TGACCCCTCT TCGCGGAGCG
V. fragilis               TAGTTCGTGG AGTGATCTGT CTGCTTAATT GCGT-----T TC-ACTAAGG ATCTATTTAT TAACGTGTGT TGCGGCACTT TGACCCCTCA TTTTTATTAA
A. pseudocassis           TAGTTTGTGG AGTGATCTGT CTGCTTAATT GCGT-----T TC-ACTAAGG ACTCAGATAT CATGTGTGTT TGCAGCTGGC TCTGACCCTT CATTGTGTGT
Spiroplectammina sp.      TAGTTCGKGG AGNGATCTGT CTGCTTAATT GCGT-----T TC-ACTAAGG G-CTTATAAA TT---ACGTG TGCTGKGGGT ACCTTGACCC CTATATTGAA
Textularia sp.            TAGTTCGTGG AGTGATCTGT CTGCTTAATT GCGT-----T TC-ACTAAGG GCCTATAAAT TACGTGTGTT GCAGGTACTT TGACCCCTTT GGTTGAAATA
S. limosum                TAGTTCGTGG AGTGATCTGT CTGCTTAATT GCGT-----T TC-ATTACGA GCCTTTCATT AAACTTTTAA ATTTTTACAC TCATTTTTGA TCGTTAATTT
G. antarctica             TAGTTCGTGG AGTGATCTGT CTGCTTAATT GCGT-----T TC-ACTATTG GGCTTATATA ATTACGTATG CTGTTAACGC TTTGACCCCT TTCTACGGAT
D. aphelis                TAGTTCGTGG AGTGATCTGT CTGCTTAATT GCGT-----T TC-ACTAAGG GCCTATAAAT TTACGTGTGT TGCATCGCAC TTTGACCCCT TTCTTTAATT
P. peruviana              TAGTTCGTGG AGTAATCTGT CTGCTTAATT GCGT-----T TC-ACTAAAT ATAAAATAGT TCGTCTATTT TATTATATTC AATGTTCTGC CTTCACAGGA
M. secans                 TAGTTCGTGG AGTGATCTGT CTGCTTAATT GCGT-----T TC-AGTAAAA AATATATAAT TTAAATTTTT ATATATTTTG ACGTTCTGCC TTATTTTTAA
Quinqueloculina sp.       TAGTTCGTGG AGTGATCTGT CTGCTTAATT GCGT-----T TC-ATATTAT ATTAAATAAT ATTATTCAAT ATAATATTAT TAGCGTTCTG CCTTTTATAG
N. haylinosphaira         TAGTTCGTGG AGTGATCTGT CTGCTTAATT GCGT-----T TC-ACATATG GCTCGTACGT GTTTTATGCT AATACTGTGA CCTCATTGTT CTTTTACAGA
M. fusca                  TAGTTAGTGG AGTGATCTGT CTGCTTAATT GCGT-----T TC-TTATGAA TTAATACGAG TTTGTATTTA GTATTCTTTT ATAAGTTTAC TATTACTTTC
T. alba                   TAGTTCGTGG AGTGATCTGT CTGCTTAATT GCGT-----T TC-GCAATGG ACTTAATAAC AAGTTGCGCT CTTCTTTTCT TTGTGGACAT TTTGCCTTGT
A. mexicana               TAGTTNGTGG AGTGATCTGT CTGCTTAATT GCGT-----T TC-ACTAAGA ACTCTATTAA AATCGTATGC ATTGTTGACT TTATATTGAC CCCTTATCTT
A. triangularis           TAGTTCGTGG AGTGATCTGT CTGCTTAATT GCGT-----T TC-ATAATAG CTCCTTATAG ACTTTTCTAT TGATATCAGC CTTAATACTT GAGAATGATC
A. rara                   TAGTTCGTGG AGTGATCTGT CTGCTTAATT GCGT-----T TC-ATAATAG CTCCAAATAG ACTTTTCTAT TGATATCAGC CTTAWYACTT TTGAATTTAT
E. scabrum                TAGTTCGTGG AGTGATCTGT CAGCTTAATT GCGT-----T TC-ACTAAGG GCTTTTATAA ATTTAATGCG CAGTGTGTTG TTGTATTTTT ATACTCACAC
N. venosus                TAGTTCGTGG AGTGATCTGT CTGCTTAATT GCGT-----T TC-ACTAAGG GCCTATAAAT TTACGTATGT TGCGGCACTT TGACCCCTCT TAATTGAGCG
```

```
B. marginata           TAGTTCGTGG AGTGATCTGT CTGCCTAATT GCGT-----T TC-ACCAAGG GCCTATAATT TTACGTGTGT TGCGGCACCT TGACCCCTCT TTTTTTAAAG
Trochammina sp.        TAGTTCGTGG AGTGATCTGT CTGCTTAATT GCGT-----T TC-ACTAAGG GCTTATAAAT TACGTGTGTT GCATGTACTT TGACCCCT-A ATCTGAAATA
Peneroplis sp.         TAGTTCGTGG AGTGATTTGT CTGCTTAATT GCGT-----T TC-AGATATA TAATTATATA ATATATTATA TAGTAATATA TAATTTAATA TTGTGCTGCC
S. orbiculus           TAGTTCGTGG AGTGATTTGT CTGCTTAATT GCGT-----T TC-AGTAAAT AAAATATATG ATATATTATA ATATTTAGTT CTGCCTTTAT GGATTTAAAG
Allogromia sp.         TAGTTCGTGG AGTGATCTGT CTGCTTAACT GCGT-----T TC-ACTATAA TGAGTATATA TTGAATACTT TGTTTGCACA TAAAGTTGCT GCATTGTTTT

                       401        411        421        431        441        451        461        471        481        491      500
                       |          |          |          |          |          |          |          |          |          |        |
G. siphonifera Ia1     CATTATATGA TATGCACATT TGTGTATTTG ATTATAACTT GTCTGGA--G TCTGG-CTCG ATTTTTTT-- ---------- ---------- ----------
G. siphonifera Ia2     CATTATATGA TATGCACATT TGTGTATTTG ATTATAACTT GTCTGGA--G TCTGG-CTCG ATTTTTTT-- ---------- ---------- ----------
G. siphonifera IIa1    AGACAATCAT ATCTTTGAGC GTCT------ -GGAATCTAC TCTATTT--- ---------- ---------- ---------- ---------- ----------
G. siphonifera IIa2    AGACAATCAT ATCTTTGAGC GTCT------ -GGAATCTAC TCTATTT--- ---------- ---------- ---------- ---------- ----------
G. siphonifera IIa3    AGACAATCAT ATCTTTGAGC GTCT------ -GGAATCTAC TCTATTT--- ---------- ---------- ---------- ---------- ----------
G. siphonifera IIa     AGACAATCAT ATCTTTGAGC GTCT------ -GGAATCTAC TCTATTT--- ---------- ---------- ---------- ---------- ----------
G. siphonifera IIb     AGACAATTAA TTCTTGTGTT TTG-AACGTT TGGAATGTAC TCTATTT--- ---------- ---------- ---------- ---------- ----------
G. calida              CAAGCACTTC ATTTATGATT TGT-TTGGAA TACGACTCTT TC-------- ---------- ---------- ---------- ---------- ----------
O. universa I          AAAATTAACA TTATGTTGAC AATTAGTCCA ACTCGGTCCA GCTC------ ---------- ---------- ---------- ---------- ----------
O. universa III        TGATTTCGAC AGTAGTCCAA CTCGGTTCGC CAGG------ ---------- ---------- ---------- ---------- ---------- ----------
G. sacculifer          TACACCTTAC AAAGGAATGG CTGATGAGAA ACAACTCGAT CCGCTCAGC- ---------- ---------- ---------- ---------- ----------
G. ruber pink          ATGACTGTGA ACTGTCATGT CTTCGGATGT GCGACTATCG GTCTAGGATC GCATCTCA-- ---------- ---------- ---------- ----------
G. ruber Ia            ATGACTGTGA ACTGTCATGT CTTCGGATGT GCGACTATTG GTCTAGGATC ACATGACTT- ---------- ---------- ---------- ----------
G. ruber Ib1           ATGACTGTGA ACTGTCATGT CTTCGGATGT ACGACTATCG GTCTAGGATC ACATGACTT- ---------- ---------- ---------- ----------
G. ruber Ib2           ATGACTGTGA ACTGTCATGT CTTCGGATGT ACGACTATCG GTCTAGGATC ACATGACTT- ---------- ---------- ---------- ----------
G. ruber IIa           GTGTGAACTA TAAGTCTTTT GACTTATGAC CATCACTCTA GACGTTTATG TTTTGGTGTT G--------- ---------- ---------- ----------
G. conglobatus         TGAACTGTAT CTCTTCGGAT ATGCGACTAT CACTCTTAGG CAACTATGAT CTGCTTGTA- ---------- ---------- ---------- ----------
G. rubescens (pink)    TTTATGGTTG ATCGGGCGTG AACCGTGTGT GCTTTAGGAC ATGCCGACCA TCGTCCAGTA CATTACTCTT ATAGTTGTTT ATTTG----- ----------
G. bulloides Ia        GCTCACGTTA --------GT AGGCTAGAG- ---------- ---------- ---------- ---------- ---------- ---------- ----------
G. bulloides Ib        TTCCCAAA-- ------GTGT AGGCTAGAG- ---------- ---------- ---------- ---------- ---------- ---------- ----------
G. bulloides IIa       TGGGCGAGGC TCT---GTGT AGGATAGAC- ---------- ---------- ---------- ---------- ---------- ---------- ----------
G. bulloides IIb       TGGGCGAGGC TCT---GTGT AGGATAGAC- ---------- ---------- ---------- ---------- ---------- ---------- ----------
G. bulloides IIc       TGGGCGAGGC TCT---GTGT AGGATAGAC- ---------- ---------- ---------- ---------- ---------- ---------- ----------
G. bulloides IId       TGGGCGAGGC TCT---GTGT AGGATAGAC- ---------- ---------- ---------- ---------- ---------- ---------- ----------
G. bulloides IIe       TGGGCGAGGC TCT---GTGT AGGATAGAC- ---------- ---------- ---------- ---------- ---------- ---------- ----------
T. quinqueloba Ia      TACAGTCAGA CCCGGTTGGG CATGTACGGA AAGGTCACAT ATAAAGTACG GTCTGCTAAA GATATGAATA ---------- ---------- ----------
T. quinqueloba Ib      CTTTTTTCGA GAATG----- ---------- ---------- ---------- ---------- ---------- ---------- ---------- ----------
T. quinqueloba IIa     GAATATTGTT GTCGTCGAGT CATAATTGCC ACTACGTCTG GTCCTTCGGG --CAGAATAG TCCTTATGCT TGTCT----- --ACACAAAA TTCTCCACAA
T. quinqueloba IIb     GAATATTGTT GTCGTCGAGT CATTATTGCC ACTACGTCTG GTCCTTTGGG GGCAGAATAG TGTTGTATAT TTTGTCTTGT CAACACAAAA TTCTTCACAA
T. quinqueloba IIc     GAATATTGTT GTCGTCGAGT CATTATTGCC ACTACGTCTG GTCCTTTGGG --CAGAATAG TGTTGTATAT TTTGTCTTGT CAACACAAAA TTCTTCACAA
T. quinqueloba IId     GAATATTGTT GTCGTCGAGT CATTATTGCC ACTACGTCTG GTCCTTTGGG --CAGAATAG TGTTTTATAT TTTGTCTTG- CAACACAAAA TTCTTCACAA
G. falconensis         ---------- ---------- ---------- ---------- ---------- ---------- ---------- ---------- ---------- ----------
H. pelagica            AATTTTCGCC ACGGGCCACC GTGGGAAACA AGTCACTTTG AGCGAT---- ---------- ---------- ---------- ---------- ----------
G. menardii            TCATGGAAAG CAGCCTACGT TGTTTC-ATA ---CGTGCAG C-TG------ -----TGTCG CCTCT-GAGT TATTGTCATT TGG------- ----------
G. ungulata            ACAGTTTCGT TTGTGCGAAC CTGTGTCGCC TCTGAGTTAT TGTCATTTGG ---------- ---------- ---------- ---------- ----------
G. hirsuta             CTGATAGACC CCTCGTAAGA ---------- ---------- -------GCG CGTTGTCTAT TGCGTTA-AT ATATGTAACA AGTGACACAT ACTG------
G. scitula             TCTTGGACCC CTCTTTTATT AAGA------ ---------- -------GCG AGTGTCTAAT GTCTTATTAT CCTTTATATA AGATAGTGTC GTATGCTG--
G. truncatulinoides    CTATTTGTAG TAAGCGCTCA TACTA----- ---------- ---------- ---------- ---------- ---------- ---------- ----------
N. pachyderma I        CCCTTA-ACT TAACGGTT-- ---------- ---------- -----AAGCG CGCGTCTTTA TTTAA---AG AGTTT--AAG -GCATTGCGC ATGCTG----
N. pachyderma II       CCCTTA-TTC ACTGCTTT-- --------AA TTAGTCTGTT T----AAGCG CGCGTCTTTA TTTAA---WG AGTTT--AAG -ACACTGCGC ATGCTG----
N. pachyderma III      CCCTTA-TAC TCGATTTCGC TTTAATTAGC GTTATTTGTT TAAT-AAGCG CGCGTCTTTA TTTAA---AG AGTTT--AAG GACACTGCGC ATGCTG----
N. pachyderma IV       CCCTTA-ACT CCAATATTCT TATTGTGTT- ---------- -----AAGCG CGTGTCTTTA TTTTA---AG AGTTTT-AAG -ACACTGCGC ATGCTG----
N. pachyderma V        CCCTTA-TTC AAG------C TTTTATTAG- ----TTTGTT T----AAGCG CGCGTCTTTA TTTAA---AG AGTTT--AAG -ACACTGCGC ATGCTG----
N. pachyderma VI       CCCTTA-TTC ACTGCTTTAA TTAGT----- ----TTTGTT T----AAGCG CGCGTCTTTA ATTAA---CG AGTTT--AAG -ACATTGCGC ATGCTG----
N. pachyderma VII      CCCTTA-ACC GAGTTAAATC GAGTT----- ---------- -----AAGCG CGCGTCTTTA TTTAA---AG AGTTT--AAG -ACATTGCGC ATGCTG----
N. dutertrei_C         CCCTTGTCTT CGAT------ ---------- ---------- -----AAGCG CGTGTCTTTT --------AG AGTTT--AA- -ACATTGCGC ATGCTG----
N. dutertrei_Ib        CCCTTGTCTT CGAT------ ---------- ---------- -----AAGCG CGTGTCTTTT --------AG AGTTT--AA- -ACATTGCGC ATGCTG----
P. obliquiloculata_BR  CCCTT--ATT CTTAT----- ---------- ---------- -----AAGCG CGTGTCTTT- -------ATG GGTTT--AA- -ACATTGCGC ATGCTG----
P. obliquiloculata_AS  CCCTT--ATT -TAAT----- ---------- ---------- -----AAGCG CGTGTCTTT- -------ATG GGTTT--AA- -ACATTGCGC ATGCTG----
G. inflata             CCCTAATAGG CTTAACTGTC TTT------- ---------- ------AGCG CGTGTCTC-A CG-----AGT TCTTT--AAA G-CACTGCGC ATGCTG----
G. crassaformis        CCCTAATAGG CTTAACTGTC TTT------- ---------- ------AGCG CGTGTCTCTA CG-----AGT TCTTT--AAA G-CACTGCGC ATGCTG----
N. incompta I          CCCTCACCTT TGAGT----- ---------- ---------- -------GCG CGTCCTAACT T-------GT AGTACATGGT G-TAAATGGA TTTGTT----
N. incompta II         CCCTCACCTT TGAGT----- ---------- ---------- -------GCG CGTCCTAGCT T-------AG AGTACATGGT G-TAAATGGA CTTGTT----
G. glutinata Ia1       TTCGCGAG-- TGCGTGTCTT TT-C--GCGC TCGCACGCTT TTTACA---- ---------- ---------- ---------- ---------- ----------
G. glutinata Ia2       TTCGAGAG-- TGTGTGTCTT TTTT--GCGC TCGCAC---- TTTACA---- ---------- ---------- ---------- ---------- ----------
G. glutinata Ia3       TTCGAGAG-- TGTGTGTCTT TTTTTTGCGC TCGCAC---- TTTACA---- ---------- ---------- ---------- ---------- ----------
C. nitida              TTCGGAAGCG TGTGTCTTTA CGCTCACACT TCA------- ---------- ---------- ---------- ---------- ---------- ----------
G. uvula               CTTGAGTCTC TCGCTTCGC- ---------- ---------- ---------- ---------- ---------- ---------- ---------- ----------
B. variabilis          -G-ATTTCAC -TAT-C-AAT -----GTGCG CGTCTTTCGC TTAGCTCACT GCGCT----- ---------- ---------- ---------- ----------
S. globigerus          -G-ATTTCAC -TAT-C-AAT -----GCGCG CGTCTTTCGC TTAGCTCACT GCGCT----- ---------- ---------- ---------- ----------
B. alata               TTCTCACGGA TATTGA---- -----GTGCG CGTCTTACGC TTAGCTCACT ATACT----- ---------- ---------- ---------- ----------
G. vivans              CGCGTCTTAG TTTGCTTAGC TCACACAA-- ---------- ---------- ---------- ---------- ---------- ---------- ----------
C. porrectus           TGTGCGTGTC TTTGTCTCGT TTAACTCATA CAA------- ---------- ---------- ---------- ---------- ---------- ----------
C. ovoidea             CCTCTTTTTA ATAAGAGTGT GCTTGCTGCG TGTCTTTGAC CAACGCTGTT TGCTTTTCAT GTGTT----- ---------- ---------- ----------
G. opercularis         TGAGGTCTGG CGAGTTGTGC GTCTTTTCAT CGTTATCACA TCATACAAC- ---------- ---------- ---------- ---------- ----------
E. aculeatum           ---------- ---------- ---------- ---------- ---------- ---------- ---------- ---------- ---------- ----------
E. vitrea              GAGCGCGTGT CTTAGTTTGC TTAGCTCACA CAA------- ---------- ---------- ---------- ---------- ---------- ----------
H. germanica           CATACACACA TATTATA--- ---------- ---------- ---------- ---------- ---------- ---------- ---------- ----------
P. mediterranensis     TGATTACGTA GCGTGTGTCT TTGATTACGT CTGGCTCGTG CGA------- ---------- ---------- ---------- ---------- ----------
S. fusiformis          TGCGCCTTAG CTTGTTTAGC TCACACAA-- ---------- ---------- ---------- ---------- ---------- ---------- ----------
V. fragilis            TGTGCGCGTG TCTTAGTTTG CTTAGCTCAC ACAA------ ---------- ---------- ---------- ---------- ---------- ----------
A. pseudocassis        CTTTGTTCAG TTTGCGCACA CTT------- ---------- ---------- ---------- ---------- ---------- ---------- ----------
Spiroplectammina sp.   ATATATATAG NGCGNGTCTC GGTTTGCTTT CACTCACACA A--------- ---------- ---------- ---------- ---------- ----------
Textularia sp.         TTACTAAAGT GCGTGTCTTA GTTTGCTTTT GCTCACACAA ---------- ---------- ---------- ---------- ---------- ----------
S. limosum             ATTAACCAAA TTTGAGATAA TGTATTGTTT TTTTAGTTA- ---------- ---------- ---------- ---------- ---------- ----------
G. antarctica          TGCGCGTGTC TTTGTCCGTT CGACACATAC AA-------- ---------- ---------- ---------- ---------- ---------- ----------
D. aphelis             AGAGAGCGCG TGTCTTAGTT TTCGCTTTGC TCATACAA-- ---------- ---------- ---------- ---------- ---------- ----------
P. peruviana           TTGTGAACTT GATTTAATAT ATAGCTTAC- ---------- ---------- ---------- ---------- ---------- ---------- ----------
M. secans              GGATTGTGAA CTCAAATATA TTTAATTTAT ATTA------ ---------- ---------- ---------- ---------- ---------- ----------
Quinqueloculina sp.    GATTGTGAAC CTTTTATATT ATTTGATAAT ATTA------ ---------- ---------- ---------- ---------- ---------- ----------
N. haylinosphaira      GTGGTGACTG CGTCCGGTAT GGTATACGCT GC-------- ---------- ---------- ---------- ---------- ---------- ----------
M. fusca               TCAAA----- ---------- ---------- ---------- ---------- ---------- ---------- ---------- ---------- ----------
T. alba                TCTTTGCATT TGAGTTGCTT TTAGTGAGTT TTAACGTTTT TAGTTTGTTT TACCCCAGTT TCGGCTGCGC GCGTAACTTT CTTTTTTAAA CTATTGCTTC
A. mexicana            TACGGGTAAG CGTGTTTGTC CTTATATTGT TCCAACATCA TACAAA---- ---------- ---------- ---------- ---------- ----------
A. triangularis        GTGTTATATA TATATATATA TATGANTTAA TTTTCGTATA TTATATTATA TAATTATGTG AATTTTTTGA GCTAAGGATT TGATTCAAAG TAAAAG----
A. rara                AATATATATT TAATCATAAT TTTATTATTA TGTGCCAATA TTATTTTATT TTTTTTTAAA GG-------- --TAAGGATT TGATTCAAAG TAAAAC----
E. scabrum             ACACACGCGC ATGTAA---- ---------- ---------- ---------- ---------- ---------- ---------- ---------- ----------
N. venosus             CGTGTCTTTG ATTGCTTAGC TCATACAA-- ---------- ---------- ---------- ---------- ---------- ---------- ----------
B. marginata           AGCGCGTGTC TTGGTTTGCT TAGCTCGCAC AATT------ ---------- ---------- ---------- ---------- ---------- ----------
```

```
Trochammina sp.         TG--GTTGGT GCGTGTCTTA GTTTACTTT- GCTCGCACAA ---------- ---------- ---------- ---------- ---------- ----------
Peneroplis sp.          TTATATATTA TTTATAAGGA TTTTAAGTGA ACATATTTTA TTATACATAT ATTATTATAT ATATTTATTA TA-------- ---------- ----------
S. orbiculus            TGAACATATT ATATATATTA TTAT------ ---------- ---------- ---------- ---------- ---------- ---------- ----------
Allogromia sp.          TTAACTTTGC ACCTTTATTG TTGCACGGTA TTCTTTTAA- ---------- ---------- ---------- ---------- ---------- ----------

                        501        511        521        531        541        551        561        571        581        591       600
                        |          |          |          |          |          |          |          |          |          |         |
G. siphonifera Ia1      ---------- ---------- ---------- ---------- ---------- ---------- ---------- ---------- ---------- ----------
G. siphonifera Ia2      ---------- ---------- ---------- ---------- ---------- ---------- ---------- ---------- ---------- ----------
G. siphonifera IIa1     ---------- ---------- ---------- ---------- ---------- ---------- ---------- ---------- ---------- ----------
G. siphonifera IIa2     ---------- ---------- ---------- ---------- ---------- ---------- ---------- ---------- ---------- ----------
G. siphonifera IIa3     ---------- ---------- ---------- ---------- ---------- ---------- ---------- ---------- ---------- ----------
G. siphonifera IIa      ---------- ---------- ---------- ---------- ---------- ---------- ---------- ---------- ---------- ----------
G. siphonifera IIb      ---------- ---------- ---------- ---------- ---------- ---------- ---------- ---------- ---------- ----------
G. calida               ---------- ---------- ---------- ---------- ---------- ---------- ---------- ---------- ---------- ----------
O. universa I           ---------- ---------- ---------- ---------- ---------- ---------- ---------- ---------- ---------- ----------
O. universa III         ---------- ---------- ---------- ---------- ---------- ---------- ---------- ---------- ---------- ----------
G. sacculifer           ---------- ---------- ---------- ---------- ---------- ---------- ---------- ---------- ---------- ----------
G. ruber pink           ---------- ---------- ---------- ---------- ---------- ---------- ---------- ---------- ---------- ----------
G. ruber Ia             ---------- ---------- ---------- ---------- ---------- ---------- ---------- ---------- ---------- ----------
G. ruber Ib1            ---------- ---------- ---------- ---------- ---------- ---------- ---------- ---------- ---------- ----------
G. ruber Ib2            ---------- ---------- ---------- ---------- ---------- ---------- ---------- ---------- ---------- ----------
G. ruber IIa            ---------- ---------- ---------- ---------- ---------- ---------- ---------- ---------- ---------- ----------
G. conglobatus          ---------- ---------- ---------- ---------- ---------- ---------- ---------- ---------- ---------- ----------
G. rubescens (pink)     ---------- ---------- ---------- ---------- ---------- ---------- ---------- ---------- ---------- ----------
G. bulloides Ia         ---------- ---------- ---------- ---------- ---------- ---------- ---------- ---------- ---------- ----------
G. bulloides Ib         ---------- ---------- ---------- ---------- ---------- ---------- ---------- ---------- ---------- ----------
G. bulloides IIa        ---------- ---------- ---------- ---------- ---------- ---------- ---------- ---------- ---------- ----------
G. bulloides IIb        ---------- ---------- ---------- ---------- ---------- ---------- ---------- ---------- ---------- ----------
G. bulloides IIc        ---------- ---------- ---------- ---------- ---------- ---------- ---------- ---------- ---------- ----------
G. bulloides IId        ---------- ---------- ---------- ---------- ---------- ---------- ---------- ---------- ---------- ----------
G. bulloides IIe        ---------- ---------- ---------- ---------- ---------- ---------- ---------- ---------- ---------- ----------
T. quinqueloba Ia       ---------- ---------- ---------- ---------- ---------- ---------- ---------- ---------- ---------- ----------
T. quinqueloba Ib       ---------- ---------- ---------- ---------- ---------- ---------- ---------- ---------- ---------- ----------
T. quinqueloba IIa      TGTTGTAGCC ATACTTGATT GTATGCGC-- --TATTAATG TCTATGTGT- -----GACAC ATTCGTGGTT CAAGACCAGT TCGGCTGCTT GCACACATTG
T. quinqueloba IIb      TATTGTAGCC GTACTTGATT GTATGCGC-- --TATTCATG TATATGTG-G TTAGT----- ---------- CAACACCAGT TCGGCTGCTT GCACACATCG
T. quinqueloba IIc      TATTGTAGCC GTACTTGATT GTATGCGC-- --TATTCATG TATATGTG-G TTAGT----- ---------- CAACACCAGT TCGGCTGCTT GCACACATCG
T. quinqueloba IId      TATTGTAGCC GTACTTCATT GTATGCGC-- --TATTCATG TATATGT--- -----GACAC ATTTGTGGTT CAAGACCAGT TCGGCTGCTT GTACACATAT
G. falconensis          ---------- ---------- ---------- ---------- ---------- ---------- ---------- ---------- ---------- ----------
H. pelagica             ---------- ---------- ---------- ---------- ---------- ---------- ---------- ---------- ---------- ----------
G. menardii             ---------- ---------- ---------- ---------- ---------- ---------- ---------- ---------- ---------- ----------
G. ungulata             ---------- ---------- ---------- ---------- ---------- ---------- ---------- ---------- ---------- ----------
G. hirsuta              ---------- ---------- ---------- ---------- ---------- ---------- ---------- ---------- ---------- ----------
G. scitula              ---------- ---------- ---------- ---------- ---------- ---------- ---------- ---------- ---------- ----------
G. truncatulinoides     ---------- ---------- ---------- ---------- ---------- ---------- ---------- ---------- ---------- ----------
N. pachyderma I         ---------- ---------- ---------- ---------- ---------- ---------- ---------- ---------- ---------- ----------
N. pachyderma II        ---------- ---------- ---------- ---------- ---------- ---------- ---------- ---------- ---------- ----------
N. pachyderma III       ---------- ---------- ---------- ---------- ---------- ---------- ---------- ---------- ---------- ----------
N. pachyderma IV        ---------- ---------- ---------- ---------- ---------- ---------- ---------- ---------- ---------- ----------
N. pachyderma V         ---------- ---------- ---------- ---------- ---------- ---------- ---------- ---------- ---------- ----------
N. pachyderma VI        ---------- ---------- ---------- ---------- ---------- ---------- ---------- ---------- ---------- ----------
N. pachyderma VII       ---------- ---------- ---------- ---------- ---------- ---------- ---------- ---------- ---------- ----------
N. dutertrei_C          ---------- ---------- ---------- ---------- ---------- ---------- ---------- ---------- ---------- ----------
N. dutertrei_Ib         ---------- ---------- ---------- ---------- ---------- ---------- ---------- ---------- ---------- ----------
P. obliquiloculata_BR   ---------- ---------- ---------- ---------- ---------- ---------- ---------- ---------- ---------- ----------
P. obliquiloculata_AS   ---------- ---------- ---------- ---------- ---------- ---------- ---------- ---------- ---------- ----------
G. inflata              ---------- ---------- ---------- ---------- ---------- ---------- ---------- ---------- ---------- ----------
G. crassaformis         ---------- ---------- ---------- ---------- ---------- ---------- ---------- ---------- ---------- ----------
N. incompta I           ---------- ---------- ---------- ---------- ---------- ---------- ---------- ---------- ---------- ----------
N. incompta II          ---------- ---------- ---------- ---------- ---------- ---------- ---------- ---------- ---------- ----------
G. glutinata Ia1        ---------- ---------- ---------- ---------- ---------- ---------- ---------- ---------- ---------- ----------
G. glutinata Ia2        ---------- ---------- ---------- ---------- ---------- ---------- ---------- ---------- ---------- ----------
G. glutinata Ia3        ---------- ---------- ---------- ---------- ---------- ---------- ---------- ---------- ---------- ----------
C. nitida               ---------- ---------- ---------- ---------- ---------- ---------- ---------- ---------- ---------- ----------
G. uvula                ---------- ---------- ---------- ---------- ---------- ---------- ---------- ---------- ---------- ----------
B. variabilis           ---------- ---------- ---------- ---------- ---------- ---------- ---------- ---------- ---------- ----------
S. globigerus           ---------- ---------- ---------- ---------- ---------- ---------- ---------- ---------- ---------- ----------
B. alata                ---------- ---------- ---------- ---------- ---------- ---------- ---------- ---------- ---------- ----------
G. vivans               ---------- ---------- ---------- ---------- ---------- ---------- ---------- ---------- ---------- ----------
C. porrectus            ---------- ---------- ---------- ---------- ---------- ---------- ---------- ---------- ---------- ----------
C. ovoidea              ---------- ---------- ---------- ---------- ---------- ---------- ---------- ---------- ---------- ----------
G. opercularis          ---------- ---------- ---------- ---------- ---------- ---------- ---------- ---------- ---------- ----------
E. aculeatum            ---------- ---------- ---------- ---------- ---------- ---------- ---------- ---------- ---------- ----------
E. vitrea               ---------- ---------- ---------- ---------- ---------- ---------- ---------- ---------- ---------- ----------
H. germanica            ---------- ---------- ---------- ---------- ---------- ---------- ---------- ---------- ---------- ----------
P. mediterranensis      ---------- ---------- ---------- ---------- ---------- ---------- ---------- ---------- ---------- ----------
S. fusiformis           ---------- ---------- ---------- ---------- ---------- ---------- ---------- ---------- ---------- ----------
V. fragilis             ---------- ---------- ---------- ---------- ---------- ---------- ---------- ---------- ---------- ----------
A. pseudocassis         ---------- ---------- ---------- ---------- ---------- ---------- ---------- ---------- ---------- ----------
Spiroplectammina sp.    ---------- ---------- ---------- ---------- ---------- ---------- ---------- ---------- ---------- ----------
Textularia sp.          ---------- ---------- ---------- ---------- ---------- ---------- ---------- ---------- ---------- ----------
S. limosum              ---------- ---------- ---------- ---------- ---------- ---------- ---------- ---------- ---------- ----------
G. antarctica           ---------- ---------- ---------- ---------- ---------- ---------- ---------- ---------- ---------- ----------
D. aphelis              ---------- ---------- ---------- ---------- ---------- ---------- ---------- ---------- ---------- ----------
P. peruviana            ---------- ---------- ---------- ---------- ---------- ---------- ---------- ---------- ---------- ----------
M. secans               ---------- ---------- ---------- ---------- ---------- ---------- ---------- ---------- ---------- ----------
Quinqueloculina sp.     ---------- ---------- ---------- ---------- ---------- ---------- ---------- ---------- ---------- ----------
N. haylinosphaira       ---------- ---------- ---------- ---------- ---------- ---------- ---------- ---------- ---------- ----------
M. fusca                ---------- ---------- ---------- ---------- ---------- ---------- ---------- ---------- ---------- ----------
T. alba                 AAGTTTTAAT TTATTTGTGA GGGGCTCATG GTGAAATTCT GCTATTTATT GGGAGAGGAT TAGTGCACA- ---------- ---------- ----------
A. mexicana             ---------- ---------- ---------- ---------- ---------- ---------- ---------- ---------- ---------- ----------
A. triangularis         ---------- ---------- ---------- ---------- ---------- ---------- ---------- ---------- ---------- ----------
A. rara                 ---------- ---------- ---------- ---------- ---------- ---------- ---------- ---------- ---------- ----------
E. scabrum              ---------- ---------- ---------- ---------- ---------- ---------- ---------- ---------- ---------- ----------
N. venosus              ---------- ---------- ---------- ---------- ---------- ---------- ---------- ---------- ---------- ----------
B. marginata            ---------- ---------- ---------- ---------- ---------- ---------- ---------- ---------- ---------- ----------
Trochammina sp.         ---------- ---------- ---------- ---------- ---------- ---------- ---------- ---------- ---------- ----------
```

```
Peneroplis sp.           ---------- ---------- ---------- ---------- ---------- ---------- ---------- ---------- ---------- ----------
S. orbiculus             ---------- ---------- ---------- ---------- ---------- ---------- ---------- ---------- ---------- ----------
Allogromia sp.           ---------- ---------- ---------- ---------- ---------- ---------- ---------- ---------- ---------- ----------

                         601        611        621        631        641        651        661        671        681        691       700
                         |          |          |          |          |          |          |          |          |          |         |
G. siphonifera Ia1       ---------- ---------- ---------- ---------- ---------- -------GAC TCA--ATTGA AC-----GCA ACGGA-CGTG ATTGCAA---
G. siphonifera Ia2       ---------- ---------- ---------- ---------- ---------- -------GAC TCA--ATTGA AC-----GCA ACGGA-CGTG ATTGCAA---
G. siphonifera IIa1      ---------- ---------- ---------- ---------- ---------- -------GGC TCA--ATTGA AC-----GCA ACGGA-CGTG ATCGCGA---
G. siphonifera IIa2      ---------- ---------- ---------- ---------- ---------- -------GGC TCA--ATTGA AC-----GCA ACGGA-CGTG ATCGCGA---
G. siphonifera IIa3      ---------- ---------- ---------- ---------- ---------- -------GGC TCA--ATTGA AC-----GCA ACGGA-CGTG ATCGCGA---
G. siphonifera IIa       ---------- ---------- ---------- ---------- ---------- -------GGC TCA--ATTGA AC-----GCA ACGGA-CGTG ATCGCGA---
G. siphonifera IIb       ---------- ---------- ---------- ---------- ---------- -------GGC TCA--ATTGA AC-----GCA ACGGA-CGTG ATCGCAA---
G. calida                ---------- ---------- ---------- ---------- ---------- -------GGC TCA--ATTGA AC-----GCA ACGGA-CGTG ATTGCAA---
O. universa I            ---------- ---------- ---------- ---------- ---------- -------GAT TCT--TTCCA AT-----GCA ACGAG-CGTG ATCGCGA---
O. universa III          ---------- ---------- ---------- ---------- ---------- -------GTC TCT--TTTGA AC-----GCA ACGGA-CGTG ATCGCAA---
G. sacculifer            ---------- ---------- ---------- ---------- ---------- -------GTT CTA--TTGGA AT-----GCA ACGGA-CGTG ATTGCAA---
G. ruber pink            ---------- ---------- ---------- ---------- --------GC CCCTGG-GAC TCT--TTTGA AC-----GCA ACGGA-CGTG ATTGCAA---
G. ruber Ia              ---------- ---------- ---------- ---------- --------GC CCCTGG-GAC TCT--TTTGA AC-----GCA ACGGA-CGTG ATTGCAA---
G. ruber Ib1             ---------- ---------- ---------- ---------- --------GC CCCTGG-GAC TCT--TTTGA AC-----GCA ACGGA-CGTG ATTGCAA---
G. ruber Ib2             ---------- ---------- ---------- ---------- --------GC CCCTGG-GAC TCT--TTTGA AC-----GCA ACGGA-CGTG ATTGCAA---
G. ruber IIa             ---------- ---------- ---------- ---------- --------GC CCTTGA-TGC TCA--TTTGA AC-----GCA ACGGA-CGTG ATTGCAA---
G. conglobatus           ---------- ---------- ---------- ---------- --------GC CCCTGG-TAC TCT--TTTGA AC-----GCA ACGGA-CGTG ATTGCAA---
G. rubescens (pink)      ---------- ---------- ---------- ---------- --------GC CCCTGG---C TTT--TTTGA AC-----GCA ACGGA-CGTG ACCTCAA---
G. bulloides Ia          ---------- ---------- ---------- ---------- ---------- -------ATT TGA--ACAGT AC-----GCA ACGGA-CGCG ATCGTAA---
G. bulloides Ib          ---------- ---------- ---------- ---------- ---------- -------ACT TGA--ACAGT AC-----GCA ACGGA-CGTG ATCGTAA---
G. bulloides IIa         ---------- ---------- ---------- ---------- ---------- -------CTC TGA--ACAGT AC-----GCA ACGAA-CGCG ATCGTAA---
G. bulloides IIb         ---------- ---------- ---------- ---------- ---------- -------CTC TGA--ACAGT AC-----GCA ACGAA-CGCG ATCGTAA---
G. bulloides IIc         ---------- ---------- ---------- ---------- ---------- -------CTC TGA--ACAGT AC-----GCA ACGAA-CGCG ATCGTAA---
G. bulloides IId         ---------- ---------- ---------- ---------- ---------- -------CTC TGA--ACAGT AC-----GCA ACGAA-CGCG ATCGTAA---
G. bulloides IIe         ---------- ---------- ---------- ---------- ---------- -------CTC TGA--ACAGT AC-----GCA ACGAA-CGCG ATCGTAA---
T. quinqueloba Ia        ---------- ---------- ---------- ---------- TCTTGCCCTA AAGATTGATT AACACCGTGA GT-----GCA ACGAG-TGAG ATTGCGA---
T. quinqueloba Ib        ---------- ---------- ---------- ---------- ACTTGCCCTA AAGATTGATT AACACCGTGA GT-----GCA ACGAG-TGAG ATTGCGA---
T. quinqueloba IIa       CCATCTCAAC ATGATTGGTC AGGCTGCTAG AGACCTTGTG TCTTGCCCCA AAGGTTGATT AACACCGTGA GT-----GCA ACGAG-TGAG ATTGCAA---
T. quinqueloba IIb       CCATCTCAAC ATGATCAGTC AGGCTGCTAG AGACCTTGTG TCTTGCCCCA AAGGTTGATT AACACCGTGA GT-----GCA ACGAG-TGAG ATTGCAA---
T. quinqueloba IIc       CCATCTCAAC ATGATCAGTC AGGCTGCTAG AGACCTTGTG TCTTGCCCCA AAGGTTGATT AACACCGTGA GT-----GCA ACGAG-TGAG ATTGCAA---
T. quinqueloba IId       ACACTCGA-C ATGATCATTC AGGCTGCTAG AGACCTTGTG TCTTGCCCCA AAGGTTGATT AACACCGTGA GT-----GCA ACGAG-TGAG ATTGCAA---
G. falconensis           ---------- ---------- ---------- ---------- ---------- -------CTT TTG--TGTGA AC-----GCA ACGGA-CGTG ATTGCAA---
H. pelagica              ---------- ---------- ---------- ---------- ---------- -------CCT CAA--TTTGA AA-----GCA ACGAA-CGTG ACCGCAG---
G. menardii              ---------- ---------- ---------- ---------- ---------- -------AGA GTA--CGTGA AT-----GCA ACGAA-CGTG ACCGTAG---
G. ungulata              ---------- ---------- ---------- ---------- ---------- -------AGA GTA--CGTGA AG-----GCA ACGAA-CGTG ACCGTAG---
G. hirsuta               ---------- ---------- ---------- ---------- ---------- -------TTG GGA--TCTGA AA-----GCA ACGAA-CGTG ACCGCAA---
G. scitula               ---------- ---------- ---------- ---------- ---------- -------TTG GGA--TCTGA AA-----GCA ACGAA-CGTG ACCGCAA---
G. truncatulinoides      ---------- ---------- ---------- ---------- ---------- -------TTG GGA--CCGGA AT-----GCA ACGAG-CGCG ACTGCAC---
N. pachyderma I          ---------- ---------- ---------- ---------- ---------- -------TTG GGC--TCTGA AA-----GCA ACGAA-CGTG ACCGCAA---
N. pachyderma II         ---------- ---------- ---------- ---------- ---------- -------TTG GGC--TCTGA AA-----GCA ACGAA-CGTG ACCGCAA---
N. pachyderma III        ---------- ---------- ---------- ---------- ---------- -------TTG GGC--TCTGA AA-----GCA ACGAA-CGTG ACCGCAA---
N. pachyderma IV         ---------- ---------- ---------- ---------- ---------- -------TTG GGC--TCTGA AA-----GCA ACGAA-CGTG ACCGCAA---
N. pachyderma V          ---------- ---------- ---------- ---------- ---------- -------TTG GGC--TCTGA AA-----GCA ACGAA-CGTG ACCGCAA---
N. pachyderma VI         ---------- ---------- ---------- ---------- ---------- -------TTG GGC--TCTGA AA-----GCA ACGAA-CGTG ACCGCAA---
N. pachyderma VII        ---------- ---------- ---------- ---------- ---------- -------TTG GGC--TCTGA AA-----GCA ACGAA-CGTG ACCGCAA---
N. dutertrei_C           ---------- ---------- ---------- ---------- ---------- -------TTG GGT--CCTGA AA-----GCA ACGAA-CGTG ACCGCAA---
N. dutertrei_Ib          ---------- ---------- ---------- ---------- ---------- -------TTG GGT--CCTGA AA-----GCA ACGAA-CGTG ACCGCAA---
P. obliquiloculata_BR    ---------- ---------- ---------- ---------- ---------- -------TTG GGT--CCTGA AA-----GCA ACGAA-CGTG ACCGCAA---
P. obliquiloculata_AS    ---------- ---------- ---------- ---------- ---------- -------TTG GGT--CCTGA AA-----GCA ACGAA-CGTG ACCGCAA---
G. inflata               ---------- ---------- ---------- ---------- ---------- -------TTG GGT--CCTGA AA-----GCA ACGAA-CGTG ACCGCAA---
G. crassaformis          ---------- ---------- ---------- ---------- ---------- -------TTG GGT--CCTGA AA-----GCA ACGAA-CGTG ACCGCAA---
N. incompta I            ---------- ---------- ---------- ---------- ---------- -------TTG GGTACCCAGA AA-----GCA ACGAA-CGTG ACCGCAA---
N. incompta II           ---------- ---------- ---------- ---------- ---------- -------TTG GGTACCCAGA AA-----GCA ACGAA-CGTG ACCGCAA---
G. glutinata Ia1         ---------- ---------- ---------- ---------- ---------- -------TTA GGT--CCTGA AA-----GCA ACGAA-CGTG ACCGCAA---
G. glutinata Ia2         ---------- ---------- ---------- ---------- ---------- -------TTA GGT--CCTGA AA-----GCA ACGAA-CGTG ACCGCAA---
G. glutinata Ia3         ---------- ---------- ---------- ---------- ---------- -------TTA GGT--CCTGA AA-----GCA ACGAA-CGTG ACCGCAA---
C. nitida                ---------- ---------- ---------- ---------- ---------- -------TTA GGT--CCTGA AA-----GCA ACGAA-CGTG ACCGCAA---
G. uvula                 ---------- ---------- ---------- ---------- ---------- -------TAT CCT--TTCGA AT-----GCA ACGAA-CGTG ACCGCAA---
B. variabilis            ---------- ---------- ---------- ---------- ---------- -------TTA GA--TCCTGA AA-----GCA ACGAA-CGTG ACCGCAA---
S. globigerus            ---------- ---------- ---------- ---------- ---------- -------TTA GA--TCCTGA AA-----GCA ACGAA-CGTG ACCGCAA---
B. alata                 ---------- ---------- ---------- ---------- ---------- -------TTA GA--TCCTGA AA-----GCA ACGAA-CGTG ACCGCAA---
G. vivans                ---------- ---------- ---------- ---------- ---------- -------TTA GGT--CCTGA AA-----GCA ACGAA-CGTG ACCGCAA---
C. porrectus             ---------- ---------- ---------- ---------- ---------- -------TTA AGC--CCTGA AA-----GCA ACGAA-CGTG ACCGCAA---
C. ovoidea               ---------- ---------- ---------- ---------- ---------- -------TTA GGT--CCTGA AA-----GCA ACGAA-CGTG ACCGCAA---
G. opercularis           ---------- ---------- ---------- ---------- ---------- -------GTT GAT--TCTGA AA-----GCA ACGAA-CGTG ACCGCAA---
E. aculeatum             ---------- ---------- ---------- ---------- ---------- -------GTA CAC--TTTGA AA-----GCA ACGAA-CGTG ACCGTAT---
E. vitrea                ---------- ---------- ---------- ---------- ---------- -------TTA GGT--CCTGA AA-----GCA ACGAA-CGTG ACCGCAA---
H. germanica             ---------- ---------- ---------- ---------- ---------- -------TTG TGC--TTTGA AA-----GCA ACGAA-CGTG ACCGCAA---
P. mediterranensis       ---------- ---------- ---------- ---------- ---------- -------TTA GAA--CCTGA AA-----GCA ACGAA-CGTG ACCGCAA---
S. fusiformis            ---------- ---------- ---------- ---------- ---------- -------TTA GGT--CCTGA AA-----GCA ACGAA-CGTG ACCGCAA---
V. fragilis              ---------- ---------- ---------- ---------- ---------- -------TTA GAT--CCTGA AA-----GCA ACGAA-CGTG ACCGCAA---
A. pseudocassis          ---------- ---------- ---------- ---------- ---------- -------CCG A--GTCCTCA TT-----GCA ACGAA-CGTG ACCGCAA---
Spiroplectammina sp.     ---------- ---------- ---------- ---------- ---------- -------TTA AGT--CCTGA AA-----GCA ACGAA-CGTG ACCGCAA---
Textularia sp.           ---------- ---------- ---------- ---------- ---------- -------TTA G--GTCCTGA AA-----GCA ACGAA-CGTG ACCGCAA---
S. limosum               ---------- ---------- ---------- ---------- ---------- -------AGG GGG--CCGGA AG-----GCA ACGAA-CGTG ACCGCAA---
G. antarctica            ---------- ---------- ---------- ---------- ---------- -------TTA AGC--CCTGA AA-----GCA ACGAA-CGTG ACCGCAA---
D. aphelis               ---------- ---------- ---------- ---------- ---------- -------TTA GGT--CCTGA AA-----GCA ACGAA-CGTG ACCGCAA---
P. peruviana             ---------- ---------- ---------- ---------- ---------- -------TAT ATAAATGTGA AT-----GCA ACGAA-CGTG ACTATAA---
M. secans                ---------- ---------- ---------- ---------- ---------- -------TTA A--TAATTGA AT-----GCA ACGAA-CGTG ACTATAA---
Quinqueloculina sp.      ---------- ---------- ---------- ---------- ---------- -------TAT T--TAAATGA AT-----GCA ACGAA-CGTG ACTATAA---
N. haylinosphaira        ---------- ---------- ---------- ---------- ---------- -------GAT C--GCCACGA AG-----GCA ACGAA-CGTG ACCGCAG---
M. fusca                 ---------- ---------- ---------- ---------- ---------- -------TAC AAT--TCTGA AA-----GCA ACGAA-CGTG ACCGCAA---
T. alba                  ---------- ---------- ---------- ---------- ---------- -------TTG A--GTCCTGA AA-----GCA ACGAA-CGTG ACCGCAT---
A. mexicana              ---------- ---------- ---------- ---------- ---------- -------TTG GG--TCCTGA AA-----GCA ACGAA-CGTG ACCGCAA---
A. triangularis          ---------- ---------- ---------- ---------- ---------- -------CTG G--AGCCTGA AG-----GCA ACGAA-CGTG ACCGCAA---
A. rara                  ---------- ---------- ---------- ---------- ---------- -------GTG G--AGCCTGA AG-----GCA ACGAA-CGTG ACCGCAA---
E. scabrum               ---------- ---------- ---------- ---------- ---------- -------TTA AGT--CCTGA AA-----GCA ACGAA-CGTG ACCGCAA---
N. venosus               ---------- ---------- ---------- ---------- ---------- -------TTA GGT--CCTGA AA-----GCA ACGAA-CGTG ACCGCAA---
B. marginata             ---------- ---------- ---------- ---------- ---------- -------AGG T----CCTGA AA-----GCA ACGAA-CGTG ACCGCAA---
Trochammina sp.          ---------- ---------- ---------- ---------- ---------- -------TTA AGT--CCTGA AA-----GCA ACGAA-CGTG ACCGCAA---
Peneroplis sp.           ---------- ---------- ---------- ---------- ---------- -------TAT T--AAAATGA AT-----GCA ACGAA-CGTG ACCGTAA---
```

```
S. orbiculus            ---------- ---------- ---------- ---------- ---------- -------ATA ATA--AATGA AT-----GCA ACGAA-CGTG ACCGTAA---
Allogromia sp.          ---------- ---------- ---------- ---------- ---------- -------ATA TAC--TCTGA AG-----GCA ACGAA-CGTG ACCGCAA---

                        701       711       721       731       741       751       761       771       781       791      800
                        |         |         |         |         |         |         |         |         |         |        |
G. siphonifera Ia1      -----GTC-C TTGT--TG-- AACA-AATT- A-TATATAT- -A-CTACTTC ATCATTA--- -GTATATATA G--TTC---G CTTCTC---- ATGTCGTAG-
G. siphonifera Ia2      -----GTC-C TTGT--TG-- AACA-AATT- A-TATATAT- -A-CTACTTC ATCATTA--- -GTATATATA G--TTC---G CTTCTC---- ATGTCGTAG-
G. siphonifera IIa1     -----GTC-C TTGT--TG-- AACT-TCAG- --TATATAT- TGACTTTCCC CGTAGGAAGG GATTGGCAAT AATTGAATGT TCG--CTTTC TATTAGGTTT
G. siphonifera IIa2     -----GTC-C TTGT--TG-- AACT-TCAG- --TATATAT- TGACTATCCC CGTAGGAAGG GATTGGCAAT AATTGAATGT TCG--CTTTC TATTAGGTGT
G. siphonifera IIa3     -----GTC-C TTGT--TG-- AACT-TCAG- --TATATAT- TGACTATCCC CGTAGGAAGG GATTGGCAAT AATTGAATGT TCG--CTTTC TATTAGGTGT
G. siphonifera IIa      -----GTC-C TTGT--TG-- AACT-TCAG- --TATATAT- TGACTTTCCC CGTAGGAAGG GATTGGCAAT AATTGAATGT TCG--CTTTC TAT-AGGTGT
G. siphonifera IIb      -----GTC-C TTGT--TG-- AACT-TCAG- --TATATAT- TGACTTTCCC CGTAGGAAGG GATTGCCAAT AATTGAATGT TCG--CTTTC TATT-GGTGT
G. calida               -----GTC-C TTGT--TG-- AACT-TCTA- --TACATAT- CATTTACATT CTTAACTGAG TGTATCTAAG ATAATAGAGT TCG--CTTTC TATT-GGTGA
O. universa I           -----CCC-C TTGT--TG-- AGCT-TATTA CACATAAGCT CTATGTCGAC GGAACGCTTT TGCGTTACGG A--------- ---------- ----------
O. universa III         -----CCC-T TTGT--TG-- AGTGATGTCA GCACTCTACG TCTATCCTAG CTCGTTGATC GCAAGTGAGA GTTGGGTG-- ---------- ----------
G. sacculifer           -----GCT-T TTGTTTTG-- AGTT-GCGG- --TTTTACTG AAAGACTCTA CAATCCGCAA ACTTCAATGA TCTGAAGTAG ---AAAACCT AC-----ATG
G. ruber pink           -----CCC-C TTGT--TG-- AGAT-TAGAG G--------- AGTCTCGATA TTCATTGGCC --AAACTTAC CGCTTCGAGC TTG--CTCGT GCAGTTTT-G
G. ruber Ia             -----CCC-C TTGT--TG-- AGAT-TAGAG AT-------- AGTCTCGATA TTCACTGGAC --AAACTGGC TGCTTTATGC AGT--CTTGT TGAAT-----
G. ruber Ib1            -----CCC-C TTGT--TG-- AGAT-TAGAG AT-------- AGTCTCGATA TTCACTGGCC --AAACTGGC TGCTTTATGC AGT--CTTGT TGAAT-----
G. ruber Ib2            -----CCC-C TTGT--TG-- AGAT-TAGAG AT-------- AGTCTCGATA TTCACTGGCC --AAACTGGC TGCTTTATGC AGT--CTTGT TGAAT-----
G. ruber IIa            -----CCC-C TTGT--TG-- AGATTTATCC GATCTCTACG TTCACTGAAC CACACTCTTT AGCGAGAGCA GTGGGGA--- ---------- ----------
G. conglobatus          -----CCC-C TTGT--TG-- AGTTTGCTTC TCTTAGGAAC TCTACGTCTA CTGATCCACC TACCTTAGCG GGTAGGTGGT GA-------- ----------
G. rubescens (pink)     -----CCC-T TAGT--TG-- AGATGCTCTT GAACCAAGCT TCTCTACGTC TGCTAGACTG CTCGACTTCG GTTGGGTGGA AA-------- ----------
G. bulloides Ia         -----TCT-C TTGT--TA-- AGTGGCCATC CTGTGAGC-- CCCTGATTTA ATGG------ -CAGG----- CGGTATCATC TCAGCCACAT T----TCCTC
G. bulloides Ib         -----TCT-C TTGT--TA-- AATGGTTATC CTGTGAGC-- CAACCGTGAC TGATTA---- -CAGCACACC TGGTATCATC -CAGACCATA T-----TGGT
G. bulloides IIa        -----TCC-C TTGT--TG-- AGTGGCCATC CTGTAAGC-- TGCTGGATTA GGAACC---- -CAG------ TGGTATTATC TCAGCCACAG A----TTTTC
G. bulloides IIb        -----TCC-C TTGT--TG-- AGTGGCCATC CTGTAAGC-- TGCTGGATTA GGAACC---- -CAG------ TGGTATTATC TCAGCCACAG A----TTTTC
G. bulloides IIc        -----TCC-C TTGT--TG-- AGTGGCCATC CTGTAAGC-- TGCTGGATTT GAAACC---- -CAG------ TGGTATTATC TCAGCCACAG A----TTTTC
G. bulloides IId        -----TCC-C TTGT--TG-- AGTGGCCATC CTGTAAGC-- TGCTGGATTA GGAACC---- -CAG------ TGGTATTATC TCAGCCACAG A----TTTTC
G. bulloides IIe        -----TCC-C TTGT--TG-- AGTGGCCATC CTGTAAGC-- C-CTGGAGTC GTC------- -CAG------ TGGTATTATC TCAGCCACAG A----TCTTC
T. quinqueloba Ia       -----GTC-T TTGT--TA-- TGTAGTGCTC AACATA--CC TA-------- ---------- TGTGCAGT-- -------AGG ATTT-AACTA CACC--ATGT
T. quinqueloba Ib       -----GTC-T TTGT--TA-- TGTAGTGCTC AACATA--CC TA-------- ---------- TGTGCAGT-- -------AGG ATTT-AACTA CACC--ATGT
T. quinqueloba IIa      -----GTC-T TTGT--TA-- TGTAGTGAAC AACATATACC TACTACT-C- ---------- --TAGTAGT- -------AGG ATTCCAACTA CACA--GTAT
T. quinqueloba IIb      -----GCC-T TTGT--TA-- TGTAGTGAAC AACATATACC TACTACTACT ACTA---TTT TGTAGTAATG T------AGG ATTCCAACTA CACA--GAAT
T. quinqueloba IIc      -----GCC-T TTGT--TA-- TGTAGTGAAC AACATATACC TACTACTACT ACTT------ TGTAGTAATG T------AGG ATTCCAACTA CACACAGAAT
T. quinqueloba IId      -----GCC-T TTGT--TA-- TGTAGTGAAC AACATATACC TACTACTACT ACTACTACTT TGTAGTAATG T------AGG ATTCCAACTA CACA--GAAT
G. falconensis          -----CCC-C TTGT--TG-- ATTGGCCATC ACGTAAGCTC ACTTGTAATT TATTACAGTG TGGTATTATC TTAGCCATTC CCTTTTAGTT GTGGCGTGAT
H. pelagica             -----CCC-T TTGT--TG-- TGTCCTTGCC AGAGACACGC GTCTTTGTTG GTTAATTCCT CACACAACGA CGGCATTGTA CCTCTGTATG GCCGTGTCTT
G. menardii             -----TGT-T TTAGTTTG-- CATTTCAAAC AAGTGACATT CATTTGACAC GGTTTGTATT CCGGCTACGT ATCATCCCAG TGCATAACCA TAACCA----
G. ungulata             -----TGT-T TTAG--TT-- TGGATTTCAG ACAAGTGTTA TTAACTTGAC ACGGTCTGAG TTGGTTCATG TGTACGCGCG CAACACACAG GATGTGTCCC
G. hirsuta              -----CGT-C TTGT--TG-- CCTTTCTAAT TTATTCTAAT TCTTTTGTAA TTAGTCTAAT AACAAAGGCT ATTC------ ---------- ----------
G. scitula              -----CGT-C TTGT--TG-- CCTTACCTAT ACTGGTATTA TACTT-AATA CCGTATAACT CAAGGCTTTA C--------- ---------- ----------
G. truncatulinoides     -----CAC-C TAAT--TG-- CCCCTTATCT ACCATTAATT ACTT--TATT AACCGCCCTG TACGGGCACC GGGGCCTTAC CTTTA----- ----------
N. pachyderma I         -----CGT-C TTGT--TG-- CCTTAATTAA GTCGTGTTTA AATGGTATTT GATTACA--- ---------- -ATTTAA--- ---------- ---CC----G
N. pachyderma II        -----CGT-C TTGT--TG-- CCTTAATAAA GTTGGCTTTA AATACGA--- ---------A TTCGTATTCG TATTTAAA-- ---------- ---CC----G
N. pachyderma III       -----CGT-C TTGT--TG-- CCTTAATAAA GTTGGCTTTT AATACAAATT CCCGAATTCG TATTTAAA-- ---------- ---------- ---CC----G
N. pachyderma IV        -----CGT-C TTGT--TG-- CCTTAAACTA GCGTATATTG CAATTTATTG YAATA----- ---------- ---------- ---------- ---TC----G
N. pachyderma V         -----CGT-C TTGT--TG-- CCTTAATAAA GTTGGCTTTA AATACGAAA- -----TTCGT ---------- -ATTTAAA-- ---------- ---CC----G
N. pachyderma VI        -----CGT-C TTGT--TG-- CCTTAACACA GTTGGCTTTA AATACGA--- ---------- ----AATTCG TATTTAAA-- ---------- ---CC----G
N. pachyderma VII       -----CGT-C TTGT--TG-- CCTTAATAAA GTTGTGTTAA TAGCAATATT AA-------- ---------- ---------- ---------- ---CC----G
N. dutertrei_C          -----CGT-C TTAT--TG-- CCTT-TATCT TGCTATATTC TAATTTAATT AGAAA----- ---------- ---------- ---------- ---TA----G
N. dutertrei_Ib         -----CGT-C TTAT--TG-- CCTT-TATCT TGTTATATTC TAATTTAATT AGGAA----- ---------- ---------- ---------- ---TA----A
P. obliquiloculata_BR   -----CGT-C TTGT--TG-- CCTT-TATCT TGTTATATCT ATTA-TTT-A AT-AATAGAA ---------- ---------- ---------- ---TA----A
P. obliquiloculata_AS   -----CGT-C TTGT--TG-- CCTT-TATCT TGTTATATCT ATTATTTTT- AT-AATAGAA ---------- ---------- ---------- ---TA----A
G. inflata              -----CGT-C TTGT--TG-- CCTCTCTATA ATACCTTCTT ATTTTTAATA AGAG------ ---------- ---------- ---------- ---TA----T
G. crassaformis         -----CGT-C TTGT--TG-- CCTCTCTATA ATATCCTCTT ATTTTTAATA AGAG------ ---------- ---------- ---------- ---TA----T
N. incompta I           -----CGT-C TTGT--TG-- TCTCTCTTT- GACAGTTATG GGTTATCCCA GTCATGTGTT TATACTTTTA TGTGTAAATA CG-------- ---TA----C
N. incompta II          -----CGT-C TTGT--TG-- TCTCTCTTTT GACAGTTATG GGTTATCCCA GTCATGTGTT TATGCTTTTA TGTGTAAATA CG-------- ---TA----C
G. glutinata Ia1        -----CCT-C TTGT--TG-- CCTCATCTCC CTACTTTTTT GAGCACTTCG GT-GTT---G AATAATTTC- ATTGAGGCTT T--------- ----------
G. glutinata Ia2        -----CCT-C TTGT--TG-- CCTCATCTCC CAACATTCTT TTTTCCTTCG G--GTTAAAG A-TA-TTTC- ATTGAGGCTT T--------- ----------
G. glutinata Ia3        -----CCT-C TTGT--TG-- CCTCATCTCC CAACATTCTT TTTTCCTTCA CGGGTTAAAG A-TA-TTTC- ATTGAGGCTT T--------- ----------
C. nitida               -----CCT-C TTGT--TG-- CCTCTGTGCT CCTACACGCT CAATGCGCTA AACACTGGTT TTCT------ ---------- ---------- ----------
G. uvula                -----CCT-C TTGT--TG-- CCTTCGTTTA ACATCCTGCA GCTCGCTGTG GGTACTGAGG GCTTCACTCG GTGACTTTGG GCTGAGCAAT CGGTCT-TTT
B. variabilis           -----CCT-C TTGT--TG-- CTTTCAT-AC CCAATGC--G CG---A-TAT ATA----CTC GTATG---TT TCA------- ---CGCATAA GAAAGCTTAT
S. globigerus           -----CCT-C TTGT--TG-- CTTTCAT-AC CCAATGC--G TG-----T-T ATA--CACTC GTGTA---TA TCG------- ---CGCATAA GAAAGCTTAT
B. alata                -----CCT-C TTGT--TG-- CTTTCAT-AC CAAAGTATTA TATATCACTC GCTGCTTCGC GGCAGTGCAT ATTTTT---- ---------- ACTTAAGAAA
G. vivans               -----CCT-C TTGT--TG-- CCTTTATACC AAACACGTTT GCTCTTTT-- -GAGCTTTCG TGCAAAAAGG CTT------- ---------- ----------
C. porrectus            -----CCT-C TTGT--TG-- CTCTCATATT ACCTTATACT GCATAATATT TGAATAATTT ATTTTATTTT TTTTTATGTT TAAGAGAGCT T---------
C. ovoidea              -----CCT-C TTGT--TG-- CCTCAAATCT ATCTCCACTT TCACATTGAA ACTTTTTTAT AAAGTTACAA TTGTGTGGTG TTATGAGGCT TTT-------
G. opercularis          -----CCT-C TTGT--TG-- CCTGTATTAC CACAACAGTC TGCACTCTGT NTTTCTGTAT AAACAGGCCT TATATT---- ---------- ----------
E. aculeatum            -----TCT-T TTGT--TA-- TATATACTAT ACGCGA---- ---------- ---------- ---------- ---------- ---------- ----------
E. vitrea               -----CCT-C TTGT--TG-- CCTTTATACC AAACATGGAT ATTCTCTTCG GAGGGTTTCA TGAAAAAAGG CTT------- ---------- ----------
H. germanica            -----CCT-C TTGT--TG-- CCTGTATATA TGTGTATTTT ATACACACCA CAGGCTAT-- ---------- ---------- ---------- ----------
P. mediterranensis      -----CCT-C TTGT--TG-- CCTTCATTTC CAATTGTTCG CTATATTTCG ATATATGTGT TTCAATACGA AGGCTT---- ---------- ----------
S. fusiformis           -----CCT-C TTGT--TG-- CCTTTATACC AAACACAGTC TGTTCCTTTC TCTCGAAAGG GCTGCCGTGA AAAAAGGCTT ---------- ----------
V. fragilis             -----CCT-C TTGT--TG-- CCTTTATACC AAACATGTTG GTTTTAAAGA ATCCTTTTTT TATACCTTCG TGCAAAAGGC TT-------- ----------
A. pseudocassis         -----CCT-C TTGT--TG-- CCTTTAGCTT TATGCAGCTA GTGGCTTGAG CATTTTTTTG CTTAAAGTTT ACTGCTGCTA TTAAAGGCTT T---------
Spiroplectammina sp.    -----CCT-C TTGT--TG-- CCTCTAAACC AAACGGTAAT TTATAATTAA AATTTATTTT TTAGTTTTTA TTGCTAAAAA GAGGCTT--- ----------
Textularia sp.          -----CCT-C TTGT--TG-- CCTTTATCCC AAACGCCGTA TATAATTTAT TATTTCGGT- ---------- ------AAAA AAAGGCT--- ----------
S. limosum              -----CCT-C TTGT--TG-- CTTTTTTTTT ATTATAATTT TTTATTTTTT TAAAATTTTA TATTTATTTT ATTAATTATT TTTATAATTT TTAAATATAC
G. antarctica           -----CCT-C TTGT--TG-- CTCTCCATTT CGATTTATAC CAGAATCGTC GCGTATTAAT TTCTTAATTT TAATTTTTTT GCACTTTCGG GTGTAATTTT
D. aphelis              -----CCT-C TTGT--TG-- CCTTTATACC AAACATGTTT TGCGTCAATT TTCGAATTGC GCGCATTTCA TGAAAAAAGG CTT------- ----------
P. peruviana            -----CCT-T TTAT--TG-- CAATACTTTA TATATGTTAA TATTTTTTAT ATTAATGTAT TATATTGCTT AA-------- ---------- ----------
M. secans               -----CCC-T TTAT--TG-- CTTTATATTT ATTAAATTAA ATAAAG---- ---------- ---------- ---------- ---------- ----------
Quinqueloculina sp.     -----CCT-T TTAT--TG-- CAATATTTTT ATATTAATAT AATTTTTAAT TATATTAATT ATATTG---- ---------- ---------- ----------
N. haylinosphaira       -----CCT-C TTGT--TG-- CCTCCCATGT GCAAACTGCA TTGTATTTCT GCGTGTTGTA CTTTAGGGTA TAATATGTGT TATGCTTTCG GTTTTTGCCA
M. fusca                -----CCT-C TTGT--TG-- CCTTACGATA TTTAGACTAC TTGTAGTCTA TTAATCTAAA GGCT------ ---------- ---------- ----------
T. alba                 -----CCT-T TTGT--TG-- CCTTCTAACT AAACTATTAA GCTTTAAATT TCTTTTTTCA TTAAGAGATT TTTTTAGCCA GTTTTAACAA AGTAGGCTCT
A. mexicana             -----CCT-C TTGT--TG-- CCTTTGCTAT TAAATATATT GTCAGTGCAT TCGTGTATTG ATATTTATAT TGAAAAAGGC TATATATTAT AT--------
A. triangularis         -----CCT-C TTGT--TG-- CCTCATTCTT AATATGAATG ATATATTTAT TTGTTTTATT ACATTTAAAT GTATTATTTA TATGTGTTAT GTATTTGATA
A. rara                 -----CCT-C TTGT--TG-- CCTCATTCTT AATATGAATA TTAATATTTA AAATGATTTT ATTATTGTTT TATTTATTCT TATTTATATG TGTTATGTAT
E. scabrum              -----CCT-C TTGT--TG-- CCTTTATATA TATTTTTATG CGCTCGCGCG TAAAATAAAA AAAAGGCTTT TTT------- ---------- ----------
N. venosus              -----CCT-C TTGT--TA-- CCTTTATAAC CAAACACGTG CAGTAATAAT TCTTATTATC TCGCTTCGTG CAAAAAGGCC TT-------- ----------
B. marginata            -----CCT-C TTGT--TG-- CCTTTATACC AAACGTGCAT ATGTAATTTT TTTAAATTGC TTTGCGCGCA AAAAGGCT-- --T------- ----------
Trochammina sp.         -----CCT-C TTGT--TG-- CCTTTAGATT TAACACTGTT TAGTCATATT TATTATGCAT CTATCAGT-- ------AAAA AAAGGCT--- ----------
Peneroplis sp.          -----CCT-T TTAT--TG-- CTATAAATAA TATATATTAT TTATATTATA ATAG------ ---------- ---------- ---------- ----------
S. orbiculus            -----CCT-T TTAT--TG-- CTATTATTAT TTAATTATAG CATAA----- ---------- ---------- ---------- ---------- ----------
```

```
Allogromia sp.            -----CAT-C TTGT--TG-- CATAATCTTA TTTA------ ---------- ---------- ---------- ---------- ---------- ----------

                          801       811       821       831       841       851       861       871       881       891      900
                          |         |         |         |         |         |         |         |         |         |        |
G. siphonifera Ia1        -CAGTCAAAC GGGCGGGCGT CTTAATTGGC GCGGTCACGA GG---CTTAA TATGCTGCGG CAG------- ---------- ---------- ----------
G. siphonifera Ia2        -CAGTCAAAC GGGCGGGCGT CTTAATTGGC GCGGTCACGA GG---CTTAA TATGCTGCGG CTG------- ---------- ---------- ----------
G. siphonifera IIa1       GTATGT--AA -TCTCAAAAT TACATGTATT TGTAATGGCG AGTGAT---C A--C--A-CC TATA------ ---------- ---------- ----------
G. siphonifera IIa2       GTATATATAG -TCTCAAAAT TACATGTATT TGTAATGGCG AGTAATATAT T-ACT-ATCC TGTA------ ---------- ---------- ----------
G. siphonifera IIa3       GTATATATAG -TCTCCAAAT TACATGTATT TGTAATGGCG AGTAATATTT -A-CT-ATCC TGTA------ ---------- ---------- ----------
G. siphonifera IIa        GTATGATTAA -TCTCAAAAT TACATGTATT TGTAGTGGCG AGTATAATTT -A-CT-ATCC TATA------ ---------- ---------- ----------
G. siphonifera IIb        GT--GA---- -TCTCAAAAT TGCATGTATT TGTGATGGCG AGTGAT---- ---CTAATCC CTTA------ ---------- ---------- ----------
G. calida                 CGGGATCTCC AATCACTTTC GAGTGAGAGT --GAGTGATC TAATCCTTTA ---------- ---------- ---------- ---------- ----------
O. universa I             ---------- ---------- ---------- ---------- ---------- ---------- ---------- ---------- ---------- ----------
O. universa III           ---------- ---------- ---------- ---------- ---------- ---------- ---------- ---------- ---------- ----------
G. sacculifer             CT-ACAGTGA GATATTGTG- ---------- ---------- ---------- ---------- ---------- ---------- ---------- ----------
G. ruber pink             TTAGCCGGTG ---------- ---------- ---------- ---------- ---------- ---------- ---------- ---------- ----------
G. ruber Ia               ------GGTG ---------- ---------- ---------- ---------- ---------- ---------- ---------- ---------- ----------
G. ruber Ib1              ------GGTG ---------- ---------- ---------- ---------- ---------- ---------- ---------- ---------- ----------
G. ruber Ib2              ------GGTG ---------- ---------- ---------- ---------- ---------- ---------- ---------- ---------- ----------
G. ruber IIa              ------TGTG ---------- ---------- ---------- ---------- ---------- ---------- ---------- ---------- ----------
G. conglobatus            ------GGTG ---------- ---------- ---------- ---------- ---------- ---------- ---------- ---------- ----------
G. rubescens (pink)       ------GGTG ---------- ---------- ---------- ---------- ---------- ---------- ---------- ---------- ----------
G. bulloides Ia           TGGTAGTAGT -GGGCCAG-- ---------- ---------- ---------- ---------- ---------- ---------- ---------- ----------
G. bulloides Ib           TTAGTGGGTC -GGGCTAG-- ---------- ---------- ---------- ---------- ---------- ---------- ---------- ----------
G. bulloides IIa          TGGTTGTAAT -GGGCCAG-- ---------- ---------- ---------- ---------- ---------- ---------- ---------- ----------
G. bulloides IIb          TGGTTGTAGT -GGGCCAG-- ---------- ---------- ---------- ---------- ---------- ---------- ---------- ----------
G. bulloides IIc          TGGTTGTAAT -GGGCCAG-- ---------- ---------- ---------- ---------- ---------- ---------- ---------- ----------
G. bulloides IId          TGGTTGTAGT -GGGCCAG-- ---------- ---------- ---------- ---------- ---------- ---------- ---------- ----------
G. bulloides IIe          TGGTTGTAGT -GGTCCAG-- ---------- ---------- ---------- ---------- ---------- ---------- ---------- ----------
T. quinqueloba Ia         AACCTTTTTC A-AAGGTTG- ---------- ---------- ---------- ---------- ---------- ---------- ---------- ----------
T. quinqueloba Ib         AACCTTTTTC A-AAGGTTG- ---------- ---------- ---------- ---------- ---------- ---------- ---------- ----------
T. quinqueloba IIa        AACCATT--- --AAGGTGA- ---------- ---------- ---------- ---------- ---------- ---------- ---------- ----------
T. quinqueloba IIb        AACCATT--- --AAGGTGA- ---------- ---------- ---------- ---------- ---------- ---------- ---------- ----------
T. quinqueloba IIc        AACCATT--- --AAGGTGA- ---------- ---------- ---------- ---------- ---------- ---------- ---------- ----------
T. quinqueloba IId        AACCATT--- --AAGGTGA- ---------- ---------- ---------- ---------- ---------- ---------- ---------- ----------
G. falconensis            TAACCACTCT AAA------- ---------- ---------- ---------- ---------- ---------- ---------- ---------- ----------
H. pelagica               GTTATTTGTG TCTCTGAATG TGCTGACGAC TTCCGTCGTG GGCTGTTTAG TTATGCATTT AGCAACGACG TAGCTGTATA AGGGGGTGTC GAGAGGTTTA
G. menardii               ---------- ---------- ---------- ---------- ---------- ---------- ---------- ---------- ---------- ----------
G. ungulata               TAACGTCTTT TGACGTGAGT GTACTAACAT TTCCAGTAAC CA-------- ---------- ---------- ---------- ---------- ----------
G. hirsuta                ---------- ---------- ---------- ---------- ---------- ---------- ---------- ---------- ---------- ----------
G. scitula                ---------- ---------- ---------- ---------- ---------- ---------- ---------- ---------- ---------- ----------
G. truncatulinoides       ---------- ---------- ---------- ---------- ---------- ---------- ---------- ---------- ---------- ----------
N. pachyderma I           CT-T---ACC GAGGCTA--T ---------- ---------- ---------- ---------- ---------- ---------- ---------- ----------
N. pachyderma II          CT-T---ACA GAGGCTA--T ---------- ---------- ---------- ---------- ---------- ---------- ---------- ----------
N. pachyderma III         CT-T---ACA GAGGCTA--T ---------- ---------- ---------- ---------- ---------- ---------- ---------- ----------
N. pachyderma IV          CT-T---ACA GAGGCTA--T ---------- ---------- ---------- ---------- ---------- ---------- ---------- ----------
N. pachyderma V           CT-T---ACA GAGGCTA--T ---------- ---------- ---------- ---------- ---------- ---------- ---------- ----------
N. pachyderma VI          CT-T---ACA GAGGCTA--T ---------- ---------- ---------- ---------- ---------- ---------- ---------- ----------
N. pachyderma VII         CT-T---ACA AAGGCTA--T ---------- ---------- ---------- ---------- ---------- ---------- ---------- ----------
N. dutertrei_C            CT-A---ACA GAGGCTA-AT ---------- ---------- ---------- ---------- ---------- ---------- ---------- ----------
N. dutertrei_Ib           CT-A---ACA GAGGCTA-AT ---------- ---------- ---------- ---------- ---------- ---------- ---------- ----------
P. obliquiloculata_BR     CT-A---ACA GAGGCTA-AT ---------- ---------- ---------- ---------- ---------- ---------- ---------- ----------
P. obliquiloculata_AS     CT-A---ACA GAGGCTA-AT ---------- ---------- ---------- ---------- ---------- ---------- ---------- ----------
G. inflata                TT-A---CCT GAGGCTA-TT ---------- ---------- ---------- ---------- ---------- ---------- ---------- ----------
G. crassaformis           TT-A---CCT GAGGCTA-TT ---------- ---------- ---------- ---------- ---------- ---------- ---------- ----------
N. incompta I             GA-C---ACA GAGACTAGAT ---------- ---------- ---------- ---------- ---------- ---------- ---------- ----------
N. incompta II            GA-C---ACA GAGACTAGAT ---------- ---------- ---------- ---------- ---------- ---------- ---------- ----------
G. glutinata Ia1          ---------- ---------- ---------- ---------- ---------- ---------- ---------- ---------- ---------- ----------
G. glutinata Ia2          ---------- ---------- ---------- ---------- ---------- ---------- ---------- ---------- ---------- ----------
G. glutinata Ia3          ---------- ---------- ---------- ---------- ---------- ---------- ---------- ---------- ---------- ----------
C. nitida                 ---------- ---------- ---------- ---------- ---------- ---------- ---------- ---------- ---------- ----------
G. uvula                  TGATCTG--- ---------- ---------- ---------- ---------- ---------- ---------- ---------- ---------- ----------
B. variabilis             --ACATACTT TTGCTACGGC AATTGTAT-- ---------- ---------- ---------- ---------- ---------- ---------- ----------
S. globigerus             --ACAT-GTA TTGCTACGGC A-TTACAT-- ---------- ---------- ---------- ---------- ---------- ---------- ----------
B. alata                  --GCTTATTC TTA------- ---------- ---------- ---------- ---------- ---------- ---------- ---------- ----------
G. vivans                 ---------- ---------- ---------- ---------- ---------- ---------- ---------- ---------- ---------- ----------
C. porrectus              ---------- ---------- ---------- ---------- ---------- ---------- ---------- ---------- ---------- ----------
C. ovoidea                ---------- ---------- ---------- ---------- ---------- ---------- ---------- ---------- ---------- ----------
G. opercularis            ---------- ---------- ---------- ---------- ---------- ---------- ---------- ---------- ---------- ----------
E. aculeatum              ---------- ---------- ---------- ---------- ---------- ---------- ---------- ---------- ---------- ----------
E. vitrea                 ---------- ---------- ---------- ---------- ---------- ---------- ---------- ---------- ---------- ----------
H. germanica              ---------- ---------- ---------- ---------- ---------- ---------- ---------- ---------- ---------- ----------
P. mediterranensis        ---------- ---------- ---------- ---------- ---------- ---------- ---------- ---------- ---------- ----------
S. fusiformis             ---------- ---------- ---------- ---------- ---------- ---------- ---------- ---------- ---------- ----------
V. fragilis               ---------- ---------- ---------- ---------- ---------- ---------- ---------- ---------- ---------- ----------
A. pseudocassis           ---------- ---------- ---------- ---------- ---------- ---------- ---------- ---------- ---------- ----------
Spiroplectammina sp.      ---------- ---------- ---------- ---------- ---------- ---------- ---------- ---------- ---------- ----------
Textularia sp.            ---------- ---------- ---------- ---------- ---------- ---------- ---------- ---------- ---------- ----------
S. limosum                ACAAA----- ---------- ---------- ---------- ---------- ---------- ---------- ---------- ---------- ----------
G. antarctica             TTTTTTTATG ACTGTATTTT TTTACGTTAA GAGAGCTT-- ---------- ---------- ---------- ---------- ---------- ----------
D. aphelis                ---------- ---------- ---------- ---------- ---------- ---------- ---------- ---------- ---------- ----------
P. peruviana              ---------- ---------- ---------- ---------- ---------- ---------- ---------- ---------- ---------- ----------
M. secans                 ---------- ---------- ---------- ---------- ---------- ---------- ---------- ---------- ---------- ----------
Quinqueloculina sp.       ---------- ---------- ---------- ---------- ---------- ---------- ---------- ---------- ---------- ----------
N. haylinosphaira         CAGTTTTTTC ACTTGTATTC ATTTCGTATA TCTTTGGTGA TTTGCGAAGA CTACTTGGAT TTATATTTGT GTTATGTGGT CGCTTCAAAT GCGGCTATGT
M. fusca                  ---------- ---------- ---------- ---------- ---------- ---------- ---------- ---------- ---------- ----------
T. alba                   ATTCTCTTCA TTAAAAACAG GGGA------ ---------- ---------- ---------- ---------- ---------- ---------- ----------
A. mexicana               ---------- ---------- ---------- ---------- ---------- ---------- ---------- ---------- ---------- ----------
A. triangularis           GTATATGAAT GTTATGTGAC ATGTGTATAA TATTATATAT ATATATATAT TATATATGAN ATGATTTGTA TTGTTAGAAT TTATTTTAAT GATATTAAAT
A. rara                   TTGATTGTAT GTGAATGTAT GTTACATGTA TTTAATCTGA TGCATTCATT GTGGTTATAT ATATATATGT ATTTATACAT GAATTTATTC GTGTATTTTA
E. scabrum                ---------- ---------- ---------- ---------- ---------- ---------- ---------- ---------- ---------- ----------
N. venosus                ---------- ---------- ---------- ---------- ---------- ---------- ---------- ---------- ---------- ----------
B. marginata              ---------- ---------- ---------- ---------- ---------- ---------- ---------- ---------- ---------- ----------
Trochammina sp.           ---------- ---------- ---------- ---------- ---------- ---------- ---------- ---------- ---------- ----------
Peneroplis sp.            ---------- ---------- ---------- ---------- ---------- ---------- ---------- ---------- ---------- ----------
S. orbiculus              ---------- ---------- ---------- ---------- ---------- ---------- ---------- ---------- ---------- ----------
Allogromia sp.            ---------- ---------- ---------- ---------- ---------- ---------- ---------- ---------- ---------- ----------
```

```
                      901        911        921        931        941        951        961        971        981        991       1000
                      |          |          |          |          |          |          |          |          |          |          |
G. siphonifera Ia1    ---------- ---------- ---------- ---------- ---------- ---------- ---------- ---------- ---------- ----------
G. siphonifera Ia2    ---------- ---------- ---------- ---------- ---------- ---------- ---------- ---------- ---------- ----------
G. siphonifera IIa1   ---------- ---------- ---------- ---------- ---------- ---------- ---------- ---------- ---------- ----------
G. siphonifera IIa2   ---------- ---------- ---------- ---------- ---------- ---------- ---------- ---------- ---------- ----------
G. siphonifera IIa3   ---------- ---------- ---------- ---------- ---------- ---------- ---------- ---------- ---------- ----------
G. siphonifera IIa    ---------- ---------- ---------- ---------- ---------- ---------- ---------- ---------- ---------- ----------
G. siphonifera IIb    ---------- ---------- ---------- ---------- ---------- ---------- ---------- ---------- ---------- ----------
G. calida             ---------- ---------- ---------- ---------- ---------- ---------- ---------- ---------- ---------- ----------
O. universa I         ---------- ---------- ---------- ---------- ---------- ---------- ---------- ---------- ---------- ----------
O. universa III       ---------- ---------- ---------- ---------- ---------- ---------- ---------- ---------- ---------- ----------
G. sacculifer         ---------- ---------- ---------- ---------- ---------- ---------- ---------- ---------- ---------- ----------
G. ruber pink         ---------- ---------- ---------- ---------- ---------- ---------- ---------- ---------- ---------- ----------
G. ruber Ia           ---------- ---------- ---------- ---------- ---------- ---------- ---------- ---------- ---------- ----------
G. ruber Ib1          ---------- ---------- ---------- ---------- ---------- ---------- ---------- ---------- ---------- ----------
G. ruber Ib2          ---------- ---------- ---------- ---------- ---------- ---------- ---------- ---------- ---------- ----------
G. ruber IIa          ---------- ---------- ---------- ---------- ---------- ---------- ---------- ---------- ---------- ----------
G. conglobatus        ---------- ---------- ---------- ---------- ---------- ---------- ---------- ---------- ---------- ----------
G. rubescens (pink)   ---------- ---------- ---------- ---------- ---------- ---------- ---------- ---------- ---------- ----------
G. bulloides Ia       ---------- ---------- ---------- ---------- ---------- ---------- ---------- ---------- ---------- ----------
G. bulloides Ib       ---------- ---------- ---------- ---------- ---------- ---------- ---------- ---------- ---------- ----------
G. bulloides IIa      ---------- ---------- ---------- ---------- ---------- ---------- ---------- ---------- ---------- ----------
G. bulloides IIb      ---------- ---------- ---------- ---------- ---------- ---------- ---------- ---------- ---------- ----------
G. bulloides IIc      ---------- ---------- ---------- ---------- ---------- ---------- ---------- ---------- ---------- ----------
G. bulloides IId      ---------- ---------- ---------- ---------- ---------- ---------- ---------- ---------- ---------- ----------
G. bulloides IIe      ---------- ---------- ---------- ---------- ---------- ---------- ---------- ---------- ---------- ----------
T. quinqueloba Ia     ---------- ---------- ---------- ---------- ---------- ---------- ---------- ---------- ---------- ----------
T. quinqueloba Ib     ---------- ---------- ---------- ---------- ---------- ---------- ---------- ---------- ---------- ----------
T. quinqueloba IIa    ---------- ---------- ---------- ---------- ---------- ---------- ---------- ---------- ---------- ----------
T. quinqueloba IIb    ---------- ---------- ---------- ---------- ---------- ---------- ---------- ---------- ---------- ----------
T. quinqueloba IIc    ---------- ---------- ---------- ---------- ---------- ---------- ---------- ---------- ---------- ----------
T. quinqueloba IId    ---------- ---------- ---------- ---------- ---------- ---------- ---------- ---------- ---------- ----------
G. falconensis        ---------- ---------- ---------- ---------- ---------- ---------- ---------- ---------- ---------- ----------
H. pelagica           AATCACAGAG ---------- ---------- ---------- ---------- ---------- ---------- ---------- ---------- ----------
G. menardii           ---------- ---------- ---------- ---------- ---------- ---------- ---------- ---------- ---------- ----------
G. ungulata           ---------- ---------- ---------- ---------- ---------- ---------- ---------- ---------- ---------- ----------
G. hirsuta            ---------- ---------- ---------- ---------- ---------- ---------- ---------- ---------- ---------- ----------
G. scitula            ---------- ---------- ---------- ---------- ---------- ---------- ---------- ---------- ---------- ----------
G. truncatulinoides   ---------- ---------- ---------- ---------- ---------- ---------- ---------- ---------- ---------- ----------
N. pachyderma I       ---------- ---------- ---------- ---------- ---------- ---------- ---------- ---------- ---------- ----------
N. pachyderma II      ---------- ---------- ---------- ---------- ---------- ---------- ---------- ---------- ---------- ----------
N. pachyderma III     ---------- ---------- ---------- ---------- ---------- ---------- ---------- ---------- ---------- ----------
N. pachyderma IV      ---------- ---------- ---------- ---------- ---------- ---------- ---------- ---------- ---------- ----------
N. pachyderma V       ---------- ---------- ---------- ---------- ---------- ---------- ---------- ---------- ---------- ----------
N. pachyderma VI      ---------- ---------- ---------- ---------- ---------- ---------- ---------- ---------- ---------- ----------
N. pachyderma VII     ---------- ---------- ---------- ---------- ---------- ---------- ---------- ---------- ---------- ----------
N. dutertrei_C        ---------- ---------- ---------- ---------- ---------- ---------- ---------- ---------- ---------- ----------
N. dutertrei_Ib       ---------- ---------- ---------- ---------- ---------- ---------- ---------- ---------- ---------- ----------
P. obliquiloculata_BR ---------- ---------- ---------- ---------- ---------- ---------- ---------- ---------- ---------- ----------
P. obliquiloculata_AS ---------- ---------- ---------- ---------- ---------- ---------- ---------- ---------- ---------- ----------
G. inflata            ---------- ---------- ---------- ---------- ---------- ---------- ---------- ---------- ---------- ----------
G. crassaformis       ---------- ---------- ---------- ---------- ---------- ---------- ---------- ---------- ---------- ----------
N. incompta I         ---------- ---------- ---------- ---------- ---------- ---------- ---------- ---------- ---------- ----------
N. incompta II        ---------- ---------- ---------- ---------- ---------- ---------- ---------- ---------- ---------- ----------
G. glutinata Ia1      ---------- ---------- ---------- ---------- ---------- ---------- ---------- ---------- ---------- ----------
G. glutinata Ia2      ---------- ---------- ---------- ---------- ---------- ---------- ---------- ---------- ---------- ----------
G. glutinata Ia3      ---------- ---------- ---------- ---------- ---------- ---------- ---------- ---------- ---------- ----------
C. nitida             ---------- ---------- ---------- ---------- ---------- ---------- ---------- ---------- -----AGCTT AGTGCGTTGG
G. uvula              ---------- ---------- ---------- ---------- ---------- ---------- ---------- ---------- ---------- ----------
B. variabilis         ---------- ---------- ---------- ---------- ---------- ---------- ---------- ---------- ---------- ----------
S. globigerus         ---------- ---------- ---------- ---------- ---------- ---------- ---------- ---------- ---------- ----------
B. alata              ---------- ---------- ---------- ---------- ---------- ---------- ---------- ---------- ---------- ----------
G. vivans             ---------- ---------- ---------- ---------- ---------- ---------- ---------- ---------- ---------- ----------
C. porrectus          ---------- ---------- ---------- ---------- ---------- ---------- ---------- ---------- ---------- ----------
C. ovoidea            ---------- ---------- ---------- ---------- ---------- ---------- ---------- ---------- ---------- ----------
G. opercularis        ---------- ---------- ---------- ---------- ---------- ---------- ---------- ---------- ---------- ----------
E. aculeatum          ---------- ---------- ---------- ---------- ---------- ---------- ---------- ---------- ---------- ----------
E. vitrea             ---------- ---------- ---------- ---------- ---------- ---------- ---------- ---------- ---------- ----------
H. germanica          ---------- ---------- ---------- ---------- ---------- ---------- ---------- ---------- ---------- ----------
P. mediterranensis    ---------- ---------- ---------- ---------- ---------- ---------- ---------- ---------- ---------- ----------
S. fusiformis         ---------- ---------- ---------- ---------- ---------- ---------- ---------- ---------- ---------- ----------
V. fragilis           ---------- ---------- ---------- ---------- ---------- ---------- ---------- ---------- ---------- ----------
A. pseudocassis       ---------- ---------- ---------- ---------- ---------- ---------- ---------- ---------- ---------- ----------
Spiroplectammina sp.  ---------- ---------- ---------- ---------- ---------- ---------- ---------- ---------- ---------- ----------
Textularia sp.        ---------- ---------- ---------- ---------- ---------- ---------- ---------- ---------- ---------- ----------
S. limosum            ---------- ---------- ---------- ---------- ---------- ---------- ---------- ---------- ---------- ----------
G. antarctica         ---------- ---------- ---------- ---------- ---------- ---------- ---------- ---------- ---------- ----------
D. aphelis            ---------- ---------- ---------- ---------- ---------- ---------- ---------- ---------- ---------- ----------
P. peruviana          ---------- ---------- ---------- ---------- ---------- ---------- ---------- ---------- ---------- ----------
M. secans             ---------- ---------- ---------- ---------- ---------- ---------- ---------- ---------- ---------- ----------
Quinqueloculina sp.   ---------- ---------- ---------- ---------- ---------- ---------- ---------- ---------- ---------- ----------
N. haylinosphaira     ACACTTTTGT ATTTTCCTTG TATGTCTCTC TGTTGCTTTT GAGATATATT TGTTTATGTG TACATCGAAA TTATCTGCAG TGGA------ ----------
M. fusca              ---------- ---------- ---------- ---------- ---------- ---------- ---------- ---------- ---------- ----------
T. alba               ---------- ---------- ---------- ---------- ---------- ---------- ---------- ---------- ---------- ----------
A. mexicana           ---------- ---------- ---------- ---------- ---------- ---------- ---------- ---------- ---------- ----------
A. triangularis       TATATTATAT ATATATGTGT ATATGTGGTT TTTATACGNG TTATATGATT TTNTTTATGT ATTTAATCAT TTTGCATAAA TTG------- ----------
A. rara               TATATATATG TGATTATAGT GAGTGTTTTG GTTTTATACG TGTGGTGTAT TTTATTTATA TATTTTAATC ATTTTATATA AATTG----- ----------
E. scabrum            ---------- ---------- ---------- ---------- ---------- ---------- ---------- ---------- ---------- ----------
N. venosus            ---------- ---------- ---------- ---------- ---------- ---------- ---------- ---------- ---------- ----------
B. marginata          ---------- ---------- ---------- ---------- ---------- ---------- ---------- ---------- ---------- ----------
Trochammina sp.       ---------- ---------- ---------- ---------- ---------- ---------- ---------- ---------- ---------- ----------
Peneroplis sp.        ---------- ---------- ---------- ---------- ---------- ---------- ---------- ---------- ---------- ----------
S. orbiculus          ---------- ---------- ---------- ---------- ---------- ---------- ---------- ---------- ---------- ----------
Allogromia sp.        ---------- ---------- ---------- ---------- ---------- ---------- ---------- ---------- ---------- ----------
```

```
                        1001       1011       1021       1031       1041       1051       1061       1071       1081       1091     1100
                        |          |          |          |          |          |          |          |          |          |        |
G. siphonifera Ia1      ---------- --GGAA---- AACTTGG--- -G-CGACCGC T--------- -----GTAAT ACTTCTCTCT -----T-AAA CCA------- ---GAGGAAG
G. siphonifera Ia2      ---------- --GGAA---- AACTTGG--- -G-CGACCGC T--------- -----GTAAT ACTTCTCTCT -----T-AAA CCA------- ---GAGGAAG
G. siphonifera IIa1     ---------- --GGAA---- AACTCGG--- -G-CGACCGC T--------- -----GTAAT ATTTCTCT-T -----T-AAA ACA------- ---GAGGAAG
G. siphonifera IIa2     ---------- --GGAA---- AACTCGG--- -G-CGACCGC T--------- -----GTAAT ATTTCTCT-T -----T-AAA ACA------- ---GAGGAAG
G. siphonifera IIa3     ---------- --GGAA---- AACTCGG--- -G-CGACCGC T--------- -----GTAAT ATTTCTCT-T -----T-AAA ACA------- ---GAGGAAG
G. siphonifera IIa      ---------- --GGAA---- AACTCGG--- -G-CGACCGC T--------- -----GTAAT ATTTCTCT-T -----T-AAA ACA------- ---GAGGAAG
G. siphonifera IIb      ---------- --GGAA---- AACTTGG--- -G-CGACCGC T--------- -----GTAAT ACTTCTCT-T -----T-AAA ACA------- ---GAGGCAG
G. calida               ---------- --GGAA---- AACTCGG--- -G-CGACCGC T--------- -----GTAAT ACTTTCTTTT -----T-AAA ACA------- ---GAGGAAG
O. universa I           ---------- --GACA---- AACTCGG--- -G-GGACAGC T--------- -----T-CAA TCAATTTTCT -----C-AAA CGA------- ---GAGGAAG
O. universa III         ---------- --GGCA---- AACTCAG--- -G-GGACAGC T--------- -----T-CAA CTATTCTTCT T----C-AAA CTA------- ---GAGGAAG
G. sacculifer           ---------- --GATA---- AACTTAA--- -G-CGACCGC T--------- -----C-CAA C-ATTGTGTT TTTT-T-AAA ACT------- ---GATGAAG
G. ruber pink           ---------- --GATA---- AACTCGG--- -G-GGACTGC T--------- ----GACTAT AACCATTTCT -----C-AAA ACA------- ---GAGGAAG
G. ruber Ia             ---------- --GATA---- AACTCGG--- -G-GGACTGC T--------- ----GACTAT AACCATTTCT -----C-AAA ACA------- ---GAGGAAG
G. ruber Ib1            ---------- --GATA---- AACTCGG--- -G-GGACTGC T--------- ----GACTAT AACCATTTCT -----C-AAA ACA------- ---GAGGAAG
G. ruber Ib2            ---------- --GATA---- AACTCGG--- -G-GGACTGC T--------- ----GACTAT AACCATTTCT -----C-AAA ACA------- ---GAGGAAG
G. ruber IIa            ---------- --GATA---- AACTCGG--- -G-GGACTGC T--------- ----GACTAT AAACTTTTCT -----C-AAA CCA------- ---GAGGAAG
G. conglobatus          ---------- --GATA---- AACTCGG--- -G-GGACTGC T--------- ----GACTAT AACCATTTCT -----C-AAA ACA------- ---AAGGAAG
G. rubescens (pink)     ---------- --GATA---- AACTTTG--- -G-GGACGTC T--------- --TAAGATAA ATATTTTTCT -----C-AAA CCA------- ---GAGGAAG
G. bulloides Ia         ---------- ATTTAA---- AACTCGA--- -G-AAACATC T--------- ---------- GTGACTTTCT TTCT-T-TAC GCA------- ---GAGGAAG
G. bulloides Ib         ---------A TGTCGA---- AACTCGG--- -G-AAACATC T--------- ---------- GTGACTTTCT TTCT-T-TAC GCA------- ---GAGGAAG
G. bulloides IIa        ---------- TTTTGA---- AACTCGG--- -G-GAACATC T--------- ---------- GTGACTTTCT TTCT-T-AAC GCA------- ---GAGGAAG
G. bulloides IIb        ---------- TTTTGA---- AACTCGG--- -G-GAACATC T--------- ---------- GTGACTTTCT TTCT-T-AAC GCA------- ---GAGGAAG
G. bulloides IIc        ---------- TTTTGA---- AACTCGG--- -G-GAACATC T--------- ---------- GTGACTTTCT TTCT-T-AAC GCA------- ---GAGGAAG
G. bulloides IId        ---------- TTTTGA---- AACTCGG--- -G-GAACATC T--------- ---------- GTGACTTTCT TTCT-T-AAC GCA------- ---GAGGAAG
G. bulloides IIe        ---------T TGTTGA---- AACTCGG--- -G-GAACATC T--------- ---------- GTGACTTTCT TTCT-T-AAC GCA------- ---GAGGAAG
T. quinqueloba Ia       ---------- --AATG---- AACTTAG--- -G-CGACTGC T--------- ---------- AT-ACTTT-- -----T-AAG ATG------- ---GTCGAAG
T. quinqueloba Ib       ---------- --AATG---- AACTTAG--- -G-CGACTGC T--------- ---------- AT-ACTTT-- -----T-AAG ATG------- ---GTCGAAG
T. quinqueloba IIa      ---------- --AATG---- AACTCAG--- -G-CGACTGC T--------- ---------- AT-ACTT--- -----T-AAG ATG------- ---GTGGAAG
T. quinqueloba IIb      ---------- --AATG---- AACTCAG--- -G-CGACTGC T--------- ---------- AT-ACTT--- -----T-AAG ATG------- ---GTGGAAG
T. quinqueloba IIc      ---------- --AATG---- AACTCAG--- -G-CGACTGC T--------- ---------- AT-ACTT--- -----T-AAG ATG------- ---GTGGAAG
T. quinqueloba IId      ---------- --AATG---- AACTCAG--- -G-CGACTGC T--------- ---------- AT-ACTT--- -----T-AAG ATG------- ---GTGGAAG
G. falconensis          ---------- --TTGT---- AACTCGG--- -G-GGACCGC T--------- ---------- GTGATTTTTC TT---C-AAA CCG------- ---GAGGAAG
H. pelagica             ---------- --AATA---- AACTCAG--- -G-GGACAGC T--------- ---------- GGTTTAATGC GT---T-AAA CCA------- ---GAGGAAG
G. menardii             ---------- --TTGC---- AACTATA--- -C-ATACCAC T--------- ---------- GCTTTCTTTT CT---C-TAA CCA------- ---GGGGAAG
G. ungulata             ---------- --TTTC---- AACTATA--- -C-ATACCAC T--------- ---------- GCTTTTTTTT CTC--T-A-A CCA------- ---GGGGAAG
G. hirsuta              ---------- --TTTA---- AACTAGA--- -C-GGACCGC T--------- ---------- GTTTCTCTTT -----T-AAA CCA------- ---GAGGAAG
G. scitula              ---------- -TTATAA--- AACTAGA--- -C-GGACCGC T--------- ---------- GTTTCTTTTC CT---T-AAA CCA------- ---GAGGAAG
G. truncatulinoides     ---------- --CTGA---- AACTAGC--- -T-GGACCGC T--------- ---------- GCTTTCCTT- -----T-AAG CCA------- ---GAGGAAG
N. pachyderma I         ---------- --TTAA---- AACTAGA--- -C-GGACCGC T--------- ---------- GTTTCTTTTC T----T-AAA CCA------- ---GAGGAAG
N. pachyderma II        ---------- --TTAA---- AACTAGA--- -C-GGACCGC T--------- ---------- GTTTCTTTTC T----T-AAA CCA------- ---GAGGAAG
N. pachyderma III       ---------- --TTAA---- AACTAGA--- -C-GGACCGC T--------- ---------- GTTTCTTTTC T----T-AAA CCA------- ---GAGGAAG
N. pachyderma IV        ---------- --TTAA---- AACTAGA--- -C-GGACCGC T--------- ---------- GTTTCTTT-C T----T-AAA CCA------- ---GAGGAAG
N. pachyderma V         ---------- --TTAA---- AACTAGA--- -C-GGACCGC T--------- ---------- GTTTCTTTTC T----T-AAA CCA------- ---GAGGAAG
N. pachyderma VI        ---------- --TTAA---- AACTAGA--- -C-GGACCGC T--------- ---------- GTTTCTTTTC T----T-AAA CCA------- ---GAGGAAG
N. pachyderma VII       ---------- --TTAA---- AACTAGA--- -C-GGACCGC T--------- ---------- GTTTCTTTTC T----T-AAA CCA------- ---GAGGAAG
N. dutertrei_C          ---------- --TTAA---- AATTAGA--- -C-GGACCGC T--------- ---------- GTA-CTTTTC T----T-AAA CCA------- ---CAGGAAG
N. dutertrei_Ib         ---------- --TTAA---- AATTAGA--- -C-GGACCGC T--------- ---------- GTA-CTTTTC T----T-AAA CCA------- ---GAGGAAG
P. obliquiloculata_BR   ---------- --CTAA---- AACTAGA--- -C-GGACCGC T--------- ---------- GTTTCTTT-C T----T-AAA CCA------- ---GAGGAAG
P. obliquiloculata_AS   ---------- --CTAA---- AACTAGA--- -C-GGACCGC T--------- ---------- GTTTCTTT-C T----T-AAA CCA------- ---GAGGAAG
G. inflata              ---------- --TTAA---- AACTAGA--- -C-GGACCGC T--------- ---------- GTTTCTTT-C T----T-AAA CCA------- ---GAGGAAG
G. crassaformis         ---------- --TTAA---- AACTAGA--- -C-GGACCGC T--------- ---------- GTTTTCTTTC T----T-AAA CCA------- ---GAGGAAG
N. incompta I           ---------- --ACCA---- AACTAGG--- -C-GTACCGC T--------- ---------- GTATCATTTC TT---T-AAA CCA------- ---GAGGAAG
N. incompta II          ---------- --ACCA---- AACTAGG--- -C-GTACCGC T--------- ---------- GTATCATTTC TT---T-AAA CCA------- ---GAGGAAG
G. glutinata Ia1        ---------- --CCAA---- AACTAGA--- -G-GGACCGC T--------- ---------- GTCAACTTCT -----T-AAA CCA------- ---GAGGAAG
G. glutinata Ia2        ---------- --CCAA---- AACTAGA--- -G-GGACCGC T--------- ---------- GTCAACTTCT -----T-AAA CCA------- ---GAGGAAG
G. glutinata Ia3        ---------- --CCAA---- AACTAGA--- -G-GGACCGC T--------- ---------- GTCAACTTCT -----T-AAA CCA------- ---GAGGAAG
C. nitida               TTGAGGCTTT --CTTA---- AACTAGA--- -G-GGACCGC T--------- ---------- GTCAACTTCT -----T-AAA CCA------- ---GAGGAAG
G. uvula                ---------- --AGTA---- AACTAGA--- -G-GGACCGC T--------- ---------- GTCAACTTCT -----T-AAA CCA------- ---GAGGAAG
B. variabilis           ---------- --CACA---- AACTAGA--- -G-GGACCGC T--------- ---------- GTTACTTTCT -----T-AAA CCA------- ---GAGGAAG
S. globigerus           ---------- --CACA---- AACTAGA--- -G-GGACCGC T--------- ---------- GTTACTTTCT -----T-AAA CCA------- ---GAGGAAG
B. alata                ---------- --CACA---- AACTAGA--- -G-GGACCGC T--------- ---------- GTTACTTTCT -----T-AAA CCA------- ---GAGGAAG
G. vivans               ---------- --TTTA---- AACTAGA--- -G-GGACCGC T--------- ---------- GTTACTTTCT -----T-AAA CCA------- ---GAGGAAG
C. porrectus            ---------- --TCTA---- AACTAGA--- -G-GGACCGC T--------- ---------- GTTACTTTCT -----T-AAA CCA------- ---GAGGAAG
C. ovoidea              ---------- --TTTA---- AACTAGA--- -G-GGACCGC T--------- ---------- GTTACTTTCT -----T-AAA CCA------- ---GAGGAAG
G. opercularis          ---------- --AATA---- AACTAGA--- -G-GGACCGC T--------- ---------- GTTACTTTCT -----T-AAA CCA------- ---GAGGAAG
E. aculeatum            ---------G TATATA---- AACCAAAATG TG-AGACCGC T--------- ---------- GTTTCTTTCT TT---T-AAA CCA------- ---GAGGAAG
E. vitrea               ---------- --TTTA---- AACTAGA--- -G-GGACCGC T--------- ---------- GTTACTTTCT -----T-AAA CCA------- ---GAGGAAG
H. germanica            ---------- --TATA---- AACTAGA--- -G-GGACCGC T--------- ---------- GTTACTTTCT TT---T-AAA CCA------- ---GAGGAAG
P. mediterranensis      ---------- --TCTA---- AACTAGA--- -G-GGACCGC T--------- ---------- GTTATCTTCT -----T-AAA CCA------- ---GAGGAAG
S. fusiformis           ---------- --TTTA---- AACTAGA--- -G-GGACCGC T--------- ---------- GTTACTTTCT -----T-AAA CCA------- ---GAGGAAG
V. fragilis             ---------- --TTTA---- AACTAGA--- -G-GGACCGC T--------- ---------- GTTATCTTCT -----T-AAA CCA------- ---GAGGAAG
A. pseudocassis         ---------- --TTAA---- AACTAGA--- -G-GGACCGC T--------- ---------- GTAATCTTT- -----T-AAA CCA------- ---GAGGAAG
Spiroplectammina sp.    ---------- --TTTA---- AACTAGA--- -G-GGACCGC T--------- ---------- GTAATCTTTT -----T-AAA CCA------- ---GAGGAAG
Textularia sp.          ---------- --TTTA---- AACTAGA--- -G-GGACCGC T--------- ---------- GTAATCTTTT -----T-AAA CCA------- ---GAGGAAG
S. limosum              ---------- --AGCT---- AACTAGA--- -G-GGACTGC T--------- ---------- GATATCTTGT -----T-AAA ACA------- ---GAGGAAG
G. antarctica           ---------- --TCTA---- AACTAGA--- -G-GGACCGC T--------- ---------- GTTACTTTCT -----T-AAA CCA------- ---GAGGAGG
D. aphelis              ---------- --TTTA---- AACTAGA--- -G-GGACCGC T--------- ---------- GTTACTTTCT -----T-AAA CCA------- ---GAGGAAG
P. peruviana            ---------- ---------- AATTAAA--- -G-GAACCGC TGTCTAAAA- ---------- TTTAGTCT-- -----T-AAA ACA------- ---GAGGAAG
M. secans               ---------- --CTTAA--- AATTAAG--- -G-GAACCGC T--------- -------GTC TAATATT-TA -GTGTT-AAA ACA------- ---GAGGAAG
Quinqueloculina sp.     ---------- --CTTAA--- AATTAAA--- -G-GAACCGC T--------- -------GTC TAATATT-TA -GTTTT-AAA ACA------- ---GAGGAAG
N. haylinosphaira       ---------- --GGGA---- AACTAGA--- -G-GGACCGC T--------- ---------- GACTCTTT-- -----A-TAA CCA------- ---GAGGAAG
M. fusca                ---------- --TTAA---- AACTAGA--- -G-GGACCGC T--------- ---------- GTCTTTACCC T----T-AAA CCA------- ---GAGGAAG
T. alba                 ---------- --ATAA---- AACTAAA--- -G-GGACCGC T--------- ---------- GCGACTTTTT -----TTAAA CCA------- ---GAGGAAG
A. mexicana             ---------- --ATAA---- AACTAGA--- -G-GGACCGC T--------- ---------- GTTTCTTTCT -----T-AAA CCA------- ---GAGGAAG
A. triangularis         ---------- --AGGNA--- AACGAGA--- -G-GGACCGC T--------- --------AG GCTAGCTTTT -----T-AAA ACA------- ---GAGGAAG
A. rara                 ---------- --AGGCT--- AACTAGA--- -G-GGACCGC T--------- --------AG GCTAGCTTTT -----T-AAA ACA------- ---GAGGAAG
E. scabrum              ---------- --ATA----- AACTAGA--- -G-GGACCGC TG-------- ---------- TAACTTTTT- -----T-AAA CCA------- ---GAGGAAG
N. venosus              ---------- --TTA----- AACTAGA--- -G-GGACCGC TG-------- ---------- TTACTTTCT- -----T-AAA CCA------- ---GAGGAAG
B. marginata            ---------- --TTTA---- AACTAGA--- -G-GGACCGC T--------- ---------- GTTACTTTCT -----T-AAA CCA------- ---GAGGAAG
Trochammina sp.         ---------- --TTTA---- AACTAGA--- -G-GGACCGC T--------- ---------- GTAATCTTTT -----T-AAA CCA------- ---GAGGAAG
Peneroplis sp.          ---------- --CATAA--- AATTAAA--- -G-GGACCGC TGTCTAAAT- ---------- -TTTAGTGT- -----T-AAA ATA------- ---GAGTAAG
S. orbiculus            ---------- ---------- AATTAAA--- -G-GGACCGC TGTCATTAC- ---------- TAAATATGTG T----T-AAA ATA------- ---GAGTAAG
Allogromia sp.          ---------- --TGCT---- AACTAGA--- -T-GGACCGC T--------- ---------- G-GATCTTTT C----T-A-A ACA------- ---GAGGAAG
```

```
                        1101      1111      1121      1131      1141      1151      1161      1171      1181      1191     1200
                        |         |         |         |         |         |         |         |         |         |         |
G. siphonifera Ia1      ATTGCGGCAA -TAACA-GGT CTG-TGATGC CC-TCAG-AT GTCCCGGG-C CGCACACGTG CTACAT-TGA ------TT-A G-CG-CA-GT GCGCATACTT
G. siphonifera Ia2      ATTGCGGCAA -TAACA-GGT CTG-TGATGC CC-TCAG-AT GTCCCGGG-C CGCACACGTG CTACAT-TGA ------TT-A G-CG-CA-GT GCGCATACTT
G. siphonifera IIa1     ATTGCGGCAA -TAACA-GGT CTG-TGATGC CC-TCAG-AT GTCCCGGG-C CGCACACGTG CTACAT-TGA ------TT-A G-CG-CA-GT GCGCATACTT
G. siphonifera IIa2     ATTGCGGCAA -TAACA-GGT CTG-TGATGC CC-TCAG-AT GTCCCGGG-C CGCACACGTG CTACAT-TGA ------TT-A G-CG-CA-GT GCGCATACTT
G. siphonifera IIa3     ATTGCGGCAA -TAACA-GGT CTG-TGATGC CC-TCAG-AT GTCCCGGG-C CGCACACGTG CTACAT-TGA ------TT-A G-CG-CA-GT GCGCATACTT
G. siphonifera IIa      ATTGCGGCAA -TAACA-GGT CTG-TGATGC CC-TCAG-AT GTCCCGGG-C CGCACACGTG CTACAT-TGA ------TT-A G-CG-CA-GT GCGCATACTT
G. siphonifera IIb      GTTGCGGCAA -TAACA-GGT CTG-TGATGC CC-TCAG-AT GTCCCGGG-C CGCACACGTG CTACAT-TGA ------TT-A G-CG-CA-GT GCGCATACTT
G. calida               ATTGCGGCAA -TAACA-GGT CTG-TGATGC CC-TCAG-AT GTCCCGGG-C CGCACACGTG CTACAT-TGA ------TT-A G-CG-CA-GT GCGCATACTT
O. universa I           GTTGCGGCAA -TAACA-GGT CTG-TGATGC CC-TCAG-AT GTCCTGGG-C CGCACACGTG CTACAT-TGA ------TC-A G-CG-CA-GT GCGTCTTTTT
O. universa III         GTTGCGGCAA -TAACA-GGT CTG-TGATGC CC-TCAG-AT GTTCTAGG-C TGCACACGTG CTACAT-TGA ------TC-A G-CA-CA-AT GCGATACATT
G. sacculifer           GTTGCGGCAA -TAACA-GGT CTG-TGATGC CC-TTCG-AT GTCCTGGG-C TGCACACGTG CTACAA-TGG ------CT-A G-CG-CA-GT GAGCATATTT
G. ruber pink           GTTGCGGCAA -TAACA-GGT CTG-TGATGC CC-TTAG-AT GTCCCGGG-C TGCACACGTG CTACAA-TGA ------GT-G G-CG-CA-AT GTGTGCAATT
G. ruber Ia             GTTGCGGCAA -TAACA-GGT CTG-TGATGC CC-TCAG-AT GTCCCGGG-C TGCACACGTG CTACAA-TGA ------GT-G G-CG-CA-AT GTGCTAATGT
G. ruber Ib1            GTTGCGGCAA -TAACA-GGT CTG-TGATGC CC-TCAG-AT GTCCCGGG-C TGCACACGTG CTACAA-TGA ------GTTG G-CG-CA-AT GTGCTAATGT
G. ruber Ib2            GTTGCGGCAA -TAACA-GGT CTG-TGATGC CC-TCAG-AT GTCCCGGG-C TGCACACGTG CTACAA-TGA ------GT-G G-CG-CA-AT GTGCTAATGT
G. ruber IIa            GTTGCGGCAA -TAACA-GGT CTG-TGATGC CC-TCAG-AT GTTCCGGG-C TGCACACGTG CTACAT-TTA ------GT-A G-CG-CA-GT GTGCACATTA
G. conglobatus          GTTGCGGCAA -TAACA-GGT CTG-TGATGC CC-TCAG-AT GTCCCGGG-C TGCACACGTG CTACAA-TGA ------GT-A G-CG-CA-GT GTGCACATTT
G. rubescens (pink)     ATTGCGGCAA -TAACA-GGT CTG-TGATGC CC-TCAG-AT GTCCCGGG-C TGCACACGTG CTACAT-TGA ------GT-A G-CG-CA-GT GTGTAAACTT
G. bulloides Ia         GTTATGGCAA -TAACA-GGT CTG-TGATGC CC-TCAG-AT GTCCAGAG-C TGCACACGTA CTACAG-TGA ------TC-G A-CT-CA-CT AAGTGTCGT-
G. bulloides Ib         GTTATGGCAA -TAACA-GGT CTG-TGATGC CC-TCAG-AT GTCCAAAG-C TGCACACGTA CTACAG-TGA ------TC-G T-AA-CA-CT T-GCGTTGTT
G. bulloides IIa        GTTATGGCAA -TAACA-GGT CTG-TGATGC CC-TTAG-AT GTCCAGGG-C TGCACACGTA CTACAT-TGA ------TC-A A-CT-CA-GT AGGCGTCGT-
G. bulloides IIb        GTTATGGCAA -TAACA-GGT CTG-TGATGC CC-TTAG-AT GTCCAGGG-C TGCACACGTA CTACAT-TGA ------TC-A A-CT-CA-GT AGGCGTCGT-
G. bulloides IIc        GTTATGGCAA -TAACA-GGT CTG-TGATGC CC-TTAG-AT GTCTAGGG-C TGCACACGTA CTACAT-TGA ------TC-A A-CT-CA-GT AGGCGTCGT-
G. bulloides IId        GTTATGGCAA -TAACA-GGT CTG-TGATGC CC-TTAG-AT GTCCAGGG-C TGCACACGTA CTACAT-TGA ------TC-A A-CT-CA-GT AGGCGTCGT-
G. bulloides IIe        GTTATGGCAA -TAACA-GGT CTG-TGATGC CC-TTAG-AT GTCCAGGG-C TGCACACGTA CTACAT-TGA ------TT-A A-CT-CA-GT AGGCGTCGT-
T. quinqueloba Ia       GTTGTGGCAA -TGACA-GGT CTG-TGATGC CC-TTAG-AT GTTCAGGG-C TGCACACGTA CTACAT-TGA ------TC-T A-GT-CA-AT AAGTATGTGT
T. quinqueloba Ib       GTTGTGGCAA -TGACA-GGT CTG-TGATGC CC-TTAG-AT GTTCAGGG-C TGCACACGTA CTACAT-TGA ------TC-T A-GT-CA-AT AAGTATGTGT
T. quinqueloba IIa      GTTGTGGCAA -TGACA-GGT CTG-TGATGC CCTTTAG-AT GTTCAAGG-C TGCACACGTA CTACAT-TGA ------TC-T A-GT-CA-AC GAGTATGTAT
T. quinqueloba IIb      GTTGTGGCAA -TGACA-GGT CTG-TGATGC CCTTTAG-AT GTTCAAGG-C TGCACACGTA CTACAT-TGA ------TC-T A-GT-CA-AC GAGTATGTAT
T. quinqueloba IIc      GTTGTGGCAA -TGACA-GGT CTG-TGATGC CCTTTAG-AT GTTCAAGG-C TGCACACGTA CTACAT-TGA ------TC-T A-GT-CA-AC GAGTATGTAT
T. quinqueloba IId      GTTGTGGCAA -TGACA-GGT CTG-TGATGC CCTTTAG-AT GTTCAAGG-C TGCACACGTA CTACAT-TGA ------TC-T A-GT-CA-AC GAGTATGTAT
G. falconensis          GTTGCGGCAA -TAACA-GGT CTG-TGATGC CC-TCAG-AT GTCCTGGG-C TGCACACGTG CTACAC-TGA ------TC-T C-CA-CA-GT TGGCATGTGA
H. pelagica             GATGCGGCAA -TAACA-GGT CTG-TGATGC CC-TCAG-AT GTTCCGGG-C TGCACACGTG CTACAT-TGA ------TT-A G-CA-CA-TT GTGTATGCCA
G. menardii             GTTGCGGCAA -TAACA-GGT CTG-TGATGC CC-TCAG-AT GTTCCGGG-C TGCACACGTG CTACAA-TGA ------TG-A G-CA-TA-GT GTGTGCCTTT
G. ungulata             GTTGCGGCAA -TAACA-GGT CTG-TGATGC CC-TCAG-AT GTTCCGGG-C TGCACACGTG CTACAA-TGA ------TG-A G-CA-CA-GT GTGTGCCTTT
G. hirsuta              GTTGCGGCAA -TAACA-GGT CTG-TGATGC CC-TTAG-AT GTTCCGGG-C TGCACACGTG CTACAA-TGA ------TC-A G-TA-CA-GT GAGCATCTCA
G. scitula              GTTGCGGCAA -TAACA-GGT CTG-TGATGC CC-TTAG-AT GTTCCGGG-C TGCACACGTG CTACAA-TGA ------TC-A G-TA-CA-GT GAGCATCTCA
G. truncatulinoides     GTTGCGGCAA -TAACA-GGT CTG-TGATGC CC-TCAG-AT GTTCCGGG-C CGCACACGTG CTACAA-TGA ------TC-A G-CA-CA-AA GAGTATTACC
N. pachyderma I         GTTGCGGCAA -TAACA-GGT CTG-TGATGC CC-TTAG-AT GTTCCGGG-C TGCACACGTG CTACAA-TGA ------TC-A G-TA-CA-GT GAGCATCTC-
N. pachyderma II        GTTGCGGCAA -TAACA-GGT CTG-TGATGC CC-TTAG-AT GTTCCGGG-C TGCACACGTG CTACAA-TGA ------TC-A G-TA-CA-GT GAGCATCTC-
N. pachyderma III       GTTGCGGCAA -TAACA-GGT CTG-TGATGC CC-TTAG-AT GTTCCGGG-C TGCACACGTG CTACAA-TGA ------TC-A G-TA-CA-GT GAGCATCTC-
N. pachyderma IV        GTTGCGGCAA -TAACA-GGT CTG-TGATGC CC-TTAG-AT GTTCCGGG-C TGCACACGTG CTACAA-TGA ------TC-A G-TA-CA-GT GAGCATCTC-
N. pachyderma V         GTTGCGGCAA -TAACA-GGT CTG-TGATGC CC-TTAG-AT GTTCCGGG-C TGCACACGTG CTACAA-TGA ------TC-A G-TA-CA-GT GAGCATCTC-
N. pachyderma VI        GTTGCGGCAA -TAACA-GGT CTG-TGATGC CC-TTAG-AT GTTCCGGG-C TGCACACGTG CTACAA-TGA ------TC-A G-TA-CA-GT GAGCATCTC-
N. pachyderma VII       GTTGCGGCAA -TAACA-GGT CTG-TGATGC CC-TTAG-AT GTTCCGGG-C TGCACACGTG CTACAA-TGA ------TC-A G-TA-CA-GT GAGCATCTC-
N. dutertrei_C          GTTGCGGCAA -TAACA-GGT CTG-TGATGC CC-TTAG-AT GTTCCGGG-C TGCACACGTG CTACAA-TGA ------TC-A G-TA-CA-GT GAGCATCTC-
N. dutertrei_Ib         GTTGCGGCAA -TAACA-GGT CTG-TGATGC CC-TTAG-AT GTTCCGGG-C TGCACACGTG CTACAA-TGA ------TC-A G-TA-CA-GT GAGCATCTC-
P. obliquiloculata_BR   GTTGCGGCAA -TAACA-GGT CTG-TGATGC CC-TTAG-AT GTTCCGGG-C TGCACACGTG CTACAA-TGA ------TC-A G-TA-CA-GT GAGCATCTC-
P. obliquiloculata_AS   GTTGCGGCAA -TAACA-GGT CTG-TGATGC CC-TTAG-AT GTTCCGGG-C TGCACACGTG CTACAA-TGA ------TC-A G-TA-CA-GT GAGCATCTC-
G. inflata              GTTGCGGCAA -TAACA-GGT CTG-TGATGC CC-TTAG-AT GTTCCGGG-C TGCACACGTG CTACAA-TGA ------TC-A G-TA-CA-GT GAGCATCTC-
G. crassaformis         GTTGCGGCAT -AAACA-GGT CTG-TGATGC CC-TTAG-AT GTTCCGGG-C TGCACACGTG CTACAA-TGA ------TC-A G-TA-CA-GT GAGCATCTC-
N. incompta I           GTTGCGGCAA -TAACA-GGT CTG-TGATGC CC-TTAG-AT GTTCTGGG-C TGCACACGTG CTACAA-TGA ------TC-A G-CA-CA-GT AAGCATCTC-
N. incompta II          GTTGCGGCAA -TAACA-GGT CTG-TGATGC CC-TTAG-AT GTTCTGGG-C TGCACACGTG CTACAA-TGA ------TC-A G-CA-CA-GT AAGCATCTC-
G. glutinata Ia1        GTTGCGGCAA -TAACA-GGT CTG-TGATGC CC-TTAG-AT GTTCCGGG-C TGCACACGTG CTACAA-TGA ------TT-G T-TG-CA-GT GAGCATCTCA
G. glutinata Ia2        GTTGCGGCAA -TAACA-GGT CTG-TGATGC CC-TTAG-AT GTTCCGGG-C TGCACACGTG CTACAA-TGA ------TT-G T-TG-CA-GT GAGCATCTCA
G. glutinata Ia3        GTTGCGGCAA -TAACA-GGT CTG-TGATGC CC-TTAG-AT GTTCCGGG-C TGCACACGTG CTACAA-TGA ------TT-G T-TG-CA-GT GAGCATCTCA
C. nitida               GTTGCGGCAA -TAACA-GGT CTG-TGATGC CC-TTAG-AT GTTCCGGG-C TGCACACGTG CTACAA-TGA ------TT-G T-TG-CA-GT GAGCATCTCA
G. uvula                GTTGCGGCAA -TAACA-GGT CTG-TGATGC CC-TTAG-AT GTTCCGGG-C TGCACACGTG CTACAA-TGA ------TC-G T-AG-CA-GT GAGCATCTTA
B. variabilis           GTTGCGGCAA -TAACA-GGT CTG-TGATGC CC-TTAG-AT GTTCCGGG-C TGCACACGTG CTACAA-TGA ------TT-A T-TG-CA-GT GAGCATCTCA
S. globigerus           GTTGCGGCAA -TAACA-GGT CTG-TGATGC CC-TTAG-AT GTTCCGGG-C TGCACACGTG CTACAA-TGA ------TT-A T-TG-CA-GT GAGCATCTCA
B. alata                GTTGCGGCAA -TAACA-GGT CTG-TGATGC CC-TTAG-AT GTTCCGGG-C TGCACACGTG CTACAA-TGA ------TT-A T-TG-CA-GT GAGCATCTCA
G. vivans               GTTGCGGCAA -TAACA-GGT CTG-TGATGC CC-TTAG-AT GTTCCGGG-C TGCACACGTG CTACAA-TGA ------TT-A T-TG-CA-GT GAGCATCTCA
C. porrectus            GTTGCGGCAA -TAACA-GGT CTG-TGATGC CC-TTAG-AT GTTCCGGG-C TGCACACGTG CTACAA-TGA ------TT-A T-TG-CA-GT GAGCATCTCA
C. ovoidea              GTTGCGGCAA -TAACA-GGT CTG-TGATGC CC-TTAG-AT GTTCCGGG-C TGCACACGTG CTACAA-TGA ------TC-A T-TG-CA-GT GAGCATCTCA
G. opercularis          GTTGCGGCAA -TAACA-GGT CTG-TGATGC CC-TTAG-AT GTTCCGGG-C TGCACACGTG CTACAA-TGA ------TT-A T-TG-CA-GT GAGCATCTCA
E. aculeatum            GTTATGGCAA -TAACA-GGT CTG-TGATGC CC-TTCG-AT GTTCCGGG-C TGCACACGTG CTACAA-TGA ------TT-A T-AT-CA-TT AAGTACATAT
E. vitrea               GTTGCGGCAA -TAACA-GGT CTG-TGATGC CC-TTAG-AT GTTCCGGG-C TGCACACGTG CTACAA-TGA ------TT-A T-TG-CA-GT GAGCATCTCA
H. germanica            GTTGCGGCAA -TAACA-GGT CTG-TGATGC CC-TTAG-AT GTTCCGGG-C TGCACACGTG CTACAA-TGA ------TC-A T-TG-CA-GT GCGCATCTCA
P. mediterranensis      GTTGCGGCAA -TAACA-GGT CTG-TGATGC CC-TTAG-AT GTTCCGGG-C TGCACACGTG CTACAA-TGA ------TT-A T-TG-CA-GT GAGCATCTCA
S. fusiformis           GTTGCGGCAA -TAACA-GGT CTG-TGATGC CC-TTAG-AT GTTCCGGG-C TGCACACGTG CTACAA-TGA ------TT-A T-TG-CA-GT GAGCATCTCA
V. fragilis             ATTGCGGCAA -TAACA-GGT CTG-TGATGC CC-TTAG-AT GTTCCGGG-C TGCACACGTG CTACAA-TGA ------TT-A T-TG-CA-GT GAGCATCTCA
A. pseudocassis         GTTGCGGCAA -TAACA-GGT CTG-TGATGC CC-TTAG-AT GTTCCGGG-C TGCACACGTG CTACAA-TGA ------TT-A T-TG-CA-GT GAGCATCTCA
Spiroplectammina sp.    GTTGCGGCAA -TAACA-GGT CTG-TGATGC CC-TTAG-AT GTTCCGGG-C TGCACACGTG CTACAA-TGA ------TT-A T-TG-CA-GT GAGCATCTCA
Textularia sp.          GTTGCGGCAA -TAACA-GGT CTG-TGATGC CC-TTAG-AT GTTCCGGG-C TGCACACGTG CTACAA-TGA ------TT-A T-TG-CA-GT GAGCATCTCA
S. limosum              GTTGCGACAA -TAACA-GGT CTG-TGATGC CC-TTAG-AT GTTCCGGG-C TGCACACGTG CTACAA-TGA ------TT-A T-TG-CA-GT GAGCATCTTA
G. antarctica           GTTGCGGCAA -TAACA-GGT CTG-TGATGC CC-TTAG-AT GTTCCNGG-C TGCACACGTG CTACAA-TGA ------TT-A T-TG-CA-GT GAGCATCTCA
D. aphelis              GTTGCGGCAA -TAACA-GGT CTG-TGATGC CC-TTAG-AT GTTCCGGG-C TGCACACGTG CTACAA-TGA ------TT-A T-TG-CA-GT GAGCATCTCA
P. peruviana            ATTATGGCAA -TAACA-GGT CTG-TGATGC CC-TCAG-AT GTTCTGGG-C TGCACACGTG CTACAA-TAA ------TC-A C-TA-CA-GT AAGTGTCTAA
M. secans               ATTATGGCAA -TAACA-GGT CTG-TGATGC CC-TCAG-AT GTTCTGGG-C TGCACACGTG CTACAA-TAA ------TT-A C-TA-TA-CT TAGTGTCTAC
Quinqueloculina sp.     ATTATGGCAA -TAACA-GGT CTG-TGATGC CC-TCAG-AT GTTCTGGG-C TGCACACGTG CTACAA-TAA ------TT-A C-TA-TA-AT AAGTGTCTAA
N. haylinosphaira       GTTGCGGCAA -TAACA-GGT CTG-TGATGC CC-TTAG-AT GTTCCGGG-C TGCACACGTG CTACAA-TGA ------TT-A T-TG-CA-GT GAGCATCTAC
M. fusca                GATGCGGCAA -TAACA-GGT CTG-TGATGC CC-TCAG-AT GTTCCGGG-C TGCACACGTG CTACAA-TGA ------TC-A T-AG-CA-GT GAGCATCTAA
T. alba                 GTTGCGGCAA -TAACA-GGT CTG-TGATGC CC-TTAG-AT GTTCCGGG-C TGCACACGTG CTACAA-TGA ------TT-A T-TG-CA-GT AAGCATCTCA
A. mexicana             GATGCGGCAA -TAACA-GGT CTG-TGATGC CC-TTAG-AT GTTCCGGG-C TGCACACGTG CTACAA-TGA ------TT-A T-TG-CA-CT GAGCATCTCA
A. triangularis         GTTGCGGCAA -TAACA-GGT CTG-TGATGC CC-TCAG-AT GTTCCGGG-C TGCACACGTG CTACAA-TGA ------TT-A T-TG-CA-GT GAGCATCTCA
A. rara                 GTTGCGGCAA -TAACA-GGT CTG-TGATGC CC-TCAG-AT GTTCCGGG-C TG-ACACGTG CTACAA-TGA ------TT-A T-TG-CA-GT GAGCATCTCA
E. scabrum              GTTGCGGCAA -TAACA-GGT CTG-TGATGC CC-TTAG-AT GTTCCGGG-C TGCACACGTG CTACAA-TGA ------TC-A T-TG-CA-GT AAGCATCTCA
N. venosus              GTTGCGGCAA -TAACA-GGT CTG-TGATGC CC-TTAG-AT GTTCCGGG-C TGCACACGTG CTACAA-TGA ------TT-A T-TG-CA-GT GAGCATCTCA
B. marginata            GTTGCGGCAA -TAACA-GGT CTG-TGATGC CC-TTAG-AT GTTCCGGG-C TGCACACGTG CTACAA-TGA ------TT-A T-TG-CA-GT GAGCATCTCA
Trochammina sp.         GTTGCGGCAA -TAACA-GGT CTG-TGATGC CC-TTAG-AT GTTCCGGG-C TGCACACGTG CTACAA-TGA ------TT-A T-TG-CA-GT GAGCATCTCA
Peneroplis sp.          ATTACGGCAA -TAACA-GGT CTG-TGATGC CC-TCAG-AT GTTCTGGG-C TGCACACGTG CTACAA-TAA ------TT-A C-AT-TA-AT AAGTATCTAT
S. orbiculus            ATTACGGCAA -TAACA-GGT CTG-TGATGC CC-TCAG-AT GTTCTGGG-C TGCACACGTG CTACAA-TAA ------TT-A C-AT-TA-AT AAGTATATAT
Allogromia sp.          ATTGCGGCAA -TAACA-GGT CTG-TGATGC CC-TCAG-AT GTTCTGGG-C TGCACACGTG CTACAA-TGA ------TT-A T-TG-CA-GT AAGCATCTAT
```

```
                       1201       1211       1221       1231       1241       1251       1261       1271       1281       1291         1300
                       |          |          |          |          |          |          |          |          |          |            |
G. siphonifera Ia1     ACA-TAT--- ---AG-ATCA TT----G-AT T--G-G-TTA TTT---ATAA CCG------- ---------- ---------- ---------- ----TCAATA
G. siphonifera Ia2     ACA-TAT--- ---AG-ATCA TT----G-AT T--G-G-TTA TTT---ATAA CCG------- ---------- ---------- ---------- ----TCAATA
G. siphonifera IIa1    TCAATAT--- ---GA-TACA TT---AG-AT T--G-GATAG CTT------- -----TT--G CTTCCAT--- ---------- ---------- ----CTAATA
G. siphonifera IIa2    TCAATAT--- ---GA-TACA TT---AG-AT T--G-GATTG TTCTG----- -----TTGAG CT-CCAT--- ---------- ---------- ----CTAATA
G. siphonifera IIa3    TCAATAT--- ---GA-TACA TT---AG-AT T--G-GATTG TTCTG----- -----TTGAG CT-CCAT--- ---------- ---------- ----CTAATA
G. siphonifera IIa     TCAATAT--- ---GA-TACA TT---AG-AT T--G-GATTG TTTTA----- -----TTAAG CT-CCAT--- ---------- ---------- ----CTAATA
G. siphonifera IIb     TCAATAT--- ---GA-TACA TT---GG-AT T--G-GTGGT GAATT----- -----GTTGG CC-CCGT--- ---------- ---------- ----CTAATA
G. calida              TCAATAT--- ---GA-TACA TT---GGTTT T--G-GTAGT AAGCTATTCA TCTGATGGAT ATGCTACTCT CCATACCAAT ACT------- ----------
O. universa I          TCC------- ---AATG--A -A-ACA-TC- ---G-G-TCT G-------G- CCG--TTTTT AAC-AGTCCT GTACG-GTTT ---TGCAATG GCTACTGATA
O. universa III        TCG------- ---AAAGGAA ACATCGGTTT TAAGGTAACG TCGATTGCCT ACCGATAGC- ---------- ---------- ---------- ----------
G. sacculifer          CA-------- ---ACCG--A -A-ACA-TC- ---G-G-T-T G-------G- CTG--TTCTG ATTGACCCCT TCTGG-GTCT CTCTGTAACA -CTACCGATG
G. ruber pink          TTGT------ ---AAGGG-A TA-AGAGTGT ---G-G-TCA GT----ATG- CCG--TCGGG GATTCCCCCT G-ACG--TCT C-CTAGCACA -CGATT-ATC
G. ruber Ia            TT-------- ---AAGGG-A TA-AGAGTGT ---G-G-TCA GT----AGA- ACG--TCGGG -ATTTCCCCT G-ACG--TCT C-CCCCGCA- -CGATT-ATC
G. ruber Ib1           TT-------- --TAAGGG-A TA-AGCGTGC ---G-G-CCA GT----AGA- ACG--TCGGG AAATTCCCCT G--CG--TCG CC-TGCGCA- -CGATT-ATC
G. ruber Ib2           TT-------- --TAAGGG-A TA-AGCGTGC ---G-G-CCA GT----AGA- ACG--TCGGC -AATTGCCCT G-GCG--TCT TCTACCGCA- -CGATT-ATC
G. ruber IIa           AT-------- --AGAGTG-A TA-GGTTCCG TGGTTTGTAC ATTTTAAGGG CTCGTCCTAG TAACCAAAGC TCCACGGGAC AT-------- -------ATC
G. conglobatus         T--------- ---GAAGAGT GA-TA-GGTG CTGGGGTTGT ACATTTTAAA GGGCTCGTCC TAGTAACCAA CCGCCCCC-A AGCATCT--- -------ATC
G. rubescens (pink)    TATTA----- ---GACTGAT GAGTCCTTAA TTTTTACATT ACGTGAATCT CATTGACTT- ---------- ---------- ---------- -------ATC
G. bulloides Ia        GTT--TTCTC CA-AATAACG TATACAGTGG ACTTGGTGTC GGGTGC-TGG CCTCTGGTCA TGTGCTTTGA TTAC------ ---------- ----------
G. bulloides Ib        GTTAG--CTC C--ATTAACG TATTGAGTGG ACTTGGTGTC GGGT-C-TGG CCTCTGGTCA TGTGCTTTGA TTAC------ ---------- ----------
G. bulloides IIa       GTT--TTCTC C--AATAACG TATCTAGTGG ACTTGGTGTC GGGTGCGTGG CCTC-GGTCG TGTACTTTGA TTAC------ ---------- ----------
G. bulloides IIb       GTTAGTTCTC C--AATAACG TATCTAGTGG ACTTGGTGTC GGGTATGTGG CCTC-GGTCA TGTACTTTGA TTAC------ ---------- ----------
G. bulloides IIc       GCT--TTCTC C--AATAACG TATCTAGTGG ACTTGGTGTC GGGTGCGTGG CCTC-GGTCG TGTACTTTGA TTAC------ ---------- ----------
G. bulloides IId       GTTAGTTCTC C--AATAACG TATCTAGTGG ACTTGGTGTC GGGTGTGTGG CCTCTGGTCA TGCGCTTTGA TTAC------ ---------- ----------
G. bulloides IIe       GTT--TTCTC C--AATAACG TATCTAGTGG ACTTGGTGTC GGGTGC-TGA CCTCTGGTCT GGTACTTTGA TTAC------ ---------- ----------
T. quinqueloba Ia      GTAACAATA- ----ATTTTG AATGTATTGG TTAAGCTTTT -CTTATATTT TGGTAAGAGG TTAAT----- ---------- ---------- ----------
T. quinqueloba Ib      GTAACAATA- ----ATTTTG AATGTATTGG TTAAGCTTTT -CTTATATTT TGGTAAGAGG TTAAT----- ---------- ---------- ----------
T. quinqueloba IIa     GTAACATTG- ---AATAATG AATGTATTGG TTAAGCTATA TTGTATTTC- -GCTAA--CG TTAAT----- ---------- ---------- ----------
T. quinqueloba IIb     GTAACATTG- ----ATTTTG CATCTATTGG TTAAGCTATA ATGTATTTC- -GGTAA--CG TTAAT----- ---------- ---------- ----------
T. quinqueloba IIc     GTAACATTG- ----ATTATG CATCTATTGG TTAAGCTATA ATGTATTTC- -GGTAA--CG TTAAT----- ---------- ---------- ----------
T. quinqueloba IId     GTAACATTG- ----ATTATG CATCTATTGG TTAAGCTATA ATGTATTTC- -GGTAA--CG TTAAT----- ---------- ---------- ----------
G. falconensis         TTTTCTCCA- ----ATGACG AACCTAGTGG ACTTGGCGTC TGTGCGCATT CGTGTGCTAA TGACTGC--- ---------- ---------- ----------
H. pelagica            CTGACCC--- ---AATACTG GAATTTCTGG TGTCCACTGG ACACAGCACA CACAGTACCG CCC------- ---------- ---------- ----------
G. menardii            GTTGTGTCC- ---TTGAATG AATAATTTTC ATTAAGCGAA TATCGAATAA AAGGAACGTT TGACGAGAGT TACACGAAAG CCTTTATTAA ATTTCTTATT
G. ungulata            GTTGTGTCTC C--ATGGTGG AAATCATTTT CATTCAACGA TTATCGAAAG AA-GGAACGT ATGACGAGAG TTTTACGAAA GCCTTTGTTA AATTTCTTAT
G. hirsuta             ATTTATTAC- ---TAACA-- CCGC---TTT ATTAAAGCAT TCAGTGTGTA GTTGTGTAAG CAGCTGCCTC ---------- ---------- ----------
G. scitula             ATTTATCTT- ---TATAT-- AGAA---TTC ATTCTATATA AGAACCGTAT TTTCAAGCAT CTGTCGTTAT TACAA----- ---------- ----------
G. truncatulinoides    TATCTTCTT- ---ATACCTA ACCCAATATG TTAAACTTAT TGCGTAAGGG -TATTGTCTT AACGA---CT TATCCGCTTT AGCGCTTTAA GATTATTGGG
N. pachyderma I        AAT-ATAA-- ---T-ACA-- CCGT---CTT TAG-CGCTTA GA-CGCGGTT AT-TGGCTTA CTTTTTTTTA TTAA-CGAGT -GAGTTTAAT TAACCG---A
N. pachyderma II       AAT-ATAA-- ---T-ATA-- CCGT---CTT TAG-CGCTTA GA-CGCGATT AA-TGGATCG ---TTTTTTA TTAAGCGAGT -C-GTTTAAT ----CG---A
N. pachyderma III      AAT-ATAA-- ---T-ACA-- CCGT---CTT TAG-CGCTTA GA-CGCGATT AA-TGGATCG ---TTTTTTA TTAAGCGAGT -C-GTTTAAT ----CG---A
N. pachyderma IV       AAT-ATAA-- ---T-ACA-- CCGT---CTT TAG-CGCTTA GA-CGCGATT AT-TGGCTTT T---TTTTTA TTAAGAAAGT -C-TATTAAT ----CG----
N. pachyderma V        AAT-ATAA-- ---T-ACA-- CCGT---CTT TAG-CGCTTA GA-CGCGATT AA-TGGATTG C---TTCTTA TTAAGCGAGT -C-GTTTAAT ----CG---A
N. pachyderma VI       AAT-ATAA-- ---T-ACA-- CCGT---CTT TAG-CGCTTA GA-CGCGATT AA-TGGATTG C---TTTTTA TTAAGCGAGT -C-GTTTAAT ----CG---A
N. pachyderma VII      AAT-ATAA-- ---T-ACA-- CCGT---CTT TAG-CGCTTA GA-CGCGATT AT-CGGCTTA C---TTTTTA TTAAGGAGGT -CTGTTTAAT ----CG---A
N. dutertrei_C         AAT-ATTA-- ---T-ACA-- CCGT---ATT AAG-CGCTTA GT-TGCGATT AT-TGGCTCA TTAT------ --TGGGTCTT TTAATTGTAT T---------
N. dutertrei_Ib        AAT-ATTA-- ---T-ACA-- CCGT---ATT AAG-CGCTTA GT-TGCGATT AT-TGGCTCA TTAT------ --TGGGTCTT TTAATTGTAT T---------
P. obliquiloculata_BR  AATTTATA-- ---T-ACA-- CCGT---ATT TAG-CGCTAA GA-TATGATT AT-TGGCTCT TTT------- --AGGGTCTT TTAATTGTAT T---------
P. obliquiloculata_AS  AATTTTTA-- ---T-ACA-- CCGT---ATT TAG-CGCTTA GA-TGCGATT AT-TGGCTCT TTT------- --AGAGTCTT TTAATTGTAT T---------
G. inflata             AATTTTA--- ---TAACA-- CCGT---ATT AAG-CGCTTG GG-TCGTAAT TG-TTAGGCC TTTTAGGTTT TTTCAATTGC GTTTC----- ----------
G. crassaformis        AATTTTA--- ---TAACA-- CCGT---ATT AAG-CGCTTG GG-TCGTAAT TG-TTAGGCC TTTTAGGTTT TTTCAATTGC GTT-C----- ----------
N. incompta I          AATTTTTA-- ---CAACA-- CCGT---CAA CAC-ACGTAG TGAGCTGCTT GATCTCTCAT TCATGCATTC TGTGCTTCGG TGCAGTGTGC AATGTGAGTT
N. incompta II         AATTTTTA-- ---CAACA-- CCGT---TAA CAC-ACGTAG TGAGCCGCTT GAAATCTCAT CTGCATATGC ATATTTCGGT ATGGATT-GC AATGTGAGTT
G. glutinata Ia1       ATTTTTT--- ---ACCT-AA CACCGCACAC GTGAGT--GC ATAC--TTGT ATGTTA-CTT TACGCAGCGG TAAA------ ---------- ----------
G. glutinata Ia2       ATTTTTT--- ---ACCTTAA CACCGCACAC GTGAGTTTAC ATAC--TTGT ATGTCA-CTT TACGCAGCGG TAAA------ ---------- ----------
G. glutinata Ia3       ATTTTTT--- ---ACCTTAA CACCGCACAC GTGAGT--AC ATACyTTTGT ATGTCA-CTT TACGCAGCGG TAAA------ ---------- ----------
C. nitida              ATTCCTT--- ---ACCACAC CGCACACGTG AGTCTTTAAC GTTTTGTTAA TGCACTTTAC GCAGCGGTAA C--------- ---------- ----------
G. uvula               TTTGATTTAC T--TAACACC GCATACGTGA GTTCCAACTA GCTTAGCAAT AAGTCAAGTA GATCTCTACG CAGCGGTAAC A--------- ----------
B. variabilis          TTTTTT-ACT A--CACC-GC ATGCGCGAGT CTATTT--GT CTGT-TCTGC TTCGGCTA-A CTGCTC---- --AAATAC-G ATCTCTGCGT GCGGTAAA--
S. globigerus          TTTTTT-ACT A--CACC-GC ATGCGCGAGT CTATTT--GT CTGC-TCTGC TTCGGCTATG CTGCTC---- --AAATAC-G ATCTCTGCGC GCGGTAAA--
B. alata               TTTTATTACT A--CACC-GC ATGCGCGAGT CTATTT-ACT CTTGCATTTA AGCTTCGGCT GTTGTGTTT- --AAATAC-G ATCTTTGCGC GCGGTAAA--
G. vivans              TTTTTTATA- ---CACCGCC TTCACGAGGC CATTTATTCA CTGTCCTTCG GGCTTTGCTT TAAATGTGCA TCTCTGTGAG CGGTAAA--- ----------
C. porrectus           TTTTTTA--- ---CACATCG CATGCGCGAG TCCATTTATT CAGATATCTT TCATTTACTT TCGGGTGAAT GTAGTGTTTG CTTTAAATGT GTATCTCTGC
C. ovoidea             TTTACTTTAT ---ACATCGC TAGCGCGAGT CCGTTTATTC TGTTCATGCC GTGGTGTCAT ATGCTATGGC TGCAGCTTTA AACGTGTATC TCTGCGCGCG
G. opercularis         TATTTAC--- ---TTACATC ACTTGCATGC AGCACCTTAA CTTTTGTTAT GGCGTCTTTA TGCGTGATAA A--------- ---------- ----------
E. aculeatum           A--------- ---------- ---------- ---------- ---------- ---------- ---------- ---------- ---------- ----------
E. vitrea              TTTTTT---- ---ACACACC GCATACGCGA GTCCATTTAT TCACTCTCGG GTGCTTTAAA TGTGCATCTC TGCGAGCGGT AAA------- ----------
H. germanica           TTTGTTATA- ---CACTGCT TGTGCGTATG TGCATCCATA TATTTATATG TGTGTGTGTA TGTATTGCAC GCAGTAA--- ---------- ----------
P. mediterranensis     TTTATTAATA ---CATCGCA AGCGCGAGTC CGTTTATTCT ATGTATCAGT TCATGCTGTT CGTAGCTTTA AATGCTGTAT CTCTGCGCGC GATAAA----
S. fusiformis          TTTTTTA--- ---CACACCG CATTCACGAG TCCATTTATT CATTCCTTCG GGTTTGCTTT AAATGTGCAT CTCTGTGAGC GGTAAA---- ----------
V. fragilis            TTTTTA---- ---CACACCG CATTCGCGAG TCCATTTATT CATCTTCGGG TGCTTAAATG TGTATCTCT- ---------G CGAGCGGTAA A---------
A. pseudocassis        ATTGATACA- ---CCGTTTG TGTTGTTGAG CTATTTTTAA TAGTTTTTTA CGACACACGG TAAA------ ---------- ---------- ----------
Spiroplectammina sp.   TTTATT---- ---ATACACC GCATGCATGA GTCCAGTTTG CCTAAATTTT AGTCTTCGGG CGAAGGATTT ATGTTAAATT GTGTTACTTT ATGCGCGGTA
Textularia sp.         TTTTTAACA- -------CAC CGCTTGCGCG TGTCCGTAAT ATTTGTGCGT ATTATGCGTA TCTTATATTG CGTGTTAC-- ---------- ATTGCGCGCG
S. limosum             TTTTTTT--- ---TAAAAGT AGTATTTATT TTTTGTTTGA TGAATTTTTA ATTTTTTTAA AATTTTAAAT TCGTAGAAAA ATGTACACTT ACA-------
G. antarctica          TTTTTT---- ---ACACATC GCATGCGCGA GTCCATTTAT TCATCACTTC GGTGTTGCTT TAAATGTGCA CCTCTGCGCG CGATAAA--- ----------
D. aphelis             TTTTTA---- ---CACACCG CATGCGCGAG TCCATTTATT CACCTTCGGG TGTTTTAAAT GTGTATCTC- ---------- ---------- ----------
P. peruviana           TATATAT--- ---AAAATAT ATATTTTAAC TACGGTTATT ATATATATTA TTAA------ ---------- ---------- ---------- ----------
M. secans              TTTTTCATG- ---TAATATA TTTAACTATA TTTTTTAGTT TTATATATTT TACATTA--- ---------- ---------- ---------- ----------
Quinqueloculina sp.    TTATTATAT- ---AAAAATT ATTCATATTT TATTATGTTT AATTTTTTCA AA-------- ---------- ---------- ---------- ----------
N. haylinosphaira      CCAGTTCGT- -------GTG CACAGTTTTT CGTTACATTA ATACTTTAAA GGGTATATAT TATGCATGTG TTGGTGGCGT CTTTCGCTAG ATGCCCTGAT
M. fusca               TAATA----- ---CATTAAA ATTTCAAATT TTTTAATTTG TTATTTTAAT AAA------- ---------- ---------- ---------- ----------
T. alba                TTTCAAAAA- ---CGCTGTT ATTTATAGAT TTTTCGTAGC TTTCTTTTTT CTATATGGAC TTTATTGTCT GTGTGGAGAG GGGAAGGTTA TGGATATTTT
A. mexicana            TTTTTACAC- ---ACTTGCG CGGGAAGGAT ATGTTTAACA TTACTGTTAT TCATTGTTCT TACTCTGTGC GCGGTAAA-- ---------- ----------
A. triangularis        ACATTGTGA- ---TTTTAGT TTATTATGAT TATATATTTA TATATTATAA TGTATTTATT TATGTTATTT TATATTATAT TAATTGTTTA TTCTAAAAAT
A. rara                ACATTGTGA- ---TTTTAGA TTATTAAAAT AATTATATTA TGAAAAGTGT ATTTATTTAT ATTTTGATTT TTATATTATT TATAATTTAA AAATTTAATC
E. scabrum             TTTTAAT--- ---TTTACAC CGCATTTGCG CGCGCGTTGT ATATATTTTT TTATATACAT TTGCTGTGCT GCAAAAGCGG TAAAAA---- ----------
N. venosus             TTTTTAC--- ---ACACCGC ATGCGCGAGT CTATTTATTC ACCATCTTGT GTGTTTTAAA TATGTATCTC TGCGCGCGGT AAA------- ----------
B. marginata           TTTTTTAC-- ---ACACCGC ATACGCGAGA CCATTTATTC ACCTTCGGGT GCTTTAAATG TGTTTTCTCT GCGAGCGGTA AA-------- ----------
Trochammina sp.        TTTTATT--- ---ACACACA GCCT-GCGCG TGTCCAATTA TATACAATTT ATGTTGTATT AATAATTG-- --TGT-GC-- ---------- ATTGCGCGCT
Peneroplis sp.         TATAATACA- ---TAATATG TTTACATATT ATAATAA--- ---------- ---------- ---------- ---------- ---------- ----------
S. orbiculus           ACTTTAT--- ---AATATAT AATATTATAT TACA------ ---------- ---------- ---------- ---------- ---------- ----------
Allogromia sp.         ATAGGAT--- ---TTTTATA ATCCGCAGGA ATTTAAATAA ATATATAATT TTTTATATAT TTATCCTGTT TA-------- ---------- ----------
```

```
                         1301       1311       1321       1331       1341       1351       1361       1371       1381       1391      1400
                         |          |          |          |          |          |          |          |          |          |         |
G. siphonifera Ia1       TC-------- ---------- ---------- ---------- ---------- ---------- ---------- ---------G CC-TTGTCT- -GAAA-----
G. siphonifera Ia2       TC-------- ---------- ---------- ---------- ---------- ---------- ---------- ---------G CC-TTGTCT- -GAAA-----
G. siphonifera IIa1      CT-------- ---------- ---------- ---------- ---------- ---------- ---------- ---------A TC-CGGCTT- -GAGA-----
G. siphonifera IIa2      CT-------- ---------- ---------- ---------- ---------- ---------- ---------- ---------A TC-CGGCTT- -GAGA-----
G. siphonifera IIa3      CT-------- ---------- ---------- ---------- ---------- ---------- ---------- ---------A TC-CGGCTT- -GAGA-----
G. siphonifera IIa       CT-------- ---------- ---------- ---------- ---------- ---------- ---------- ---------A TC-CGGCTT- -GAGA-----
G. siphonifera IIb       CT-------- ---------- ---------- ---------- ---------- ---------- ---------- ---------G TC-CTGTCT- -GAGA-----
G. calida                ---------- ---------- ---------- ---------- ---------- ---------- ---------- ---------A TC-TAGTCT- -GAAA-----
O. universa I            GT-------- ---------- ---------- ---------- ---------- ---------- ---------- ---------G TC-TGGTGTT -CAAC-----
O. universa III          ---------- ---------- ---------- ---------- ---------- ---------- ---------- ---------A CC-TGGTCCC -CAGT-----
G. sacculifer            AT-------- ---------- ---------- ---------- ---------- ---------- ---------- ---------C CC-TCCTCT- -GAAA-----
G. ruber pink            A--------- ---------- ---------- ---------- ---------- ---------- ---------- ---------G CC-TTTCCT- -TAAC-----
G. ruber Ia              A--------- ---------- ---------- ---------- ---------- ---------- ---------- ---------G CC-TTCCCT- -TAAC-----
G. ruber Ib1             A--------- ---------- ---------- ---------- ---------- ---------- ---------- ---------G CC-TTCCCT- -TAAC-----
G. ruber Ib2             A--------- ---------- ---------- ---------- ---------- ---------- ---------- ---------G CC-TTCCCT- -TAAC-----
G. ruber IIa             A--------- ---------- ---------- ---------- ---------- ---------- ---------- ---------A CC-TTCCCC- -TTAC-----
G. conglobatus           A--------- ---------- ---------- ---------- ---------- ---------- ---------- ---------A CC-TTCCCT- -TTAC-----
G. rubescens (pink)      AA-------- ---------- ---------- ---------- ---------- ---------- ---------- ---------A CC-TGCTCT- -GAAA-----
G. bulloides Ia          ---------- ---------- ---------- ---------- ---------- ---------- ---------- ---------T GT-CACTTT- -AAAC-----
G. bulloides Ib          ---------- ---------- ---------- ---------- ---------- ---------- ---------- ---------T GAACACTTT- -AAAC-----
G. bulloides IIa         ---------- ---------- ---------- ---------- ---------- ---------- ---------- ---------T GT-CACTTT- -AAAC-----
G. bulloides IIb         ---------- ---------- ---------- ---------- ---------- ---------- ---------- ---------T GT-CACTTT- -AAAC-----
G. bulloides IIc         ---------- ---------- ---------- ---------- ---------- ---------- ---------- ---------T GT-CACTTT- -AAAC-----
G. bulloides IId         ---------- ---------- ---------- ---------- ---------- ---------- ---------- ---------T GT-CACTTT- -AAAC-----
G. bulloides IIe         ---------- ---------- ---------- ---------- ---------- ---------- ---------- ---------T GT-CACTTT- -AAAC-----
T. quinqueloba Ia        ---------- ---------- ---------- ---------- ---------- ---------- ---------- ---------A CA-GAACTT- -CGAG-----
T. quinqueloba Ib        ---------- ---------- ---------- ---------- ---------- ---------- ---------- ---------A CA-GAACTT- -CGAG-----
T. quinqueloba IIa       ---------- ---------- ---------- ---------- ---------- ---------- ---------- ---------T CA-GAACTT- -CGAG-----
T. quinqueloba IIb       ---------- ---------- ---------- ---------- ---------- ---------- ---------- ---------T CA-GAACTT- -CGAG-----
T. quinqueloba IIc       ---------- ---------- ---------- ---------- ---------- ---------- ---------- ---------T CA-GAACTT- -CGAG-----
T. quinqueloba IId       ---------- ---------- ---------- ---------- ---------- ---------- ---------- ---------T CA-GAACTT- -CGAG-----
G. falconensis           ---------- ---------- ---------- ---------- ---------- ---------- ---------- ---------C GT-CACTTT- -AAAC-----
H. pelagica              ---------- ---------- ---------- ---------- ---------- ---------- ---------- ---------G TC-GACCAT- -GAGA-----
G. menardii              CCAGTGAACT TGAACGATTC GATCTTTGAT GA-------- ---------- ---------- ---------- ---------T TG-AGTCTT- -TGAT-----
G. ungulata              TCCAGTGAAC ATGACTGATT GAATATCTTT T-----GATC TTTGAATGTT GTTGGTACAC ATTTTGCGAC ATTAC----- ---------- ----------
G. hirsuta               ---------- TGATAT---- GCGC--GGTA AC-------- ---------- ---------- ---------- ---------A CC-TGTCTC- -GAGA-----
G. scitula               ---------- TGATCAGTAT GCGC--GGTA AC-------- ---------- ---------- ---------- ---------G CC-TGTTTC- -GAGA-----
G. truncatulinoides      CGGTTATGCC TGCCTCGAGA GAGTGTACAG GGCAGCAAGA GTAATGATTC CCTCGTTT-- ---------- ---------T TA-CTACTA- -ATAC-----
N. pachyderma I          TGATT----- ---TCTAATG TGCGCGGTAA A--------- ---------- ---------- ---------- ---------G CC-TGCTTC- -GAGA-----
N. pachyderma II         TGTTT----- ---TCTAATG TGCGCGGTAA A--------- ---------- ---------- ---------- ---------G CC-TGCTTC- -GAGA-----
N. pachyderma III        TGTTT----- ---TCTAATG TGCGCGGTAA A--------- ---------- ---------- ---------- ---------G CC-TGCTTC- -GAGA-----
N. pachyderma IV         TGTTT----- ---TCTAATG TGCGCGGTAA A--------- ---------- ---------- ---------- ---------G CC-TGCTTC- -GAGA-----
N. pachyderma V          TGTTT----- ---TCTAATG TGCGCGGTAA A--------- ---------- ---------- ---------- ---------G CC-TGCTTC- -GAGA-----
N. pachyderma VI         TGTTT----- ---TTTAATG TGCGCGGTAA A--------- ---------- ---------- ---------- ---------G CC-TGCTTC- -GAGA-----
N. pachyderma VII        TGTTT----- ---TCTAATG TGCGCGGTAA A--------- ---------- ---------- ---------- ---------G CC-TGCTTC- -GAGA-----
N. dutertrei_C           ---------- ---TCTAATG CGCGCGGTAA A--------- ---------- ---------- ---------- ---------G CC-TGCTTC- -GAGA-----
N. dutertrei_Ib          ---------- ---TCTAATG CGCGCGGTAA A--------- ---------- ---------- ---------- ---------G CC-TGCTTC- -GAGA-----
P. obliquiloculata_BR    ---------- ---TCTTATG CGCGCGGTAA A--------- ---------- ---------- ---------- ---------G CC-TGCTTC- -GAGA-----
P. obliquiloculata_AS    ---------- ---TCTAATG CGCGCGGTAA A--------- ---------- ---------- ---------- ---------G CC-TGCTTC- -GAGA-----
G. inflata               ---------- ---TCCAATG CGCACGGTAA A--------- ---------- ---------- ---------- ---------G CC-TGCTTC- -GAGA-----
G. crassaformis          ---------- ---TCCAATG CACACGGTAA A--------- ---------- ---------- ---------- ---------G CC-TGCTTC- -GAGA-----
N. incompta I            G--------- ---TCTAATG CGCGCGGTTA T--------- ---------- ---------- ---------- ---------G CC-TGTACC- -GAGA-----
N. incompta II           A--------- ---TCTAATG CGCGCGGTTA T--------- ---------- ---------- ---------- ---------G CC-TGTACC- -GAGA-----
G. glutinata Ia1         ---------- ---------- ---------- ---------- ---------- ---------- ---------- ---------G CC-TGCTTC- -GAGA-----
G. glutinata Ia2         ---------- ---------- ---------- ---------- ---------- ---------- ---------- ---------G CC-TGCTTC- -GAGA-----
G. glutinata Ia3         ---------- ---------- ---------- ---------- ---------- ---------- ---------- ---------G CC-TGCTTC- -GAGA-----
C. nitida                ---------- ---------- ---------- ---------- ---------- ---------- ---------- ---------G CC-TGCTTC- -GAGA-----
G. uvula                 ---------- ---------- ---------- ---------- ---------- ---------- ---------- ---------G CC-TGCTTC- -GAGA-----
B. variabilis            ---------- ---------- ---------- ---------- ---------- ---------- ---------- ---------G CC-TACTTC- -GAAA-----
S. globigerus            ---------- ---------- ---------- ---------- ---------- ---------- ---------- ---------G CT-TACTTC- -GAAA-----
B. alata                 ---------- ---------- ---------- ---------- ---------- ---------- ---------- ---------G CC-TACTTC- -GAAA-----
G. vivans                ---------- ---------- ---------- ---------- ---------- ---------- ---------- ---------G CC-TGCTTC- -GACA-----
C. porrectus             GCGCGATAAA ---------- ---------- ---------- ---------- ---------- ---------- ---------G CC-TGCTTC- -GAAA-----
C. ovoidea               ATAAA----- ---------- ---------- ---------- ---------- ---------- ---------- ---------G CC-TACTTT- -GAAA-----
G. opercularis           ---------- ---------- ---------- ---------- ---------- ---------- ---------- ---------G CC-TGCTTC- -GAAA-----
E. aculeatum             ---------- ---------- ---------- ---------- ---------- ---------- ---------- ---------T CC-TACTCT- -GAGA-----
E. vitrea                ---------- ---------- ---------- ---------- ---------- ---------- ---------- ---------G CC-TGCTTC- -GAAA-----
H. germanica             ---------- ---------- ---------- ---------- ---------- ---------- ---------- ---------A GC-CTACTT- -CGAA-----
P. mediterranensis       ---------- ---------- ---------- ---------- ---------- ---------- ---------- ---------G CC-TGCTTT- -GAAA-----
S. fusiformis            ---------- ---------- ---------- ---------- ---------- ---------- ---------- ---------G CC-TGCTT-- -CGAA----A
V. fragilis              ---------- ---------- ---------- ---------- ---------- ---------- ---------- ---------G CC-TGCTTC- -GAGA-----
A. pseudocassis          ---------- ---------- ---------- ---------- ---------- ---------- ---------- ---------G CC-TGCTTC- -GAAA-----
Spiroplectammina sp.     AA-------- ---------- ---------- ---------- ---------- ---------- ---------- ---------G CC-TGCTTC- -GAAA-----
Textularia sp.           GTAAA----- ---------- ---------- ---------- ---------- ---------- ---------- ---------G CC-TACTTC- -GAAA-----
S. limosum               ---------- ---------- ---------- ---------- ---------- ---------- ---------- ---------A CC-TACTTC- -GAAA-----
G. antarctica            ---------- ---------- ---------- ---------- ---------- ---------- ---------- ---------G CC-TACTTC- -GAGA-----
D. aphelis               ---------- ---------- ---------- ---------- ---------- ---------- ---------- ---------T GC-GCGCGG- -TAAA-----
P. peruviana             ---------- ---------- ---------- ---------- ---------- ---------- ---------- ---------A CC-TATTTC- -GAAA-----
M. secans                ---------- ---------- ---------- ---------- ---------- ---------- ---------- ---------A CC-TATTTC- -GAAA-----
Quinqueloculina sp.      ---------- ---------- ---------- ---------- ---------- ---------- ---------- ---------A CC-TATTTC- -GAAA-----
N. haylinosphaira        ATGTGTGTGT GTTGCCTGTT AGTGTTAGCG GGCTGATGCA TTTTTACGAC TA-------- ---------- ---------G GC-TACTTC- -GAAA-----
M. fusca                 ---------- ---------- ---------- ---------- ---------- ---------- ---------- ---------G CC-TATTTC- -GAAA-----
T. alba                  ACAATCAGTA AATTCCAGCT TA-------- ---------- ---------- ---------- ---------- ---------G CC-TGCTTC- -GAAA-----
A. mexicana              ---------- ---------- ---------- ---------- ---------- ---------- ---------- ---------G CC-TGCTTC- -GACA-----
A. triangularis          TTAATCACAT A--------- ---------- ---------- ---------- ---------- ---------- ---------G CC-TACTTC- -GGCA-----
A. rara                  ACATA----- ---------- ---------- ---------- ---------- ---------- ---------- ---------G CC-TACTTC- -GGCA-----
E. scabrum               ---------- ---------- ---------- ---------- ---------- ---------- ---------- ---------G CC-TGCTTC- -GAAA-----
N. venosus               ---------- ---------- ---------- ---------- ---------- ---------- ---------- ---------G CC-TGCTTC- -GAAA-----
B. marginata             ---------- ---------- ---------- ---------- ---------- ---------- ---------- ---------G CC-TGCTTC- -GAAA-----
Trochammina sp.          GTAAA----- ---------- ---------- ---------- ---------- ---------- ---------- ---------G CC-TGCTTC- -GAAA-----
Peneroplis sp.           ---------- ---------- ---------- ---------- ---------- ---------- ---------- ---------A CC-TATTTC- -GAAA-----
S. orbiculus             ---------- ---------- ---------- ---------- ---------- ---------- ---------- ---------A CC-TATTTC- -GAAA-----
Allogromia sp.           ---------- ---------- ---------- ---------- ---------- ---------- ---------- ---------A CC-AACTTT- -GAAA-----
```

```
                           1401       1411       1421       1431       1441       1451       1461       1471       1481       1491      1500
                           |          |          |          |          |          |          |          |          |          |         |
G. siphonifera Ia1         GGAC------ -------T-A GG-TAATCTA TTGTAAGTGC TGGT----TC C--TCCT--- CCCGTT---G --AGCA--TT TTA---ATAA TGG--TC--T
G. siphonifera Ia2         GGAC------ -------T-A GG-TAATCTA TTGTAAGTGC TGGT----TC C--TCCT--- CCCGTT---G --AGCA--TT TTA---ATAA TGG--TC--T
G. siphonifera IIa1        AGGC------ -------T-G GG-TAATCAA TTGTAAGTGC TGGT----TC C--TCCT--- CCCGTT---G --AGCA--TT TTA---ATAA TGG--CC--T
G. siphonifera IIa2        AGGC------ -------T-G GG-TAATCAA TTGTAAGTGC TGGT----TC C--TCCT--- CCCGTT---G --AGCA--TT TTA---ATAA TGG--CC--T
G. siphonifera IIa3        AGGC------ -------T-G GG-TAATCAA TTGTAAGTGC TGGT----TC C--TCCT--- CCCGTT---G --AGCA--TT TTA---ATAA TGG--CC--T
G. siphonifera IIa         AGGC------ -------T-G GG-TAATCAA TTGTAAGTGC TGGT----TC C--TCCT--- CCCGTT---G --AGCA--TT TTA---ATAA TGG--CC--T
G. siphonifera IIb         AGGC------ -------T-G GG-TAATCAA TTGTAAGTGC TGGT----TC C--TACT--- CCCGTT---G --AGCA--TT TTA---ATAA TGG--CC--T
G. calida                  AGAC------ -------T-G GG-TAATCTA TTGTAAGTGC TGGT----TC C--TCTT--- CCCGTT---G --AGCA--TT TTA---ATAA TGG--TC--T
O. universa I              ACAC------ -------T-G GG-CAATCTC TTGAAAATAC TGAT----AG A--AT--ACC CA-ACTCG-- -AGCAAATCT ACTACACGCT GGAGGCACAA
O. universa III            GGAC------ -------T-G GG-CAATCTC TTCTAAATGC TGGT----AA G--GT--ACC CACACTCG-- -AGCTATTTA ATCACACGCG GGAGGCACAC
G. sacculifer              AGAG------ -------A-G GG-TAAGCCG TTCGAAATTC TGGT----AA C-GATT--CC CCGTAGTTA- -AGCAAACTT AAACCATAGT GGTGTCA-AA
G. ruber pink              AGGG------ -------C-G GG-TAACCTT TTCAAAATAC CAGT----TT G--ATTTT-A -----A--AG C---TTTGGG TCA-ATTAG- GGTG-AT---
G. ruber Ia                AGGG------ -------C-G GG-CAACCTT TTCAAAATAC TGGT----TT G--ATGTT-A -----A--AG T---ATTGAG TCG-TTTTTT TTGGAT----
G. ruber Ib1               AGGG------ -------C-G GG-CAACCTT TTCAAAATAC CGGT----TT G--ATATT-A -----A--AG T---ATAGAG TCG-TTTTTT TTGGAT----
G. ruber Ib2               AGGG------ -------C-G GG-CAACCTT TTCAAAATAC CGGT----TT G--ATGTT-A -----A--AG T---ATAGAG TCG-TTTTTT TTGGAT----
G. ruber IIa               AGGG------ -------C-G GG-TAACCTC TTCGAAGTGC TGGC----AT G--ATTTCCC TAGTTTTGGC ATTTGAATCC TTGTCGCAGC AGCCCTGAGA
G. conglobatus             AGGG------ -------T-G GG-TAACCTC TTCGAAGTGC TGGT----TT G--ATTTCCC -TAGTGTTTG TCATAATAAT CCTCGTCGCA CCTACTCTAG
G. rubescens (pink)        GGAG------ -------C-G GG-TAATCTT TTCGAAGTTC TAGT----TT G--ATTT--T CCTGTGGCGA ACGTTTTGAG TCCATGTTCC GTGTGTTTGC
G. bulloides Ia            ACTG------ ------GT-C GT-TAGACTC GTGCAA-GCA ATTC----A- G-AGC-AACG AATTG---CA AAC------A CTCTTTG--- ----------
G. bulloides Ib            ACTG------ ------GT-C GT-TAGACTT TTAGT--GCA ATCCAT--TT GCAGC-ATCG AATCGCGAAA ATGA-----G CATTGTTT-- ----------
G. bulloides IIa           ACTG------ ------GT-C GT-TAGACTC GTGCAATCCA ATAG----AA G-AGGTGACT AAGTTTCCCA --GT-----A CCGTGTT--- ----------
G. bulloides IIb           ACTG------ ------GT-C GT-TAGACTC GTGCAATCCA ATAG----AA G-AGGTGAAT AAGTTTCCCA AAGT-----A CCGTGTTT-- ----------
G. bulloides IIc           ACTG------ ------GT-C GT-TAGACTC GTGCAATCCA ATAG----AA G-AGGTGACT CAGTATCCCA --GT-----A CCGTGTT--- ----------
G. bulloides IId           ACTG------ ------GT-C GT-TAGACTC GTGCAATCCA ATAG----AA G-AGGTGAAT AAGTTTCCCA AAGT-----A CCGTGTTT-- ----------
G. bulloides IIe           ACTG------ ------GT-C GT-TAGACTC GTGCAATCCA TTTG----AA G-AGGTGACT CGTGTTTTGT AGT------A CCGTGTTG-- ----------
T. quinqueloba Ia          AGAG------ ------TT-C CG-ACAACAG TCAATCTAGT TATT----GC TTGTAGTCG- ---ATCTCAT GATTGTTTAG TTT--TATGC AAC-TTCGGG
T. quinqueloba Ib          AGAG------ ------TT-C CG-ACAACAG TCAATCTAGT TATT----GC TTGTAGTCG- ---ATCTCAT GATTGTTTAG TTT--TATGC AAC-TTCGGG
T. quinqueloba IIa         AAGG------ ------TT-C CG-ACAACAG TCAATCTAGT TATT----GC TTGTAGTCG- ---ATTGCAT AATTGTACAG TT---CACGC AACGCTCGG-
T. quinqueloba IIb         AAGG------ ------TT-C CG-ACAACAG TCAATCTAGT TATT----GC TTGTAGTTGT TA-ATTGTAT AATTGTCTAG TT---CACGC AACGTTCGT-
T. quinqueloba IIc         AAGG------ ------TT-C CG-ACAACAG TCAATCTAGT TATT----GC TTGTAGTTGT TA-ATTGTAT AATTGTCTAG TT---CACGC AACGTTCGT-
T. quinqueloba IId         AAGG------ ------TT-C CG-ACAACAG TCAATCTAGT TATT----GC TTGTAGTTGT TA-ATTGTAT AATTGTTTAG TT---CACGC AACGTTCGT-
G. falconensis             ACTG------ ------GT-C GT-TAGACTC GTGCGATCCC TCGG----AA AACGTGATTT CTGTTCCCAA ACAAAGCACT TTCGTACCCT CGCTCAAACA
H. pelagica                GAGG------ -------T-C GA-AAATCCA TTTGAAGTGT TGAT----AT GAGGTCAAGG CAAATTGCAC CACACACAGA TGGCTCTTTG TGTACTCCAT
G. menardii                AGTT------ ------GT-T GG-TACACAT CTTGTGACAT TGCT----TC C--CTAT--- CGCTATCCTA TGCTGTAGTG CGTTTGCAAT GCTGATGTTT
G. ungulata                ---------- ---------- ---------- ---------- --------TT CCCTATTGA- TCTCTGGATT GTAGTGCGTT TGTAATGCTG ATACTTTAAA
G. hirsuta                 GTCG------ ------AT-G GG-TAATCCA TTAGAAGTAA TGAT----TC CTCTCTTTTA TATAGCACAC TGATATACAG CATCGGTTCC CGAGGTAACC
G. scitula                 GTAA------ ------AT-G GG-TAATCCA TTGGAAGTAA TGAT----TT CTCTATTTTA TAATAAAATG CACGGTTTAT ATACGGCATT CATTCCCGAG
G. truncatulinoides        CGCG------ ------CT-T GG-TAACATA TTACACTCGA TTAA----GT AGGGTCTTTG CTTCTAATAG CAATTGCTAT CCTCACCCTT AGGCTCT-AA
N. pachyderma I            GTAA------ ------GC-G GG-TAATCCA TTGGAAGTAA TGAT----TT CTC-TATATC TAGT-----G CACAACTATG TACGG--CAT TCATTCCCGA
N. pachyderma II           GTAA------ ------GC-G GG-TAATCCA TTGGAAGTAA TGAT----TT CTC-TATATC TAGT-----G CACAACTATG CACGG--CAC TCATTCCCGA
N. pachyderma III          GTAA------ ------GC-G GG-TAATCCA TTGGAAGTAA TGAT----TT CTC-TATATC TAGT-----G CACAACTATG CACGG--CAC TCATTCCCGA
N. pachyderma IV           GTAA------ ------GC-G GG-TAATCCA TTGGAAGTAA TGAT----TT CTC-TATATC TAGT-----G CACAACTATG CACGG--CAT TCATTCCCGA
N. pachyderma V            GTAA------ ------GC-G GG-TAATCCA TTGGAAGTAA TGAT----TT CTC-TATATC TAGT-----G CACAACTATG CACGG--CAC TCATTCCCGA
N. pachyderma VI           GTAA------ ------GC-G GG-TAATCCA TTGGAAGTAA TGAT----TT CTC-TATATC TAGT-----G CACAACTATG CACGG--CAC TCATTCCCGA
N. pachyderma VII          GTAA------ ------GC-G GG-TAATCCA TTGGAAGTAA TGAT----TT CTC-TATATC TAGT-----G CACAACTATG CACGG--CAC TCATTCCCGA
N. dutertrei_C             GTAA------ ------GT-G GG-TAATCCA TTGGAAGTAA TGAT----TT CTC-TTTTTA TA-------G CACACCTATA TACGG--CAT TCATTCCCGG
N. dutertrei_Ib            GTAA------ ------GT-G GG-TAATCCA TTGGAAGTAA TGAT----TT CTCCTTTTTA ATA------G CACACCTATA TACGG--CAT TCATTCCCGG
P. obliquiloculata_BR      GTAA------ ------GT-G GG-TAATCCA TTGGAAGTAA TGAT----TT CTC-TTTTA- TATA-----G CACACCTATA TACGG--CAT TCATCCCCGG
P. obliquiloculata_AS      GTAA------ ------GT-G GG-TAATCCA TTGGAAGTAA TGAT----TT CTC-TTT-AT TATA-----G CACACCTATA TACGG--CAT TCATTCCCGG
G. inflata                 GTAA------ ------GT-G GG-TAATCCA TTGGAAGTAA TGAT----TT CCTTATTTTT ATAAACA--G CACACCAATA TACGG--CAT TCATTCCCGA
G. crassaformis            GTAA------ ------GT-G GG-TAATCCA TTGGAAGTAA TGAT----TT CCTTATTTTT ATAAACA--G CACACCAATA TACGG--CAT TCATTCCCGA
N. incompta I              GGTG------ ----ACGTCG GG-TAAACAT TCTCTAATGC TGAC----CT ATTTTTCTGA AATAT----G CACAACTCTA ATATGA-CAT TCATTCCCGG
N. incompta II             GGTG------ ----ACGTCG GG-TAACCAT TCTCTAATGC TGAC----CT ATTTTTCTGA AATAT----G CACAACTCTA ATATGA-CAT TCATTCCCGG
G. glutinata Ia1           GCAA------ ------GT-G GG-TAATCAA TTAGAAGTAA CGAT----TT CCCAAATTAG CACACTTATA TGCGGCGTTT GCGCCCGGGT ACCACTTGTT
G. glutinata Ia2           GCAA------ ------GT-G GG-TAATCAA TTAGAAGTAA CGAT----TT CCCAAATTAG CACACTTATT TACGGCGTTT GTGCCCGGGT TTCACTTGTT
G. glutinata Ia3           GCAA------ ------GT-G GG-TAATCAA TTAGAAGTAA CGAT----TT CCCAAATTAG CACACTTATT TACGGCGTTT GTGCCCGGGT TTCACTTGTT
C. nitida                  GCAA------ ------GT-G GG-TAATCAA TTAGAAGTAA CGAT----TT CCCAAGTTAG CACACTTATA TACGGCGTTT ATGCCCGGGT TTCACT-CGT
G. uvula                   GCAA------ ------GT-G GG-TAATCAA TTAGAAGTAA CGAT----TT CTTCGCATTA GCACACTTAA TATAAGGCGT TGACGCTCAT GATTCG-CTC
B. variabilis              GTAA------ ------GC-G GG-TAATCAA TTAGAAGTAA TGAT----TT CCT-ATTTT- -CTCT----- ---------- -------GCA CACATATATG
S. globigerus              GTAA------ ------GC-G GG-TAATCAA TTAGAAGTAA TGAT----TT CCT-ATTTT- -CTCT----- ---------- -------GCA CACATATATG
B. alata                   GTAA------ ------GC-G GG-TAATCAA TTAGAAGTAA TGAT----TT CCT-ATTTTT TCT------- ---------- -------GCA CACATATATG
G. vivans                  GTAA------ ------GT-G GG-TAATCAA TTAGAAGTAA TGAT----TT CCTTTTTTTC CAGCACACAT ATATACGGCG TTTATACCCG GGCTATCCTT
C. porrectus               GTAA------ ------GT-G GG-TAATCAA TTAGAAGTAA TGAT----TT CCTTTTTTTT TCTGCACACA TATATATGGC ACTTATACCC GGGTAACCTT
C. ovoidea                 GTTTA----- ------GT-G GG-TAATCAA TTAGAAGTAA TGAT----TT CCAAGTTATA GCACACTGAT ATACGGCATC TTTACCCAGC TAGCCTTTTG
G. opercularis             GTAA------ ------GT-G GG-TAATCAA TTAGAAGTAA TGAT----TT CCTTTATATG CACATTTATG TTTGGCACTG ATCCCCAGCC TAACTCTTGT
E. aculeatum               ATA------- ------GT-T GG-TAATC-- --AGAAGTAA TGAT----CT CTCTCGTATA CGCATACATG TATCGTATAT ATTAATATAT ATTAATTTAT
E. vitrea                  GTAA------ ------GT-G GG-TAATCAA TTAGAAGTAA TGAT----TT CCTTTTTTTC TAGCACACAT ATATACGGCG TTTATACCCG GGCTGCCTTG
H. germanica               AGTT------ ------GT-G GG-TAATCAA TTTGAAGTAA TGAT----TT CTCCAAATAT ATATACTGCA CACTCATATA CAGTGTCTTA TGTCCATGAA
P. mediterranensis         GTTTA----- ------GT-G GG-TAATCAA TTAGAAGTAA TGAT----TT CCTTAATTTA TCGCACTTAT ATATGCGGCG TCTTTACCCG GCTGGCCTTT
S. fusiformis              GTAA------ ------GT-G GG-TAATCAA TTAGGAGTAA TGAT----TT CCTTTTTTTT CAGCACACAT ATATACGGCG TTTATACCCG GGCTGTCCTT
V. fragilis                GTAA------ ------GT-G GG-TAATCAA TTAGAAGTAA TGAT----TT CCTTTTTTAT AGCACACATA TATACAGCAT TTATGCCCGG GTTGGCCTTG
A. pseudocassis            GTAA------ ------GT-G GG-TAATCAA TTAGAAGTAA TGAT----TT CCCTTTTTAG CACACCTATG CACGGCTGTT GCGTCCGGTC TAAGTTTACT
Spiroplectammina sp.       GTAA------ ------GC-G GG-TAATCAA TTAGAAGTAA TGAT----TT CCC-AATTTA TA-------G CACACATATA TACGG--CAT TTTTACCCGG
Textularia sp.             GTAA------ ------GT-G GG-TAATCAA TTAGAAGTAA TGAT----TT CCTTTATATA GCACACATAT ATGCGGCATC TTTACCCGGG TTAAGCTGTC
S. limosum                 GTCA------ ------GT-A GG-TAATCAG TTCGAAGTAA TGAT----TT CCTCTTTTTA TTTATATGAA TTTTAATTTT ATATATTTTT AATTGACGCA
G. antarctica              GCAA------ ------GT-G GG-TAATCAA TTAGAAGTAA TGAT----TT CCTTTTTTTT CTGCACGCAT ATATATGGCA CTTATACCCG GGTAGCCTTG
D. aphelis                 GCCT------ ------GC-T TC-GACAGTA AGTGGGTAAT CAATTAGAAG TAATGATTTC CTTTTTTTTA GCACACATAT ATACGGCGTC TATGCCCGGG
P. peruviana               GTGA------ ------AT-G GG-TAATCAT TTAAAAATCG TGGT----TA ACATTTTATA CAACAATTTT ATATATTGTG TAATGTTTAT TATATACTTT
M. secans                  GTGA------ ------AT-G GG-TAATCAT TTGAAAATCG TGAT----TA TTTCAATATA CTACATTTTT AAGTTATAAA TTTTTAATTA TAGTTTACTA
Quinqueloculina sp.        GTGA------ ------AT-G GG-TAATCAT TTAAAAATCG TGAT----TA TTTCATTTAT ACTACATTTT ATAGTTATAA ATTTATTAAT ATACTTTATT
N. haylinosphaira          GTAA------ ------GT-A GC-TAATCAA TTCGAAGTAA TGAT----TT CCTTTGCATA TTTTATATAT GTCCTGTATT TTATGGGTGT TTTCTCTTTT
M. fusca                   GTAA------ ------AT-A GG-TAATCAA TTAAAAGTCG TGAT----TT CCATACAACA CATTTAAAAA GGAATTATTG TTTATAGTTT AAATTTATTT
T. alba                    GTTA------ ------GC-A GG-TAATCAC TTGGAAGTAA TGAT----TT CCCTTATATC AAAATTTATT TTTTGGTTTT GCGAGCACAC AATGTGCTGC
A. mexicana                GTAA------ ------GT-G GG-TAATCAA TTAGAAGTAA TGAT----TT CCTTATTTAG CACAATTATA TACGGCGTTT ATGACCGGGT TATCCTTTGT
A. triangularis            GTCA------ ------GT-A GG-TAATCAA TTAGAAGTAA TGAT----TT CCCCTTTTTT ATTTAAATGT ATTTCAAA-T TTATTTTTTT AATATGTTTA
A. rara                    GTCA------ ------GT-A GG-TAATCAA TTAGAAGTAA TGAT----TT CCCCTTTTTT ATTTAAATAT AATATAAAAT TTATTTTTTA TTTGTGTTTA
E. scabrum                 GTAA------ ------GT-G GG-TAATCAA TTAGAAGTAA TGAT----TT CCCTAAATTT TTTTTAATAG CACACATATA TACGGCATCT TTACCCGTTA
N. venosus                 GTAA------ ------GT-G GG-TAATCAA TTAGAAGTAA TGAT----TT CCTTATATAG CACACATATA TACGGCATCT TTACCCGGCC TGCCTTGTTG
B. marginata               GTAA------ ------GT-G GG-TAATCAA TTAGAAGTAA TGAT----TT CCTTTTTTTA GCACACATAT ATACGGCGTC TATGCCCGGG TTACCTTGTT
Trochammina sp.            GTAA------ ------GT-G GG-TAATCAA TTAGAAGTAA TGAT----TT CCTTTATTAA TTTATTAGT- --GCACAATT AT---ATACG GCATCTTTAC
Peneroplis sp.             GTAA------ ------AT-G GG-TAATCAT TTAAAAATCG TGAT----TA TTTCATACAT ATGGTATATT AATATTGTAT AATACTATAC ATTTTATAGT
S. orbiculus               GTAA------ ------AT-C GG-TAATCAT TTAAAAATCG TGAT----TA ATTAA----- ---------- ---------- ---------- ----------
Allogromia sp.             GTAA------ ------GT-T GG-TAATCAA TTCGAAGTAA TGAT----TT CCTTTT---- ---------- --GCACAATA AT---AATAT TTTATTCGAT
```

```
                      1501       1511       1521       1531       1541       1551       1561       1571       1581       1591      1600
                      |          |          |          |          |          |          |          |          |          |         |
G. siphonifera Ia1    CTCTACATCC CTAGCACATG ATG---TC-- TAGTGCGATT G---TAGTTG AGTCTTG--C CATT------ ---------- ---------- ----------
G. siphonifera Ia2    CTCTACATCC CTAGCACATG ATG---TC-- TAGTGCGATT G---TAGTTG AGTTCTG--C CATT------ ---------- ---------- ----------
G. siphonifera IIa1   CTCTACATCC CTAGTA-AT- ATG---AC-- TAGTGCGATT G---TAGTTG AGCCTTG--C CATT------ ---------- ---------- ----------
G. siphonifera IIa2   CTCTACATCC CTAGCA-AT- ATG---CC-- TAGTGCGATT G---TAGTTG AGCCTTG--C CATT------ ---------- ---------- ----------
G. siphonifera IIa3   CTCTACATCC CTAGCA-AT- ATG---CC-- TAGTGCGATT G---TAGTTG AGCCTTG--C CATT------ ---------- ---------- ----------
G. siphonifera IIa    CTCTACATCC CTAGTA-AT- ATG---CC-- TAGTGCGATT G---TAGTTG AGCCTTG--C CATT------ ---------- ---------- ----------
G. siphonifera IIb    CTCTACATCC CTAGTA-AC- ------CC-- TAGTGCGATT G---TAGTTG AGCCTTG--C CATT------ ---------- ---------- ----------
G. calida             CTCTACGTCC CTAGTA--T- AT----TC-- TAGTGCGATT G---TAGTTG AGTCT-G--C CATT------ ---------- ---------- ----------
O. universa I         -CGG------ -TCCAAGGGC TTA--TCCCT TGCGA----- -C--TGGTGC AGCCAATG-- -ACG------ ---------- ---------- ----------
O. universa III       ACGG------ -TTCAAATCT CATTTGCGAC TGTTGCCGCC ATGA------ ---------- ---------- ---------- ---------- ----------
G. sacculifer         -CGGGTCCGC TTCCATCGGA AAAGATTCCT CCGGAAAAAG GC--TTATGC AGGCATT-TC -ACG------ ---------- ---------- ----------
G. ruber pink         GGTTCGATAG -AACCAATAT CA-CCCAGCT GCTA------ CTCGTGCGCG CGTG-T---- ---------- ---------- ---------- ----------
G. ruber Ia           GGTGGTTTGT TAACCAATAC CATCCGGCAA CTGC------ -TCGCGAGTT TGTGGTT--- ---------- ---------- ---------- ----------
G. ruber Ib1          GGTGGTTTAC GAACCAATAC CATCCG-CAA CTGC------ -TCGCGAGTT TGTGGTT--- ---------- ---------- ---------- ----------
G. ruber Ib2          GGTGGTTTAC GAACCAATAC CATCCG-CAA CTGC------ -TCGCGAGTT TGTGGTT--- ---------- ---------- ---------- ----------
G. ruber IIa          CGTAGTGAAC GCTGCAAAGT GATTCCAGTC TTAGGTTTGC ---------- ---------- ---------- ---------- ---------- ----------
G. conglobatus        GACATAGTGA ACATTGTAGA CTTTGTCATA ATGATTCCCG TCTTAGGTTT GCGACGCATG CTTT------ ---------- ---------- ----------
G. rubescens (pink)   GGTGTCCTCT GTCAGTTTCA TGTATTGTG- ---------- ---------- ---------- ---------- ---------- ---------- ----------
G. bulloides Ia       -------GGC --ACTGTTT- -----ACT-- --CCC----- -GTTTACAGA GGAGAGATAG AC-------- ---------- ---TAA-CCA CCTATTCTCG
G. bulloides Ib       -------ACA --CCCGTTCT ----AGAGCA GTGGTTATTC ---------- ---------- ---------- ---------- ---TAA-CCA TATTTCCACT
G. bulloides IIa      -------CGC CTCCCGTGTC AGT-AGGG-- -CCCC----- ----TCGCAT ATA------- ---------- ---------- -------CCA GTCGAAGTGG
G. bulloides IIb      -------AGC -TCCCGTGTT CGT-AGCGA- -CCCC----- ----TCGCAA ATG------- ---------- ---------- -------CCA GTCGATGTGG
G. bulloides IIc      -------AGT CTCCCGTGTT CGT-AGTGG- -CCCC----- ----GCGCA- TTG------- ---------- ---------- -------CCA GTCGTAGTGG
G. bulloides IId      -------AGC -TCCCGTGTT CGT-AGAGA- -CCCC----- ----TCGCA- TTG------- ---------- ---------- -------CCA GTCGATGTGG
G. bulloides IIe      -------ATC -TCCCGTTGT AGT-AGAGA- -CCCC----- ----TCGCA- TTA------- ---------- ---------- -------CCA GTCGATGTGG
T. quinqueloba Ia     TCATGATT-- ---------- ---------- ---------- ---------- ---------- ---------- ---------- ---------- ----------
T. quinqueloba Ib     TCATGATT-- ---------- ---------- ---------- ---------- ---------- ---------- ---------- ---------- ----------
T. quinqueloba IIa    TTATCATT-- ---------- ---------- ---------- ---------- ---------- ---------- ---------- ---------- ----------
T. quinqueloba IIb    TTATCATT-- ---------- ---------- ---------- ---------- ---------- ---------- ---------- ---------- ----------
T. quinqueloba IIc    TTATCATT-- ---------- ---------- ---------- ---------- ---------- ---------- ---------- ---------- ----------
T. quinqueloba IId    TTATCATT-- ---------- ---------- ---------- ---------- ---------- ---------- ---------- ---------- ----------
G. falconensis        AACTTTGAAA CTCCTGGTAA CTTTTTGGAT TCTTTTCTTG TCATCTTTTC GTGTCATCTT T--------- ---------- ---------- ----------
H. pelagica           TATTCACTGA GTAAGTGCGA GTCACAAAGG GGCATT---- ---------- ---------- ---------- ---------- ---------- ----------
G. menardii           TAAACGCCCC TCATGTCTCG AGCGTTCCTC GATCCATTGT GCAAGTCAGG CGATCAGGTT TCG------- ---------- ---------- ----------
G. ungulata           CGCCTATCCT TTTGGTTAAG CGATCAGATA TCG------- ---------- ---------- ---------- ---------- ---------- ----------
G. hirsuta            TTGTGTTACT TATGTGCGAG CGTGATGTCA GA-GCTTAAC GTTTATTATT CTTTATGTCT AATAGATCTC TGATTTTACT G--------- ----------
G. scitula            GCGCCTTTGT AGTGTCTATG TGCGAATGTA ATGTTATGGC TTTGTTATTA ACGGCTATTC CTTAATAGTT TTGTTATTAA CGGCTATAGT TATCG-----
G. truncatulinoides   GGATATTACT ATCTCTCTAA GGTCTACAGG GAGGTGTATA ---------- ---------- ---------- ---------- ---------- ----------
N. pachyderma I       GACGGCT-AG TTCCGTCTTT T-A-GTGCGA AT-GTAGTG- TTATTCAAAC G--------- ---------- ---------- ---------- ----------
N. pachyderma II      GACGGCT-AG TTTCGTCTTT T-A-GTGTGA AT-GTAGTG- TTATTCAAAC G--------- ---------- ---------- ---------- ----------
N. pachyderma III     GACGGCT-AG TTTCGTCTTT T-A-GTGTGA AT-GTAGTG- TTATTCAAAC G--------- ---------- ---------- ---------- ----------
N. pachyderma IV      GACGGCT-AG TTTCGTCTTT T-A-GTGCGA AT-GTAATG- TTATTCAAAC G--------- ---------- ---------- ---------- ----------
N. pachyderma V       GACGGCT-AG TTTCGTCTTT T-A-GTGTGA AT-GTATGTG TTATTCAAAC G--------- ---------- ---------- ---------- ----------
N. pachyderma VI      GACGGCT-AG TTTCGTCTTT T-A-GTGTGA AT-GTAGTG- TTATTCAAAC G--------- ---------- ---------- ---------- ----------
N. pachyderma VII     GACGGCT-AG TTTCGTCTTT T-A-GTGTGA AT-GTGGTG- TTATTCAAAC G--------- ---------- ---------- ---------- ----------
N. dutertrei_C        GACGACT-AG TTTCGTCTTT TTT-GTGCGA AT-GTAATG- -TATTCTTTA TCCG------ ---------- ---------- ---------- ----------
N. dutertrei_Ib       GACGACT-AG TTTCGTCTTT TTTTGTGCGA AT-GTAATG- -TATTCTTTA TCCG------ ---------- ---------- ---------- ----------
P. obliquiloculata_BR GATGACT-AG TTTCGTCTTT TTT-GTGCGA AT-GTAATG- -TATTTCTTA TCCG------ ---------- ---------- ---------- ----------
P. obliquiloculata_AS GATGACT-AG TTTCGTCTTT TTT-GTGCGA AT-GTAATG- -TATTCTTTA TCCG------ ---------- ---------- ---------- ----------
G. inflata            GACGGCTTAG TTCCGACTTT ----GTGCGA AT-GTAATG- -TATT-CTTA TCCG------ ---------- ---------- ---------- ----------
G. crassaformis       GACGGCTTAG TTCCGACTTT ----GTGCGA AT-GTAATG- -TATT-CTTA TCCG------ ---------- ---------- ---------- ----------
N. incompta I         GAGGACT-AG TTGCCTCTTT ----GTGTGA GT-GTAATG- -CAAACATGA ATAGCGACTG TCGCTGTTTA CTCA------ ---------- ----------
N. incompta II        GAGGACT-AG TTGCCTCTTT ----GTGTGA GT-GCAATG- -CAAACATGT CGGCGTAAGA CTTCATTCA- ---------- ---------- ----------
G. glutinata Ia1      GGTACTTCTG TGCGTGCAGA TGTTTTTT-- -CCG------ ---------- ---------- ---------- ---------- ---------- ----------
G. glutinata Ia2      GGAACTTTTG TGCGTGCAGA TGTTTTTTTT -CCG------ ---------- ---------- ---------- ---------- ---------- ----------
G. glutinata Ia3      GGAACTTTTG TGCGTGCAGA TGTTTTTTTT -CCG------ ---------- ---------- ---------- ---------- ---------- ----------
C. nitida             TGATTCCTTT GTGCGCGTAG ATGGATTCTT -CCG------ ---------- ---------- ---------- ---------- ---------- ----------
G. uvula              TCACGAGT-G TTTCATTGCA CGTGTTGATG TGCGGGTTCG ATTCCTGCTT TAC------- ---------- ---------- ---------- ----------
B. variabilis         CGG--CATAT -CTACCCGGC ATGCCT-TGT TGCATGTTCT TT-GTGCGTA GATGTGTACC CCTTTTTCCG ---------- ---------- ----------
S. globigerus         CGG--CACAT -CTACCCGGC ATGCCT-TGT TGCATGTTCT TT-GTGCGTA GATGTGTACC CCTTTTTCCG ---------- ---------- ----------
B. alata              TGG--CGCAT -TTACCCGGC ATGCCT-TGT TGCATGTTAT TT-GTGCGTA GATGTGTAC- ---TTTTCCA ---------- ---------- ----------
G. vivans             GTTGGTAGCT TTTGTGTGTA TAGATGTTTT TTCCGTA--- ---------- ---------- ---------- ---------- ---------- ----------
C. porrectus          GTTGTTACTT TTGTGCGTAT ACGTGATAAT CTTTACCGAT TATACTACCA T--------- ---------- ---------- ---------- ----------
C. ovoidea            TGCTAGTCTT TTGTGTGTAG AGATGAAACA ACTTGTCTTA TCATTGACTC ACACTTTTTT GGTGTGTTCA GTGTATGAGT TTAGTTTGTT TATTCTTACC
G. opercularis        TAGTGCTTGT GTGCGTTCAG TGGACCTTTT ACGGTCTCTA ACCAT----- ---------- ---------- ---------- ---------- ----------
E. aculeatum          ACGTA----- ---------- ---------- ---------- ---------- ---------- ---------- ---------- ---------- ----------
E. vitrea             TTGTAGCTTT TGTGCGTATA GATGTTTTTT CCG------- ---------- ---------- ---------- ---------- ---------- ----------
H. germanica          AATTTTATTA TTTTTGTGTG TGCATTCGAC GCTCG----- ---------- ---------- ---------- ---------- ---------- ----------
P. mediterranensis    TGTGCCAGAT TCTTGTGTGT ATTGACGATT GCAAACATTA TGTTTCAATT GCCTATTTAC GGTACCAC-- ---------- ---------- ----------
S. fusiformis         GTTGATAGCT TTTGTGTGTA TAGATGTTTT TTCCG----- ---------- ---------- ---------- ---------- ---------- ----------
V. fragilis           TTGTCAGCTT CTGTGTGTAT AGATGTATAT CTCAATTAGA TTCTTTATGT CTCTTTTTAG ATTCTG---- ---------- ---------- ----------
A. pseudocassis       TAGGCTCGCG TACGTAGCAG TTTTTTCG-- ---------- ---------- ---------- ---------- ---------- ---------- ----------
Spiroplectammina sp.  ATTAAGCTTG TCTTAATTTT T---GTGTGT AATAATGTTT TTTCCG---- ---------- ---------- ---------- ---------- ----------
Textularia sp.        TTAACTTTTG TGTGTATCGA ---------- ---------- --------TG TTTTTTCCG- ---------- ---------- ---------- ----------
S. limosum            CATATTTATG TCAGTGTTTT TTCAATTACT CTTATTGTTT TAAAAGAATT TTATTTTTTT ATATTATTCC ATAGATATTT GTTATTGGCA CTTTTGACAT
G. antarctica         TTGTTACTTC TGTGCGTATC AGTGATACTT TTGTGTTTAC CACAATCGTA TATTACCA-- ---------- ---------- ---------- ----------
D. aphelis            TTACCTTGTT GTAGCTTTTG TGCGTATAGA TGTTTTTTCC GTA------- ---------- ---------- ---------- ---------- ----------
P. peruviana          ATTGTATTAA TATCATACAC AA-------- ---------- ---------- ---------- ---------- ---------- ---------- ----------
M. secans             TTTTTATTAT TTATAA---- ---------- ---------- ---------- ---------- ---------- ---------- ---------- ----------
Quinqueloculina sp.   GTATTATTAA TTATTTATAA ---------- ---------- ---------- ---------- ---------- ---------- ---------- ----------
N. haylinosphaira     CAGAGTTTAC GTCTCCGTAT TATTCAGTG- ---------- ---------- ---------- ---------- ---------- ---------- ----------
M. fusca              ATTCTATTAC TTTAATTTCT T--------- ---------- ---------- ---------- ---------- ---------- ---------- ----------
T. alba               TCTCTGTCTT GCTTGGGTAG CAATTACGTC TACTTTTTAG CTATACGGCA GAGATGTTCC TGACTTTTTT TATAAACAAA AAAACAAGGA ATTTACAGT-
A. mexicana           GGTAGCTTTT GTGTTCATGA ATGTATTTTT CCG------- ---------- ---------- ---------- ---------- ---------- ----------
A. triangularis       TTTAAAATTG ATGCACACTT TTATGTCTAT GTTTCTATT- -AACATATTA GGATATTAAT ACTATTTATT AATAGTTTAA TTTCTAATTT TGTTATAGTT
A. rara               TTTAAAATTG ATGCACACTT TTATGTCTAT GTTTCTATTT TTACATGTTA GAATATTAAT ACTATTTATT AATAGTTTAA TTTTTAATTT TGTGATAGTT
E. scabrum            GTTTTTTTTT TTAAAATACT ATTTTGTGCG TATCGATCGA ATCCCGTA-- ---------- ---------- ---------- ---------- ----------
N. venosus            CAGGTTTCTT GTGTGTATTG ATGTTTTTTC CGT------- ---------- ---------- ---------- ---------- ---------- ----------
B. marginata          GTAGCTTTTG TGCGTATAGA TGTTTTTTCC GT-------- ---------- ---------- ---------- ---------- ---------- ----------
Trochammina sp.       CCGG--CTTA AGCTTGCCTT AAG---TTTT GTGCGTAT-- -----CGATG TTTTTTCCG- ---------- ---------- ---------- ----------
Peneroplis sp.        ACTATTA--- ---------- ---------- ---------- ---------- ---------- ---------- ---------- ---------- ----------
S. orbiculus          ---------- ---------- ---------- ---------- ---------- ---------- ---------- ---------- ---------- ----------
Allogromia sp.        TAATCTTAGT CTTTTTAGAT TTTGTATTAA AGTTAAAA-- ---------- ---------- ---------- ---------- ---------- ----------
```

```
                          1601       1611       1621       1631       1641       1651       1661       1671       1681       1691      1700
                          |          |          |          |          |          |          |          |          |          |         |
G. siphonifera Ia1        ---------- ---------- TATGC--AA- --GTGTCAAT T-----CTCA -------GTG -GGGACAGCC ATTTGA--TA ATTCTTTGGC TCGGCCTCAA
G. siphonifera Ia2        ---------- ---------- TATGC--AA- --GTGTCAAT T-----CTCA -------GTG -GGGACAGCC ATTTGA--TA ATTCTTTGGC TCGGCCTCAA
G. siphonifera IIa1       ---------- ---------- TATGC--AA- --GTGTCAAT T-----CTCA -------GTG -GGGACAGAC ATTTGA--TA ATTCTTTGTC TCGTTCTTAA
G. siphonifera IIa2       ---------- ---------- TATGC--AA- --GTGTCAAT T-----CTCA -------GTG -GGGACAGAC ATTTGA--TA ATTCTTTGTC TCGTTCTTAA
G. siphonifera IIa3       ---------- ---------- TATGC--AA- --GTGTCAAT T-----CTCA -------GTG -GGGACAGAC ATTTGA--TA ATTCTTTGTC TCGTTCTTAA
G. siphonifera IIa        ---------- ---------- TATGC--AA- --GTGTCAAT T-----CTCA -------GTG -GGGACAGAC ATTTGA--TA ATTCTTTGTC TCGTTCTTAA
G. siphonifera IIb        ---------- ---------- TATGC--AA- --GTGTCAAT T-----CTCA -------GTG -GGGACAGAC ATTTGA--TA ATTCTTTGTC TCGTTCTTAA
G. calida                 ---------- ---------- TATGC--AA- --GTGTCAAT T-----CTCA -------GTG -GGGACAGAC ATTTGA--TA ATTCTTTGTC TCGTTCTTAA
O. universa I             ---------- ---------- TATGC--AAC --TTGTCAAC T-----CTAC -------GTG -GGGATAGTC GCCTG---AA ATTCTTCGAC TCG-TTCTAA
O. universa III           ---------- --------CG TATGC--AAC --TTGTCAAT T-----CTTC -------GTG -GGGATCGTC GATTG---CA ACTATTCGAC TCG-CCACAA
G. sacculifer             ---------- ---------- TATGC---TC --CTATAAAT T-----CCTG -------GTA -GGGATAGTC TATTG---TA ACTGGTAGAC TTG---TCAA
G. ruber pink             ---------- ---------- TATTC----C --TGG-TGAC T-----CATG -------GTG -GGGACCGAC ATTTG---TA ATTGTCTGTC GCG-TGTTAA
G. ruber Ia               ---------- ---------- ---------C --TGG-CGAC T-----CATG -------GTG -GGGACCGAC GTTTG---TA ATTTTTTGTC GCG-TGTTAA
G. ruber Ib1              ---------- ---------- ---------C --TGG-TGAC T-----CATG -------GTG -GGGACCGAT GTTTG---TA ATTGTTTGTC GCG-TGTCAA
G. ruber Ib2              ---------- ---------- ---------C --TGG-TGAC T-----CATG -------GTG -GGGACCGAT GTTTG---TA ATTGTTTGTC GCG-TGTCAA
G. ruber IIa              ---------- -----GACAC ATGCAATTCC --TGGTTGAC T-----CATC -------GTG -GGGACTGAT TCTTG---TA ATTATTTTTC ACGG-TTCAA
G. conglobatus            ---------- ---------- -------TCC --TGGTTGAC T-----CATC -------GTG -GGAACTGAT TCTTG---TA ATTATTTGTC ACGG-TTCAA
G. rubescens (pink)       ---------- ---------- GGCGT-TCTT --TGGTTGAC T-----CATC -------GTG -GGGACTGAT TCTTG---TA ATTATGTTTC ACG-TGTCAA
G. bulloides Ia           ACTCGC---- ---------- CCGCC--CAT --CTTTCAAT T-----CTTG -------GTG -GGGACAGTA GGTTGT--TA ACTTTCTTAC TCGGTCCTAA
G. bulloides Ib           GCTC------ ---------- GCCTC-TCAA --CTGTCAAT T-----CTTG -------GTG -GGGACAGTC AGTTGT--TA ACTTTCTGAC TCG-TCTGAA
G. bulloides IIa          GCCCT----- ---------- TTGTG--CTT --TTTTCAAA T-----CTTA -------GTG -GGGACAGAC ATCTGT--TA ACTTTTTGTC TCGGTCCCAA
G. bulloides IIb          GTCCT----- ---------- TTGTG--CTT --TTTTCAAT T-----CTTA -------GTG -GGGACAGAC ATCTGT--TA ACTTTTTGTC TCGGTCCCAA
G. bulloides IIc          GTCCT----- ---------- TTGTG--CTT --TTTTCAAA T-----CTTA -------GTG -GGGACAGAC ATCTGT--TA ACTTTTTGTC TCGGTCCCAA
G. bulloides IId          GTCCT----- ---------- TTGTG--CTT --TTTTCAAA T-----CTTA -------GTG -GGGACAGAC ATCTGT--TA ACTTTTTGTC TCGGTCCCAA
G. bulloides IIe          GTCTCT---- ---------- TTGTG--CTT --TTTTCAAA T-----CTTA -------GTG -GGGACAGAC AATTGT--TA ACTTTTTGTC TCGGTCCTAA
T. quinqueloba Ia         ---------- ---------- CACTA--GTA CGCTTCTAAT T-----CACA -------GTG -GGGACAGTC GTTTG---TA ATTCTGAGAC TCGGT-TCAA
T. quinqueloba Ib         ---------- ---------- CACTA--GTA CGCTTCTAAT T-----CACA -------GTG -GGGACAGTC GTTTG---TA ATTCTGAGAC TCGGT-TCAA
T. quinqueloba IIa        ---------- ---------- CGCTA--GTA --TTGTTAAT T-----CACA -------GTG -GGGACAGTC GTTTG---TA ATTCTGAGAC TCGGT-TCAA
T. quinqueloba IIb        ---------- ---------- CTCTA--GTA --TTGTTAAT T-----CACA -------GTG -GGGACAGTC GTTTG---TA ATTCTGAGAC TCGGT-TCAA
T. quinqueloba IIc        ---------- ---------- CTCTA--GTA --TTGTTAAT T-----CACA -------GTG -GGGACAGTC GTTTG---TA ATTCTGAGAC TCGGT-TCAA
T. quinqueloba IId        ---------- ---------- CTCTA--GTA --TTGTTAAT T-----CACA -------GTG -GGGACAGTC GTTTG---TA ATTCTGAGAC TCGGT-TCAA
G. falconensis            ---------- ---------- TCGTC-GCAA --CTCTCAAT T-----CACA -------GTG -GGGACAGTC GTTTGT--CA ACTTTTCGTC TCGGTCTCAA
H. pelagica               ---------- ---------- TCTGC---AA --TTGTCAAT T-----CACA -------GTG -GGGATAGTC GATTGG--TA GCAATTCGAC TCGGTCCTAA
G. menardii               ---------- ---------- CATGT-ACCA --TTGTTAAC T-----CCGG -------GTA -GGGACAGAC CTTTG---AA ACTCTCGGTC TCGGTCTCAA
G. ungulata               ---------- ---------- CATGT-ACCA --TTGTTAAA T-----CCGG -------GTA -GGGACAGAC CTTTG---AA ATTCTCGGTC TCGGTCTTAA
G. hirsuta                ---------- ---------- TATGT-GCAA --TTGTTAAT T-----CGTG -------GTG -GGGACAGAC CATTGT--TA ACTATTGGTC TCGGTCTTAA
G. scitula                ---------- ---------- TATGT-GCGA --TTGTCAAT T-----CGTG -------GTG -GGGACAGAC CATTGT--TA ACTGTTGGTC TCGGTCTTAA
G. truncatulinoides       ---------- ---------- -TGGC-GCAC --TTACTTAT T-----CACG -------GTG -GGGACAGAC CATTGT--TA ACTGTTGGTC TCGGTCTCAA
N. pachyderma I           ---------- ---------- TACGT-GCAA --TTGTCAAT T-----CATG -------GTG -GGGACAGAC CATTGT--TA ACTGTTGGTC TCGGTCTTAA
N. pachyderma II          ---------- ---------- TGCGT-GCAA --TTGTCAAT T-----CATG -------GTG -GGGACAGAC CATTGT--TA ACTGTTGGTC TCGGTCTTAA
N. pachyderma III         ---------- ---------- TGCGT-GCAA --TTGTCAAT T-----CATG -------GTG -GGGACAGAC CATTGT--TA ACTGTTGGTC TCGGTCTTAA
N. pachyderma IV          ---------- ---------- TGCGT-GCAA --TTGTCAAT T-----CATG -------GTG -GGGACAGAC CATTGT--TA ACTGTTGGTC TCGGTCTTAA
N. pachyderma V           ---------- ---------- TGCGT-GCAA --TTGTCAAT T-----CATG -------GTG -GGGACAGAC CATTGT--TA ACTGTTGGTC TCGGTCTTAA
N. pachyderma VI          ---------- ---------- TGCGT-GCAA --TTGTCAAT T-----CATG -------GTG -GGGACAGAC CATTGT--TA ACTGTTGGTC TCGGTCTTAA
N. pachyderma VII         ---------- ---------- TGCGT-GCAA --TTGTCAAT T-----CATG -------GTG -GGGACAGAC CATTGT--TA ACTGTTGGTC TCGGTCTTAA
N. dutertrei_C            ---------- ---------- TATGT-GCGA --TTGTCAAT T-----CATG -------GTG -GGGACAGAC CATTGT--TA ATTGTTGGTC TCGGTCTTAA
N. dutertrei_Ib           ---------- ---------- TATGT-GCGA --TTGTCAAT T-----CATG -------GTG -GGGACAGAC CATTGT--TA ATTGTTGGTC TCGGTCTTAA
P. obliquiloculata_BR     ---------- ---------- TATGT-GCGA --TTGTCAAT T-----CATG -------GTG -GGGACAGAC CATTGT--TA ATTGTTGGTC TCGGTCTTAA
P. obliquiloculata_AS     ---------- ---------- TATGT-GCGA --TTGTCAAT T-----CATG -------GTG -GGGACAGAC CATTGT--TA ATTGTTGGTC TCGGTCTTAA
G. inflata                ---------- ---------- TATGT-GCGA --TTGTCAAT T-----CATG -------GT- -GGGACAGAC CATTGT--TA ATTGTTGGTC TCGGTCTTAA
G. crassaformis           ---------- ---------- TATGT-GCGA --TTGTCAAT T-----CATG -------GTG -GGGACAGAC CATTGT--TA ATTGTTGGTC TCGGTCTTAA
N. incompta I             ---------- ---------- TATGT-GCCC --CTGTCAAT T-----CGTG -------GTG -GGGACAGAC CATTGC--TA ATTGTTGGTC TCGCTTATAA
N. incompta II            ---------- ---------- TATGT-GCCC --CTGTCAAT T-----CGTG -------GTG -GGGACAGAC CATTGC--TA ATTGTTGGTC TCGCTTATAA
G. glutinata Ia1          ---------- ---------- CATGT-GCAA --TTGTCAAT T-----CATG -------GTG -GGGACAGAC CATTGT--TA ATTGTTGGTC TCGGTCTTAA
G. glutinata Ia2          ---------- ---------- TAAGT-GCAA --TTGTCAAT T-----CATG -------GTG -GGGACAGAC CATTGT--TA ATTGTTGGTC TCGGTCTTAA
G. glutinata Ia3          ---------- ---------- TAAGT-GCAA --TTGTCAAT T-----CATG -------GTG -GGGACAGAC CATTGT--TA ATTGTTGGTC TCGGTCTTAA
C. nitida                 ---------- ---------- TATGT-GCAA --TTGTCAAT T-----CATG -------GTG -GGGACAGAC CATTGT--TA ATTGTTGGTC TCGGTCTTAA
G. uvula                  ---------- --------CT TATGT-GCAA --TTGTCAAT T-----CATG -------GTG -GGGACAGAC CATTGT--TA ATTGTTGGTC TCGGTCTTAA
B. variabilis             ---------- ---------- CATGT-GCAA --TTGTCAAT T-----CATG -------GTG -GGGACAGAC CATTGTTTCA ATTGTTGGTC TCGGTCTTAA
S. globigerus             ---------- ---------- CATGT-GCAA --TTGTCAAT T-----CATG -------GTG -GGGACAGAC CATTGTTTCA ATTGTTGGTC TCGGTCTCAA
B. alata                  ---------- ---------- CATGT-GCAA --TTGTCAAT T-----CATG -------GTG -GGGACAGAC CATTGTTTCA ATTGTTGGTC TCGGTCTTAA
G. vivans                 ---------- ---------- --TGT-GCAA --TTGTCAAT T-----CATG -------GTG -GGGACAGAC CATTGT--TA ATTGTTGGTC TCGGTCTTAA
C. porrectus              ---------- ---------- -ATGT-GCAA --TTGTCAAT T-----CATG -------GTG -GGGACAGAC CATTGT--TA ATTGTTGGTC TCGGTCTTAA
C. ovoidea                GT-------- ---------- -ATGT-GCAA --TTGTCAAT T-----CATG -------GTG -GGGACAGAC CATTGT--TA ATTGTTGGTC TCGGTCTTAA
G. opercularis            ---------- ---------- TCGTG-CAA- --TTGTCAAT T-----CATG -------GTG -GGGACAGAC CATTGT--TA ATTGTTGGTC TCGGTCTTAA
E. aculeatum              ---------- ---------- TACGC-GTAA --ATATTAAT T-----CATG -------GTG -GGGATAGTC CATTGT--TA ATTGTTGGTC TCGGTCTAAA
E. vitrea                 ---------- ---------- TATGT-GCGA --TTGTCAAT T-----CATG -------GTG -GGGACAGAC CATTGT--TA ATTGTTGGTC TCGGTCTTAA
H. germanica              ---------- ---------- TATGT-GCAA --TTGTCAAT T-----CATG -------GTG -GGGACAGAC CATTGT--TA ATTGTTGGTC TCGGTCTTAA
P. mediterranensis        ---------- ---------- -ATGT-GCAA --TTGTCAAT T-----CATG -------GTG -GGGACAGAC CATTGT--TA ATTGTTGGTC TCGGTCTTAA
S. fusiformis             ---------- ---------- TATGT-GCAA --TTGTCAAT T-----CATG -------GTG -GGGACAGAC CATTGT--TA ATTGTTGGTC TCGGTCTTAA
V. fragilis               ---------- ---------- TATGT-GCAA --TTGTCAAT T-----CATG -------GTG -GGGACAGAC CATTGT--TA ATTGTTGGTC TCGGTCTTAA
A. pseudocassis           ---------- ---------- TGCGT-GCAA --TTGTTAAT T-----CATG -------GTG -GGGACAGAC CATTGT--TA ATTGTTGGTC TCGGTCTCAA
Spiroplectammina sp.      ---------- ---------- TATGT-GCAA --TTGTCAAT T-----CATG -------GTG -GGGACAGAC CATTGT--TA ATTGTTGGTC TCGGTCTTAA
Textularia sp.            ---------- ---------- CATGT-GCAA --TTGTCAAT T-----CATG -------GTG -GGGACAGAC CATTGT--TA ATTGTTGGTC TCGGTCTTAA
S. limosum                GTGCTCCA-- ---------- TTTTT-TTA- --ATGACAAT T-----CGTG -------GTG -GGGACAGAC CATTGA--TA ATTGTTGGTC TCGGTCTCAA
G. antarctica             ---------- ---------- TATGT-GCAA --TTGTCAAT T-----CATG -------GTG -GGGACAGAC CATTGT--TA ATTGTTGGTC TCGGTCTTAA
D. aphelis                ---------- ---------- --TGT-GCGA --TTGTCAAT T-----CATG -------GTG -GGGACAGAC CATTGT--TA ATTGTTGGTC TCGGTCTTAA
P. peruviana              ---------- --------TA TGTTG-TAT- --TAATTAAT T-----TAAG -------GTG -GGGATAGTG TATTGT--TA ATTATTACAC TTGGCCTTAA
M. secans                 ---------- ---------- CTGTA-GTAT --TAATTAAT T-----TAAG -------GTG -GGGATAGTG AATTGT--TA ATTATTTCAC TTGGCCTTAA
Quinqueloculina sp.       ---------- ---------- CTGTA-GTAT --TAATTAAT T-----TAAG -------GTG -GGGATAGTG AATTGT--TA ATTATTTCAC TTGGCCTTAA
N. haylinosphaira         ---------- ---------- GCATG-TGCT --CCATTAAT T-----CGTG -------GTG -GGGACAGAC CATTGT--TA ATTGTTGGTC TCGGTCTCAA
M. fusca                  ---------- ---------- TGTGT-TATT ---AATTAAT T----CCAAG -------GTG -GGGACAGAC CATTGT--TA ATTGTTGGTC TCGTTTTTAA
T. alba                   ---------- ---------- GCGTG-CTTT --TTGTCAAT T-----CATG -------GTG -GGGACAGAC CATTGT--TA ATTATTGGTC TCGGTCTTAA
A. mexicana               ---------- ---------- TATGT-GCAA --ATGTCAAT T-----CATT -------GTG -GGGACAGAC CATTGT--TA ATTGTTGGTC TCGGTCTTAA
A. triangularis           ACTGACATGT GCTCTCATGT TTTAT-TAAA --TGTTCAAT T-----CGTG -------GTG -GGGACAGAC CATTGT--TA ATTGTTGGTC TCGGTCTCAA
A. rara                   ACTGACATGT GCT---CTCA TATTT-TAAA --TGTTCAAT T-----CGTG -------GTG -GGGACAGAC CATTGT--TA ATTGTTGGTC TCGGTCTCAA
E. scabrum                ---------- ---------- TGTGC-AAT- --TTGTCAAT T-----CATG -------GTG -GGGATAGAC CATTGT--TA ATTGTTGGTC TCGGTCTTAA
N. venosus                ---------- ---------- ATGTG-CGA- --TTGTCAAT T-----CATG -------GTG -GGGACAGAC CATTGT--TA ATTGTTGGTC TCGGTCTTAA
B. marginata              ---------- ---------- ATGTG-CGA- --TTGTCAAT T-----CATG -------GTG -GGGACAGAC CATTGT--TA ATTGTTGGTC TCGGTCTTAA
Trochammina sp.           ---------- ---------- TATGT-GCAA --TTGTCAAT T-----CATG -------GTG -GGGACAGAC CATTGT--TA AGTGTTGGTC TCGGTCTTAA
Peneroplis sp.            ---------- ---------C AAATA-CCAT --TAATTAAT T-----TAAG -------GTG -GGGATAGTG TATTGT--TA ATTATTACAC TTGGCCTTAA
S. orbiculus              ---------- ---------- TATAA-ATA- --TAATTAAT T-----TAAG -------GTG -GGGATAGTG TATTGT--TA ATTATTACAC TTGGCCTTAA
Allogromia sp.            ---------- ---------- TATGT-GCT- --CCTTTATT T-----CATG -------GTG -GGGACTGAC CATTGT--TA ATTGTTGGTC ACG-TCTCAA
```

```
                           1701      1711      1721      1731      1741      1751      1761      1771      1781      1791 1800
                           |         |         |         |         |         |         |         |         |         |    |
G. siphonifera Ia1         CTAGGAATGC CTTGTACGGG TCTT-GGTTC AAC-AGACCA CCCGGAATAC GTCCCTGCCC TTTGTACACA CCGCCCGTCG CTCT-TACCG A-TG--AATT
G. siphonifera Ia2         CTAGGAATGC CTTGTACGGG TCTT-GGTTC AAC-AGACCA CCCGGAATAC GTCCCTGCCC TTTGTACACA CCGCCCGTCG CTCT-TACCG A-TG--AATT
G. siphonifera IIa1        CTAGGAATGC CTTGTACGGG TCTT-GGTTC AAC-AGACCA CCCGGAATAC GTCCCTGCCC TTTGTACACA CCGCCCGTCG CTCT-TACCG A-TG--AATT
G. siphonifera IIa2        CTAGGAATGC CTTGTACGGG TCTT-GGTTC AAC-AGACCA CCCGGAATAC GTCCCTGCCC TTTGTACACA CCGCCCGTCG CTCT-TACCG A-TG--AATT
G. siphonifera IIa3        CTAGGAATGC CTTGTACGGG TCTT-GGTTC AAC-AGACCA CCCGGAATAC GTCCCTGCCC TTTGTACACA CCGCCCGTCG CTCT-TACCG A-TG--AATT
G. siphonifera IIa         CTAGGAATGC CTTGTACGGG TCTT-GGTTC AAC-AGACCA CCCGGAATAC GTCCCTGCCC TTTGTACACA CCGCCCGTCG CTCT-TACCG A-TG--AATT
G. siphonifera IIb         CTAGGAATGC CTTGTACGGG TCTT-GGTTC AAC-AGACCA CCCGGAATAC GTCCCTGCCC TTTGTACACA CCGCCCGTCG CTCT-TACCG A-TG--ACTT
G. calida                  CTAGGAATGC CTTGTACGGG TCTT-GGTTC AAC-AGACCA CCCGGAATAC GTCCCTGCCC TTTGTACACA CCGCCCGTCG CTCT-TACCG A-TG--AATT
O. universa I              CTAGGAATGC CTTGTACGGG CCTT-GGTTC ATT-ATGCCG CCCGGAATAC GTCCCTGCCC TTTGTACACA CCGCCCGTCG CTCT-TACCG ATTG--AACT
O. universa III            CTAGGAATGC CTCGTACGGG TCTC-GGTTC ACC-ATACCG CCCGGAATAC GTCCCTGCCC TTTGTACACA CCGCCCGTCG CTCT-TACCG ATTG--AACT
G. sacculifer              CTAGGAATGC CTTGTACGGG TG--GGGTAG CAC-ATTCCA CCCGGGATAC GTCCCTGCCT TTTGTACACA CCGCCCGTCG CTCT-TACCA ATTG--AACA
G. ruber pink              CCGGGAATGC CTTGTACTGT CG----ATTC ACT-AAGTTA TGGGGAATAC GTCCCTGCCC TTTGTACACA CCGCCCGTCG CTCT-TACCG A-TG--GCTT
G. ruber Ia                CTAGGAATGC CTTGTACTGT CG----ATTC ACT-AAGTTA CTGGGAATAC GTCCCTGCCC TTTGTACACA CCGCCCGTCG CTCT-TACCG A-TG--GCTG
G. ruber Ib1               CTAGGAATGC CTTGTACTGT CG----ATTC ACT-AAGTTA TTGGGAATAC GTCCCTGCCC TTTGTACACA CCGCCCGTCG CTCT-TACCG A-TG--GCTT
G. ruber Ib2               CTAGGAATGC CTTGTACTGT CG----ATTC ACT-AAGTTA TTGGGAATAC GTCCCTGCCC TTTGTACACA CCGCCCGTCG CTCT-TACCG A-TG--GCTT
G. ruber IIa               CCAGGAATGC CTTGTACCGG CG----GCTC ATT-AAACCG CTGGGAATAC GTCCCTGCCC TTTGTACACA CCGCCCGTCG CTCT-TACCG A-TG--ACTT
G. conglobatus             CCAGGAATGC CTTGTACTGG CG----GCTC ATT-AAACCG CTCGGTATAC GTCCCTGCCC TTTGTACACA CCGCCCGTCG CTCT-TACCG A-TG--ACTT
G. rubescens (pink)        CCGGGAATGC CTTGTACAGG TTCCCGGTTC AAC-AGACCA CCTGGAATAC GTCCCTGCCC TTTGTACACA CCGCCCGTCG CTCT-CACCA A-TG--AGCT
G. bulloides Ia            CCAGGAATGC CTCGTACAGG TT---GGTTC ACC-ATACCA CCTGGAATTA GTCCCTGCCC TTTGTACACA CCGCCCGTCG CTTT-TACCA A-TG--AACT
G. bulloides Ib            CCAGGAATGC CTCGTACAGG TT---GGTTC ACC-ATACCA CCTGGAAT-A GTCCCTGCCC TTTGTACACA CCGCCCGTCG CTTT-TACCA A-TG--AACT
G. bulloides IIa           CCAGGAATGC CTCGTACAGG TT---GGTTC ACC-ATACCA CCTGGAATTA GTCCCTGCCC TTTGTACACA CCGCCCGTCG CTTT-TACCA A-TG--AACT
G. bulloides IIb           CCAGGAATGC CTCGTACAGG TT---GGTTC ACC-ATACCA CCTGGAATTA GTCCCTGCCC TTTGTACACA CCGCCCGTCG CTTT-TACCA A-TG--AACT
G. bulloides IIc           CCAGGAATGC CTCGTACAGG TT---GGTTC ACC-ATACCA CCTGGAATTA GTCCCTGCCC TTTGTACACA CCGCCCGTCG CTTT-TACCA A-TG--AACT
G. bulloides IId           CCAGGAATGC CTCGTACAGG TT---GGTTC ACC-ATACCA CCTGGAATTA GTCCCTGCCC TTTGTACACA CCGCCCGTCG CTTT-TACCA A-TG--AACT
G. bulloides IIe           CCAGGAATGC CTCGTACAGG TT---GGTTC ACC-ACACCA CCTGGAATTA GTCCCTGCCC TTTGTACACA CCGCCCGTCG CTTT-TACCA A-TG--AACT
T. quinqueloba Ia          CTAGGAATGC CTAGTATTGA TG----GTTC ACT-AAACTT TCTGGAATAA GTCCCTGCCC TTTGTACACA CCGCCCGTCG CTTT-TACCA A-TG--GCCC
T. quinqueloba Ib          CTAGGAATGC CTAGTATTGA TG----GTTC ACT-AAACTT TCTGGAATAA GTCCCTGCCC TTTGTACACA CCGCCCGTCG CTTT-TACCA A-TG--GCCC
T. quinqueloba IIa         CCAGGAATGC CTCGTATTTG TG----GTTC ATT-ATACTC CGTGGAATAA GTCCCTGTCC TTTGTACACA CCGCCCGTCG CTTT-TACCA A-TG--GCCC
T. quinqueloba IIb         CCAGGAATGC CTCGTATTTG TG----GTTC ATT-AAACTC CGTGGAATAA GTCCCTGTCC TTTGTACACA CCGCCCGTCG CTTT-TACCA A-TG--GCCC
T. quinqueloba IIc         CCAGGAATGC CTCGTATTTG TG----GTTC ATT-AAACTC CGTGGAATAA GTCCCTGTCC TTTGTACACA CCGCCCGTCG CTTT-TACCA A-TG--GCCC
T. quinqueloba IId         CCAGGAATGC CTCGTATTTG TG----GTTC ATT-AAACTC CGTGGAATAA GTCCCTGTCC TTTGTACACA CCGCCCGTCG CTTT-TACCA A-TG--GCCC
G. falconensis             CTAGGAATGC CTCGTACGAG TT---GGTTC ATC-AGACCA CTCGGAATAC GTCCCTGCCC TTTGTACACA CCGCCCGTCG CTTT-TACCG A-TG--AACT
H. pelagica                CTAGGAATGC CTCGTACGGG TG----AATC ATT-ATTTCA CCCGGAATAC GTCCCTGCCC TTTGTACACA CCGCCCGTCG CTCT-TACCG A-TG--AATT
G. menardii                CCAGGAATGC CTCGTACGGA TTC--GGTTA AAC-ATACCA TCCGGAATAT GTCCCTGCCC TTTGTACACA CCGCCCGTCG CTCT-TACCG A-TT--GTCT
G. ungulata                CCAGGAATGC CTCGTACGAG T-T--GGTTA AAC-ATACCA CTCGGAATAT GTCCCTGCCC TTTGTACACA CCGCCCGTCG CTCT-TACCG A-TT--GTCT
G. hirsuta                 CTAGGAATGC CTCGTACGGG TCT-TGGTTC AAC-AAGCCA CCCGGAATAC GTCCCTGCCC TTTGTACACA CCGCCCGTCG CTCT-TACCG A-TG--AACT
G. scitula                 CTAGGAATGC CTCGTACGGG TCT-TGGTTC AAC-AAACCA CCCGGAATAC GTCCCTGCCC TTTGTACACA CCGCCCGTCG CTCT-TACCG A-TG--GACT
G. truncatulinoides        CTAGGAATGC CTCGTACGGG TT---GGTTC ACC-AAACCA CCCGGAATAT GTCCCTGCCC TTTGTACACA CCGCCCGTCG CTCT-TATCG A-TG--AACT
N. pachyderma I            CTAGGAATGC CTTGTACGGG TCTTTGGTTC AAC-AAACCA CCCGGAATAC GTCCCTGCCC TTTGTACACA CCGCCCGTCG CTCT-TACCG A-TG--GACT
N. pachyderma II           CTAGGAATGC CTTGTACGGG TCTTTGGTTC AAC-AAACCA CCCGGAATAC GTCCCTGCCC TTTGTACACA CCGCCCGTCG CTCT-TACCG A-TG--GACT
N. pachyderma III          CTAGGAATGC CTTGTACGGG TCTTTGGTTC AAC-AAACCA CCCGGAATAC GTCCCTGCCC TTTGTACACA CCGCCCGTCG CTCT-TACCG A-TG--GACT
N. pachyderma IV           CTAGGAATGC CTTGTACGGG TCTTTGGTTC AAC-AAACCA CCCGGAATAC GTCCCTGCCC TTTGTACACA CCGCCCGTCG CTCT-TACCG A-TG--GACT
N. pachyderma V            CTAGGAATGC CTTGTACGGG TCTTTGGTTC AAC-AAACCA CCCGGAATAC GTCCCTGCCC TTTGTACACA CCGCCCGTCG CTCT-TACCG A-TG--GACT
N. pachyderma VI           CTAGGAATGC CTTGTACGGG TCTTTGGTTC AAC-AAACCA CCCGGAATAC GTCCCTGCCC TTTGTACACA CCGCCCGTCG CTCT-TACCG A-TG--GACT
N. pachyderma VII          CTAGGAATGC CTTGTACGGG TCTTTGGTTC AAC-AAACCA CCCGGAATAC GTCCCTGCCC TTTGTACACA CCGCCCGTCG CTCT-TACCG A-TG--GACT
N. dutertrei_C             CTAGGAATGC CTTGTACGGG TCTTTGGTTC AAC-AAACCA CCCGGAATAC GTCCCTGCCC TTTGTACACA CCGCCCGTCG CTCT-TACCG A-TG--AACT
N. dutertrei_Ib            CTAGGAATGC CTTGTACGGG TCTTTGGTTC AAC-AAACCA CCCGGAATAC GTCCCTGCCC TTTGTACACA CCGCCCGTCG CTCT-TACCG A-TG--AACT
P. obliquiloculata_BR      CTAGGAATGC CTTGTACGGG TCTTTGGTTC AAC-AAACCA CCCGGAATAC GTCCCTGCCC TTTGTACACA CCGCCCGTCG CTCT-TACCG A-TG--AACT
P. obliquiloculata_AS      CTAGGAATGC CTTGTACGGG TCTTTGGTTC AAC-AAACyA CCCGGAATAC GTCCCTGCCC TTTGTACACA CCGCCCGTCG CTCT-TACCG A-TG--AACT
G. inflata                 CTAGGAATGC CTTGTACGGG TCTTTGGTTC AAC-AAACCA CCCGGAATAC GTCCCTGCCC TTTGTACACA CCGCCCGTCG CTCT-TACCG A-TG--GACT
G. crassaformis            CTAGGAATGC CTTGTACGGG TCTTTGGTTC AAC-AAACCA TCCGGAATAC GTCCCTGCCC TTTGTACACA CCGCCCGTCG CTCT-TACCG A-TG--GACT
N. incompta I              CTAGGAATGC CTTGTACGGG TCTTTGGTTC AAC-AAACCA CCCGGAATAC GTCCCTGCCC TTTGTACACA CCGCCCGTCG CTCT-TACCG A-TG--AACT
N. incompta II             CTAGGAATGC CTTGTACCGG TCTTTGGTTC ATC-AAACCA CCGGGAATAC GTCCCTGCCC TTTGTACACA CCGCCCGTCG CTCT-TACCG A-TG--AACT
G. glutinata Ia1           CTAGGAATGC CTTGTACGGG TCTTTGGTTC AAC-AAACCA CCCGGAATAC GTCCCTGCCC TTTGTACACA CCGCCCGTCG CTCT-TACCG A-TG--GACT
G. glutinata Ia2           CTAGGAATGC CTTGTACGGG TCTTTGGTTC AAC-AAACCA CCCGGAATAC GTCCCTGCCC TTTGTACACA CCGCCCGTCG CTCT-TACCG A-TG--GACT
G. glutinata Ia3           CTAGGAATGC CTTGTACGGG TCTTTGGTTC AAC-AAACCA CCCGGAATAC GTCCCTGCCC TTTGTACACA CCGCCCGTCG CTCT-TACCG A-TG--GACT
C. nitida                  CTAGGAATGC CTTGTACGGG TCTTTGGTTC AAC-AAACCA CCCGGAATAC GTCCCTGCCC TTTGTACACA CCGCCCGTCG CTCT-TACCG A-TG--GACT
G. uvula                   CTAGGAATGC CTTGTATGAG TCTTTGGTTC AAC-AAACCA CTCAGAATAT GTCCCTGCCC TTTGTACACA CCGCCCGTCG CTCT-TACCG A-TG--GACT
B. variabilis              CTAGGAATGC CTTGTACGGG TCTTTGGTTC AAC-AAACCA CCCGGAATAC GTCCCTGCCC TTTGTACACA CCGCCCGTCG CTCT-TACCG A-TG--GACT
S. globigerus              CTAGGAATGC CTTGTACGGG TCTTTGGTTC AAC-AAACCA CCCGGAATAC GTCCCTGCCC TTTGTACACA CCGCCCGTCG CTCT-TACCG A-TG--GACT
B. alata                   CTAGGAATGC CTTGTACGGG TCTTTGGTTC AAC-AAACCA CCCGGAATAC GTCCCTGCCC TTTGTACACA CCGCCCGTCG CTCT-TACCG A-TG--GACT
G. vivans                  CTAGGAATGC CTTGTACGGG TCTTTGGTTC AAC-AAACCA CCCGGAATAC GTCCCTGCCC TTTGTACACA CCGCCCGTCG CTCT-TACCG A-TG--GACT
C. porrectus               CTAGGAATGC CTTGTACGGA TCTTTGGTTC AAC-AAACCA TCCGGAATAC GTCCCTGCCC TTTGTACACA CCGCCCGTCG CTCT-TACCG A-TG--GACT
C. ovoidea                 CTAGGAATGC CTTGTACGGG TCTTTGGTTC AAC-AAACCA CCCGGAATAC GTCCCTGCCC TTTGTACACA CCGCCCGTCG CTCT-TACCG A-TG--GACT
G. opercularis             CTAGGAATGC CTTGTACGGG TCTTTGGTTC AAC--AACCA CCCGGAATAC GTCCCTGCCC TTTGTACACA CCGCCCGTCG CTCT-TACCG A-TG--GATT
E. aculeatum               CTAGGAATGC CTTGTACGGG TTATTGGTTC AAC-AAACCA CCCGGAATAC GTCCCTGCCC TTTGTACACA CCGCCCGTCG CTCT-TACCG A-TG--AACT
E. vitrea                  CTAGGAATGC CTTGTACGGG TCTTTGGTTC AAC-AAACCA CCCGGAATAC GTCCCTGCCC TTTGTACACA CCGCCCGTCG CTCT-TACCG A-TG--GACT
H. germanica               CTAGGAATGC CTTGTACGGG TCTTTGGTTC AAC-AAACCA CCCGGAATAC GTCCCTGCCC TTTGTACACA CCGCCCGTCG CTCT-TACCG A-TG--GACT
P. mediterranensis         CTAGGAATGC CTTGTACGGG TCTTTGGTTC AAC-AGACCA CCCGGAATAC GTCCCTGCCC TTTGTACACA CCGCCCGTCG CTCT-TACCG A-TG--GACT
S. fusiformis              CTAGGAATGC CTTGTACGGG TCTTTGGTTC AAC-AAACCA CCCGGAATAC GTCCCTGCCC TTTGTACACA CCGCCCGTCG CTCT-TACCG A-TG--GACT
V. fragilis                CTAGGAATGC CTTGTACGGG TCTTTGGTTC AAC-AAACCA CCCGGAATAC GTCCCTGCCC TTTGTACACA CCGCCCGTCG CTCT-TACCG A-TG--GACT
A. pseudocassis            CTAGGAATGC CTTGTACGGG TCTTTGGTTC AAC-AGACCA CCCGGAATAC GTCCCTGCCC TTTGTACACA CCGCCCGTCG CTCT-TACCG A-TG--GACT
Spiroplectammina sp.       CTAGGAATGC CTTGTACGGG TCTT-GGTTC AAC-AAACCA CCCGGAATAC GTCCCTGCCC TTTGTACACA CCGCCCGTCG CTCT-TACCG A-TG--GACT
Textularia sp.             CTAGGAATGC CTTGTACGGG TCTTTGGTTC AAC-AAACCA CCCGGAATAC GTCCCTGCCC TTTGTACACA CCGCCCGTCG CTCT-TACCG A-TG--GACT
S. limosum                 CTAGGAATGC CTTGTACTGG TCTTTGGTTC AAC-AAACCA CCAGGAATAT GTCCCTGCCC TTTGTACACA CCGCCCGTCG CTCT-TACCG A-TG--GGCT
G. antarctica              CTAGGAATGC CTTGTACGGA TCTTTGGTTC AAC-AAACCA TCCGGAATAC GTCCCTGCCC TTTGTACACA CCGCCCGTCG CTCT-TACCG A-TG--GACT
D. aphelis                 CTAGGAATGC CTTGTACGGG TCTTTGGTTC AAC-AAACCA CCCGGAATAC GTCCCTGCCC TTTGTACACA CCGCCCGTCG CTCT-TACCG A-TG--GACT
P. peruviana               CTAGGAATGC CTTGTACTGT TCCTTGGTTC ATC-ATACCA ACAGGAATAC GTCCCTGCCC TTTGTACACA CCGCCCGTCG CTCT-TACCG A-TG--AATT
M. secans                  CTAGGAATGC CTTGTACTGT TCCTTGGTTC AAC-ATACCA ACAGGAATAC GTCCCTGCCC TTTGTACACA CCGCCCGTCG CTCT-TACCG A-TG--AATT
Quinqueloculina sp.        CTAGGAATGC CTTGTACTGT TCCTTGGTTC AAC-ATACCA ACAGGAATAC GTCCCTGCCC TTTGTACACA CCGCCCGTCG CTCT-TACCG A-TG--AATT
N. haylinosphaira          CTAGGAATGC CTTGTACGGG TCTTTGGTTC AAC-AGACCA CCCGGAATAT GTCCCTGCCC TTTGTACACA CCGCCCGTCG CTCT-TACCG A-TG--GACT
M. fusca                   CTAGGAATGC CTTGTACTGG TCTTTGGTTC ATC-AGACCA CCAGGAATAC GTCCCTGCCC TTTGTACACA CCGCCCGTCG CTCT-TACCG A-TG--GACT
T. alba                    CTAGGAATGC CTTGTACGGG TCTTTGGTTC AAC-AAACCA CCCGGAATAC GTCCCTGCCC TTTGTACACA CCGCCCGTCG CTCT-TACCG A-TG--GACT
A. mexicana                CTAGGAATGC CTTGTACGGG TCTTTGGTTC AAC-AAACCA CCCGGAATAC GTCCCTGCCC TTTGTACACA CCGCCCGTCG CTCT-TACCG A-TG--GACT
A. triangularis            CTAGGAATGC CTTGTACGGG TCTTTGGTTC AAC-AAACCA CCCGGAATAT GTCCCTGCCC TTTGTACACA CCGCCCGTCG CTCT-TACCG A-TG--GACT
A. rara                    CTAGGAATGC CTTGTACGGG TCTTTGGTTC AAC-AAACCA CCCGGAATAT GTCCCTGCCC TTTGTACACA CCGCCCGTCG CTCT-TACCG A-TG--GACT
E. scabrum                 CTAGGAATGC CTTGTACGGG TCTTTGGTTC AAC-AAACCA CCCGGAATAC GTCCCTGCCC TTTGTACACA CCGCCCGTCG CTCT-TACCG A-TG--GACT
N. venosus                 CTAGGAATGC CTTGTACGGG TCTTTGGTTC AAC-AAACCA CCCGGAATAC GTCCCTGCCC TTTGTACACA CCGCCCGTCG CTCT-TACCG A-TG--GACT
B. marginata               CTAGGAATGC CTTGTACGGG TCTTTGGTTC AAC-AAACCA CCCGGAATAC GTCCCTGCCC TTTGTACACA CCGCCCGTCG CTCT-TACCG A-TG--GACT
Trochammina sp.            CTAGGAATGC CTTGTACGGG TCTTTGGTTC AAC-AAACCA CCCGGAATAC GTCCCTGCCC TTTGTACACA CCGCCCGTCG CTCT-TACCG A-TG--GACT
Peneroplis sp.             CTAGGAATGC CTTGTACTCT TCTTTGGTTT AAC-ATACCA AGAGGAATAC GTCCCTGCCC TTTGTACACA CCGCCCGTCG CTCT-TACCG A-TG--AATT
S. orbiculus               CTAGGAATGC CTTGTACTCT TCTTTGGTTT AAC-ATACCA AGAGGAATAC GTCCCTGCCC TTTGTACACA CCGCCCGTCG CTCT-TACCG A-TG--AATT
Allogromia sp.             CTAGGAATGC CTTGTACTGG TCT-TGGTTC AAC-AAACCA CCAGGAATAT GTCCCTGCCC TTTGTACACA CCGCCCGTCG CTCT-TACCG A-TG--GACT
```

```
                         1801       1811       1821       1831       1841       1851       1861       1871       1881       1891      1900
                         |          |          |          |          |          |          |          |          |          |         |
G. siphonifera Ia1       GTA-CTG-TG AG-----TTT GCAGGACCGA -----ACCCA ---------- ---------- ---------- ---------- ---------- ----------
G. siphonifera Ia2       GTA-CTG-TG AG-----TTT GCAGGACCGA -----ACCCA ---------- ---------- ---------- ---------- ---------- ----------
G. siphonifera IIa1      TCC-CTG-TG AG-----TTT GAAGGACTGA -----TGGTT GNAAAATC-- ---------- ---------- ---------- ---------- ----------
G. siphonifera IIa2      TCC-CTG-TG AG-----TTT GAAGGACTGA -----TGGTT GCAAAATC-- ---------- ---------- ---------- ---------- ----------
G. siphonifera IIa3      TCC-CTG-TG AG-----TTT GAAGGACTGA -----TGGTT G-AAAATC-- ---------- ---------- ---------- ---------- ----------
G. siphonifera IIa       TCC-CTG-TG AG-----TTT GAAGGACTGG -----TGGTT GCAAAATC-- ---------- ---------- ---------- ---------- ----------
G. siphonifera IIb       TCC-CTG-TG AG-----TTT GAAGGACTGG -----TGGTT GCATGACT-- ---------- ---------- ---------- ---------- ----------
G. calida                TCA-CTG-TG AG-----TTC AATGGACCGA -----TTTTT TCCC------ ---------- ---------- ---------- ---------- ----------
O. universa I            TTA-CTG-TG AG-----TTT AAAGGACCGA -----TCA-- ---------- ---------- ---------- ---------- ---------- ----------
O. universa III          ATA-CTG-CG AG-----TTT GAAGGACCAG -----GTCTC TGGCA----- ---------- ---------- ---------- ---------- ----------
G. sacculifer            GCA-CTA-CG AG-----TTT AAAGGCCCGA -----GAA-- ---------- ---------- ---------- ---------- ---------- ----------
G. ruber pink            TGG-CTG-CG AG-----TAA AAGGGACTTT -----CG--- ---------- ---------- ---------- ---------- ---------- ----------
G. ruber Ia              TGT-GTG-TG AG-----TAA GAGCGACTGT -----TA--- ---------- ---------- ---------- ---------- ---------- ----------
G. ruber Ib1             TGT-GTG-TG AG-----TAA GACGGACAAT -----TA--- ---------- ---------- ---------- ---------- ---------- ----------
G. ruber Ib2             TGT-GTG-TG AG-----TAA GACGGACAAT -----TA--- ---------- ---------- ---------- ---------- ---------- ----------
G. ruber IIa             TGA-CTG-TG AG-----TAG GACTGACCGT -----TTA-- ---------- ---------- ---------- ---------- ---------- ----------
G. conglobatus           TGA-CTG-TG AG-----TAG GCTGGATCGA ---------- ---------- ---------- ---------- ---------- ---------- ----------
G. rubescens (pink)      TGA-GTG-TG AG-----TAG GTCAGACTGA -----GTAAT TATTTAC--- ---------- ---------- ---------- ---------- ----------
G. bulloides Ia          ACT-TTG-CG AG-----AGT GTGGGACCTA -----TGAGC TTTCAAG--- ---------- ---------- ---------- ---------- ----------
G. bulloides Ib          ACT-TTG-CG AG-----ACT ATGG-ACTT- GA---AGCAT AGGATTGAAC TGC------- ---------- ---------- ---------- ----------
G. bulloides IIa         TCT-TTG-CG AG-----CGT GAGAGACTAA -----AAT-- ---------- ---------- ---------- ---------- ---------- ----------
G. bulloides IIb         TCT-TTG-CG AG-----AGT AAGAGACTTG -----TAATA ---------- ---------- ---------- ---------- ---------- ----------
G. bulloides IIc         TCT-TTG-CG AG-----AGT GAGAGACTGA -----AATGT ---------- ---------- ---------- ---------- ---------- ----------
G. bulloides IId         TCT-TTG-CG AG-----AGT GAGAGACTTA -----AAGAT A--------- ---------- ---------- ---------- ---------- ----------
G. bulloides IIe         TCT-TTG-TG AG-----AGT GAGGGACTGG ---------- ---------- ---------- ---------- ---------- ---------- ----------
T. quinqueloba Ia        TCG-TTG-TG AG-----TGA GCTGGACAAG -----TTATT T--------- ---------- ---------- ---------- ---------- ----------
T. quinqueloba Ib        TCG-TTG-TG AG-----TGA GCTGGACAAG -----TTATT T--------- ---------- ---------- ---------- ---------- ----------
T. quinqueloba IIa       TCG-TTG-TG AG-----ATA GCTGGACAAG -----TATTT A--------- ---------- ---------- ---------- ---------- ----------
T. quinqueloba IIb       TCG-TTG-TG AG-----ATA GCTGGACAAG -----TATTT A--------- ---------- ---------- ---------- ---------- ----------
T. quinqueloba IIc       TCG-TTG-TG AG-----ATA GCTGGACAAG -----TATTT A--------- ---------- ---------- ---------- ---------- ----------
T. quinqueloba IId       TCG-TTG-TG AG-----ATA GCTGGACAAG -----TATTT A--------- ---------- ---------- ---------- ---------- ----------
G. falconensis           TTT-TTG-CG AG-----TAT GATGGACTAA -----CTTTG CGCTCAATTT GAA------- ---------- ---------- ---------- ----------
H. pelagica              ACG-CTG-TG AG-----TTT AAGGGACTGG -----CTGTC ATTCAATGAT AAGC------ ---------- ---------- ---------- ----------
G. menardii              GTG-TTG-TG AG-----TAT TAGCGATAGA -----TCT-- ---------- ---------- ---------- ---------- ---------- ----------
G. ungulata              GTG-TTG-TG AG-----TAT TCGCGATAGA -----ACTAC TGACTAGACT ATACGATGTT GTTG------ ---------- ---------- ----------
G. hirsuta               TCT-TTG-TG AG-----TCT AAGGGACTGG -----GTAAT GATTCATTCT TTTCTAAAGT TTGAATTACC ---------- ---------- ----------
G. scitula               ACC-TTG-TG AG-----CCT GAGGGACTGG -----ATTTT TCTATTTCTT TAATAGATTT CTAATC---- ---------- ---------- ----------
G. truncatulinoides      CAC-TTG-TG AG-----GCT GAGGGACTGC -----GTCTA TTTATACCCG TGTAATCCTA ATATCTCACA TACATTTCGC TAAAGGCGAT TTTGCTTTAA
N. pachyderma I          TTT-TTG-TG AG-----TCT AAGGGACTGG -----GTTAA ACCACTCGTT TA-TT----- --CGGATGGC A--------- ---------- ----------
N. pachyderma II         TTT-TTG-TG AG-----TCT AAGGGACTGG -----GTTAA ACCACTCGTT TA-TT----- --CGGATGGC A--------- ---------- ----------
N. pachyderma III        TTT-TTG-TG AG-----TCT AAGGGACTGG -----GTTAA ACCACTCGTT TA-TTTAATA AGCGGATGGC A--------- ---------- ----------
N. pachyderma IV         TTT-TTG-TG AG-----TCT AAGGGACTGG -----GTTAA ACCATTCGTT C--TT----- --CGGATGGC A--------- ---------- ----------
N. pachyderma V          TTT-TTG-TG AG-----TCT AAGGGACTGG -----GTTAA ACCACTCGCT T----TATTA AGCGGATGGC A--------- ---------- ----------
N. pachyderma VI         TTT-TTG-TG AG-----TCT AAGGGACTGG -----GTTAA ACCACTCGCT TAATTTATTA AGCGGATGGC A--------- ---------- ----------
N. pachyderma VII        TTT-TTG-TG AG-----TCT AAGGGACTGG -----GTTAA ACCGCTCGTT TA-TT----- --CGGATGGC A--------- ---------- ----------
N. dutertrei_C           TCT-TTG-TG AG-----TCT AAGGGACTGG -----GTTAA TCTGTAATTC TATTATAG-- AT-------- ---------- ---------- ----------
N. dutertrei_Ib          TCT-TTG-TG AG-----TCT AAGGGACTGG -----GTTAA TCTGTAATTC TATTATGG-- AT-------- ---------- ---------- ----------
P. obliquiloculata_BR    TCT-TTG-TG AG-----TCT AAGGGACTGG -----GTTAA TAGCTATTTT TA-TAGTT-- AT-------- ---------- ---------- ----------
P. obliquiloculata_AS    TCT-TTG-TG AG-----TCT AAGGGACTGG -----GTTAA TATTTATTTT TAATAGGT-- AT-------- ---------- ---------- ----------
G. inflata               TCT-TTG-TG AG-----TCT TGGGGACTGG -----GTTAA GCGATTTCTT TTAGAATGAG CTC------- ---------- ---------- ----------
G. crassaformis          TCT-TTG-TG AG-----TCT TGGGGACTGG -----GTTAA GCGATTTCTT TTTGAATGAG CTC------- ---------- ---------- ----------
N. incompta I            CGT-TTG-TG AG-----TTT TAAGGACTGG -----ATTAA GCTATATGC- ---------- ---------- ---------- ---------- ----------
N. incompta II           CGT-TTG-TG AG-----TTT TAAGGACTGG -----ATTAA GCTATATGC- ---------- ---------- ---------- ---------- ----------
G. glutinata Ia1         TCT-CTG-TG AG-----TTT GAGGGACTGG -----ATTTG ATC-GCTTCG GCGCATTAAT AT-------- ---------- ---------- ----------
G. glutinata Ia2         TCT-CTG-TG AG-----TTT GAGGGACTGG -----ATTTG ATC-GCACTC GTGCGCATTA ACAT------ ---------- ---------- ----------
G. glutinata Ia3         TCT-CTG-TG AG-----TTT GAGGGACTGG -----ATTTG ATC-GCATTC GTGCGCATTA ACAT------ ---------- ---------- ----------
C. nitida                TCT-CTG-TG AG-----TTT GAGGGACT-G GGTTAA--CG CTT-CGGCGT GAA-C----- ---------- ---------- ---------- ----------
G. uvula                 TCT-CTG-TG AG-----TTT GAGGGACTGG -----AGCTT ACTTTACTGT GAGTCT---- ---------- ---------- ---------- ----------
B. variabilis            TCT-CTG-TG AG-----TTT GAGGGACTGT -----C--TT TGGCTGTT-A TA---CGT-C TCG--GCG-T ATACTACCAT CT-------- ----------
S. globigerus            TCT-CTG-TG AG-----TTT GAGGGACTGT -----C--TT TGGCTGTT-A TA---CGT-C TCG--GCG-T ATACTACCAT CT-------- ----------
B. alata                 TCT-CTG-TG AG-----TTT GAGGGACTGC -----TGTCT CGGTACTAAT ATTATCTCAC GATACATATT TACGCGCAT- ---------- ----------
G. vivans                TCT-CTG-TG AG-----TTT GAGGGACTGG -----GAACG CAGTGCTTTT TTGCTCTGCA CACC------ ---------- ---------- ----------
C. porrectus             TCT-CTG-TG AG-----TTT GAGGGACTGG -----TAAAT TAAATAATAT CATGTATTTA TACGTGTTTT ATTTGTCAC- ---------- ----------
C. ovoidea               TCT-CTG-TG AG-----TTT GAGGGACTGG -----GTAAT CTTTACACC- ---------- ---------- ---------- ---------- ----------
G. opercularis           TCT-CTG-TG AG-----TTT GAAGGACTGG -----CCTTC TGTGC----- ---------- ---------- ---------- ---------- ----------
E. aculeatum             TCG-CTA-TG AA-----TCT ATTAGACTGC -----GTTAT ACG------- ---------- ---------- ---------- ---------- ----------
E. vitrea                TCT-CTG-TG AG-----TTT GAGGGACTGG -----GAACG CAGGTTTTTT ATTAAATCTG CACACC---- ---------- ---------- ----------
H. germanica             TCT-CTG-TG AG-----TTT GAGGGACTGG -----ATTTT ATATC----- ---------- ---------- ---------- ---------- ----------
P. mediterranensis       TCT-CTG-TG AG-----TTT GAGGGACTGG -----GTTAC CTTAAATAAC C--------- ---------- ---------- ---------- ----------
S. fusiformis            TCT-CTG-TG AG-----TTT GAGGGACTGG -----GAACG CAGTGCTCTT TACTGAGCTT ACTGCACACC ---------- ---------- ----------
V. fragilis              TCT-CTG-TG AG-----TTT GAGGGACTGG -----GTATT TTTGCC---- ---------- ---------- ---------- ---------- ----------
A. pseudocassis          TCT-CTG-TG AG-----TTT GAGGGACTGG -----ATCTT GCATC----- ---------- ---------- ---------- ---------- ----------
Spiroplectammina sp.     TCT-CTG-TG AG-----TTT GAGGGACTGG -----GTATT AAGAAAAAAT TTATTTTATC TTT------- ---------- ---------- ----------
Textularia sp.           TCT-CTG-TG AG-----TTT GAGGGACTGG -----GTACT GCTATAAATT TATTTACTGC TATCACC--- ---------- ---------- ----------
S. limosum               ACT-CTG-TG AG-----TTT GAGGGACTGG -----TTTGA GTAGTTTTTT ATTAAATTAC ACAAAC---- ---------- ---------- ----------
G. antarctica            TCT-CTG-TG AG-----TTT GAGGGACTGG -----TATAT GATGCCACAG TTTTTTTTAT TCGTAAAAAA AATTGTCATC CCAC------ ----------
D. aphelis               TCT-CTG-TG AG-----TTT GAGGGACTGG -----GAACG CAGGAATTTA TTTCTGCACA CC-------- ---------- ---------- ----------
P. peruviana             ATA-TTA-TA AA-----TCT AAGGGACTTA -----AAGAA TATTTTATAT ATTT------ ---------- ---------- ---------- ----------
M. secans                ATA-TTA-TA AA-----TCT AAGGGACTTA -----AAATA TAATTTATTA TAT------- ---------- ---------- ---------- ----------
Quinqueloculina sp.      ATA-TTA-TA AA-----TCT AAGGGACTTA -----AAATA TATTTTATAT AT-------- ---------- ---------- ---------- ----------
N. haylinosphaira        TCT-CTG-TG AG-----TTT GAGGGACTGG -----GAAAC TCCTTTTC-- ---------- ---------- ---------- ---------- ----------
M. fusca                 TCT-TTG-TG AG-----TTT GAGGGACTGG -----AATAT TTCGGTATC- ---------- ---------- ---------- ---------- ----------
T. alba                  TCT-CTG-TG AG-----TTT GAGGGACTGG -----GTACC TTAGCTTTGT CGGTTAGGCG GTTTTTATTA ACTGCTTCTG ACACTATAAA CACT------
A. mexicana              TCT-CTG-TG AG-----TTT GAGGGACTGG -----GAAAA TATAACGAAT TTATTCGTTG TATCACC--- ---------- ---------- ----------
A. triangularis          ACT-CTG-TG AG-----TTT GAGGGACTGG -----TTTGA ATTTTAAAGT ATATGTATAT TTATTTATAT ATATTACTTT TTATATAATT CAGGC-----
A. rara                  ACT-CTG-TG AG-----TTT AAGGGACTGG -----TTTGA ATTAATATAA TTTTTTTAGA AAATTTATTT TTTATTATTA TATTATATTA TATTCAGGC-
E. scabrum               TCT-CTG-TG AG-----CTT GAGGGACTGA -----CTGCG TATCGCG--- ---------- ---------- ---------- ---------- ----------
N. venosus               TCT-CTG-TG AG-----TTT GAGGGACTGG -----GAACG CATATCTCTA TATGCACACC ---------- ---------- ---------- ----------
B. marginata             TCT-CTG-TG AG-----TTT GAGGGACTGG -----GAACG CGAAGTTATT TTTATAACAT ACGCACCTGC C--------- ---------- ----------
Trochammina sp.          TCT-CTG-TG AG-----TTT GAGGGACTGG -----ATAT- --TGTAATTT CGGTTACTAC CATC------ ---------- ---------- ----------
Peneroplis sp.           ATA-TTA-TA AA-----TCT AAGGGACATA -----CATAT TCTGAAATTG AATA------ ---------- ---------- ---------- ----------
S. orbiculus             ATA-TTA-TA AA-----TCT AAGGGATTTA -----TTATA ATTAAAAT-- ---------- ---------- ---------- ---------- ----------
Allogromia sp.           ATT-CTG-TG AG-----TAT ACAGGACGGG -----AACTC TATCTTCTGA TTGAGTTC-- ---------- ---------- ---------- ----------
```

```
                          1901       1911       1921       1931       1941       1951       1961       1971
                          |          |          |          |          |          |          |          |
G. siphonifera Ia1        ---------- -------TTT TTGGG-TTTG GAAAT-GCAG -TCAAA---- --CAGTA-CG ATTTAA-AGG AAAGAGAA
G. siphonifera Ia2        ---------- -------TTT TTGGG-TTTG GAAAT-GCAG -TCAAA---- --CAGTA-CG ATTTAA-AGG AAAGAGAA
G. siphonifera IIa1       ---------- ---------- ------ATTG GAAAT-TCTG -TCAAA---- --CAGCG-AG ATTTAA-AGG AAAGAGAA
G. siphonifera IIa2       ---------- ---------- ------ATTG GAAAT-TCTG -TCAAA---- --CAGCG-AG ATTTAA-AGG AAAGAGAA
G. siphonifera IIa3       ---------- ---------- ------ATTG GAAAT-TCTG -TCAAA---- --CAGCG-AG ATTTAA-AGG AAAGAGAA
G. siphonifera IIa        ---------- ---------- ------ATTG GAAAT-TCTG -TCAAA---- --CAGCG-AG ATTTAA-AGG AAAGAGAA
G. siphonifera IIb        ---------- ---------- ------ATTG GAAAT-TCTG -TCAAA---- --CAGCG-AG ATTTAA-AGG AAAGAGAA
G. calida                 ---------- ---------- ------TTTG GAAAT-TTGG -TCAAA---- --CAGTG-AG ATTTAA-AGG AAAGAGAA
O. universa I             ---------- ---------- ------TTTG GAAAT-TTAG -TCAAA---- --CAGAG-TT GTTTAA-AGG AAAGAGAA
O. universa III           ---------- ---------- ------CTTG GAAAT-TTAG -TCAAA---- --CAGAG-TT GTTTAA-AGG AAAGAGAA
G. sacculifer             ---------- ---------- ------ATTG GAAAT-TTAG -TCAAA---- --CAGTG-CT GTTTAA-AGG AAAGAGAA
G. ruber pink             ---------- ---------- -----TTTTG GAACT--TTG -TCGAA---- --CAGAT-GG GGCTAA-AGG AAAGAGAA
G. ruber Ia               ---------- ---------- -----AGAGG GAAAT--TTG -TCGAA---- --TGCAT-TT GGCTAA-AGG AAAGAGAA
G. ruber Ib1              ---------- ---------- -----AGCTG GAAAT--TTG -TCGAA---- --TGCAT-TG GGCTAA-AGG AAAGAGAA
G. ruber Ib2              ---------- ---------- -----AGCTG GAAAT--TTG -TCGAA---- --TGCAT-TG GGCTAA-AGG AAAGAGAA
G. ruber IIa              ---------- ---------- ------TCGG GAAAT--CCG -TCGAA---- --CAGTT-AG AGTTAA-AGG AAAGAGAA
G. conglobatus            ---------- ---------- -----AATGG GAAAG--CTG -TCAAA---- --CAGTT-AG AGTTAA-AGG AAAGAGAA
G. rubescens (pink)       ---------- ---------- ------TTGG G-AAA-TCTG -TCAAA---- --CACTC-AA ATTTAA-AGG AGAGAGAA
G. bulloides Ia           ---------- ---------- ----TACAGG G-AAC-CCAT -TCGAC---- --CAACG-GA GTTTAA-AGG AAAAAGAA
G. bulloides Ib           ---------- ---------- ----GAT-GG A-AAT-GTAT -TCGAT---- --CAACG-GA GTTTAA-AGG AAAAAGAA
G. bulloides IIa          ---------- ---------- ----ATCGCG G-AAC-TCAC -TCGAC---- --CGACG-GG ACTTAA-AGG AAAAAGAA
G. bulloides IIb          ---------- ---------- ----ATCGTG G-AAC-TCAC -TCGAC---- --CGACG-GG ATTTAA-AGG AAAAAGAA
G. bulloides IIc          ---------- ---------- -----GTGTG G-AAC-TCAC -TCGAC---- --CGACG-GG ACTTAA-AGG AAAAAGAA
G. bulloides IId          ---------- ---------- ----ATCGCG G-AAC-TCAC -TCGAC---- --CGACG-GG ATTTAA-AGG AAAAAGAA
G. bulloides IIe          ---------- ---------- --TGAACCAG G-AAC-TCAC -TCGAC---- --CAACG-GA ATTTAA-AGG AAAAAGAA
T. quinqueloba Ia         ---------- ---------- --TAACTTGA AAAGT-TC-- -TCAAT---- --CAAGG-TT TGCTAA-AGG AAAAAGAA
T. quinqueloba Ib         ---------- ---------- --TAACTTGA AAAGT-TC-- -TCAAT---- --CAAGG-TT TGCTAA-AGG AAAAAGAA
T. quinqueloba IIa        ---------- ---------- --CTACTTCA AAAGT-TC-- -TCAAT---- --CAAGG-TT TGCTAA-AGG AAAAAGAA
T. quinqueloba IIb        ---------- ---------- --CTACTTCA AAAGT-TC-- -TCAAT---- --CAAGG-TT TGCTAA-AGG AAAAAGAA
T. quinqueloba IIc        ---------- ---------- --CTACTTCA AAAGT-TC-- -TCAAT---- --CAAGG-TT TGCTAA-AGG AAAAAGAA
T. quinqueloba IId        ---------- ---------- --CTACTTCA AAAGT-TC-- -TCAAT---- --CAAGG-TT TGCTAA-AGG AAAAAGAA
G. falconensis            ---------- ---------- -TCTGTTGTG GAAAT-TCAC -TCGAA---- --CAACG-GG ATTTAA-AGG AAAAAGAA
H. pelagica               ---------- ---------- ------TATG GAAAC-TTAA -ACAAA---- --CAGTG-TG CTTTAA-AGG AAAGAGAA
G. menardii               ---------- ---------- ------TATC TAAAC-CTTC TACGAA---- --CAATG-TG G-CTAAGAGG AAAGAGAA
G. ungulata               ---------- ---------- ----TAGTCT AAAAC-GT-C TACGAA---- --CAATG-TG G-CTAAGAGG AAAGAGAA
G. hirsuta                ---------- ---------- ------TATA GAAAC-TTAG -CCGAA---- --CAAGG-TG GTTTAA-AGG AAAGAGAA
G. scitula                ---------- ---------- ------TATG GAAAC-TTAG -TCGAA---- --CAAGG-TG GTCTAA-AGG AAAGAGAA
G. truncatulinoides       GGATTTACAC GACCTTA--- ------CGCA CAAAC-TTAT -TCGAC---- --CAAGT-GG GTTTAA-AGG AAAGAGAA
N. pachyderma I           ---------- ---------- GTAACCTATG GAAAC-TTAT -GCGAA---- --CAAAG-TG GTCTAA-AGG AAAGAGAA
N. pachyderma II          ---------- ---------- GT-ACCTATG GAAAC-TTAT -GCGAA---- --CAAAG-TG GTCTAA-AGG AAAGAGAA
N. pachyderma III         ---------- ---------- GT-ACCTATG GAAAC-TTAT -GCGAA---- --CAAAG-TG GTCTAA-AGG AAAGAGAA
N. pachyderma IV          ---------- ---------- GT-ACCTATG GAAAC-TTAT -GCGAA---- --CAAAG-TG GTCTAA-AGG AAAGAGAA
N. pachyderma V           ---------- ---------- GT-ACCTATG GAAAC-TTAT -GCGAA---- --CAAAG-TG GTCTAA-AGG AAAGAGAA
N. pachyderma VI          ---------- ---------- GT-ACCTATG GAAAC-TTAT -GCGAA---- --CAAAG-TG GTCTAA-AGG AAAGAGAA
N. pachyderma VII         ---------- ---------- GT-ACCTATG GAAAC-TTAT -GCGAA---- --CAAAG-TG GTCTAA-AGG AAAGAGAA
N. dutertrei_C            ---------- ---------- AT-ACCTATG GAAAC-TTAT -ACGAA---- --CAATG-TG GTTTAA-AGG AAAGAGAA
N. dutertrei_Ib           ---------- ---------- AT-ACCTATG GAAAC-TTAT -ACGAA---- --CAATG-TG GTTTAA-AGG AAAGAGAA
P. obliquiloculata_BR     ---------- ---------- AT-ACCTATG GAAAC-TTAT -ACGAA---- --CAATG-TG GTTTAA-AGG AAAGAGAA
P. obliquiloculata_AS     ---------- ---------- AT-ACCTATG GAAAC-TTAT -ACGAA---- --CAATG-TG GTTTAA-AGG AAAGAGAA
G. inflata                ---------- ---------- -T-ACCTATG GAAAC-CAAT -ACGAA---- --CAATG-TG GTCTAA-AGG AAAGAGAA
G. crassaformis           ---------- ---------- -T-ACCTATG GAAAC-CAAT -ACGAA---- --CAATG-TG GTCTAA-AGG AAAGAGAA
N. incompta I             ---------- ---------- GTAACCTATG GAAAT-TCAT -GCGA----- --TGAAC-TG GTTTAA-AGG AAAGAGAA
N. incompta II            ---------- ---------- GTAACCTATG GAAAT-TCAT -GCGAA---- --TGAAC-TG GTTTAA-AGG AAAGAGAA
G. glutinata Ia1          ---------- ---------- -----CTATG GAAAC-TTAA -ACGAA---- --CAGTG-TG GTCTAA-AGG AAAGAGAA
G. glutinata Ia2          ---------- ---------- -----CTATG GAAAC-TTAA -ACGAA---- --CAGTG-TG GTCTAA-AGG AAAGAGAA
G. glutinata Ia3          ---------- ---------- -----CTATG GAAAC-TTAA -ACGAA---- --CAGTG-TG GTCTAA-AGG AAAGAGAA
C. nitida                 ---------- ---------- -----CTATG GAAAC-TTAA -ACGAA---- --CAGTG-TG GTCTAA-AGG AAAGAGAA
G. uvula                  ---------- ---------- -----CTATG GAAAC-TTAA -ACGAA---- --CAGTG-TG GTCTAA-AGG AAAGAGAA
B. variabilis             ---------- ---------- -------TAG GAAAC-TTAA -ACGAA---- --CAGTG-TG GTCTAA-AGG AAAGAGAA
S. globigerus             ---------- ---------- -------TAG GAAAC-TTAA -ACGAA---- --CAGTG-TG GTCTAA-AGG AAAGAGAA
B. alata                  ---------- ---------- -------TAG GAAAC-TTAA -ACGAA---- --CAGTG-TG GTCTAA-AG- --------
G. vivans                 ---------- ---------- ------TATG GAAAC-TTAA -ACGAA---- --CAGTG-TG GTCTAA-AGG AAAGAGAA
C. porrectus              ---------- ---------- ------TATA GAAAC-TTAA -ACGAA---- --CAGTG-GG GTCTAA-AGG AAAGAGAA
C. ovoidea                ---------- ---------- ------TATG GAAAC-TTAA -ACGAA---- --CAGTG-TG GTCTAA-AGG AAAGAGAA
G. opercularis            ---------- ---------- ------TATG GAAAN-TCAA -ACGAA---- --CAGTG-TG ATCTAA-AGG AAAGAGAA
E. aculeatum              ---------- ---------- ------CGCG GAAAG-ATAT -ATGAA---- --TAGTG-TG GTTTAA-AGG AAAGAGAA
E. vitrea                 ---------- ---------- ------TATG GAAAC-TTAA -ACGAA---- --CAGTG-TG GTCTAA-AGG AAAGAGAA
H. germanica              ---------- ---------- ------TATG GAAAC-TCAA -ACGAA---- --CAGTG-TG GTCTAA-AGG AAAGAGAA
P. mediterranensis        ---------- ---------- ------TATG GAAAC-TTAA -ACGAA---- --CAGTG-TG GTCTAA-AGG AAAGAGAA
S. fusiformis             ---------- ---------- ------CATG GAAAC-TTAA -ACGAA---- --CAGTG-TG GTCTAA-AGG AAAGAGAA
V. fragilis               ---------- ---------- ------TATG GAAAC-TTAA -ACGAA---- --CAGTG-TG GTCTAA-AGG AAAGAGAA
A. pseudocassis           ---------- ---------- ------TATG GAAAC-TTAA -ACGAA---- --CAGTG-TG GTCTAA-AGG AAAGAGAA
Spiroplectammina sp.      ---------- ---------- ACCACCTATG GGAAC-TTAA -ACGAA---- --CAGTG-TG GTCTAA-AGG AAAGAGAA
Textularia sp.            ---------- ---------- ------TACG GAAAC-TTAA -ACGAA---- --CAGTG-TG GTCTAA-AGG AAAGAGAA
S. limosum                ---------- ---------- ------TATA GAAAC-TTAA -ACGAA---- --CAGTG-TG GTCTAA-AGG AAAGAGAA
G. antarctica             ---------- ---------- ------TATA GAAAC-TTAA -ACGAA---- --CAGTG-GG GTCTAA-AGG AAAGAGAA
D. aphelis                ---------- ---------- ------TATG GAAAC-TTAA -ACRAA---- --CAGTG-TG GTCTAA-AGG AAARARAA
P. peruviana              ---------- ---------- ------TTCG GAAAC-TTAT -ATGCA---- --TAATG-TG ATTTAA-AGG AAAGAGAA
M. secans                 ---------- ---------- ------TTCG GAAAC-TTAT -ATGCA---- --TAATG-TG ATTTAA-AGG AAAGAGAA
Quinqueloculina sp.       ---------- ---------- ------TTCG GAAAC-TTAT -ATGCA---- --TAATG-TG ATTTAA-AGG AAAGAGAA
N. haylinosphaira         ---------- ---------- ------TATG GAAAC-TTAA -ACGAA---- --CAGTG-TG GTCTAA-AGG AAAGAGAA
M. fusca                  ---------- ---------- ------TATG GAAAC-TTAA -ACGAA---- --CAATG-TG GTCTAA-AGG AAAGAGAA
T. alba                   ---------- ---------- ------TATG GAAAC-TTAA -ACGAA---- --CAGTG-TG GTCTAA-AGG AAAGAGAA
A. mexicana               ---------- ---------- ------TATG GAAAC-TTAA -ACGAA---- --CAGTG-TG GTCTAA-AGG AAAGAGAA
A. triangularis           ---------- ---------- ------TATG GAAAC-TCAT -ACGAA---- --CAGTG-TG GTCTAA-AGG AAAGAGAA
A. rara                   ---------- ---------- ------TATG GAAAC-TTAT -ACGAA---- --CAGTG-TG GTCTAA-AGG AAAGAGAA
E. scabrum                ---------- ---------- -----AATG GAAAC-TTAA -ACGAA---- --CAGTG-TG GTCTAA-AGG AAAGAGAA
N. venosus                ---------- ---------- ------TATG GAAAC-TTAA -ACGAA---- --CAGTG-TG GTCTAA-AGG AAAGAGAA
B. marginata              ---------- ---------- ------TATG GAAAC-TTAA -ACGAA---- --CAGTG-TG GTCTAA-AGG AAAGAGAA
Trochammina sp.           ---------- ---------- ------TATG GAAAC-TTAA -ACGAA---- --CAGTG-TG GTCTAA-AGG AAAGAGAA
Peneroplis sp.            ---------- ---------- ------TATT GAAAC-TTAT -ATACA---- --TAATG-TG ATTTAA-AGG AAAGAGAA
S. orbiculus              ---------- ---------- ------ATAA AAAAC-TTAT -ATACA---- --TAATG-TG ATTTAA-AGG AAAGAGAA
Allogromia sp.            ---------- ---------- ------TATA AGAAT-GTAC -GCGAA---- --CAGTG-TG GTCTAA-AGG AAAGAGAA
```
