# Supplementary material for: Ecological partitioning and diversity in tropical planktonic foraminifera
Source: BMC Evol Biol. 2012 Apr 16;12:54. doi: 10.1186/1471-2148-12-54 (PMC3361484; doi:10.1186/1471-2148-12-54)
Supplement: Additional file 3 — Figure S2. SSU rDNA sequence alignments for four of the most common Arabian Sea planktonic foraminiferal morphospecies, showing the unambiguously aligned nucleotide sites used to reconstruct the phylogenies in Figure 3: Globigerinella siphonifera and Globigerinella calida (668 bp), Globigerinoides ruber and Globigerinoides conglobatus (589 bp), Globigerina bulloides (669 bp), and Turborotalita quinqueloba (748 bp). [file 1471-2148-12-54-S3.PDF]

Figure S2 - SSU rDNA sequence alignments for four of the most common Arabian Sea planktonic foraminiferal morphospecies

The unambiguously aligned nucleotide sites used to reconstruct the phylogeneies in Figure 3 are shown in grey. For full species names and GenBank accession numbers please see Table S1 (Additional file 1).

Globigerinella siphonifera and *Globigerinella calida* (668 bp)

```
                        1         11        21        31        41        51        61        71        81        91       100
                        |         |         |         |         |         |         |         |         |         |         |
G. siphonifera Ia1      GCACCACAAG AGCGTGGAGC ATGTGGCTTA ATTTGACTCA ACGCGGGGAA TCTTACCGGG TCCGGACACA CTGAGGATTG ACAGACA--- --GTT--GTC
G. siphonifera Ia2      GCACCACAAG AGCGTGGAGC ATGTGGCTTA ATTTGACTCA ACGCGGGGAA TCTTACCGGG TCCGGACACA CTGAGGATTG ACAGACA--- --GTT--GTC
G. siphonifera IIa1     GCACTACAAG AGCGTGGAGC ATGTGGCTTA ATTTGACTCA ACGCGGGGAA TCTTACCGGG TCCGGACACA CTGAGGATTG ACAGACA--- --ATT--GTC
G. siphonifera IIa2     GCACTACAAG AGCGTGGAGC ATGTGGCTTA ATTTGACTCA ACGCGGGGAA TCTTACCGGG TCCGGACACA CTGAGGATTG ACAGACA--- --ATT--GTC
G. siphonifera IIa3     GCACCACAAG AGCGTGGAGC ATGTGGCTTA ATTTGACTCA ACGCGGgGAA TCTTACCGGG TCCGGACACA CTGAGGATTG ACAGACA--- --ATT--GTC
G. siphonifera IIb      GCACTACAAG AGCGTGGAGC ATGTGGCTTA ATTTGACTCA ACGCGGGGAA TCTTACCGGG TCCGGACACA CTGAGGATTG ACAGACA--- --ATT--GTC
G. calida               GCACCACAAG AGCGTGGAGC ATGTGGCTTA ATTTGACTCA ACGCGGGGAA TCTTACCGGG TCCGGACATA CTGAGGATTG ACAGACA--- --TCACCACG

                        101       111       121       131       141       151       161       171       181       191       200
                        |         |         |         |         |         |         |         |         |         |         |
G. siphonifera Ia1      TTT-----CC CTCCC---TT CGGGGTGGGA TT-----ACA AAAGATAACA GATCTTTCAT GATCATGTGA TGGGTGGTGC ATGGCCGTTC TTAGTTCGTG
G. siphonifera Ia2      TTT-----CC CTCCC---TT CGGGGTGGGA TT-----ACA AAAGATAACA GATCTTTCAT GATCATGTGA TGGGTGGTGC ATGGCCGTTC TTAGTTCGTG
G. siphonifera IIa1     TTT--TTGTC AAATTAAA-- ---------- AG-----ACA AAAGATAACA GATCTTTCAT GATTATGTGA TGGGTGGTGC ATGGCCGTTC TTAGTTCGTG
G. siphonifera IIa2     TTT--TTGTC AAATTTAA-- ---------- AG-----ACA AAAGATAACA GATCTTTCAT GATTATGTGA TGGGTGGTGC ATGGCCGTTC TTAGTTCGTG
G. siphonifera IIa3     TTT--TTGTC AAATTTAA-- ---------- AG-----ACA AAAGATAACA GATCTTTCAT GATTATGTGA TGGGTGGTGC ATGGCCGTTC TTAGTtCGTG
G. siphonifera IIb      CTT--TTGGC -TTAAA---- ---------- AG-----ACA AAAGATAACA GATCTTTCAT GATTATGTGA TGGGTGGTGC ATGGCCGTTC TTAGTTCGTG
G. calida               TCTTTTTGTC CTTAAAA--- ---------- AG-----ACA AAAGATAACA GATCTTTCAT GATTATGTGG TGGGTGGTGC ATGGCCGTTC TTAGTTCGTG

                        201       211       221       231       241       251       261       271       281       291       300
                        |         |         |         |         |         |         |         |         |         |         |
G. siphonifera Ia1      GAGTGATCTG TCTGCTTAAT TGCGTTTCAA AT----TTTA TATTTGTTAA CATGCGAGTC TTCAATGACG GCCGCTCCAC CTTATAGACA TTATATGATA
G. siphonifera Ia2      GAGTGATCTG TCTGCTTAAT TGCGTTTCAA AT----TTTA TATTTGTTAA CATGCGAGTC TTCAATGACG GCCACTCCAC CTTATAGACA TTATATGATA
G. siphonifera IIa1     GAGTGATCTG TCTGCTTAAT TGCGTTTCAA AT----ATAT TTTATTATAT A-CGAGTCTA CAAA-----G GAGTGGTTCA TTAACAGACA ATCATATCTT
G. siphonifera IIa2     GAGTGATCTG TCTGCTTAAT TGCGTTTCAA AT----ATAT TTTATTATAT AACGAGTCTA CAAA-----G GAGTGGTTCA TTAACAGACA ATCATATCTT
G. siphonifera IIa3     GAGTGATCTG TCTGCTTAAT TGcGTTTCAA AT----ATAT TTTATTATAT AACGAGTCTA CAAA-----G GAGTGGTTCA TTAACAGACA ATCATATCTT
G. siphonifera IIb      GAGTGATCTG TCTGCTTAAT TGCGTTTCAA AT----ATAC TTTTTTTACA A-CGAGTCTA CAAA-----G GAGTGGTTCA GTTACAACAG ACAATTAATT
G. calida               GAGTGATCTG TCTGCTTAAT TGCGTTTCAA AT----GTAT TTTTGATTTC T-CAAACGAG TCTA-----C GAAGATGTCT ATTCATAACA AGCACTTCAT

                        301       311       321       331       341       351       361       371       381       391       400
                        |         |         |         |         |         |         |         |         |         |         |
G. siphonifera Ia1      TGCACATTTG TGTATTTGAT TATAACTTGT CTGGAGTCTG GCTCGATTTT TTTGA----- CTCAATTGAA CGCAACGGAC GTGATTGCAA GTCCTTGTTG
G. siphonifera Ia2      TGCACATTTG TGTATTTGAT TATAACTTGT CTGGAGTCTG GCTCGATTTT TTTGA----- CTCAATTGAA CGCAACGGAC GTGATTGCAA GTCCTTGTTG
G. siphonifera IIa1     TGAGCGTCTG G-AATCTACT CTATTT---- ---------- ---------- ---GG----- CTCAATTGAA CGCAACGGAC GTGATCGCGA GTCCTTGTTG
G. siphonifera IIa2     TGAGCGTCTG G-AATCTACT CTATTT---- ---------- ---------- ---GG----- CTCAATTGAA CGCAACGGAC GTGATCGCGA GTCCTTGTTG
G. siphonifera IIa3     TGAGCGTCTG G-AATCTACT CTATTT---- ---------- ---------- ---GG----- CTCAATTGAA CGCAACGGAC GtGATCGCGA GTCCTTGtTG
G. siphonifera IIb      CTTGTGTTTT G-AACGTTTG GAATGTACTC TATTT----- ---------- ---------- ---GG----- CTCAATTGAA CGCAACGGAC GTGATCGCAA GTCCTTGTTG
G. calida               TTATGATTTG T-TTGGAATA CGACTCTTTC ---------- ---------- ---GG----- CTCAATTGAA CGCAACGGAC GTGATTGCAA GTCCTTGTTG

                        401       411       421       431       441       451       461       471       481       491       500
                        |         |         |         |         |         |         |         |         |         |         |
G. siphonifera Ia1      AACAAATTAT ATATAT---- --A-CTACTT CATCATTA-- --GTATATAT AG--TTC--- GCTTCTC--- -ATGTCGTAG --CAGTCAAA CGGGCGGGCG
G. siphonifera Ia2      AACAAATTAT ATATAT---- --A-CTACTT CATCATTA-- --GTATATAT AG--TTC--- GCTTCTC--- -ATGTCGTAG --CAGTCAAA CGGGCGGGCG
G. siphonifera IIa1     AACTTCAG-T ATATAT---- -TGACTTTCC CCGTAGGAAG GGATTGGCAA TAATTGAATG TTCG--CTTT CTATTAGGTT TGTATGT--A ATCTCAAAAT
G. siphonifera IIa2     AACTTCAG-T ATATAT---- -TGACTATCC CCGTAGGAAG GGATTGGCAA TAATTGAATG TTCG--CTTT CTATTAGGTG TGTATATATA G-TCTCAAAA
G. siphonifera IIa3     AACTTCAG-T ATATAT---- -TGACTTTCC CCGTAGGAAG GGATTGGCAA TAATTGAATG TTCG--CTTT CTAT-AGGTG TGTATGATTA A-TCTCAAAA
G. siphonifera IIb      AACTTCAG-T ATATAT---- -TGACTTTCC CCGTAGGAAG GGATTGCCAA TAATTGAATG TTCG--CTTT CTATT-GGTG TGT------- GATCTCAAAA
G. calida               AACTTCTA-T ACATAT---- -CATTTACAT TCTTAACTGA GTGTATCTAA GATAATAGAG TTCG--CTTT CTATT-GGTG ACGGGATCTC CAATCACTTT

                        501       511       521       531       541       551       561       571       581       591       600
                        |         |         |         |         |         |         |         |         |         |         |
G. siphonifera Ia1      TCTTAATTGG CGCGGTCACG AGG---CTTA ATATGCTGCG GCAG------ GGAAAACTTG GGCGACCGCT GTAATACTTC TCTCTTAAAC CAGAGGAAGA
G. siphonifera Ia2      TCTTAATTGG CGCGGTCACG AGG---CTTA ATATGCTGCG GCTG------ GGAAAACTTG GGCGACCGCT GTAATACTTC TCTCTTAAAC CAGAGGAAGA
G. siphonifera IIa1     TACATGTATT TGTAATGGCG AGTGAT---C A--C--A-CC TATA------ GGAAAACTCG GGCGACCGCT GTAATATTTC TCT-TTAAAA CAGAGGAAGA
G. siphonifera IIa2     TTACATGTAT TTGTAATGGC GAGTAATATA TT-ACT-ATC CTGTA----- GGAAAACTCG GGCGACCGCT GTAATATTTC TCT-TTAAAA CAGAGGAAGA
G. siphonifera IIa3     TTACATGTAT TTGTAGTGGC GAGTATAATT T-A-CT-ATC CTATA----- GGAAAACTCG GGCGACCGCT GTAATATTTC TCT-TTAAAA CAGAGGAAGA
G. siphonifera IIb      TTGCATGTAT TTGTGATGGC GAGTGATCTA ATCCCTTA-- ---------- GGAAAACTTG GGCGACCGCT GTAATACTTC TCT-TTAAAA CAGAGGCAGG
G. calida               CGAGTGAGAG T--GAGTGAT CTAATCCTTT A--------- ---------- GGAAAACTCG GGCGACCGCT GTAATACTTT CTTTTTAAAA CAGAGGAAGA

                        601       611       621       631       641       651       661       671       681       691       700
                        |         |         |         |         |         |         |         |         |         |         |
G. siphonifera Ia1      TTGCGGCAAT AACAGGTCTG TGATGCCCTC AGATGTCCCG GGCCGCACAC GTGCTACATT GATTAGCGCA GTGCGCATAC TTACA-TATA GATCATT---
G. siphonifera Ia2      TTGCGGCAAT AACAGGTCTG TGATGCCCTC AGATGTCCCG GGCCGCACAC GTGCTACATT GATTAGCGCA GTGCGCATAC TTACA-TATA GATCATT---
G. siphonifera IIa1     TTGCGGCAAT AACAGGTCTG TGATGCCCTC AGATGTCCCG GGCCGCACAC GTGCTACATT GATTAGCGCA GTGCGCATAC TTTCAATATG ATACATT---
G. siphonifera IIa2     TTGCGGCAAT AACAGGTCTG TGATGCCCTC AGATGTCCCG GGCCGCACAC GTGCTACATT GATTAGCGCA GTGCGCATAC TTTCAATATG ATACATT---
G. siphonifera IIa3     TTGCGGCAAT AACAGGTCTG TGATGCCCTC AGaTGTCCCG GGCCGCACAC GTGCTACATT GAtTAGCGCA GTGCGCATAC TTTCAATATG ATACATT---
G. siphonifera IIb      TTGCGGCAAT AACAGGTCTG TGATGCCCTC AGATGTCCCG GGCCGCACAC GTGCTACATT GATTAGCGCA GTGCGCATAC TTTCAATATG ATACATT---
G. calida               TTGCGGCAAT AACAGGTCTG TGATGCCCTC AGATGTCCCG GGCCGCACAC GTGCTACATT GATTAGCGCA GTGCGCATAC TTTCAATATG ATACATT---
```

```
                          701        711        721        731        741        751        761        771        781        791      800
G. siphonifera Ia1        ---G-ATTGG -TTATTT--- ATAACCG--- ---------- ---------- -------TCA ATATCGCCTT GTCTGAAAGG A-----CTAG GTAATCTATT
G. siphonifera Ia2        ---G-ATTGG -TTATTT--- ATAACCG--- ---------- ---------- -------TCA ATATCGCCTT GTCTGAAAGG A-----CTAG GTAATCTATT
G. siphonifera IIa1       --AG-ATTGG ATAGCTT--- ---------T T--GCTTCCA T--------- -------CTA ATACTATCCG GCTTGAGAAG G-----CTGG GTAATCAATT
G. siphonifera IIa2       --AG-ATTGG ATTGTTCTG- ---------T TGAGCT-CCA T--------- -------CTA ATACTATCCG GCTTGAGAAG G-----CTGG GTAATCAATT
G. siphonifera IIa3       --AG-ATTGG ATTGTTTTA- ---------T TAAGCT-CCA T--------- -------CTA ATACTATCCG GCTTGAGAAG G-----CTGG GTAATCAATT
G. siphonifera IIb        --GG-ATTGG TGGTGAATTG TTGGCCCCGT CTAATACT-- ---------- ---------- -----GTCCT GTCTGAGAAG G-----CTGG GTAATCAATT
G. calida                 --GGTTTTGG TAGTAAGCTA TTCATCTGAT GGATATGCTA CTCTCCATAC CAATACT--- -----ATCTA GTCTGAAAAG A-----CTGG GTAATCTATT

                          801        811        821        831        841        851        861        871        881        891      900
G. siphonifera Ia1        GTAAGTGCTG GTTCCTCCTC CCGTTGAGCA TTTTAATAAT GGTCTCTCTA CATCCCTAGC A---CATGAT GTC---TAGT GCGATTGTAG TTGAGTCTTG
G. siphonifera Ia2        GTAAGTGCTG GTTCCTCCTC CCGTTGAGCA TTTTAATAAT GGTCTCTCTA CATCCCTAGC A---CATGAT GTC---TAGT GCGATTGTAG TTGAGTTCTG
G. siphonifera IIa1       GTAAGTGCTG GTTCCTCCTC CCGTTGAGCA TTTTAATAAT GGCCTCTCTA CATCCCTAGT A----AT-AT GAC---TAGT GCGATTGTAG TTGAGCCTTG
G. siphonifera IIa2       GTAAGTGCTG GTTCCTCCTC CCGTTGAGCA TTTTAATAAT GGCCTCTCTA CATCCCTAGC A----AT-AT GCC---TAGT GCGATTGTAG TTGAGCCTTG
G. siphonifera IIa3       GTAAGTGCTG GTTCCTCCTC CCGTTGAGCA TTTTAATAAT GGCCTCTCTA CATCCCTAGT A----AT-AT GCC---TAGT GCGATTGTAG TTGAGCCTTG
G. siphonifera IIb        GTAAGTGCTG GTTCCTACTC CCGTTGAGCA TTTTAATAAT GGCCTCTCTA CATCCCTAGT A----AC--- -CC---TAGT GCGATTGTAG TTGAGCCTTG
G. calida                 GTAAGTGCTG GTTCCTCTTC CCGTTGAGCA TTTTAATAAT GGTCTCTCTA CGTCCCTAGT A-----T-AT -TC---TAGT GCGATTGTAG TTGAGTCT-G

                          901        911        921        931        941        951        961        971        981        991      1000
G. siphonifera Ia1        CCATTTATGC AAGTGTCAAT TCTCAGTGGG GACAGCCATT TGATAATTCT TTGGCTCGGC CTCAACTAGG AATGCCTTGT ACGGGTCTTG GTTCAACAGA
G. siphonifera Ia2        CCATTTATGC AAGTGTCAAT TCTCAGTGGG GACAGCCATT TGATAATTCT TTGGCTCGGC CTCAACTAGG AATGCCTTGT ACGGGTCTTG GTTCAACAGA
G. siphonifera IIa1       CCATTTATGC AAGTGTCAAT TCTCAGTGGG GACAGACATT TGATAATTCT TTGTCTCGTT CTTAACTAGG AATGCCTTGT ACGGGTCTTG GTTCAACAGA
G. siphonifera IIa2       CCATTTATGC AAGTGTCAAT TCTCAGTGGG GACAGACATT TGATAATTCT TTGTCTCGTT CTTAACTAGG AATGCCTTGT ACGGGTCTTG GTTCAACAGA
G. siphonifera IIa3       CCATTTATGC AAGTGTCAAT TCTCAGTGGG GACAGACATT TGATAATTCT TTGTCTCGTT CTTAACTAGG AATGCCTTGT ACGGGTCTTG GTTCAACAGA
G. siphonifera IIb        CCATTTATGC AAGTGTCAAT TCTCAGTGGG GACAGACATT TGATAATTCT TTGTCTCGTT CTTAACTAGG AATGCCTTGT ACGGGTCTTG GTTCAACAGA
G. calida                 CCATTTATGC AAGTGTCAAT TCTCAGTGGG GACAGACATT TGATAATTCT TTGTCTCGTT CTTAACTAGG AATGCCTTGT ACGGGTCTTG GTTCAACAGA

                          1001       1011       1021       1031       1041       1051       1061       1071       1081       1091     1100
G. siphonifera Ia1        CCACCCGGAA TACGTCCCTG CCCTTTGTAC ACACCGCCCG TCGCTCTTAC CGATGAATTG TACTGTGAGT T----TGCAG GACCGAACCC A--------T
G. siphonifera Ia2        CCACCCGGAA TACGTCCCTG CCCTTTGTAC ACACCGCCCG TCGCTCTTAC CGATGAATTG TACTGTGAGT T----TGCAG GACCGAACCC A--------T
G. siphonifera IIa1       CCACCCGGAA TACGTCCCTG CCCTTTGTAC ACACCGCCCG TCGCTCTTAC CGATGAATTT CCCTGTGAGT T----TGAAG GACTGATGGT TGNAAAATC-
G. siphonifera IIa2       CCACCCGGAA TACGTCCCTG CCCTTTGTAC ACACCGCCCG TCGCTCTTAC CGATGAATTT CCCTGTGAGT T----TGAAG GACTGATGGT TGCAAAATC-
G. siphonifera IIa3       CCACCCGGAA TACGTCCCTG CCCTTTGTAC ACACCGCCCG TCGCTCTTAC CGATGAATTT CCCTGTGAGt T----TGAAG GACTGGTGGt TGCAAAATC-
G. siphonifera IIb        CCACCCGGAA TACGTCCCTG CCCTTTGTAC ACACCGCCCG TCGCTCTTAC CGATGACTTT CCCTGTGAGT T----TGAAG GACTGGTGGT TGCATGACT-
G. calida                 CCACCCGGAA TACGTCCCTG CCCTTTGTAC ACACCGCCCG TCGCTCTTAC CGATGAATTT CACTGTGAGT T----CAATG GACCGATTTT TTCCC-----

                          1101       1111       1121       1131       1141       1151
G. siphonifera Ia1        TTTTGGGT-- --TTGGAAAT GCAGTCAAAC AGTACGATTT AAAGGAAAGA GAA
G. siphonifera Ia2        TTTTGGGT-- --TTGGAAAT GCAGTCAAAC AGTACGATTT AAAGGAAAGA GAA
G. siphonifera IIa1       -------A-- --TTGGAAAT TCTGTCAAAC AGCGAGATTT AAAGGAAAGA GAA
G. siphonifera IIa2       -------A-- --TTGGAAAT TCTGTCAAAC AGCGAGATTT AAAGGAAAGA GAA
G. siphonifera IIa3       -------A-- --TTGGAAAT TCTGTCAAAC AGCGAGATTT AAAGGAAAGA GAA
G. siphonifera IIb        -------A-- --TTGGAAAT TCTGTCAAAC AGCGAGATTT AAAGGAAAGA GAA
G. calida                 -------T-- --TTGGAAAT TTGGTCAAAC AGTGAGATTT AAAGGAAAGA GAA
```

Globigerinoides ruber and *Globigerinoides conglobatus* (589 bp)

```
                          1          11         21         31         41         51         61         71         81         91       100
G. ruber pink             GCACCACAAG CGCGTGGAGC ATGTGGCTTA ATTTGACTCA ACGCGGGAAA TCTTACCGGG TCCGGACATA TCGAGGATTG ACAGACAGTT ------ACCA
G. ruber Ia               GCACCACAAG CGCGTGGAGC ATGTGGCTTA ATTTGACTCA ACNCGGGAAA TCTTACCGGG TCCGGACATA TCGAGGATTG ACAGACAGTT -----ATACA
G. ruber Ib1              GCACCACAAG CGCGTGGAGC ATGTGGCTTA ATTTGACTCA ACACGGGAAA TCTTACCGGG TCCGGACGTA TCGAGGATTG ACAGATAGTT -----ATACA
G. ruber Ib2              GCACCACAAG CGCGTGGAGC ATGTGGCTTA ATTTGACTCA ACACGGGAAA TCTTACCGGG TCCGGACGTA TCGAGGATTG ACAGATAGTT -----ATACA
G. ruber IIa              GCACCACAAG CGCGTGGAGC ATGTGGCTTA ATTTGACTCA ACACGGGAAA TCTTACCGGG TCCGGACATA CCGAGGATTG ACAGACTGTT ----------
G. conglobatus            GCACCACAAG CGCGTGGAGC ATGTGGCTTA ATTTGACTCA ACCGGGGAAA TCTTACCGGG TCCGGACATA CCGAGGATTG ACAGACAGTT ----------

                          101        111        121        131        141        151        161        171        181        191      200
G. ruber pink             TGCCCTTTCG AA-------- -----AGGAG TCAG-----G TTTTAAACCT AATGGCTCTT TCATGATTAT ATGATAGGTG GTGCATGGCC GTTCTTAGTT
G. ruber Ia               TGCCCT--AC AAGT------ -----AGGAG TCAG-----G TTTTAAACCT AATGGCTCTT TCATGATTAT ATGATAGGTG GTGCATGGCC GTTCTTAGTT
G. ruber Ib1              TGCCCT--AC AAGCAAGT-- -----AGGAG TCAG-----G TTTTAAACCT AATGGCTCTT TCATGATTAT ATGATAGGTG GTGCATGGCC GTTCTTAGTT
G. ruber Ib2              TGCCCT--AC AAGCAAGT-- -----AGgAG tCAG-----G TTTTAAACCT AATGGCTCTT TCATGATTAT ATGATAGGTG GTGCATGGCC GTTCTTAGTT
G. ruber IIa              ----AACTGC AACACATGCT GCTAA----- ---------G TTTTAAACTT AATAGCTCTT TCATGATTAT ATGATAGGTG GTGCATGGCC GTTCTTAGTT
G. conglobatus            ----AACTTG CCCTACCTAT -----AGGAG CTAG-----G TTCTAAACCT AATAGCTCTT TCATGATTAT ATGATAGGTG GTGCATGGCC GTTCTTAGTT

                          201        211        221        231        241        251        261        271        281        291      300
G. ruber pink             CGTGGAGTGA TCTGTCTGCT TAATTGCGTT TC-----AAA TTTT-TATTT ---------G AGGACCAGGT TTTGGTTTGG GTAGTGGTCC TACGATGACT
G. ruber Ia               CGTGGAGTGA TCTGTCTGCT TAATTGCGTT TC-----AAA ATTT-AACTT ---------G AGGACCGGGT TTTGGTTTGG CTAGTGGTTC TACGATGACT
G. ruber Ib1              CGTGGAGTGA TCTGTCTGCT TAATTGCGTT TC-----AAA ATTT-AACTT ---------G AGGACCGGGT TTTGGTTTGG CTAGTGGTCC TACGATGACT
G. ruber Ib2              CGTGGAGTGA TCTGTCTGCT TAATTGCGTT TC-----AAA TTTTTAACC- ---------G AGGACCGgGT TTTGGTTTGG CTAGTGGTCC TACGATGACT
G. ruber IIa              CGTGGAGTGA TCTGTCTGCT TAATTGCGTT TC-----AAA ATCTAAACCT ---------G AGCGACAAGG TGCTGCTATC GAAACTGTCT TTGAGTGTGA
G. conglobatus            CGTGGAGTGA TCTGTCTGCT TAATTGCGTT TC------AA ATATGAATTT GAGTACCAGG TCCCAAGTGT TTCAAGTAAA TCTGTCTCTG AGTGTGAACT

                          301        311        321        331        341        351        361        371        381        391      400
G. ruber pink             GTGAACTGTC ATGTCTTCGG ATGTGCGACT ATCGGTCTAG GATCGCATCT CA-------- GCCCCTGGGA CTCTTTTGAA CGCAACGGAC GTGATTGCAA
G. ruber Ia               GTGAACTGTC ATGTCTTCGG ATGTGCGACT ATTGGTCTAG GATCACATGA CTT------- GCCCCTGGGA CTCTTTTGAA CGCAACGGAC GTGATTGCAA
G. ruber Ib1              GTGAACTGTC ATGTCTTCGG ATGTACGACT ATCGGTCTAG GATCACATGA CTT------- GCCCCTGGGA CTCTTTTGAA CGCAACGGAC GTGATTGCAA
```

```
G. ruber Ib2        GTGAACTGTC ATGTCTTCGG ATGTACGACT ATCGGTCTAG GATCACATGA CTT------- GCCCCTGGGA CTCTTTTGAA CGCAACGGAC GTGATTGCAA
G. ruber IIa        ACTATAAGTC TTTTGACTTA TGACCATCAC TCTAGACGTT TATGTTTTGG TGTTG----- GCCCTTGATG CTCATTTGAA CGCAACGGAC GTGATTGCAA
G. conglobatus      GTATCTCTTC GGATATGCGA CTATCACTCT TAGGCAACTA TGATCTGCTT GTA------- GCCCCTGGTA CTCTTTTGAA CGCAACGGAC GTGATTGCAA

                    401        411        421        431        441        451        461        471        481        491       500
                    |          |          |          |          |          |          |          |          |          |         |
G. ruber pink       CCCCTTGTTG AG-----AT- TAGAGG---- -----AGTCT CGATATTCAT TGGCC--AAA CTTACCGCTT CGAGCTTGCT CGTGCAGTTT TGTTAGCCG-
G. ruber Ia         CCCCTTGTTG AG-----AT- TAGAGAT--- -----AGTCT CGATATTCAC TGGAC--AAA CTGGCTGCTT TATGCAGTCT TGTTGAAT-- --------G-
G. ruber Ib1        CCCCTTGTTG AG-----AT- TAGAGAT--- -----AGTCT CGATATTCAC TGGCC--AAA CTGGCTGCTT TATGCAGTCT TGTTGAAT-- --------G-
G. ruber Ib2        CCCCTTGTTG AG-----AT- TAGAGAT--- -----AGTCT CGATATTCAC TGGCC--AAA CTGGCTGCTT TATGCAGTCT TGTTGAAT-- --------G-
G. ruber IIa        CCCCTTGTTG AG-----ATT TATCCGATCT CTACGTTCAC TGAACCACAC TCTTTAGCGA GAGCAGTGGG GA-------- ---------- --------T-
G. conglobatus      CCCCTTGTTG AG-----TTT GCTTCTCTTA GGAACTCTAC GTCTACTGAT CCACCTACCT TAGCGGGTAG GTGGTGA--- ---------- --------G-

                    501        511        521        531        541        551        561        571        581        591       600
                    |          |          |          |          |          |          |          |          |          |         |
G. ruber pink       ----GTGGAT AAACTCGGGG GACTGCTGAC TATAACCATT TCTCAAAACA GAGGAAGGTT GCGGCAATAA CAGGTCTGTG ATGCCCTTAG ATGTCCCGGG
G. ruber Ia         ----GTGGAT AAACTCGGGG GACTGCTGAC TATAACCATT TCTCAAAACA GAGGAAGGTT GCGGCAATAA CAGGTCTGTG ATGCCCTCAG ATGTCCCGGG
G. ruber Ib1        ----GTGGAT AAACTCGGGG GACTGCTGAC TATAACCATT TCTCAAAACA GAGGAAGGTT GCGGCAATAA CAGGTCTGTG ATGCCCTCAG ATGTCCCGGG
G. ruber Ib2        ----GTGGAT AAACTCGGGG GACTGCTGAC TATAACCATT TCTCAAAAcA GAGGAAGGTT GCGGCAATAA CAGGTCTGTG ATGCCCTCAg ATGTCCCGGG
G. ruber IIa        ----GTGGAT AAACTCGGGG GACTGCTGAC TATAAACTTT TCTCAAACCA GAGGAAGGTT GCGGCAATAA CAGGTCTGTG ATGCCCTCAG ATGTTCCGGG
G. conglobatus      ----GTGGAT AAACTCGGGG GACTGCTGAC TATAACCATT TCTCAAAACA AAGGAAGGTT GCGGCAATAA CAGGTCTGTG ATGCCCTCAG ATGTCCCGGG

                    601        611        621        631        641        651        661        671        681        691       700
                    |          |          |          |          |          |          |          |          |          |         |
G. ruber pink       CTGCACACGT GCTACAATGA GT-GGCGCAA TGTGT----- GCAATTTTGT -AAGGG-ATA AGAGTGT--- G-G-TCAGT- ---ATG-CCG --TCGGGGAT
G. ruber Ia         CTGCACACGT GCTACAATGA GT-GGCGCAA TGTGC----- TAATGTTT-- -AAGGG-ATA AGAGTGT--- G-G-TCAGT- ---AGA-ACG --TCGGG-AT
G. ruber Ib1        CTGCACACGT GCTACAATGA GTTGGCGCAA TGTGC----- TAATGTTT-- TAAGGG-ATA AGCGTGC--- G-G-CCAGT- ---AGA-ACG --TCGGGAAA
G. ruber Ib2        CTGCACACGT GCTACAATgA GT-GGCGCAA TGTGC----- TAATGTTT-- TAAGGG-ATA AGCGTGC--- G-G-CCAGT- ---AGA-ACG --TCGGC-AA
G. ruber IIa        CTGCACACGT GCTACATTTA GT-AGCGCAG TGTGC----- ACATTAAT-- AGAGTG-ATA GGTTCCGTGG TTTG--TACA TTTTAAGGGC TCGTCCTAGT
G. conglobatus      CTGCACACGT GCTACAATGA GT-AGCGCAG TGTGC----- ACATTTT--- -GAAGAGTGA TA-GGTGCTG GGGTTGTACA TTTTAAAGGG CTCGTCCTAG

                    701        711        721        731        741        751        761        771        781        791       800
                    |          |          |          |          |          |          |          |          |          |         |
G. ruber pink       TCCCCCTG-A CG--TCTC-C TAGCACACGA TT-----ATC AGCCTTTCCT TAACAGGGCG GGTAACCTTT TCAAAATACC AGTTTGA--- --TTTT-A--
G. ruber Ia         TTCCCCTG-A CG--TCTC-C CCCGCA-CGA TT-----ATC AGCCTTCCCT TAACAGGGCG GGCAACCTTT TCAAAATACT GGTTTGA--- --TGTT-A--
G. ruber Ib1        TTCCCCTG-- CG--TCGCC- TGCGCA-CGA TT-----ATC AGCCTTCCCT TAACAGGGCG GGCAACCTTT TCAAAATACC GGTTTGA--- --TATT-A--
G. ruber Ib2        TTGCCCTG-G CG--TCTTCT ACCGCA-CGA TT-----ATC AGCCTTCCCT TAACAGGGCG GGCAACCTTT TCAAAATACC GGTTTGA--- --TGTT-A--
G. ruber IIa        AACCAAAGCT CCACGGGACA T--------- -------ATC AACCTTCCCC TTACAGGGCG GGTAACCTCT TCGAAGTGCT GGCATGA--- --TTTCCCTA
G. conglobatus      TAACCAACCG CCCCC-AAGC ATCT------ -------ATC AACCTTCCCT TTACAGGGTG GGTAACCTCT TCGAAGTGCT GGTTTGA--- --TTTCCC-T

                    801        811        821        831        841        851        861        871        881        891       900
                    |          |          |          |          |          |          |          |          |          |         |
G. ruber pink       ---A--AGC- --TTTGGGTC A-ATTAG-GG TG-AT---GG TTCGATAG-A ACCAATATCA -CCCAGCTGC TA------CT CGTGCGCGCG TG-T------
G. ruber Ia         ---A--AGT- --ATTGAGTC G-TTTTTTTT GGAT----GG TGGTTTGTTA ACCAATACCA TCCGGCAACT GC-------T CGCGAGTTTG TGGTT-----
G. ruber Ib1        ---A--AGT- --ATAGAGTC G-TTTTTTTT GGAT----GG TGGTTTACGA ACCAATACCA TCCG-CAACT GC-------T CGCGAGTTTG TGGTT-----
G. ruber Ib2        ---A--AGT- --ATaGAGTC G-TTTTTTTT GGAT----GG TGGTTTACGA ACCAATACCA TCCG-CAACT GC-------T CGCGAGTTTG TGGTT-----
G. ruber IIa        GTTTTGGCAT TTGAATCCTT GTCGCAGCAG CCCTGAGACG TAGTGAACGC TGCAAAGTGA TTCCAGTCTT AGGTTTGC-- ---------- ----------
G. conglobatus      AGTGTTTGTC ATAATAATCC TCGTCGCACC TACTCTAGGA CATAGTGAAC ATTGTAGACT TTGTCATAAT GATTCCCGTC TTAGGTTTGC GACGCATGCT

                    901        911        921        931        941        951        961        971        981        991       1000
                    |          |          |          |          |          |          |          |          |          |         |
G. ruber pink       -------TAT TC-------- -CTGG-TGAC TCATGGTGGG GACCGACATT TGTAATTGTC TGTCGCG-TG TTAACCGGGA ATGCCTTGTA CTGTCGATTC
G. ruber Ia         ---------- ---------- -CTGG-CGAC TCATGGTGGG GACCGACGTT TGTAATTTTT TGTCGCG-TG TTAACTAGGA ATGCCTTGTA CTGTCGATTC
G. ruber Ib1        ---------- ---------- -CTGG-TGAC TCATGGTGGG GACCGATGTT TGTAATTGTT TGTCGCG-TG TCAACTAGGA ATGCCTTGTA CTGTCGATTC
G. ruber Ib2        ---------- ---------- -CTGG-TGAC TCATGGTGGG GACCGATGTT TGTAATTGTT TGTCGCG-TG TCAACTAGGA ATGCCTTGTA CTGTCGATTC
G. ruber IIa        --GACACATG CAATTC---- -CTGGTTGAC TCATCGTGGG GACTGATTCT TGTAATTATT TTTCACGG-T TCAACCAGGA ATGCCTTGTA CCGGCGGCTC
G. conglobatus      TT-------- ----TC---- -CTGGTTGAC TCATCGTGGG AACTGATTCT TGTAATTATT TGTCACGG-T TCAACCAGGA ATGCCTTGTA CTGGCGGCTC

                    1001       1011       1021       1031       1041       1051       1061       1071       1081       1091      1100
                    |          |          |          |          |          |          |          |          |          |         |
G. ruber pink       ACTAAGTTAT GGGGAATACG TCCCTGCCCT TTGTACACAC CGCCCGTCGC TCTTACCGAT GGCTTTGGCT GCGAGTAA-- ---AAGGGAC TTTCG-TTTT
G. ruber Ia         ACTAAGTTAC TGGGAATACG TCCCTGCCCT TTGTACACAC CGCCCGTCGC TCTTACCGAT GGCTGTGTGT GTGAGTAA-- ---GAGCGAC TGTTA-AGAG
G. ruber Ib1        ACTAAGTTAT TGGGAATACG TCCCTGCCCT TTGTACACAC CGCCCGTCGC TCTTACCGAT GGCTTTGTGT GTGAGTAA-- ---GACGGAC AATTA-AGCT
G. ruber Ib2        ACTAAGTTAT TGGGAATACG TCCCTGCCCT TTGTACACAC CGCCCGTCGC TCTTACCGAT GGCTTTGTGT GTGAGTAA-- ---GaCGGAC AATTA-AGCT
G. ruber IIa        ATTAAACCGC TGGGAATACG TCCCTGCCCT TTGTACACAC CGCCCGTCGC TCTTACCGAT GACTTTGACT GTGAGTAG-- ---GACTGAC CGTTTA-TCG
G. conglobatus      ATTAAACCGC TCGGTATACG TCCCTGCCCT TTGTACACAC CGCCCGTCGC TCTTACCGAT GACTTTGACT GTGAGTAG-- ---GCTGGAT CGA---AATG

                    1101       1111       1121       1131
                    |          |          |          |
G. ruber pink       GGAACTTT-- ---GTCGAAC AGATGGGGCT AAAG
G. ruber Ia         GGAAATTT-- ---GTCGAAT GCATTTGGCT AAAG
G. ruber Ib1        GGAAATTT-- ---GTCGAAT GCATTGGGCT AAAG
G. ruber Ib2        GGAAATTT-- ---GTCGAAT GCATTGGGCT AAAG
G. ruber IIa        GGAAATCC-- ---GTCGAAC AGTTAGAGTT AAAG
G. conglobatus      GGAAAGCT-- ---GTCAAAC AGTTAGAGTT AAAG
```

Globigerina bulloides (669 bp)

```
                    1          11         21         31         41         51         61         71         81         91        100
                    |          |          |          |          |          |          |          |          |          |         |
G. bulloides IIa    GCACCACAAG AGCGTGGAGT ATGTGGCTTA ATTTGACTCA ACGCGGAAAA GCTTATCTGG TCCGGACACA GTGAGGATTG ACAGAC---- -GGTC----T
G. bulloides Ib     GCACCACAAG AGCGTGGAGT ATGTGGCTTA ATTTGACTCA ACGCGGAAAA GCCTATTTGG TCCGGACACA GTGAGGATTG ACAGAC---- -ATGT---CG
G. bulloides IIa    GCACCACAAG AGCGTGGAGT ATGTGGCTTA ATTTGACTCA ACGCGGAAAA GCTTATCTGG TCCGGACGCA GTGAGGATTG ACAGAC---- -AGTT----T
G. bulloides IIb    GCACCACAAG AGCGTGGAGT ATGTGGCTTA ATTTGACTCA ACGCGGAAAA GCTTATCTGG TCCGGACGCA GTGAGGATTG ACAGAC---- -AGTTAGA--
G. bulloides IIc    GCACCACAAG AGCGTGGAGT ATGTGGCTTA ATTTGACTCA ACGCGGAAAA GCTTATCTGG TCCGGACACA GTGAGGATTG ACAGAC---- -AGTT---GG
```

```
G. bulloides IId    GCACCACAAG AGCGTGGAGT ATGTGGCTTA ATTTGACTCA ACGCGGAAAA GCTTATCTGG TCCGGACACA GTGAGGATTG ACAGAC---- -AGTTAGA--
G. bulloides IIe    GCACCACAAG AGCGTGGAGT ATGTGGCTTA ATTTGACTCA ACGCGGAAAA GCTTATCTGG TCCGGACACA GTGAGGATTG ACAGAC---- -GGTT----T

                    101        111        121        131        141        151        161        171        181        191       200
                    |          |          |          |          |          |          |          |          |          |         |
G. bulloides IIa    TATTGGTGGA CTCT-AAGAC GTC----ATG GTAA-----A GTTGAAGTTC TTTCATGATC TTGTGGCAGG TGGTGCATGG CCGTTCTTAG TTCGTGTAGT
G. bulloides Ib     TATTGGTGGA CTTT-ATGA- ---CAAAATG GTAA-----A GTTGAAGTTC TTTCATGATC TTGTGGTAGG TGGTGCATGG CCGTTCTTAG TTCGTGTAGT
G. bulloides IIa    CAGGAGTGGT TCTT-GGTAA A--CAA-TTG AGAG-----A GTTGAAGTTC TTTCATGATC TTGTGAGAGG TGGTGCATGG CCGTTCTTAG TTCGTGTAGT
G. bulloides IIb    CAGAAGTGGT TTA--GGTAA ---CAA-TTG AGAG-----A GTTGAAGTTC TTTCATGATC TTGTGAGAGG TGGTGCATGG CCGTTCTTAG TTCGTGTAGT
G. bulloides IIc    CAGGAGTGGT TCTT-GGTAA AAACAA-ATG AGAG-----A GTTGAAGTTC TTTCATGATC TTGTGAGAGG TGGTGCATGG CCGTTCTTAG TTCGTGTAGT
G. bulloides IId    CAGAAGTGAG GTTTTGGTAA ---CAA-TTG AGAG-----A GTTGAAGTTC TTTCATGATC TTGTGAGAGG TGGTGCATGG CCGTTCTTAG TTCGTGTAGT
G. bulloides IIe    TATTAGTGGT TTCT-AAAAC ACA----TTG AGAA-----A GTTGAAGTTC TTTCATGATC TTGTGAGAGG TGGTGCATGG CCGTTCTTAG TTCGTGTAGT

                    201        211        221        231        241        251        261        271        281        291       300
                    |          |          |          |          |          |          |          |          |          |         |
G. bulloides IIa    GATACGTCTG CCTAATCGCG TCACGATAAC CTATTTCTCG ACAATCCAA- ----AGATCT TCG-TGCAGT ATCTATTGGT ACGGTGCTCA CGTTA-----
G. bulloides Ib     GATACGTCTG CCTAATCGCG TCACGATAAG CTATTTCTCG ACAATCCAA- ----AAATCA CGGCGGGAGG TTCTACTGGT ACGGGTTCCC AAA-----GT
G. bulloides IIa    GATATGTCTG CCTAATCGCG TCACGATAAC CTATTGGTCG ACAACCCAA- ----TTATCA CAACTGCAGC ATAACTCCCC TTGGGTGGGC GAGGCTCTGT
G. bulloides IIb    GATATGTCTG CCTAATCGCG TCACGATAAC CTATTGGTCG ACAACCCAA- ----TTATCA CAACTGCAGC ATAACTCCCC TTGGGTGGGC GAGGCTCTGT
G. bulloides IIc    GATATGTCTG CCTAATCGCG TCACGATAAC CTATTGGTCG ACAACCCAA- ----TTATCA CAACTGCAGC ATAACTCCCC TTGGGTGGGC GAGGCTCTGT
G. bulloides IId    GATATGTCTG CCTAATCGCG TCACGATAAC CTATTGGTCG ACAACCCAA- ----TTATCA CAACTGCAGC ATAACTCCCC TTGGGTGGGC GAGGCTCTGT
G. bulloides IIe    GATATGTCTG CCTAATCGCG TCACGATAAC CTATTGGTCG ACAACCCAA- ----TTATCA CAACTGCAGC ATAACTCCCC TTGGATGGGC GAGGCTCTGT

                    301        311        321        331        341        351        361        371        381        391       400
                    |          |          |          |          |          |          |          |          |          |         |
G. bulloides IIa    -----GTAGG CTAGAGATTT GAACAGTACG CAACGGACGC GATCGTAATC TCTTGTTAAG TGGCCATCCT GTGAGC---- -CCCTGATTT AATGG---CAG
G. bulloides Ib     -----GTAGG CTAGAGACTT GAACAGTACG CAACGGACGT GATCGTAATC TCTTGTTAAA TGGTTATCCT GTGAGC---- -CAACCGTGA CTGATTACAG
G. bulloides IIa    -----GTAGG ATAGACCTCT GAACAGTACG CAACGAACGC GATCGTAATC CCTTGTTGAG TGGCCATCCT GTAAGC---- -TGCTGGATT AGGAACCCAG
G. bulloides IIb    -----GTAGG ATAGACCTCT GAACAGTACG CAACGAACGC GATCGTAATC CCTTGTTGAG TGGCCATCCT GTAAGC---- -TGCTGGATT AGGAACCCAG
G. bulloides IIc    -----GTAGG ATAGACCTCT GAACAGTACG CAACGAACGC GATCGTAATC CCTTGTTGAG TGGCCATCCT GTAAGC---- -TGCTGGATT TGAAACCCAG
G. bulloides IId    -----GTAGG ATAGACCTCT GAACAGTACG CAACGAACGC GATCGTAATC CCTTGTTGAG TGGCCATCCT GTAAGC---- -TGCTGGATT AGGAACCCAG
G. bulloides IIe    -----GTAGG ATAGACCTCT GAACAGTACG CAACGAACGC GATCGTAATC CCTTGTTGAG TGGCCATCCT GTAAGC---- -C-CTGGAGT CGTC---CAG

                    401        411        421        431        441        451        461        471        481        491       500
                    |          |          |          |          |          |          |          |          |          |         |
G. bulloides IIa    G-----CGGT ATCATC---T CAGCCACATT TCCTCTGGTA GTAGT---GG GCCAG----A TTTA---AAA CTCGAGAAAC ATCTGTGACT TTCTTTCTTT
G. bulloides Ib     CACACCTGGT ATCATC---- CAGACCATAT -TGGTTTAGT GGGTC---GG GCTAG---AT GTCG---AAA CTCGGGAAAC ATCTGTGACT TTCTTTCTTT
G. bulloides IIa    ------TGGT ATTATC---T CAGCCACAGA TTTTCTGGTT GTAAT---GG GCCAG----T TTTG---AAA CTCGGGGAAC ATCTGTGACT TTCTTTCTTA
G. bulloides IIb    ------TGGT ATTATC---T CAGCCACAGA TTTTCTGGTT GTAGT---GG GCCAG----T TTTG---AAA CTCGGGGAAC ATCTGTGACT TTCTTTCTTA
G. bulloides IIc    ------TGGT ATTATC---T CAGCCACAGA TTTTCTGGTT GTAAT---GG GCCAG----T TTTG---AAA CTCGGGGAAC ATCTGTGACT TTCTTTCTTA
G. bulloides IId    ------TGGT ATTATC---T CAGCCACAGA TTTTCTGGTT GTAGT---GG GCCAG----T TTTG---AAA CTCGGGGAAC ATCTGTGACT TTCTTTCTTA
G. bulloides IIe    ------TGGT ATTATC---T CAGCCACAGA TCTTCTGGTT GTAGT---GG TCCAG---TT GTTG---AAA CTCGGGGAAC ATCTGTGACT TTCTTTCTTA

                    501        511        521        531        541        551        561        571        581        591       600
                    |          |          |          |          |          |          |          |          |          |         |
G. bulloides IIa    ACGCAGAGGA AGGTTATGGC AATAACAGGT CTGTGATGCC CTCAGATGTC CAGAGCTGCA CACGTACTAC AGTGATC--- --GACTCACT AAGTGTCGT-
G. bulloides Ib     ACGCAGAGGA AGGTTATGGC AATAACAGGT CTGTGATGCC CTCAGATGTC CAAAGCTGCA CACGTACTAC AGTGATC--- --GTAACACT T-GCGTTGTT
G. bulloides IIa    ACGCAGAGGA AGGTTATGGC AATAACAGGT CTGTGATGCC CTTAGATGTC CAGGGCTGCA CACGTACTAC ATTGATC--- --AACTCAGT AGGCGTCGT-
G. bulloides IIb    ACGCAGAGGA AGGTTATGGC AATAACAGGT CTGTGATGCC CTTAGATGTC CAGGGCTGCA CACGTACTAC ATTGATC--- --AACTCAGT AGGCGTCGT-
G. bulloides IIc    ACGCAGAGGA AGGTTATGGC AATAACAGGT CTGTGATGCC CTTAGATGTC TAGGGCTGCA CACGTACTAC ATTGATC--- --AACTCAGT AGGCGTCGT-
G. bulloides IId    ACGCAGAGGA AGGTTATGGC AATAACAGGT CTGTGATGCC CTTAGATGTC CAGGGCTGCA CACGTACTAC ATTGATC--- --AACTCAGT AGGCGTCGT-
G. bulloides IIe    ACGCAGAGGA AGGTTATGGC AATAACAGGT CTGTGATGCC CTTAGATGTC CAGGGCTGCA CACGTACTAC ATTGATT--- --AACTCAGT AGGCGTCGT-

                    601        611        621        631        641        651        661        671        681        691       700
                    |          |          |          |          |          |          |          |          |          |         |
G. bulloides IIa    GTT--TT--- --CTCCAAAT AACGTATACA GTGGACTTGG TGTCGGGTGC -TGGCCTCTG GTCATGTGCT TTGATTACTG T-CACTTTAA ACACTGGTCG
G. bulloides Ib     GTTAG----- --CTCC-ATT AACGTATTGA GTGGACTTGG TGTCGGGT-C -TGGCCTCTG GTCATGTGCT TTGATTACTG AACACTTTAA ACACTGGTCG
G. bulloides IIa    GTT--TT--- --CTCC-AAT AACGTATCTA GTGGACTTGG TGTCGGGTGC GTGGCCTC-G GTCGTGTACT TTGATTACTG T-CACTTTAA ACACTGGTCG
G. bulloides IIb    GTTAGTT--- --CTCC-AAT AACGTATCTA GTGGACTTGG TGTCGGGTAT GTGGCCTC-G GTCATGTACT TTGATTACTG T-CACTTTAA ACACTGGTCG
G. bulloides IIc    GCT--TT--- --CTCC-AAT AACGTATCTA GTGGACTTGG TGTCGGGTGC GTGGCCTC-G GTCGTGTACT TTGATTACTG T-CACTTTAA ACACTGGTCG
G. bulloides IId    GTTAGTT--- --CTCC-AAT AACGTATCTA GTGGACTTGG TGTCGGGTGT GTGGCCTCTG GTCATGCGCT TTGATTACTG T-CACTTTAA ACACTGGTCG
G. bulloides IIe    GTT--TT--- --CTCC-AAT AACGTATCTA GTGGACTTGG TGTCGGGTGC -TGACCTCTG GTCTGGTACT TTGATTACTG T-CACTTTAA ACACTGGTCG
```

***Turborotalita quinqueloba* (748 bp)**

```
                      1          11         21         31         41         51         61         71         81         91        100
                      |          |          |          |          |          |          |          |          |          |         |
T. quinqueloba Ia     GCACCACAAG AGCGTGGAGT ATGTGGCTTA ATTCGACTCA ACGCGCAATA ACTTACTTGG TCCGAACGCT TTGAGGATTG ACAGTT---- -TCTGGTACA
T. quinqueloba Ib     GCACCACAAG AGCGTGGAGT ATGTGGCTTA ATTCGACTCA ACGCGCAATA ACTTATTTGG TCCGAACGCT TTGAGGATTG ACAGTT---- -TCTGGTACA
T. quinqueloba IIa    GCACCACAAG AGCGTGGAGT ATGTGGCTTA ATTCGACTCA ACGCGCAACA ATTTACTTGG TCCGAACGCT TTGGGGATTG ACAGTT---- -ATTGTATAG
T. quinqueloba IIb    GCACCACAAG AGCGTGGAGT ATGTGGCTTA ATTCGACTCA ACGCGCAACA ATTTACTTGG TCCGAACGCT TTGAGGATTG ACAGTT---- -ATTGTATAG
T. quinqueloba IIc    GCACCACAAG AGCGTGGAGT ATGTGGCTTA ATTCGACTCA ACGCGCAACA ATTTACTTGG TCCGAACGCT TTGAGGATTG ACAGTT---- -ATTGTATAG
T. quinqueloba IId    GCACCACAAG AGCGTGGAGT ATGTGGCTTA ATTCGACTCA ACGCGCAACA ATTTACTTGG TCCGAACGCT TTGAGGATTG ACAGTT---- -ATTGTATAG

                      101        111        121        131        141        151        161        171        181        191       200
                      |          |          |          |          |          |          |          |          |          |         |
T. quinqueloba Ia     ATGTGCGGCT TGTTTGTTAC AACTACGAAT GATTCGAATT GTTGTAAATA TGACTTGGCC GGCCTTCGGG TGTCTTGGAT CGG-CGTATC AGGTCGTACA
T. quinqueloba Ib     ATGTGCGGCT TGTTTGTTAC AACTGCGAAT GACTCGATTT GTTGTAAATA TGACTTGACC GGCCTTCGGG TGTCTTGGAT GGGTCGTATC AGGTCGTACA
T. quinqueloba IIa    TTCTGATATG AGAGGTCTTT GTAGTCAACG TGTAGGTAGT TGTA------ ---------- ---------- ---------- ---------- ----------
T. quinqueloba IIb    TTCTGATATG GGTGGTATTT GTAGTCAACG TGTAGGTAGT TGTA------ ---------- ---------- ---------- ---------- ----------
T. quinqueloba IIc    TTCTGATATG GGTGGTATTT GTAGTCAACG TGTAGGTAGT TGTA------ ---------- ---------- ---------- ---------- ----------
T. quinqueloba IId    TTCTGATATG GGTGGTATTT GTAGTCAACG TGTAGGTAGT TGTA------ ---------- ---------- ---------- ---------- ----------
```

```
                        201        211        221        231        241        251        261        271        281        291      300
T. quinqueloba Ia       TTG-----TA TTCAATGAGA AAGTTCTTTT ATGATTATGT GGTAGGTGGT GCATGGCCGT CTTTAATTCG TGGAGTGATC TGTCTGCTTA ATTGCGCATT
T. quinqueloba Ib       TTG-----TA TTCAATGAGA AAGTTCTTTT ATGATTATGT GGTAGGTGGT GCATGGCCGT CTTTAATTCG TGGAGTGATC TGTCTGCTTA ATTGCGCATT
T. quinqueloba IIa      --------TA TTAAATATGA AAGTTCTTTT ATGATTATGT GATAGGTGGT GCATGGCCGT CCTTAATTCG TGGAGTGATT TGTCTGCTTA ATTGCGCATT
T. quinqueloba IIb      --------TA TTAAATATGA AAGTTCTTTT ATGATTATGT GATAGGTGGT GCATGGCCGT CCTTAATTCG TGGAGTGATT TGTCTGCTTA ATTGCGCATT
T. quinqueloba IIc      --------TA TTAAATATGA AAGTTCTTTT ATGATTATGT GATAGGTGGT GCATGGCCGT CCTTAATTCG TGGAGTGATT TGTCTGCTTA ATTGCGCATT
T. quinqueloba IId      --------TA TTAAATATGA AAGTTCTTTT ATGATTATGT GATAGGTGGT GCATGGCCGT CCTTAATTCG TGGAGTGATT TGTCTGCTTA ATTGCGCATT

                        301        311        321        331        341        351        361        371        381        391      400
T. quinqueloba Ia       GCAAATTGTA ATTGATCT-- ---TTGAACA GATCCGTCTT --TTTATGTT G--AATAGTA CGTACAGTCA GACCCGGTTG GGCATGTACG GAAAGGTCAC
T. quinqueloba Ib       GCAAATTGTA ATTAATCT-- ---GGCTTGG ATGATCGTTT GATTTCGATT TGAATGCCAT TTCTTTTTTC GAGAATG--- ---------- ----------
T. quinqueloba IIa      GCAAATTGTA ATTGATCT-- ---TTTACCA GTCTCGTTCC GATTGATGTT GTGAATATTG TAGAATATTG TTGTCGTCGA GTCATAATTG CCACTACGTC
T. quinqueloba IIb      GCAAATTGTA ATTGATCT-- ---TTTACCA GTCTCGTTCT GATTTATGTT GT-AATATTG TAGAATATTG TTGTCGTCGA GTCATTATTG CCACTACGTC
T. quinqueloba IIc      GCAAATTGTA ATTGATCT-- ---TTTACCA GTCTCGTTCT GATTTATGTT GT-AATATTG TAGAATATTG TTGTCGTCGA GTCATTATTG CCACTACGTC
T. quinqueloba IId      GCAAATTGTA ATTGATCT-- ---TTTACCA GTCTCGTTCT GATTTTTGTT GTGAATATTG TAGAATATTG TTGTCGTCGA GTCATTATTG CCACTACGTC

                        401        411        421        431        441        451        461        471        481        491      500
T. quinqueloba Ia       ATATAAAGTA CGGTCTGCTA AAGATATGAA TA-------- ---------- ---------- ---------- ---------- ---------- ----------
T. quinqueloba Ib       ---------- ---------- ---------- ---------- ---------- ---------- ---------- ---------- ---------- ----------
T. quinqueloba IIa      TGGTCCTTCG GG--CAGAAT AGTCCTTATG CTTGTCT--- ----ACACAA AATTCTCCAC AATGTTGTAG CCATACTTGA TTGTATGCGC TATTAATGTC
T. quinqueloba IIb      TGGTCCTTTG GGGGCAGAAT AGTGTTGTAT ATTTTGTCTT GTCAACACAA AATTCTTCAC AATATTGTAG CCGTACTTGA TTGTATGCGC TATTCATGTA
T. quinqueloba IIc      TGGTCCTTTG GG--CAGAAT AGTGTTGTAT ATTTTGTCTT GTCAACACAA AATTCTTCAC AATATTGTAG CCGTACTTGA TTGTATGCGC TATTCATGTA
T. quinqueloba IId      TGGTCCTTTG GG--CAGAAT AGTGTTTTAT ATTTTGTCTT G-CAACACAA AATTCTTCAC AATATTGTAG CCGTACTTCA TTGTATGCGC TATTCATGTA

                        501        511        521        531        541        551        561        571        581        591      600
T. quinqueloba Ia       ---------- ---------- ---------- ---------- ---------- ---------- ---------- ---------- ---------- --T-----CT
T. quinqueloba Ib       ---------- ---------- ---------- ---------- ---------- ---------- ---------- ---------- ---------- --A-----CT
T. quinqueloba IIa      TATGTTGGAC ACATTCGTGG TTCAAGACCA GTTCGGCTGC TTGCACACAT TGCCATCTCA ACATGATTGG TCAGGCTGCT AGAGACCTTG TGT-----CT
T. quinqueloba IIb      TATGTG--GT TAGT------ --CAACACCA GTTCGGCTGC TTGCACACAT CGCCATCTCA ACATGATCAG TCAGGCTGCT AGAGACCTTG TGT-----CT
T. quinqueloba IIc      TATGTG--GT TAGT------ --CAACACCA GTTCGGCTGC TTGCACACAT CGCCATCTCA ACATGATCAG TCAGGCTGCT AGAGACCTTG TGT-----CT
T. quinqueloba IId      TATGT--GAC ACATTTGTGG TTCAAGACCA GTTCGGCTGC TTGTACACAT ATACACTCGA -CATGATCAT TCAGGCTGCT AGAGACCTTG TGT-----CT

                        601        611        621        631        641        651        661        671        681        691      700
T. quinqueloba Ia       TGCCCTAAAG ATTGATTAAC ACCGTGAGTG CAACGAGTGA GATTGCGAGT CTTTGTTATG TAGTGCTCAA CATA--CCTA ---------- ----------
T. quinqueloba Ib       TGCCCTAAAG ATTGATTAAC ACCGTGAGTG CAACGAGTGA GATTGCGAGT CTTTGTTATG TAGTGCTCAA CATA--CCTA ---------- ----------
T. quinqueloba IIa      TGCCCCAAAG GTTGATTAAC ACCGTGAGTG CAACGAGTGA GATTGCAAGT CTTTGTTATG TAGTGAACAA CATATACCTA ---CTACT-C ----------
T. quinqueloba IIb      TGCCCCAAAG GTTGATTAAC ACCGTGAGTG CAACGAGTGA GATTGCAAGC CTTTGTTATG TAGTGAACAA CATATACCTA ---CTACTAC TACTA---TT
T. quinqueloba IIc      TGCCCCAAAG GTTGATTAAC ACCGTGAGTG CAACGAGTGA GATTGCAAGC CTTTGTTATG TAGTGAACAA CATATACCTA ---CTACTAC TACTT-----
T. quinqueloba IId      TGCCCCAAAG GTTGATTAAC ACCGTGAGTG CAACGAGTGA GATTGCAAGC CTTTGTTATG TAGTGAACAA CATATACCTA ---CTACTAC TACTACTACT

                        701        711        721        731        741        751        761        771        781        791      800
T. quinqueloba Ia       -TGTGCAGT- -----AGGAT TT-AACTACA CC--ATGTAA CCTTTTTCA- --AAGGTTGA ATGAACTTAG GCGACTGCTA TACTTTTAAG ATGGTCGAAG
T. quinqueloba Ib       -TGTGCAGT- -----AGGAT TT-AACTACA CC--ATGTAA CCTTTTTCA- --AAGGTTGA ATGAACTTAG GCGACTGCTA TACTTTTAAG ATGGTCGAAG
T. quinqueloba IIa      ---TAGTAGT -----AGGAT TCCAACTACA CA--GTATAA CCATT----- --AAGGTGAA ATGAACTCAG GCGACTGCTA TACTT-TAAG ATGGTGGAAG
T. quinqueloba IIb      TTGTAGTAAT GT---AGGAT TCCAACTACA CA--GAATAA CCATT----- --AAGGTGAA ATGAACTCAG GCGACTGCTA TACTT-TAAG ATGGTGGAAG
T. quinqueloba IIc      -TGTAGTAAT GT---AGGAT TCCAACTACA CACAGAATAA CCATT----- --AAGGTGAA ATGAACTCAG GCGACTGCTA TACTT-TAAG ATGGTGGAAG
T. quinqueloba IId      TTGTAGTAAT GT---AGGAT TCCAACTACA CA--GAATAA CCATT----- --AAGGTGAA ATGAACTCAG GCGACTGCTA TACTT-TAAG ATGGTGGAAG

                        801        811        821        831        841        851        861        871        881        891      900
T. quinqueloba Ia       GTTGTGGCAA TGACAGGTCT GTGATGCCC- TTAGATGTTC AGGGCTGCAC ACGTACTACA TTGATCTAGT CAATAAGTAT GTGTGTAACA -----ATA-A
T. quinqueloba Ib       GTTGTGGCAA TGACAGGTCT GTGATGCCC- TTAGATGTTC AGGGCTGCAC ACGTACTACA TTGATCTAGT CAATAAGTAT GTGTGTAACA -----ATA-A
T. quinqueloba IIa      GTTGTGGCAA TGACAGGTCT GTGATGCCCT TTAGATGTTC AAGGCTGCAC ACGTACTACA TTGATCTAGT CAACGAGTAT GTATGTAACA -----TTGAA
T. quinqueloba IIb      GTTGTGGCAA TGACAGGTCT GTGATGCCCT TTAGATGTTC AAGGCTGCAC ACGTACTACA TTGATCTAGT CAACGAGTAT GTATGTAACA -----TTG-A
T. quinqueloba IIc      GTTGTGGCAA TGACAGGTCT GTGATGCCCT TTAGATGTTC AAGGCTGCAC ACGTACTACA TTGATCTAGT CAACGAGTAT GTATGTAACA -----TTG-A
T. quinqueloba IId      GTTGTGGCAA TGACAGGTCT GTGATGCCCT TTAGATGTTC AAGGCTGCAC ACGTACTACA TTGATCTAGT CAACGAGTAT GTATGTAACA -----TTG-A

                        901        911        921        931        941        951        961        971        981        991     1000
T. quinqueloba Ia       TTTT---GAA TGTATTGGTT AAGC---TTT T-CTTATATT TTGGTAAGAG -----GTTAA TACAGAACTT CGAGAGAGTT CCGACAACAG TCAATCTAGT
T. quinqueloba Ib       TTTT---GAA TGTATTGGTT AAGC---TTT T-CTTATATT TTGGTAAGAG -----GTTAA TACAGAACTT CGAGAGAGTT CCGACAACAG TCAATCTAGT
T. quinqueloba IIa      TAAT---GAA TGTATTGGTT AAGC---TAT ATTGTATTTC --GCTAA--C -----GTTAA TTCAGAACTT CGAGAAGGTT CCGACAACAG TCAATCTAGT
T. quinqueloba IIb      TTTT---GCA TCTATTGGTT AAGC---TAT AATGTATTTC --GGTAA--C -----GTTAA TTCAGAACTT CGAGAAGGTT CCGACAACAG TCAATCTAGT
T. quinqueloba IIc      TTAT---GCA TCTATTGGTT AAGC---TAT AATGTATTTC --GGTAA--C -----GTTAA TTCAGAACTT CGAGAAGGTT CCGACAACAG TCAATCTAGT
T. quinqueloba IId      TTAT---GCA TCTATTGGTT AAGC---TAT AATGTATTTC --GGTAA--C -----GTTAA TTCAGAACTT CGAGAAGGTT CCGACAACAG TCAATCTAGT

                       1001       1011       1021       1031       1041       1051       1061       1071       1081       1091     1100
T. quinqueloba Ia       TATTGCTTGT AGTCG----- --ATCT---- CATGATTGTT TAGTTTTATG CAAC----TT CGGGTCATGA TTCACTAGTA CGCTTC---T AATTCACAGT
T. quinqueloba Ib       TATTGCTTGT AGTCG----- --ATCT---- CATGATTGTT TAGTTTTATG CAAC----TT CGGGTCATGA TTCACTAGTA CGCTTC---T AATTCACAGT
T. quinqueloba IIa      TATTGCTTGT AGTCG----- --ATTG---- CATAATTGTA CAGTT-CACG CAAC---GCT CGG-TTATCA TTCGCTAGTA --TTGT---T AATTCACAGT
T. quinqueloba IIb      TATTGCTTGT AGTTG----T TAATTG---- TATAATTGTC TAGTT-CACG CAAC---GTT CGT-TTATCA TTCTCTAGTA --TTGT---T AATTCACAGT
T. quinqueloba IIc      TATTGCTTGT AGTTG----T TAATTG---- TATAATTGTC TAGTT-CACG CAAC---GTT CGT-TTATCA TTCTCTAGTA --TTGT---T AATTCACAGT
T. quinqueloba IId      TATTGCTTGT AGTTG----T TAATTG---- TATAATTGTT TAGTT-CACG CAAC---GTT CGT-TTATCA TTCTCTAGTA --TTGT---T AATTCACAGT

                       1101       1111       1121       1131       1141       1151       1161       1171       1181       1191     1200
T. quinqueloba Ia       GGGGACAGTC GTTTGTAATT CTGAGACTCG GTTCAACTAG GAATGCCTAG TATTGATGGT TCACTAAACT TTCTGGAATA AGTCCCTGCC CTTTGTACAC
T. quinqueloba Ib       GGGGACAGTC GTTTGTAATT CTGAGACTCG GTTCAACTAG GAATGCCTAG TATTGATGGT TCACTAAACT TTCTGGAATA AGTCCCTGCC CTTTGTACAC
T. quinqueloba IIa      GGGGACAGTC GTTTGTAATT CTGAGACTCG GTTCAACCAG GAATGCCTCG TATTTGTGGT TCATTATACT CCGTGGAATA AGTCCCTGTC CTTTGTACAC
T. quinqueloba IIb      GGGGACAGTC GTTTGTAATT CTGAGACTCG GTTCAACCAG GAATGCCTCG TATTTGTGGT TCATTAAACT CCGTGGAATA AGTCCCTGTC CTTTGTACAC
T. quinqueloba IIc      GGGGACAGTC GTTTGTAATT CTGAGACTCG GTTCAACCAG GAATGCCTCG TATTTGTGGT TCATTAAACT CCGTGGAATA AGTCCCTGTC CTTTGTACAC
T. quinqueloba IId      GGGGACAGTC GTTTGTAATT CTGAGACTCG GTTCAACCAG GAATGCCTCG TATTTGTGGT TCATTAAACT CCGTGGAATA AGTCCCTGTC CTTTGTACAC
```

```
                       1201       1211       1221       1231       1241       1251       1261       1271       1281       1291      1300
                       |          |          |          |          |          |          |          |          |          |         |
T. quinqueloba Ia      ACCGCCCGTC GCTTTTACCA ATGGCCCTCG TTGTGAGTGA GCTGGACAAG -----TTATT TTA-----AC TTGAAAAGTT CTCAATCAAG GTTTGCTAAA
T. quinqueloba Ib      ACCGCCCGTC GCTTTTACCA ATGGCCCTCG TTGTGAGTGA GCTGGACAAG -----TTATT TTA-----AC TTGAAAAGTT CTCAATCAAG GTTTGCTAAA
T. quinqueloba IIa     ACCGCCCGTC GCTTTTACCA ATGGCCCTCG TTGTGAGATA GCTGGACAAG -----TATTT ACT-----AC TTCAAAAGTT CTCAATCAAG GTTTGCTAAA
T. quinqueloba IIb     ACCGCCCGTC GCTTTTACCA ATGGCCCTCG TTGTGAGATA GCTGGACAAG -----TATTT ACT-----AC TTCAAAAGTT CTCAATCAAG GTTTGCTAAA
T. quinqueloba IIc     ACCGCCCGTC GCTTTTACCA ATGGCCCTCG TTGTGAGATA GCTGGACAAG -----TATTT ACT-----AC TTCAAAAGTT CTCAATCAAG GTTTGCTAAA
T. quinqueloba IId     ACCGCCCGTC GCTTTTACCA ATGGCCCTCG TTGTGAGATA GCTGGACAAG -----TATTT ACT-----AC TTCAAAAGTT CTCAATCAAG GTTTGCTAAA

                       1301       1311
                       |          |
T. quinqueloba Ia      GGAAAAAGAA GTCGTAAC
T. quinqueloba Ib      GGAAAAAGAA GTCGTAAC
T. quinqueloba IIa     GGAAAAAGAA GTCGTAAC
T. quinqueloba IIb     GGAAAAAGAA GTCGTAAC
T. quinqueloba IIc     GGAAAAAGAA GTCGTAAC
T. quinqueloba IId     GGAAAAAGAA GTCGTAAC
```
